# Photogeneration of α-Bimetalloid Radicals via Selective Activation of Multifunctional C1 Units

**DOI:** 10.1021/jacs.4c02261

**Published:** 2024-05-28

**Authors:** Lewis McGhie, Alessandro Marotta, Patrick O. Loftus, Peter H. Seeberger, Ignacio Funes-Ardoiz, John J. Molloy

**Affiliations:** † Department of Biomolecular Systems, Max-Planck-Institute of Colloids and Interfaces, Potsdam 14476, Germany; ‡ Department of Chemistry and Biochemistry, Freie Universität Berlin, Berlin 14195, Germany; § Department of Chemistry, Instituto de Investigación Química de la Universidad de La Rioja (IQUR), Universidad de La Rioja Madre de Dios 53, Logroño 26004, Spain

## Abstract

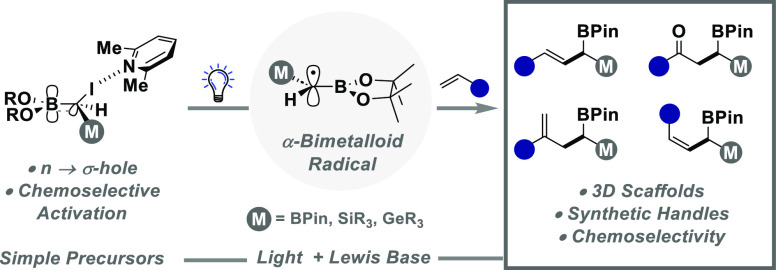

Light-driven strategies that enable the chemoselective activation of a specific bond in multifunctional systems are comparatively underexplored in comparison to transition-metal-based technologies, yet desirable when considering the controlled exploration of chemical space. With the current drive to discover next-generation therapeutics, reaction design that enables the strategic incorporation of an sp^3^ carbon center, containing multiple synthetic handles for the subsequent exploration of chemical space would be highly enabling. Here, we describe the photoactivation of ambiphilic C1 units to generate α-bimetalloid radicals using only a Lewis base and light source to directly activate the C–I bond. Interception of these transient radicals with various SOMOphiles enables the rapid synthesis of organic scaffolds containing synthetic handles (B, Si, and Ge) for subsequent orthogonal activation. In-depth theoretical and mechanistic studies reveal the prominent role of 2,6-lutidine in forming a photoactive charge transfer complex and in stabilizing *in situ* generated iodine radicals, as well as the influential role of the boron p-orbital in the activation/weakening of the C–I bond. This simple and efficient methodology enabled expedient access to functionalized 3D frameworks that can be further derivatized using available technologies for C–B and C–Si bond activation.

## Introduction

**Figure 1:**
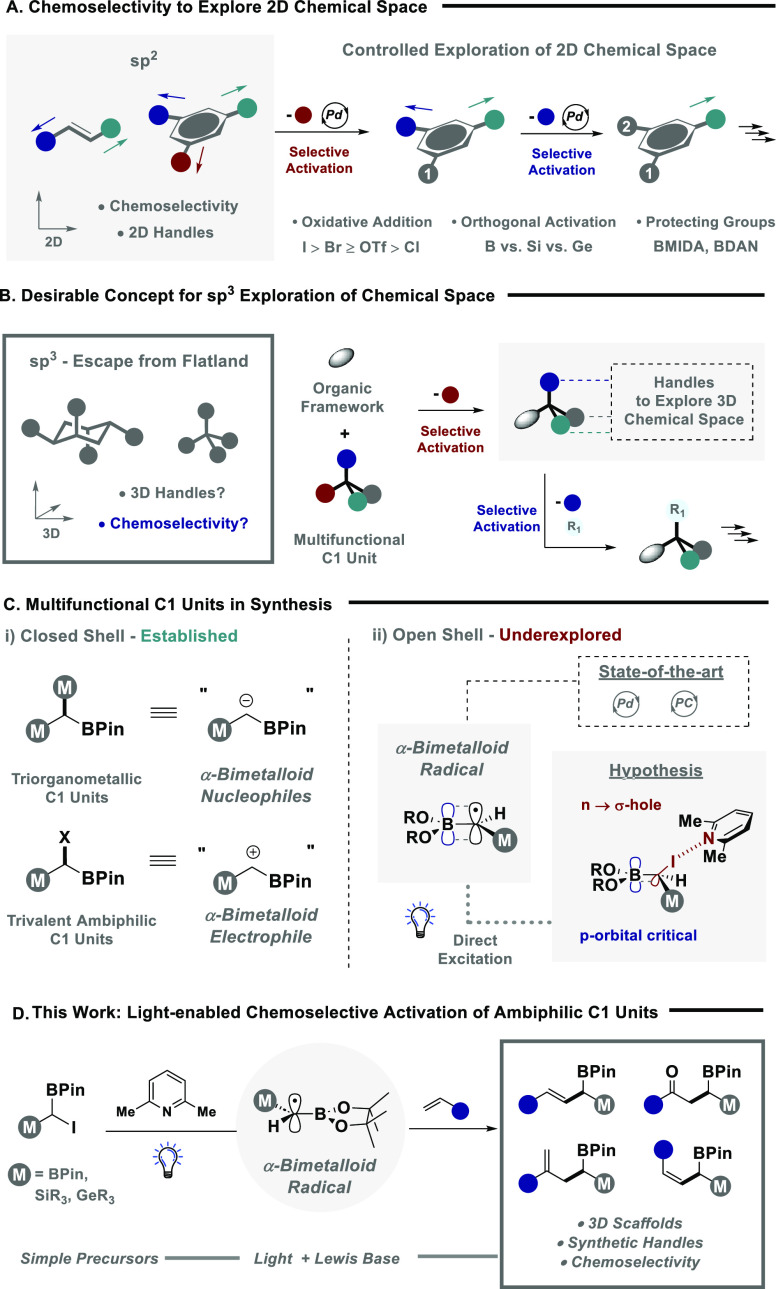
(A) Chemoselective exploration of 2D chemical space using Pd catalysis. (B) Concept to explore 3D chemical space. (C) Multifunctional C1 units in synthesis. (D) Light-enabled generation of α-bimetalloid radicals.

The rational design and construction of molecules that target a specific biological function remain a core construct in the discovery of next-generation therapeutics.^1^ In contemporary medicinal chemistry, there is an overwhelming reliance on synthetic tools that facilitate the rapid and efficient exploration of chemical space in a strategically controlled manner.^2^ In this regard, palladium-catalyzed cross-coupling reactions have been revolutionary, where preinstalled metals/metalloids or (pseudo)halides are leveraged as sp^2^ exit vectors enabling the efficient extension into 2D chemical space (Figure 1A).^3^ The translation to multifunctional platforms has seen the inception of chemoselective activation strategies, exploiting differences in bond dissociation energy for oxidative addition (I > Br ≥ OTf > Cl),^4^ and orthogonal reactivity of pendant nucleophiles for selective engagement in transmetalation (B vs Si vs Ge).^5^ The utility of boron-protecting groups has further expanded this concept to the iterative and automated synthesis of complex molecules,^6,7^ and given this impact, it serves as no great surprise that an estimated greater than 40% of all C–C bond formations in medicinal chemistry are currently achieved via the Suzuki–Miyaura cross-coupling reaction.^8^

Given the current drive to “escape from flatland” in the design of novel pharmaceuticals,^9^ multifunctional sp^3^ systems where the described orthogonal reactivity could be emulated would be highly enabling when considering the synthesis of molecules that occupy 3D chemical space (Figure 1B). However, while selective functionalization of a multifunctional C1 unit is desirable, moving from positionally distinct synthetic handles (sp^2^) to unifying them on a single atom (sp^3^) coincides with constraints on intrinsic reactivity due to a direct influence on electronic and steric parameters.^10^

Classic solutions to overcome this obstacle include deprotonation and harnessing reactive organometallics to generate α-bimetalloid anion synthetic equivalents (Figure 1C).^11^ These reactive intermediates are still frequently employed and have been leveraged in elegant strategies for mono- or chemoselective activation (M = Li, B, Si) enabling efficient reactivity with various electrophiles,^12^ and application in transition metal-catalyzed cross-coupling.^13^ The recent emergence of trivalent ambiphilic C1 units that contain at least one pendant C–X bond has enabled extension to α-bimetalloid electrophile synthetic equivalents (Figure 1C),^14^ where selective engagement of the C–X bond is achieved via exposure to nucleophilic species such as amines, and organometallics.^14a,15^ However, while closed shell reactivity has been comparatively well established and continues to expand, synthetic tools that enable the efficient generation of an α-bimetalloid radical are conspicuously underexplored, yet desirable given their orthogonal reactivity to closed shell paradigms. The current state of the art requires palladium and light as a prerequisite for efficient selective activation,^14a,14c^ or photoredox catalysts to grant expedient access to cyclopropyl scaffolds.^16^ Motivated by the untapped potential of boron hybridization on photochemical processes,^17^ we envisaged that trivalent ambiphilic C1 units, containing a trigonal planar boron moiety,^18,19^ could serve as potent precursors for the operationally simple, light-enabled generation of α-bimetalloid radicals, providing a complementary approach to existing organometallic and transition metal technologies (Figure 1C, right). Here, it was anticipated that the boron p-orbital could modulate electron density at the C–I bond, resulting in a buildup of positive charge in the σ-hole of the iodine atom and ultimately a weakening of the bond. This bond could then be selectively targeted using a Lewis basic additive to generate a halogen bonding charge transfer complex that can absorb light.^20,21^ Absorption of a photon would facilitate the selective photoinduced homolytic cleavage of the C–I bond in the presence of metalloids (B, Si, Ge) that are commonly cleaved via photoactivation.^14a,22^ This would then generate an iodine radical stabilized by the Lewis base,^23,24^ and an α-bimetalloid radical primed for subsequent reactivity.

Herein, we describe the operationally simple in situ generation of α-bimetalloid radicals from easily accessible ambiphilic precursors using only light and a simple 2,6-lutidine additive (Figure 1D). Interception of these transient radicals with a series of SOMOphiles enables the rapid construction of versatile frameworks that contain multiple synthetic handles (B, Si, Ge) for the subsequent exploration of the 3D chemical space. In-depth mechanistic and computational studies unveil the importance of both the boron p-orbital and Lewis base for efficient reactivity, with the Lewis base serving a dual role in both photoactivation of the C–I bond via halogen bonding and stabilization of the iodine radical. The mild, catalyst-free protocol could be strategically paired with energy transfer catalysis to grant unprecedented access to *Z*-isomers containing two functional handles, rendering the process stereodivergent. The power of the overall construct for the efficient exploration of chemical space was demonstrated by product derivatization via the chemoselective activation of specific synthetic handles.

## Results and Discussion

We commenced our reaction optimization by investigating the efficiency of radical generation using substrate **1a**, containing both boron and silicon handles, in the presence of styrene **2** and a Lewis basic additive (Table 1). Implementing 2,6-lutidine as an additive under 370 nm (Kessil, 40 W) light irradiation afforded the allylic product *E***-3** in an appreciable yield and selectivity (entry 1). The use of lower energy photons was beneficial using 390 nm, suppressing competitive degradation (entry 2). However, conversion to product was unsatisfactory when employing 427 nm irradiation (entry 3).

**Table 1:**
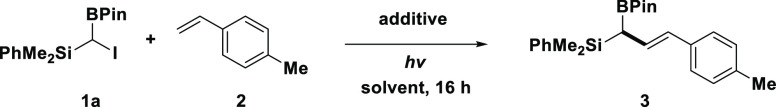
Optimization of Reaction Conditions^a^

entry	λ (nm)^b^	solvent	additive	yield (%)	*E*:*Z*^c^
1	370	MeCN	2,6-lutidine	37	86:14
2	390	MeCN	2,6-lutidine	69	89:11
3	427	MeCN	2,6-lutidine	43	86:14
4	390	MeCN	Et_3_N	0	n.d.
5	390	MeCN	pyridine	28	79:21
6	390	MeCN	PPh_3_	20	>95:5
7	390	MeCN	2,6-dimethyl-4-(dimethylamino)pyridine	46	74:26
8	390	THF	2,6-lutidine	3	n.d.
9	390	DMF	2,6-lutidine	0	n.d.
10	390	MeCN		0	n.d.
11		MeCN	2,6-lutidine	0	n.d.

a Standard conditions: **1a** (0.2 mmol), **2** (3 equiv), additive (1.5 equiv), solvent (0.05 M), rt 16 h.

b Reactions run using Kessil lamps 40 W.

c Determined by ^1^H NMR spectroscopy against a known internal standard (1,3,5-trimethoxybenzene).

The use of alternative Lewis basic additives such as triethylamine, pyridine, and triphenylphosphine identified that 2,6-lutidine was required for efficient reactivity (entries 4–6). Increasing electron density on the Lewis base backbone was found to be detrimental to reactivity and selectivity (entry 7), while the reaction was also found to be suppressed in Lewis basic solvents (entries 8 and 9). These preliminary reactions indicate that intricate control of steric parameters and Lewis basicity of both the additive and reaction media is critical to mitigate undesired polar reactivity with the boron p-orbital (for a comprehensive list of trialed Lewis basic additives, please see ESI for full details).^25^ Control reactions highlight that both additive and light are required for efficient reactivity (entries 10 and 11). It is pertinent to note in both control reactions close to full recovery of substrate **1a** was achieved, indicating that both light and Lewis base are required for efficient activation of the C–I bond and that the developed method is orthogonal to ground-state reactivity previously established in the literature (Figure 1c).^11−15^

**Scheme 1:**
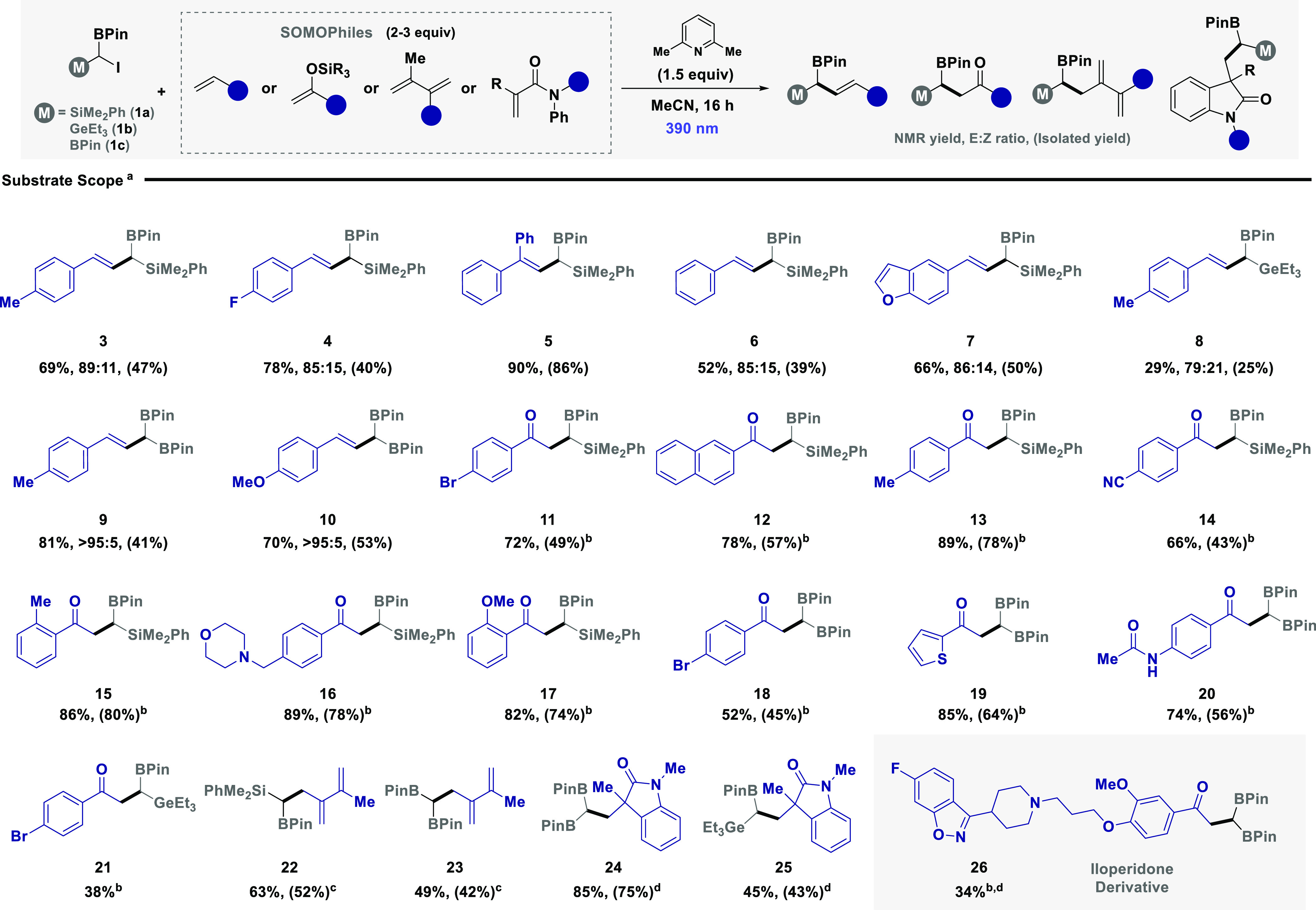
Establishing the Substrate Scope (a) Reactions were performed in MeCN on a 0.2 mmol scale using **1** (1 equiv), SOMOphile (3 equiv), and 2.6-lutidine (1.5 equiv) under 390 nm irradiation (40 W). NMR yield and *E*:*Z* ratio were determined by ^1^H NMR spectroscopy against a known internal standard (1,3,5-trimethoxybenzene). (b) 20 equiv of water was added. (c) 427 nm light was used for 48 h. (d) 2 equiv of SOMOphile was used.

With efficient radical generation and a general set of reaction conditions established, the scope and compatibility of the developed protocol were assessed for a series of diverse SOMOphiles (Scheme 1). Ambiphilic reagent **1a** was efficiently coupled with styrene SOMOphiles to afford synthetically versatile *E*-allylic products containing a pendant boron and silicon handle, in appreciable yield and good selectivity (**3–7**). Translation to ambiphilic reagent **1b**,^14a^ was comparatively unsuccessful (**8**), due to *in situ* degradation of the highly reactive allylic product under model reaction conditions. Pleasingly, ambiphilic reagent **1c**, containing *geminal*-BPin substituents, was amenable to the protocol, enabling the rapid construction of *E*-allylic boronic esters in good selectivity (**9** and **10**). With a slight modification of the model reaction conditions, silyl enol ethers could also be leveraged as effective SOMOphiles to enable access to β-substituted ketones. Utilizing reagent **1a**, the reaction was tolerant of halides (**11**), enabling an additional handle for subsequent reactivity. Electron-neutral (**12**, **13**, and **15**), electron-poor (**14**), and electron-rich (**17**) silyl enol ethers were also tolerated in good yield. Intriguingly, aliphatic amines, typically prone to oxidation under photoredox reactivity,^26^ were cleanly transformed to the target ketone (**16**). Alternative iodides **1b** and **1c** were also effective in engaging silyl enol ethers furnishing substrates **18–21** in moderate to good yield. The target reactivity was extended to diene SOMOphiles, to construct Diels–Alder compatible dienes containing boron and silicon as synthetic linchpins (**22** and **23**). The synthesis of oxindole derivatives (**24** and **25**) was achieved via a tandem process employing a phenyl-substituted acrylamide SOMOphile. Finally, the silyl enol ether derivative of iloperidone, a potent antipsychotic therapeutic,^27^ was easily transformed to the corresponding ketone containing two boron handles for further downstream synthetic manipulations (**26**).

**Figure 2:**
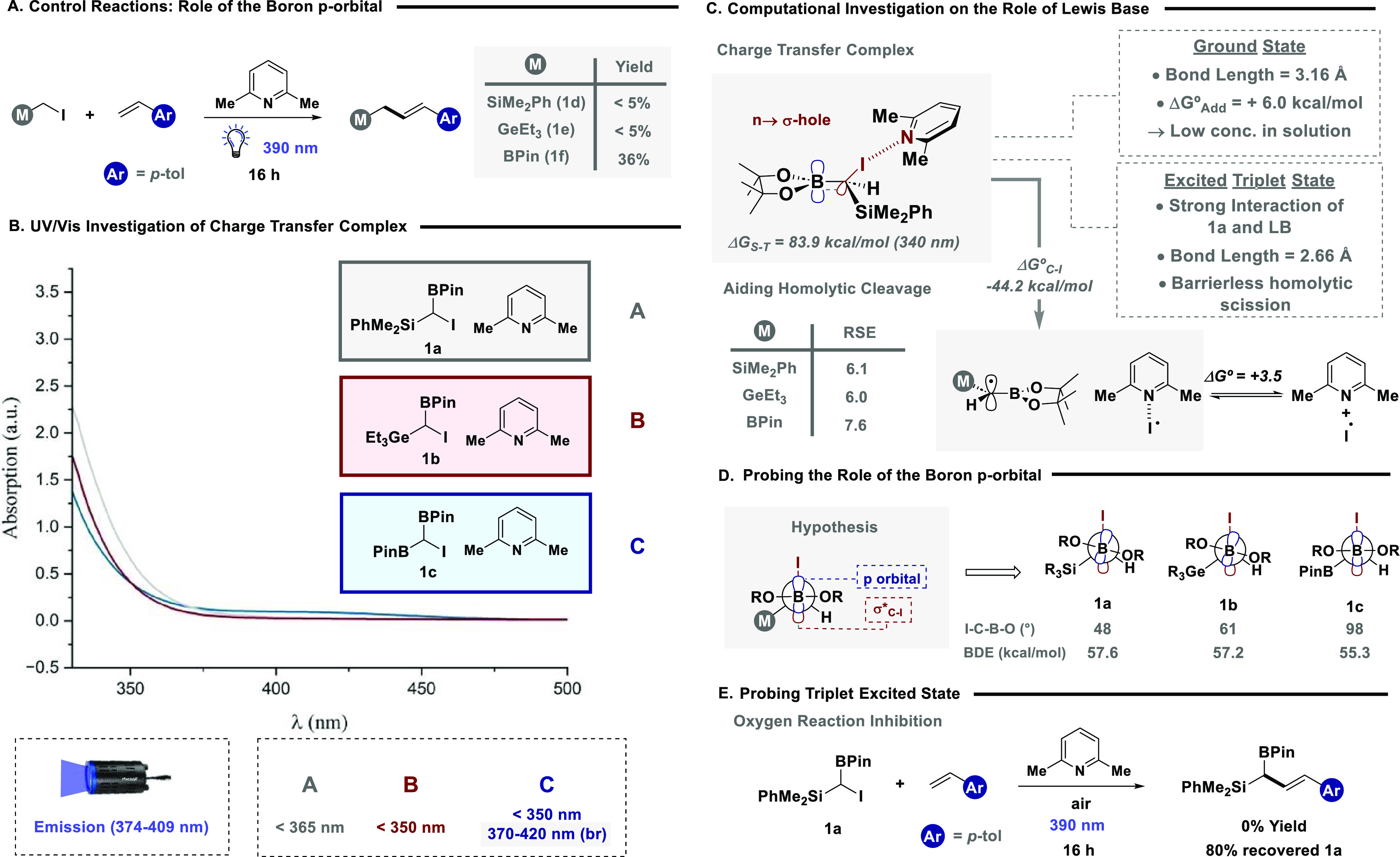
(A) Probing the role of the boron p-orbital for efficient reactivity. (B) UV/vis investigation on charge transfer complex formation. (C) Computational investigation on the role of the Lewis base. (D) Probing the role of the boron p-orbital. (E) Reaction inhibition by inclusion of oxygen.

Given that the developed protocol mitigates the use of a photocatalyst or transition metal for efficient activation, we next set out to determine the *modus operandi* and key factors for the observed selective light-driven reactivity. As highlighted previously, the judicious choice of 2,6-lutidine was required for efficient reactivity, with alternative Lewis bases and inorganic bases proving ineffective (see ESI for full details). This is indicative that 2,6-lutidine, *inter alia*, is a key component for efficient radical generation. To probe our preliminary hypothesis that the trigonal planar boron p-orbital is also required, we assessed iodides without an adjacent boron substituent (Figure 2A). Utilizing α-silyl and α-germanyl substrates **1d** and **1e** respectively led to no observed reactivity with complete retention of starting materials. However, employing the analogous α-boryl substituent, reactivity was restored underpinning the pivotal role of the boron p-orbital for efficient C–I bond activation. UV/vis analysis of ambiphilic starting materials (**1a–1c**), with solutions prepared in the dark at model reaction concentrations, determined that the precursors did not absorb the light of the incident Kessil lamp (see ESI for full details), while control reactions in the absence of additive (Table 1, entry 12) also support that efficient direct excitation of the reagents is not feasible. The inclusion of 2,6-lutidine led to a subtle alteration in absorption properties for both **1a** and **1b** leading to a small bathochromic shift (10 nm), while **1c** developed a broad band ranging from 370 to 420 nm (Figure 2B). An increase in concentration also led to a further bathochromic shift to longer wavelengths (10–20 nm; see ESI for full details). ^1^H and ^11^B NMR analysis of reaction components showed no notable sign of the previously envisioned halogen-bonding charge transfer complex and no dative interaction with the boron p-orbital to form a negatively charged boronate. However, when preparing solutions of both ambiphilic reagent and additive in the presence of light irradiation (390 nm, 20 min), small concentrations of triiodide were observed supporting light-induced homolytic cleavage of the C–I bond for all three precursors.^28^ At this stage of the study, the origin of light-driven activation proved elusive and challenging to rationalize given that no clear precursor or intermediate was determined to be efficiently photoactive.

To interrogate the underlying origin of activation, a computational study of the model system was conducted at the SMD(MeCN) ωB97xD/def2TZVPP//ωB97xD/def2SVP level of theory (Figure 2C). We initiated our study by probing the feasibility of halogen bonding between ambiphilic reagents **1a** and 2,6-lutidine in both the ground state and excited state (see the ESI for full details).

Pleasingly, the formation of an n → σ-hole charge transfer complex was shown to be energetically feasible with an estimated halogen bond length of 3.16 Å (see further details in the Supporting Information, Figures S36 and S37). We compare this interaction with the alternative formation of a boronate complex via the dative interaction of the Lewis base with the boron p-orbital. However, this interaction is not possible as the potential energy increases when the N center approximates the boron center (see ESI for full details). Predicted UV/vis of the charge transfer complex indicated that a new band with a bathochromic shift is generated, and the tail has significant overlap with the Kessil lamp at reaction concentration. However, with a relatively low NBO value for the σ-hole and an energy of formation established at 6.0 kcal/mol, it is foreseeable that a complex of this nature likely exists in low concentrations in solution, resulting in challenging detection by standard spectroscopic tools (UV/vis and NMR).^29^

On translation to the triplet excited state, again a clear adduct between **1a** and 2,6-lutidine could be modeled with a shortened bond length of 2.66 Å. This structure shows a perpendicular interaction that comes from the partial C–I bond breaking, WBI (Wiberg Bond Index) = 0.2440, and N–I bond formation, WBI = 0.2045. Subsequent homolytic bond scission of the C–I bond in the triplet excited state was found to be highly energetically favored (−44.2 kcal/mol) through an almost barrierless conical intersection between the very close triplet electronic surfaces (<2 kcal/mol), estimated from the relaxed scan of the PES in both states (see ESI for full details). This generates comparatively stabilized α-bimetalloid radicals (RSE between 6.0 and 7.6 kcal/mol) and an iodine radical that is further stabilized by coordination to 2,6-lutidine (3.5 kcal/mol), due to the strong donor–acceptor interaction between the N lone pair and the partially occupied p orbital of iodine (WBI = 0.1627). These radical stabilities (RSE) are in sharp contrast to the more unstable **1d** and **1e** derived radicals (3.5 kcal/mol higher BDE). Having established the prominent role of 2,6-lutidine in both the activation of the C–I bond and the stabilization of the ensuing iodine radical, we next set out to unveil the pivotal role of the boron p-orbital. We initially envisaged that preferential alignment of the boron p-orbital and C–I antibonding orbital would result in a weakened bond and larger buildup of positive charge at the σ-hole at iodine, prompting interaction with a Lewis base.^20a^ While this was apparent for substrate **1c** (dihedral angle 98°, BDE 55.3 kcal/mol), the intrinsic electronic properties of **1a** and **1b** are not immediately clear, given the dihedral angles of 48° and 61° respectively. To gain a deeper insight, we analyzed the HOMO and LUMO structures of **1a** (see ESI). The HOMO is located on the lone pairs of iodine showing significant repulsion with both C–B and C–Si σ-bonds. However, analysis of the LUMO indicates that the C–I σ* orbital is delocalized with both the p-orbital of boron and d-orbitals from silicon. It is anticipated that this delocalization again results in a weakened C–I bond and buildup of positive charge at the σ-hole. In summary, computational studies reveal that 2,6-lutidine plays a critical role in both the activation of ambiphilic reagents, through the formation of a photoactive halogen bonding charge transfer complex, and in the stabilization of transient iodine radicals. The boron p-orbital was shown to influence the intrinsic properties of the C–I bond through orbital alignment and delocalization of the LUMO, creating a weakened bond for selective activation.

**Scheme 2:**
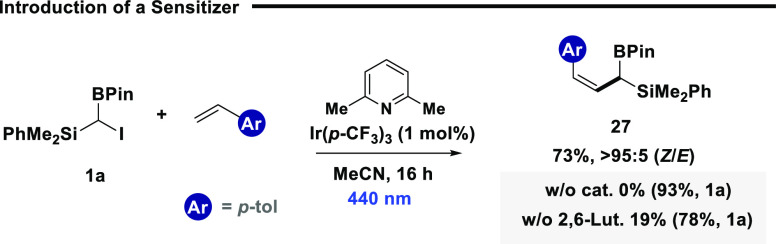
Stereodivergent Synthesis of *Z*-Isomers

Our computational investigation supported the formation of a charge transfer complex in the triplet excited state (*vide supra*). In order to probe this reactivity we carried out reactions in the presence of oxygen a known triplet quencher (Figure 2E).^30^ Under an oxygen atmosphere, reactivity was shown to be completely suppressed, leading to retention of the ambiphilic precursor **1a** in 80%. Inspired by this control reaction, we envisaged that the inclusion of an external photosensitizer may enable the reaction to be carried out efficiently at longer wavelengths (Scheme 2). However, on initially probing this reaction we found that activation also coincided with an energy transfer catalyzed geometric isomerization enabling the stereodivergent access to synthetically versatile *Z*-isomers (Scheme 2).^31,32^ Reaction optimization identified that the iridium catalyst Ir(p-CF_3_)_3_ could be used under 440 nm to enable expedient access to the corresponding *Z*-isomer (see ESI for full details). In the absence of 2,6-lutidine or the photocatalyst at 440 nm, reactivity was suppressed significantly, resulting in the retention of **1a**. These results highlight the importance of photocatalyst and additive for efficient reactivity also at 440 nm. It is pertinent to note that during reaction analysis no Ley-type photoredox activation of boron was observed providing further indication that 2,6-lutidine does not coordinate with the boron p-orbital.^25b^

**Scheme 3:**
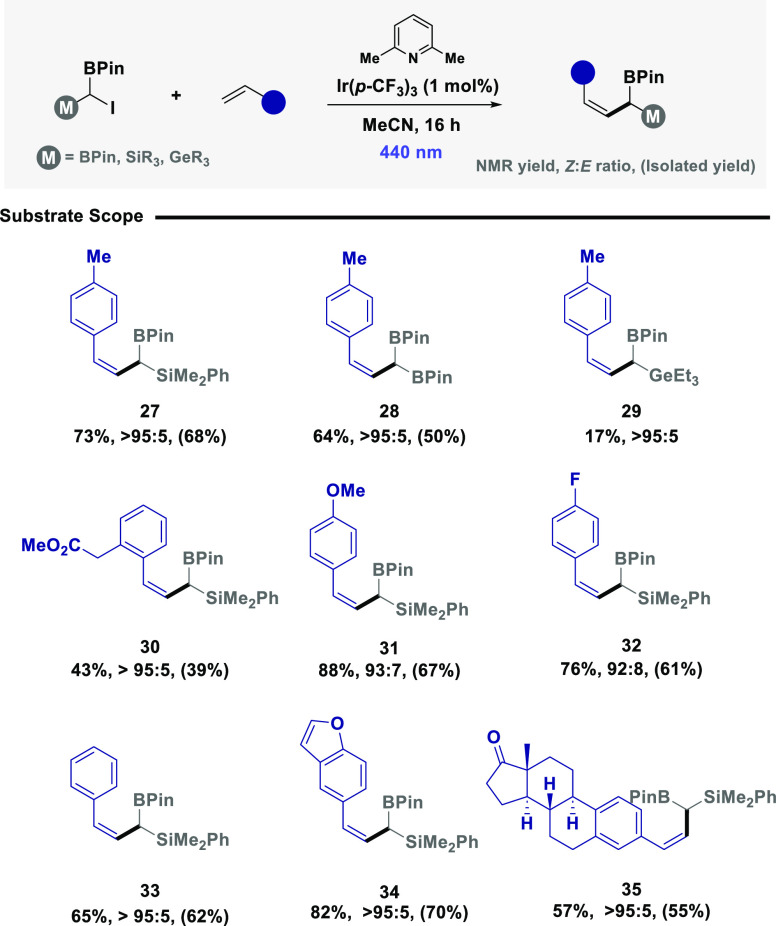
Substrate Scope (a) Reactions were performed in MeCN on a 0.2 mmol scale using **1** (1 equiv), SOMOphile (3 equiv), 2.6-lutidine (1.5 equiv), and Ir(*p*-CF_3_)_3_ (1 mol %) under 440 nm irradiation (40 W). NMR yield and *Z*:*E* ratio were determined by ^1^H NMR spectroscopy against a known internal standard (1,3,5-trimethoxybenzene).

Catalyst screening identified that reactivity was contingent on excited state triplet energies and not on the photoredox properties of the excited state catalyst. However, while this is indicative of a sensitized process, energy transfer to charge transfer complexes is currently unknown, and direct homolytic activation of nonconjugated σ-bonds via energy transfer is, at present, limited to one synthetic example.^33^ The model reaction conditions were compatible with ambiphilic reagent **1a** and a range of styrenes (Scheme 3), including electron-neutral (**27**, **30**, **33**, and **35**), electron-rich (**31**), electron-poor (**32**) and heterocyclic scaffolds (**34**). While ambiphilic reagent **1c** afforded the target *Z*-isomer (**28**) in good yield and selectivity, the use of germanium derivative **1b** was comparatively unsuccessful (**29**), again due to the competitive degradation of the formed product.

**Figure 3:**
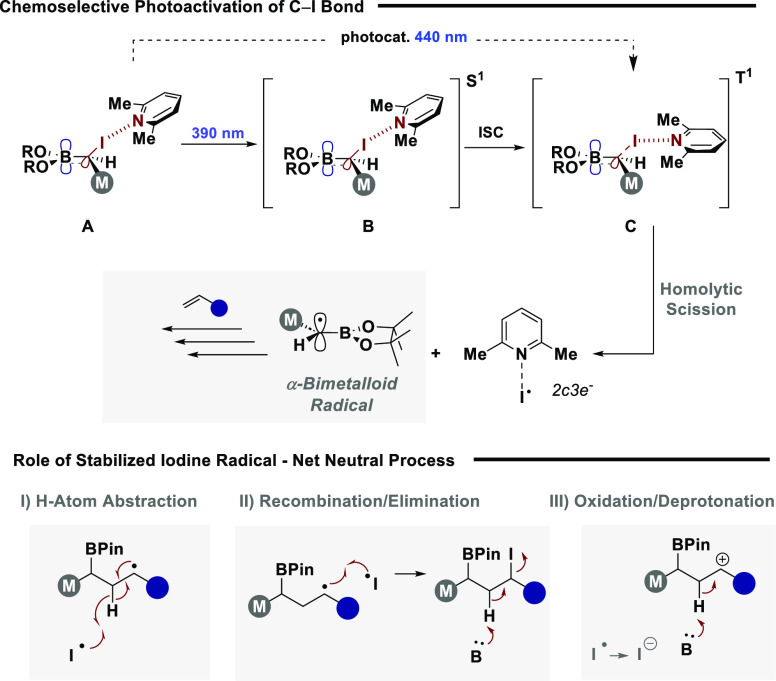
Proposed mechanism of activation.

Guided by our mechanistic analysis thus far, we propose the following mode of activation and mechanistic hypothesis (Figure 3). It is proposed that an n → σ-hole interaction (A) between 2,6-lutidine and the ambiphilic reagent results in bond weakening and a bathochromic shift in absorption properties to aid efficient excitation (Figure 3, top).^34^ Absorption of a photon is first envisaged to excite the adduct to the singlet excited state (B), where the heavy atom effect of iodine is predicted to enable facile inter-system crossing to the calculated triplet excited state (C).^35^ Computational analysis then supports a barrierless homolytic scission of the C–I bond to form an α-bimetalloid radical primed for reactivity with a SOMOphile and a stabilized iodine radical. While the quantum yield could not be determined accurately due to no clear overlap between incident light and absorbing intermediates, light on/off experiments supported the absence of an efficient radical chain (see ESI for full details).^36^ As a result, we propose the stabilized iodine radical serves to close the mechanism, resulting in a net neutral process. Contingent on employed SOMOphile, this could occur via H-atom abstraction (Figure 3, bottom),^37^ radical recombination and subsequent elimination, or oxidation followed by deprotonation.^38^

**Figure 4:**
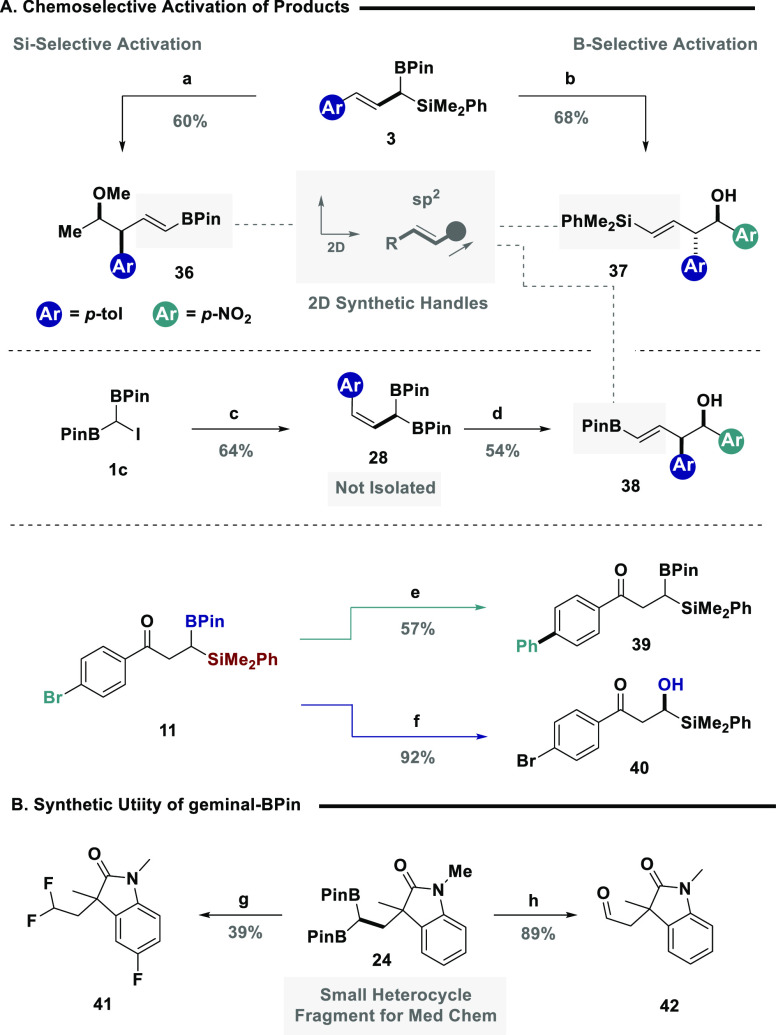
(A) Chemoselective activation of products: (a) **3**, 1,1-dimethoxyethane (1.03 equiv), TiCl_4_ (1.2 equiv), DCM, −78 °C; (b) **3**, 4-nitrobenzaldehyde (1 equiv), MeCN, 50 °C; (c) **1c** (1 equiv), 4-methylstyrene (3 equiv), 2–6-lutidine (1.5 equiv), MeCN, 390 nm irradiation; (d) **28**, 4-nitrobenzaldehyde (1 equiv), MeCN, 50 °C; (e) **11** (1 equiv), phenylboronic acid (1.5 equiv), Pd(OAc)_2_ (5 mol %), SPhos (10 mol %), K_3_PO_4_ (3 equiv), 1,4-dioxane, H_2_O, 80 °C; (f) **11**, NaBO_3_·H_2_O (3 equiv), THF, H_2_O, 0 °C—rt; (B) synthetic utility of *geminal*-BPin: (g) **24** (1 equiv), AgNO_3_ (0.2 equiv), selectfluor (3 equiv), TFA, H_3_PO_4_, H_2_O, DCM, 50 °C; (h) **24** (1 equiv), NaBO_3_·H_2_O (3 equiv), THF, H_2_O, 0 °C—rt.

The overarching vision of the study was to facilitate the light-driven, chemoselective activation of ambiphilic reagents to grant expedient access to organic scaffolds containing multiple synthetic handles that can permit the subsequent exploration of chemical space. To demonstrate the power of the methodology we next focused on product derivatization, paying particular attention to complimenting existing chemoselective activation modes (Figure 4).^14a,39^ Allylic system **3** containing both boron and silicon handles with orthogonal reactivity, was activated via a Hosomi–Sakurai reaction to grant access to vinyl BPin **36**, as a single diastereomer, in good yield. Conventional allylation via activation of the boron motif enabled the synthesis of **37**, containing a silicon handle for further derivitization. Complementary 3D vectors were accessed through the *Z*-allylic systems, with the allylation of **28** giving the *syn* vinyl BPin **38** in great diastereoselectivity. Aryl ketone **11** provided three orthogonal handles available for chemoselective transformations. This allowed selective activation of the aryl bromide through an sp^2^-selective Suzuki–Miyaura cross-coupling reaction to furnish biphenyl species **39**, while selective oxidation of the boron motif allowed for the formation of α-silyl alcohol **40**, which intriguingly avoided a Brooke rearrangement. In both of these examples, all other functional handles remained available for further downstream manipulations. Finally, *geminal*-BPin systems have been shown to be versatile precursors in synthesis, and to demonstrate their utility, oxindole fragment **24** was converted into the corresponding geminal difluoride (**41**),^40^ a key motif in medicinal chemistry. Similarly, the translation of both boron motifs to a carbonyl was achieved in the synthesis of aldehyde **42**.^41^

## Conclusions

In summary, we have developed an operationally simple chemoselective strategy to activate ambiphilic reagents to generate α-bimetalloid radicals using light and a simple Lewis base additive. These transient radicals can engage various SOMOphiles to grant expedient access to scaffolds containing multiple synthetic handles as 3D exit vectors. In-depth mechanistic and computational investigations revealed the prominent role of the boron p-orbital and 2,6-lutidine in the activation of the C–I bond via the formation of a halogen bonding charge transfer complex, while computational investigations also highlighted that photoactivated homolytic bond cleavage occurs in the triplet excited state, leading to the inception of a photocatalyzed stereodivergent approach to *Z*-isomers. The power of the method was demonstrated in product derivatization via chemoselective activation of pendant synthesis handles permitting the accurate exploration of chemical space. It is envisaged this mild platform to access high-energy α-bimetalloid radicals can be strategically paired with asymmetric cross-coupling protocols that will prove expansive as a future entry point into chiral hydrocarbons.^42^

## Supporting Information Available

Experimental details, analytical data for compound characterization, reaction optimization, mechanistic studies, and computational studies (PDF)

## Abbreviations

BDE: bond dissociation energy
DCM: dichloromethane
DMF: dimethylfromamide
ESI: electronic supporting information
HOMO: highest occupied molecular orbital
LUMO: lowest unoccupied molecular orbital
NMR: nuclear magnetic resonance
PES: potential energy surface
RSE: radical stabilization energy
SOMO: singly occupied molecular orbital
TFA: trifluoroacetic acid
UV: ultraviolet
