# Supplementary material for: Photogeneration of α-Bimetalloid Radicals via Selective Activation of Multifunctional C1 Units
Source: J Am Chem Soc. 2024 May 28;146(23):15850–9. doi: 10.1021/jacs.4c02261 (PMC11177267; doi:10.1021/jacs.4c02261)

Supplementary Information for

Photogeneration of α-Bimetalloid Radicals via Selective Activation of Multifunctional C_1 Units

Lewis McGhie, Alessandro Marotta, Patrick O. Loftus, Peter H. Seeberger, Ignacio Funes-Ardoiz, and John J. Molloy

Table of Contents

General Information ... 3

Synthesis of Starting Materials ... 4

- **α-Iodo Precursors** ... 4
- **SOMOphiles & Lewis Base Derivatives** ... 6

Experimental Setup for Photoreactions ... 13

Reaction Optimization: *E*-Allylic Boronic Esters ... 15

Substrate Scope ... 17

Mechanistic Studies ... 30

- **Control Reactions** ... 30
- **UV/Vis Analysis** ... 32
- **Cyclic Voltammetry Studies** ... 39
- **Time Interval Studies** ... 45
- **NMR Analysis** ... 49

Reaction Optimization: *Z*-Allylic Boronic Esters ... 51

Substrate Scope: *Z*-Allylic Boronic Esters ... 52

Derivatization of Products ... 56

Computational Investigation ... 60

References ... 81

NMR Spectra ... 84

General Information

All chemicals were purchased as reagent grade and used without further purification unless stated otherwise. Commercial styrenes were passed through a pipette containing silica (1 cm) prior to use. Dry solvents were obtained by passing solvents through activated alumina columns and storing them over activated 4 Å molecular sieves for 24 h prior to use. Degassed solvent refers to bubbling argon through the solvent for a minimum of 15 min. Solvents for purification (extraction and chromatography) were purchased as technical grade and distilled on the rotary evaporator prior to use. For column chromatography SiO_2 (40-63 μm for flash chromatography, Macherey Nagel or VWR) and C18-(50 µm Büchi FlashPure EcoFlex 4 g) were used as a stationary phase. Analytical thin layer chromatography (TLC) was performed on pre-coated TLC sheets ALUGRAM® XtraSIL G/UV_{254} (Macherey Nagel). UV light (254 nm), potassium permanganate ($KMnO_4$), vanillin and *p*-anisaldehyde stain solutions were used for visualization. Concentration under reduced pressure was performed at ~10 mbar and 40 °C, drying at ~10–2 mbar and ambient temperature. NMR spectra were measured on either a Varian 400 MHz, Bruker Ascend 400 MHz, Varian 600 MHz or Bruker Ascend 700 MHz at ambient temperature. The chemical shifts are referenced to the residual solvent peak as internal standard and are reported in ppm. The resonance multiplicity is abbreviated as: s (singlet), d (doublet), t (triplet), q (quartet), quint (quintet), m (multiplet) and br (broad). Assignments of unknown compounds are based on APT, DEPT, COSY(HH), HMBC, HSQC and NOESY spectra. Carbon atoms bearing boron were not observed by ^{13}C NMR and are not reported. High-resolution mass spectra were measured by the MS service of Freie Universität Berlin. UV/Vis spectra were recorded using a Shimadzu UV-1900 I spectrophotometer. The samples were prepared and recorded in UV-grade cuvettes (PlastiBrand) or quartz cuvettes with a pathlength of 1 cm. IR spectra were recorded on a Perkin-Elmer Spectrum 100 FT-IR spectrometer, selected adsorption bands are reported in wavenumbers (cm^{-1}). Photoreactions were performed using Kessil PR-160L lamps (370, 390, 427 and 440 nm). Photoreaction setup, including light source emission is comprehensively described (*vide infra*).

Synthesis of Starting Materials

α-Iodo Precursors

Dimethyl(phenyl)(4,4,5,5-tetramethyl-1,3,2-dioxaborolan-2-yl)silane (S1)

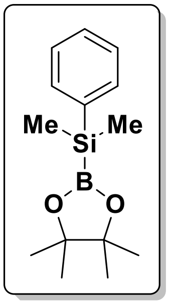

S1 was prepared according to a modified procedure of Oestreich *et al*.[1] To an oven-dried round-bottom flask lithium chunks (999 mg, 144 mmol, 4.0 equiv.) were suspended in anhydrous THF (30 mL) under an atmosphere of argon. Chloro(dimethyl)phenylsilane (6.0 mL, 36 mmol, 1.0 equiv.) was added dropwise at 0 ºC and the reaction mixture stirred overnight. The reaction mixture was added dropwise to a solution of 4,4,5,5-tetramethyl-1,3,2-dioxaborolane (10.4 mL, 72 mmol, 2.0 equiv.) in *n*-hexane (40 mL) at 0 ºC under an atmosphere of argon. The reaction mixture was stirred at ambient temperature overnight. The mixture was diluted with *n*-hexane and filtered through a pad of Celite to remove residual solid. The crude material was concentrated *in vacuo* before being taken up in *n*-hexane and filtered through another pad of Celite. The solvent was removed *in vacuo* and the crude material was used in the next step without further purification.

((Dimethyl(phenyl)silyl)iodomethyl)boronic acid, pinacol ester (1a)

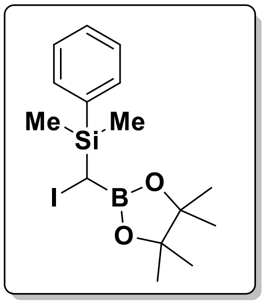

1a was prepared according to a modified procedure of Gevorgyan *et al*.[2] To a solution of **S1** (1.0 mL, 3.8 mmol, 1.0 equiv) in THF (10 mL) at -100 ºC was added LiHMDS (1 M in THF, 4.2 mL, 1.1 equiv) under argon atmosphere. After 1 h diiodomethane (0.34 mL, 4.2 mmol, 1.1 equiv) in 10 mL *n*-hexane/THF (1:1) was added to the reaction mixture over 20 min at -100 ºC. The reaction was stirred for 1 h at -100 ºC and allowed to gradually warm to ambient temperature and stirred overnight. Upon completion, the reaction was quenched with sat. aq. NH_4Cl solution (5 mL). The resulting mixture was diluted with diethyl ether (20 mL), and washed with water (10 mL). The organic layer was dried over Na_2SO_4, filtered and concentrated in vacuo. The crude residue was purified by flash column chromatography (SiO_2, 0→5% EtOAc/*n*-hexane) to yield **1a** as a pale-yellow oil (885 mg, 59% yield).

^{1}H NMR (400 MHz, $CDCl_3$): δ = 7.60 – 7.53 (m, 2H), 7.41 – 7.29 (m, 3H), 2.05 (s, 1H), 1.17 (s, 6H), 1.13 (s, 6H), 0.51 (s, 3H), 0.49 (s, 3H) ppm; analytical data in agreement with literature.[2]

(Chloromethyl)triethylgermane (S2)

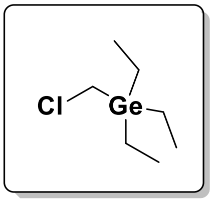

S2 was prepared according to a procedure of Schoenebeck *et al.*[3] To an oven-dried round-bottom flask was added chlorotriethylgermane (1.7 mL, 10 mmol, 1 equiv.) to anhydrous THF (30 mL) and cooled to -78 ºC. Chloroiodomethane (2.2 mL, 30 mmol, 3 equiv.) was added and the reaction mixture was stirred for 10 minutes. MeLi.LiBr (1.5 M in Et_2O, 22 mL, 28 mmol, 2.8 equiv.) was added dropwise to the reaction mixture at -78 ºC and stirred for 1 h. The reaction mixture was quenched by the addition of sat. aq. NH_4Cl and extracted with Et_2O, combined organic layers dried over Na_2SO_4, filtered and concentrated *in vacuo*. The residue was purified by silica plug (pentane) to yield **S2** as a colourless oil. (1.67 g, 80% yield).

^{1}H NMR (400 MHz, $CDCl_3$): δ = 3.01 (s, 2H), 1.06 (t, J = 8.0 Hz, 9H), 0.87 (q, J = 7.8 Hz, 6H) ppm; analytical data in agreement with literature.[3]

(Iodo(triethylgermyl)methyl)boronic acid, pinacol ester (1b)

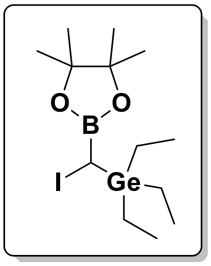

1b was prepared according to a procedure of Schoenebeck *et al*.[3] To an oven-dried round-bottom flask was added **S2** (209 mg, 1 mmol, 1 equiv.) and anhydrous THF (5 mL) and cooled to -78 ºC. *s*-BuLi (1.4 M, 0.86 mL, 1.2 mmol, 1.2 equiv.) was added dropwise and the reaction mixture was stirred for 1 h at -78 ºC. After 1 h, 2-isopropoxy-4,4,5,5-tetramethyl-1,3,2-dioxaborolane (0.24 mL, 1.2 mmol, 1.2 equiv.) was added dropwise and the reaction mixture stirred for a further 4 h at -78 ºC. After completion, the reaction was quenched with addition of sat. aq. NH_4Cl at - 78 ºC and gradually warmed to ambient temperature. The solution was extracted with Et_2O, combined organic layers dried over Na_2SO_4, filtered and concentrated *in vacuo*. The crude residue was obtained as a colourless oil (207 mg, 0.62 mmol, 62%) and used in the next step with no further purification. To an oven-dried glass vial was added the crude intermediate, NaI (279 mg, 1.9 mmol, 3 equiv.) and anhydrous MeCN (1.5 mL). The vial was sealed and the reaction mixture stirred at 80 ºC overnight. The reaction mixture was cooled to ambient temperature and quenched by the addition of sat. aq. $Na_2S_2O_3$. The mixture was extracted with Et_2O, combined organic layers were dried over Na_2SO_4, filtered and concentrated *in vacuo*. The crude residue was purified by flash column chromatography (SiO_2, 0→5% Et_2O/*n*-pentane) to yield **1b** as a colourless oil (205 mg, 48% yield over two steps).

^{1}H NMR (400 MHz, $CDCl_3$): δ = 2.04 (s, 1H), 1.25 (s, 12H), 1.09 – 1.04 (m, 9H), 0.97 – 0.92 (m, 6H) ppm; analytical data in agreement with literature.[3]

Methylenediboronic acid, pinacol ester (S3)

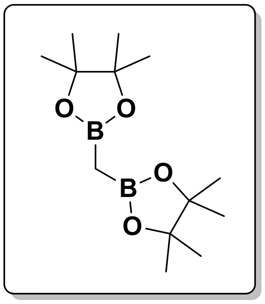

S3 was prepared according to a procedure of Fu *et al.*[4] To an oven-dried round-bottom flask was added CuI (381 mg, 2 mmol, 0.1 equiv.), lithium methoxide (1.90 g, 50 mmol, 2.5 equiv.), and bis(pinacolato)diboron (11.2 g, 44 mmol, 2.2 equiv.). The flask was sealed and purged with argon before the addition of DMF (60 mL, 0.3 M) and dibromomethane (1.4 mL, 20 mmol, 1 equiv.). The reaction mixture was heated to 40 ºC and stirred for 24 h. After the reaction was complete, the reaction was cooled to ambient temperature before being diluted with EtOAc and filtered through silica gel with washes of EtOAc. Solvent was removed *in vacuo* and the crude material purified by flash column chromatography (SiO_2, 0→5% EtOAc/*n*-hexane) to yield **S3** as a white solid (3.70 g, 69%).

^{1}H NMR (400 MHz, $CDCl_3$): δ = 1.23 (s, 24H), 0.35 (s, 2H) ppm; analytical data in agreement with literature.[4]

(Iodomethylene)diboronic acid, pinacol ester (1c)

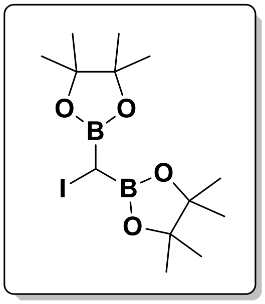

1c was prepared according to a procedure of Marder *et al.*[5] To a solution of **S3** (2.68 g, 10 mmol, 1.0 equiv) in THF (10 mL) at 0 ºC was slowly added lithium diisopropylamide (5.5 ml, 11 mmol, 1.1 equiv.) in THF/heptane/ethylbenzene (2M) under argon atmosphere. After 1 h, zinc chloride (1.50 g, 11 mmol, 1.1 equiv.) was added and mixture stirred at 0 ºC for 10 min, and then warmed to ambient temperature over 2 h. The above mixture was slowly added dropwise to a solution of iodine (2.54 g, 10 mmol, 1 equiv.) in DCM (20 mL) at 0 ºC under an argon atmosphere. The reaction mixture was warmed to ambient temperature and stirred for 2 h. The suspension was washed with Na_2SO_3 solution and extracted with DCM, and the organic phase was washed with brine, dried over Na_2SO_4, filtered and concentrated in

vacuo. The crude residue was dissolved in *n*-hexane (150 mL, pre-chilled at -78 ºC), filtered through a pad of celite, washed with *n*-hexane (150 mL, pre-chilled at -78 ºC), and concentrated under reduced pressure. The crude residue was re-dissolved in *n*-hexane (150 mL, pre-chilled at -78 ºC), filtered through a pad of celite/MgSO4, washed with *n*-hexane (150 mL, pre-chilled at -78 ºC), and concentrated under reduced pressure. The crude mixture was purified by recrystallization under n-hexane at -78 ºC to give **1c** as an off-white solid (1.58 g, 40% yield).

^{1}H NMR (400 MHz, $CDCl_3$): δ = 2.07 (s, 1H), 1.26 (s, 12H), 1.25 (s, 12H) ppm; analytical data in agreement with literature.[5]

(Iodomethyl)dimethyl(phenyl)silane (1d)

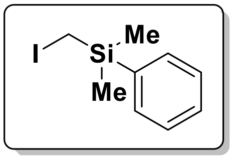

1d was prepared according to a procedure of Tilley *et al.*[6] To an oven-dried round-bottom flask was added (chloromethyl)dimethyl(phenyl)silane (1.8 mL, 10 mmol, 1 equiv.) and NaI (2.62 g, 17.5 mmol, 1.75 equiv.) before being sealed and purged with argon. Anhydrous acetone (10 mL) was added and the reaction stirred at reflux for 24 h. The reaction mixture was cooled to ambient temperature and solvent removed *in vacuo* to give a slurry. The slurry was taken up in *n*-hexane and passed through a pad of Celite®, with washes of *n*-hexane. The solvent was removed *in vacuo* to yield **1d** as a colourless oil (2.48 g, 90%).

^{1}H NMR (400 MHz, $CDCl_3$): δ = 7.56 – 7.52 (m, 2H), 7.44 – 7.35 (m, 3H), 2.19 (s, 2H), 0.45 (s, 6H) ppm; analytical data in agreement with literature.[6]

Triethyl(iodomethyl)germane (1e)

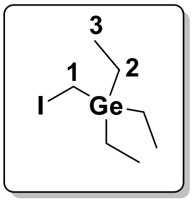

1e was prepared according to a modified procedure of Tilley *et al.*[6] To an oven-dried round-bottom flask was added **S2** (209 mg, 1 mmol, 1 equiv.), NaI (300 mg, 2 mmol, 2 equiv.) and anhydrous acetone (3 mL). The reaction mixture was stirred at reflux overnight. The reaction mixture was cooled to ambient temperature and the solvent removed *in vacuo* to give a slurry. The slurry was taken up in *n*-pentane and passed through a pad of Celite®, with washes of *n*-pentane. The solvent was removed in vacuo to yield **1e** as a colourless oil (241 mg, 80%).

^{1}H NMR (400 MHz, $CDCl_3$): δ = 2.11 (s, 2H, H1), 1.07 – 1.02 (m, 9H, H3), 0.90 – 0.83 (m, 6H, H2) ppm; **^{13}C NMR** (101 MHz, $CDCl_3$) δ = 8.8 (C3), 4.8 (C2), -18.8 (C1) ppm; **IR** (ATR): ṽ = 2951.05, 2907, 2873, 1462, 1426, 1379, 1234, 1073, 1015, 970, 812, 751, 701 cm^{-1}; **HRMS** (EI) calcd. For $C_7H_{17}GeI$ $[M-C_2H_5]^+$ 272.9195, found 272.9202.

SOMOphiles & Lewis Base Derivatives

General Procedure A: Suzuki-Miyaura cross-coupling

The reaction was performed according to a modified procedure of Watson *et al.*[7] A two necked flask equipped with a reflux condenser and a stirring bar was charged with arylbromide (1 equiv.), potassium vinyltrifluoroborate (1.2 equiv.), $Pd(OAc)_2$ (4 mol%), SPhos (8 mol%), K_3PO_4 (3 equiv.). The flask was sealed and purged with nitrogen before the addition of degassed 1,4 dioxane (0.4 M) and H_2O (5.5 equiv.). The reaction mixture was heated to 80 °C for 16 h. After cooling the crude reaction mixture was diluted with EtOAc and passed through a celite plug. After concentration under reduced pressure, the residue was diluted with EtOAc and washed with H_2O. Organics were extracted with EtOAc (3 x). The combined

organic phases were dried over Na_2SO_4, filtered, and concentrated under reduced pressure. The crude residue was purified by flash column chromatography (SiO_2, specified combination of solvents).

General Procedure B: Silyl-enol ether formation

The reaction was performed according to a modified procedure of Gademann *et al.*[8] Ketone (1 equiv.) was added as a solution in anhydrous DCM (0.5 M) to an oven-dried round bottom flask under a nitrogen atmosphere. Triethylamine (1.2 equiv.) was added dropwise. The reaction mixture was stirred for 1 h at ambient temperature before the dropwise addition of TBSOTf (1.2 equiv.). The solution was stirred overnight at ambient temperature. Upon completion, the reaction was quenched using cold sat. aq. NH_4Cl. Organics were extracted with diethyl ether (3 x). The combined organic extracts were dried over $MgSO_4$, filtered, and concentrated under reduced pressure. The crude residue was purified by flash column chromatography on deactivated SiO_2 (5 % Et_3N in *n*-hexane) using *n*-hexane as the eluent.

2,6-Dimethyl-4-dimethylaminopyridine (S4)

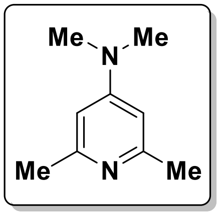

S4 was prepared according to a procedure of Mitzel *et al*.[9] To an oven-dried vial was added 4-chloro-2,6-dimethylpyridine (0.25 mL, 2 mmol, 1 equiv.) and aqueous dimethylamine solution (2.5 mL, 20 mmol, 40 wt%, 10 equiv.). Vial was sealed and reaction mixture stirred at 160 ºC for 3 days. Reaction mixture was cooled to ambient temperature and water added. Aqueous phase was basified with addition of 10% NaOH solution and organics extracted with DCM (3 x 5 mL). Combined organics were washed with brine, dried over Na_2SO_4, filtered and concentrated in vacuo to yield **S4** as an off-white solid (168 mg, 55%).

^{1}H NMR (400 MHz, $CDCl_3$): δ = 6.24 (s, 2H), 3.01 (s, 6H), 2.49 (s, 6H) ppm; analytical data in agreement with literature.[9]

5-Vinylbenzofuran (S5)

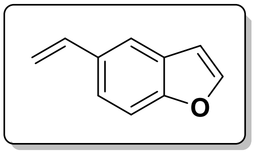

Prepared according to General Procedure **A**, 5-bromobenzofuran (0.63 mL, 5 mmol) was converted to **S5** yielding a colourless oil (605 mg, 84%) after purification by flash column chromatography (SiO_2, 0→6% Et_2O/*n*-hexane).

^{1}H NMR (400 MHz, $CDCl_3$) δ = 7.62 – 7.61 (m, 2H), 7.45 (d, *J* = 8.5 Hz, 1H), 7.40 (dd, *J* = 8.5, 1.6 Hz, 1H), 6.82 (dd, *J* = 17.6, 10.9 Hz, 1H), 6.75 (dd, *J* = 2.2, 0.9 Hz, 1H), 5.73 (dd, *J* = 17.6, 0.9 Hz, 1H), 5.22 (dd, *J* = 10.9, 0.9 Hz, 1H) ppm; analytical data in agreement with literature.[10]

Methyl 2-(2-bromophenyl)acetate (S6)

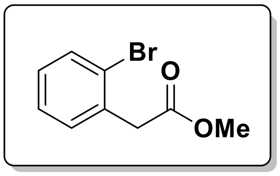

2-Bromophenylacetic acid (1.08 g, 5 mmol, 1 equiv.) was dissolved in MeOH (7.0 mL, 0.7 M) before the addition of H_2SO_4 (60 µL). The reaction mixture was stirred at reflux for 24h. After cooling, the solvent was removed under reduced pressure. The residue was dissolved in Et_2O (20 mL) and washed with sat. aq. $NaHCO_3$ (20 mL) and brine (10 mL). The organic phase was dried over Na_2SO_4 and concentrated under reduced pressure yielding **S6** as a colourless oil (1.09 g, quant.).

^{1}H NMR (400 MHz, $CDCl_3$) δ = 7.57 (d, *J* = 8.0 Hz, 1H), 7.29 – 7.28 (m, 2H), 7.17 – 7.13 (m, 1H), 3.81 (s, 2H), 3.72 (s, 3H) ppm; analytical data in agreement with literature.[11]

Methyl 2-(2-vinylphenyl)acetate (S7)

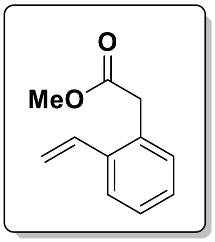

Prepared according to General Procedure **A**, **S6** (1.09 g, 4.7 mmol) was converted to **S7** yielding a yellow oil (721 mg, 87%), after purification by flash column chromatography (SiO_2, 0→10% EtOAc/*n*-hexane).

^{1}H NMR (400 MHz, $CDCl_3$) δ = 7.52 (dd, *J* = 7.2, 1.7 Hz, 1H), 7.30 – 7.20 (m, 3H), 6.95 (dd, *J* = 17.3, 11 Hz, 1H), 5.66 (dd, *J* = 17.3, 1.3 Hz, 1H), 5.34 (dd, *J* = 11, 1.3 Hz, 1H), 3.71 (s, 2H), 3.68 (s, 3H) ppm; analytical data in agreement with literature.[12]

(8*R*,9*S*,13*S*,14*S*)-13-Methyl-17-oxo-7,8,9,11,12,13,14,15,16,17-decahydro-6*H*cyclopenta[a]phenanthren-3-yl trifluoromethanesulfonate (S8)

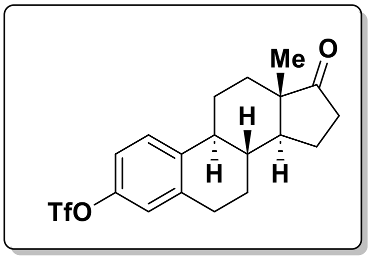

To an oven-dried round bottom flask, Estrone (500 mg, 1.8 mmol, 1 equiv.) was added. The flask was sealed and purged with nitrogen before the addition of anhydrous DCM (3.6 mL) and pyridine (0.44 mL, 5.5 mmol, 3 equiv.) via syringe. The reaction mixture was cooled to 0 °C and triflic anhydride (0.37 mL, 2.2 mmol, 1.2 equiv.) was added dropwise. The reaction mixture was stirred at ambient temperature for 12 h. After completion, the reaction mixture was diluted with H_2O (5 mL) and extracted with DCM (3 x 10 mL). The combined organic phases were dried over MgSO4 and filtered. The resulting solution was concentrated under reduced pressure and purified via column chromatography (SiO_2, 15% EtOAc/*n*-hexane), yielding **S8** as a white solid (650 mg, 90 %).

^{1}H NMR (400 MHz, $CDCl_3$) δ = 7.34 (d, *J* = 8.7 Hz, 1H), 7.04 (dd, *J* = 8.7, 2.6 Hz, 1H), 6.99 (d, *J* = 2.6 Hz, 1H), 2.94 (dd, *J* = 8.9, 4.3 Hz, 2H), 2.52 (dd, *J* = 19.1, 8.9 Hz, 1H), 2.43 – 2.38 (m, 1H), 2.30 (td, *J* = 10.9, 4.3 Hz, 1H), 2.21 – 1.96 (m, 4H), 1.67 – 1.44 (m, 6H), 0.92 (s, 3H) ppm; analytical data in agreement with literature.[13]

(8*R*,9*S*,13*S*,14*S*)-13-Methyl-3-vinyl-6,7,8,9,11,12,13,14,15,16-decahydro-17*H*-cyclopenta[a]phenanthren-17-one (S9)

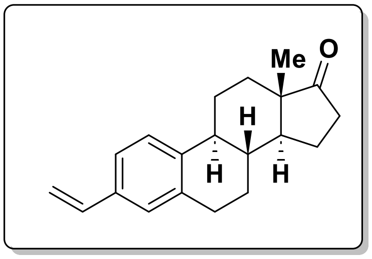

Prepared according to General Procedure **A**, **S8** (650 mg, 1.62 mmol, 1 equiv.) was converted to **S9**, yielding a white solid (380 mg, 84 %) after purification by flash column chromatography (SiO_2, 0→9% EtOAc/*n*-hexane).

^{1}H NMR (400 MHz, $CDCl_3$) δ = 7.27 – 7.25 (m, 1H), 7.23 – 7.20 (m, 1H), 7.15 (s, 1H), 6.67 (dd, *J* = 17.5, 10.9 Hz, 1H), 5.70 (d, *J* = 17.5 Hz, 1H), 5.19 (d, *J* = 10.9 Hz, 1H), 2.92 (dd, *J* = 9.1, 4.2 Hz, 2H), 2.54 – 2.41 (m, 2H), 2.31 (t, *J* = 10.8 Hz, 1H), 2.20 – 1.95 (m, 4H), 1.69 – 1.40 (m, 6H), 0.91 (s, 3H) ppm; analytical data in agreement with literature.[14]

4-(1-((*tert*-butyldimethylsilyl)oxy)vinyl)benzonitrile (S10)

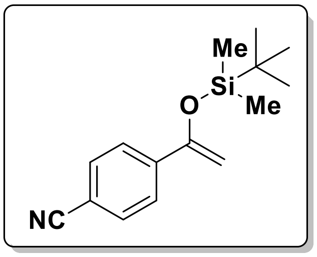

Prepared according to General Procedure **B,** 4-cyano acetophenone (726 mg, 5 mmol) was converted to **S10** yielding a colourless oil (1.24 g, 95%).

^{1}H NMR (400 MHz, DMSO-d_6): δ = 7.87 – 7.83 (m, 2H), 7.81 – 7.77 (m, 2H), 5.26 (d, *J* = 2.4 Hz, 1H), 4.62 (d, *J* = 2.4 Hz, 1H), 0.97 (s, 9H), 0.21 (s, 6H) ppm; analytical data in agreement with literature.[15]

***tert*-butyldimethyl((1-(naphthalen-2-yl)vinyl)oxy)silane (S11)**

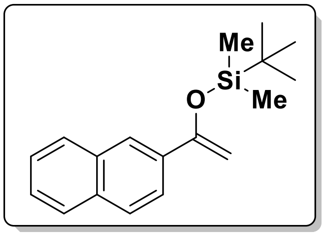

Prepared according to General Procedure **B,** 2-Acetylnaphthalene (851 mg, 5 mmol) was converted to **S11** yielding an off-white solid (1.27 g, 88%).

^{1}H NMR (400 MHz, DMSO-d_6): δ = 8.09 (d, *J* = 1.8 Hz, 1H), 7.93 – 7.87 (m, 3H), 7.76 (dd, *J* = 8.7, 1.8 Hz, 1H), 7.55 – 7.49 (m, 2H), 5.20 (d, *J* = 2.0 Hz, 1H), 4.56 (d, *J* = 2.0 Hz, 1H), 1.01 (s, 9H), 0.23 (s, 6H) ppm; analytical data in agreement with literature.[16]

((1-(4-Bromophenyl)vinyl)oxy)(*tert*-butyl)dimethylsilane (S12)

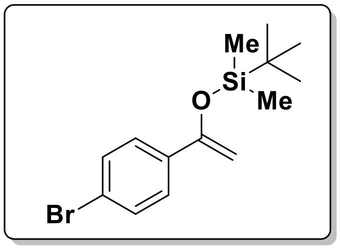

Prepared according to General Procedure **B**, 4-bromo acetophenone (1.99 g, 10 mmol) was converted to **S12** yielding a colourless oil (2.41 g, 77%).

^{1}H NMR (400 MHz, DMSO-d_6) δ = 7.58 – 7.53 (m, 4H), 5.08 (d, *J* = 2.1 Hz, 1H), 4.46 (d, *J* = 2.1 Hz, 1H), 0.96 (s, 9H), 0.20 (s, 6H) ppm; analytical data in agreement with literature.[8]

***tert*-Butyldimethyl((1-(p-tolyl)vinyl)oxy)silane (S13)**

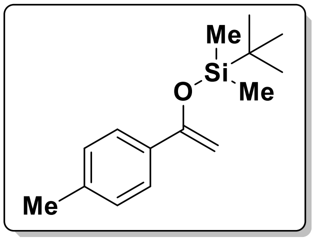

Prepared according to General Procedure **B**, 4-methyl acetophenone (1.34 mL, 10 mmol) was converted to **S13** yielding a colourless oil (2.50 g, quant.).

^{1}H NMR (400 MHz, DMSO-d_6) δ = 7.48 (d, *J* = 8.2 Hz, 2H), 7.13 (d, *J* = 8.2 Hz, 2H), 4.95 (d, *J* = 1.7 Hz, 1H), 4.36 (d, *J* = 1.7 Hz, 1H), 2.35 (s, 3H), 0.96 (s, 9H), 0.18 (s, 6H) ppm; analytical data in agreement with literature.[17]

1-(4-(Bromomethyl)phenyl)ethan-1-one (S14)

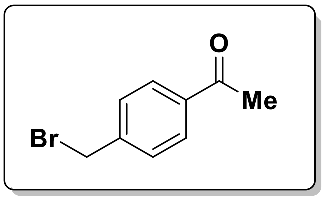

In an oven dried flask equipped with a reflux condenser a solution of 4-methylacetophenone (2.7 mL, 20 mmol) in degassed MeCN (22 mL) was prepared. NBS (3.92 g, 22 mmol) and AIBN (328 mg, 2 mmol) were added under a flow of Argon. The mixture was heated to reflux and stirred for 4 h. The reaction mixture was gradually cooled to ambient temperature before being concentrated under reduced pressure. The residue was purified by flash column chromatography (SiO_2, 0→10% EtOAc/*n*-hexane) affording **S14** as a white solid (2.61 g, 61%).

^{1}**H NMR** (400 MHz, $CDCl_3$): δ = 7.94 (d, J = 8.3 Hz, 2H), 7.49 (d, J = 8.3 Hz, 2H), 4.50 (s, 2H), 2.60 (s, 3H) ppm; analytical data in agreement with literature.[18]

1-(4-(Morpholinomethyl)phenyl)ethan-1-one (S15)

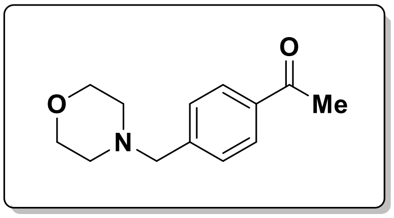

In a flame dried Schlenk flask, a solution of **S14** (2.00 g, 9.4 mmol) in dry MeCN (45 mL) was prepared. K_2CO_3 (1.30 g, 9.4 mmol) was added under a flow of argon. The flask was sealed and placed in an ice bath. Morpholine (0.81 ml, 9.4 mmol) was added dropwise. The reaction mixture was stirred at ambient temperature overnight. The solvent was evaporated under reduced pressure and the residue was dissolved in EtOAc. The organic layer was washed with H_2O and brine, dried over Na_2SO_4, filtered and the solvent removed under reduced pressure. The residue was purified by flash column chromatography (deactivated SiO_2 (5% Et_3N in *n*-hexane), 0→25% EtOAc/*n*-hexane) affording **S15** as a yellow oil (1.33 g, 65%).

^{1}**H NMR** (400 MHz, $CDCl_3$): δ = 7.92 (d, J = 8.3 Hz, 2H), 7.44 (d, J = 8.2 Hz, 2H), 3.72 (t, J = 4.7 Hz, 4H), 3.55 (s, 2H), 2.60 (d, J = 1.1 Hz, 3H), 2.50 – 2.39 (m, 4H) ppm; analytical data in agreement with literature.[19]

4-(4-(1-((*tert*-butyldimethylsilyl)oxy)vinyl)benzyl)morpholine (S16)

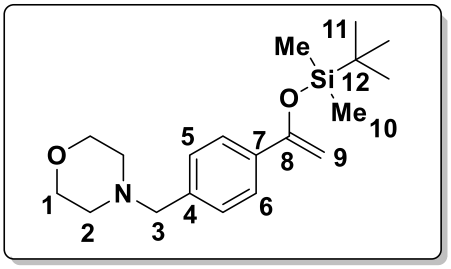

Prepared according to General Procedure **B**, **S15** (1.01 g, 5 mmol) was converted to **S16** yielding a pale yellow solid (1.55 g, 93%).

R*f* (100% EtOAc) = 0.79; ^{1}**H NMR** (400 MHz, DMSO-d_6) δ 7.55 (d, J = 8.4 Hz, 2H, H6), 7.29 (d, J = 8.4 Hz, 2H, H5), 4.98 (d, J = 2.0 Hz, 1H, H9), 4.40 (d, J = 2.0 Hz, 1H, H9), 3.56 (t, J = 4.7 Hz, 4H, H1 and H2), 3.45 (s, 2H, H3), 2.33 (t, J = 4.7 Hz, 4H), 0.96 (s, 9H, H11), 0.19 (s, 6H, H10) ppm; ^{13}**C NMR** (101 MHz, DMSO-d_6) δ 154.9 (C8), 138.2 (C4), 135.8 (C7), 128.8 (C5), 124.7 (C6), 91.0 (C9), 66.2, 62.1 (C3), 53.2, 25.7 (C11), 18.0 (C12), -4.7 (C10) ppm; **IR** (ATR) $\tilde{\nu}$ = 3660, 2959, 2932, 2893, 2858, 2807, 2166, 1616, 1568, 1510, 1473, 1464, 1456, 1412, 1393, 1363, 1350, 1312, 1292, 1255, 1207, 1180, 1162, 1117, 1106, 1072, 1004, 940, 915, 868, 834, 813, 780, 734, 711, 688, 662 cm^{-1}; **HRMS** (ESI) calcd. for $C_{19}H_{31}NO_2Si^+$ $[M+H]^+$ 334.2197, found 334.2151.

***tert*-butyl((1-(2-methoxyphenyl)vinyl)oxy)dimethylsilane (S17)**

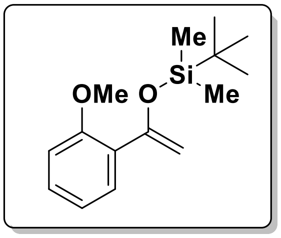

Prepared according to General Procedure **B**, 2-methoxyacetophenone (0.69 mL, 5 mmol) was converted to **S17** yielding a colourless oil (1.23 g, 93%).

^{1}**H NMR** (400 MHz, DMSO-d_6): δ = 7.43 (dt, J = 7.7, 1.6 Hz, 1H), 7.33 – 7.24 (m, 1H), 7.03 (d, J = 8.3 Hz, 1H), 6.94 (td, J = 7.5, 1.2 Hz, 1H), 4.95 (s, 1H), 4.57 (s, 1H), 3.79 (s, 3H), 0.91 (s, 9H), 0.12 (s, 6H) ppm; analytical data in agreement with literature.[20]

tert-butyldimethyl((1-(o-tolyl)vinyl)oxy)silane (S18)

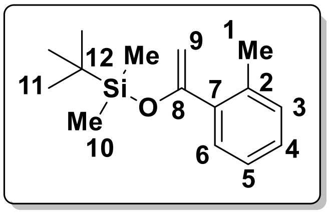

Prepared according to General Procedure **B**, 2-methylacetophenone (0.65 mL, 5 mmol) was converted to **S18** yielding a colourless oil (1.06 g, 85%).

R*f* (100% EtOAc) = 0.89; **^{1}H NMR** (400 MHz, DMSO-*d*$_6$): δ = 7.27 – 7.10 (m, 4H, H3, H4, H5, and H6), 4.56 (d, *J* = 1.1 Hz, 1H, H9), 4.39 (d, *J* = 1.0 Hz, 1H, H9), 2.33 (s, 3H, H1), 0.87 (s, 9H, H11), 0.08 (s, 6H, H10) ppm; **^{13}C NMR** (101 MHz, DMSO-*d*$_6$) δ 157.2 (C8), 138.6 (C7), 135.0 (C2), 130.3 (CAr), 128.2 (CAr), 128.1 (CAr), 125.5 (CAr), 95.7 (C9), 25.5 (C11), 20.1 (C1), 17.9 (C12), -4.8 (C10) ppm; **IR** (ATR) $\tilde{\nu}$ = 2959, 2932, 2889, 2861, 2355, 1630, 1573, 1487, 1473, 1464, 1391, 1363, 1303, 1272, 1254, 1203, 1131, 1090, 1044, 1014, 1004, 940, 834, 811, 780, 769, 729, 692, 658 cm^{-1}; **HRMS** (EI) calcd. for $C_{15}H_{24}OSi$ [M]$^+$ 248.1596, found 248.1635.

N-(4-(1-((_tert_-butyldimethylsilyl)oxy)vinyl)phenyl)acetamide (S19)

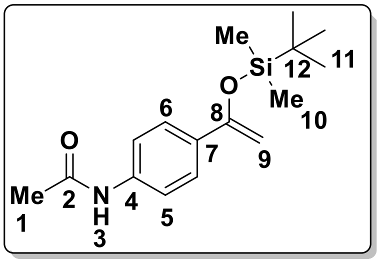

Prepared according to General Procedure **B**, *N*-(4-Acetylphenyl)acetamide (886 mg, 5 mmol) was converted to **S19** yielding a beige solid (1.20 g, 60%) containing 23% 4'-acetamidoacetophenone and 15% *tert*-butyldimethylsilanol as impurities**.**

R*f* (100% EtOAc) = 0.71; **^{1}H NMR** (400 MHz, CD_3CN): δ = 7.58 – 7.46 (m, 4H, H5 and H6), 4.87 (d, *J* = 1.6 Hz, 1H, H9), 4.39 (d, *J* = 1.6 Hz, 1H, H9), 2.05 (s, 3H, H1), 0.99 (s, 9H, H11), 0.20 (s, 6H, H10) ppm; **^{13}C NMR** (101 MHz, CD_3CN): δ = 169.6 (C2), 156.4 (C8), 140.3 (C4), 133.6 (C7), 126.6 (ArCH), 119.6 (ArCH), 90.9 (C9), 26.1 (C11), 24.4 (C1), 18.9 (C12), -4.5 (C10) ppm; **IR** (ATR) $\tilde{\nu}$ = 3295, 3187, 3117, 3059, 2957, 2931, 2887, 2859, 1666, 1597, 1531, 1510, 1472, 1463, 1403, 1372, 1362, 1313, 1293, 1280, 1253, 1181, 1109, 1079, 1012, 1003, 971, 957, 940, 830, 813, 781, 729, 691, 666 cm^{-1}; **HRMS** (ESI) calcd. for $C_{16}H_{26}NO_2Si^+$ [M+H]$^+$ 291.1635, found 291.1655.

3-(1-(3-(4-(1-((_tert_-butyldimethylsilyl)oxy)vinyl)-2-methoxyphenoxy)propyl)piperidin-4-yl)-6-fluorobenzo[d]isoxazole (S20)

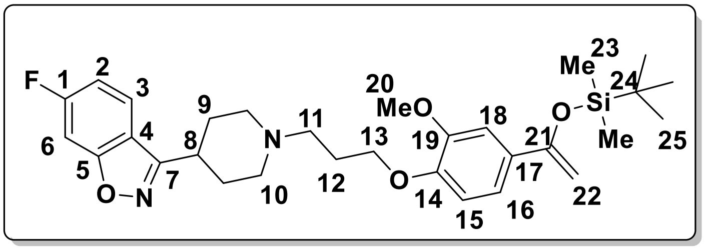

Prepared according to General Procedure **B**, 1-[4-[3-[4-(6-fluoro-1,2-benzoxazol-3-yl)piperidin-1-yl]propoxy]-3-methoxyphenyl]ethanone (427 mg, 1 mmol) was converted to **S20** yielding a white solid (449 mg, 83%)**.**

R*f* (100% EtOAc) = 0.75; **^{1}H NMR** (400 MHz, CD_3CN) δ = 7.83 (dd, *J* = 8.7, 5.2 Hz, 1H, H3), 7.35 (dd, *J* = 8.9, 2.1 Hz, 1H, H6), 7.26 – 7.09 (m, 3H, H18, H15, and H2), 6.91 (d, *J* = 9.0 Hz, 1H, H16), 4.86 (d, *J* = 1.8 Hz, 1H, H22), 4.36 (d, *J* = 1.7 Hz, 1H, H22), 4.06 (t, *J* = 6.7 Hz, 2H, H13), 3.80 (s, 3H, H20), 3.16 – 2.95 (m, 3H, H8 and H10), 2.51 (t, *J* = 7.2 Hz, 2H, H11), 2.13 (td, *J* = 11.6, 2.5 Hz, 2H, H10), 2.05 (ddd, *J* = 12.0, 3.4, 2.0 Hz, 2H, H9), 1.97-1.86 (m, 4H, H12 and H9), 0.99 (s, 9H, H25), 0.20 (s, 6H, H23) ppm; **^{13}C NMR** (101 MHz, CD_3CN) δ = 165.1 (d, J_{CF} = 247.2 Hz, C1), 164.5 (d, J_{CF} = 14.4 Hz, C5), 162.7 (C7), 156.5 (C21), 149.8 (C14), 131.4 (C17), 124.4 (d, J_{CF} = 11.4 Hz, C3), 118.8 (C15), 118.6 (C4), 113.4 (C16), 113.2 (d, J_{CF} = 25.7 Hz, C2), 110.0 (C18), 98.0 (d, J_{CF} = 27.1 Hz, C6), 90.4 (C22), 67.9 (C13), 56.3 (C20), 55.7 (C11), 54.3 (C10), 35.1 (C8),

31.5 (C9), 27.7 (C12), 26.1 (C25), 18.9 (C24), -4.5 (C23) ppm; **^{19}F NMR** (564 MHz, CD_3CN) δ -111.91 (dq, *J* = 9.0, 4.5 Hz) ppm; **IR** (ATR) $\tilde{\nu}$ = 3084, 2955, 2930, 2891, 2857, 2815, 2779, 2166, 1741, 1634, 1612, 1572, 1515, 1498, 1474, 1463, 1449, 1420, 1382, 1332, 1310, 1273, 1253, 1222, 1180, 1148, 1132, 1116, 1083, 1051, 1038, 1011, 1002, 958, 948, 914, 895, 866, 831, 819, 794, 780, 760, 746, 693, 667, 655 cm^{-1}; **HRMS** (ESI) calcd. for $C_{30}H_{41}FN_2O_4SiNa^+$ $[M+Na]^+$ 563.2712, found 563.2744.

***tert*-butyldimethyl((1-(thiophen-2-yl)vinyl)oxy)silane (S21)**

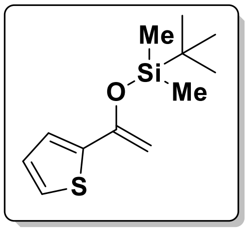

S21 was prepared according to a procedure of Yamada *et al.*[20] To an oven-dried flask under argon atmosphere was added 1-(thiophen-2-yl)ethan-1-one (0.54 mL, 5 mmol) and anhydrous acetonitrile (11 mL, 0.45 M), followed by the addition of NaI (1.05 g, 7 mmol, 1.4 equiv.) and trimethylamine (1.1 mL, 7.5 mmol, 1.5 equiv.). The reaction mixture was cooled to 0 ºC before the addition of *tert*-butyldimethylsilyl chloride (980 mg, 6.5 mmol, 1.3 equiv.). The reaction mixture was allowed to warm to ambient temperature and stirred overnight. The reaction was quenched by addition of sat. aq. $NaHCO_3$ and extracted once with *n*-hexane and separated into aqueous, acetonitrile, and *n*-hexane layers. The acetonitrile layer was further extracted twice with *n*-hexane. The combined *n*-hexane layers were washed with brine, dried over anhydrous Na_2SO_4 and the solvent was removed *in vacuo*. The crude residue was purified via column chromatography on deactivated silica (5 % Et_3N in *n*-hexane) using *n*-hexane as eluent, yielding **S21** as a colourless oil (560 mg, 89%).
^{1}H NMR (400 MHz, DMSO-*d*$_6$): δ = 7.47 (dd, *J* = 5.0, 1.1 Hz, 1H), 7.28 (dd, *J* = 3.7, 1.2 Hz, 1H), 7.03 (dd, *J* = 5.0, 3.7 Hz, 1H), 4.88 (d, *J* = 2.1 Hz, 1H), 4.32 (d, *J* = 2.1 Hz, 1H), 0.97 (s, 9H), 0.21 (s, 6H) ppm; analytical data in agreement with literature.[20]

N-methyl-N-phenylmethacrylamide (S22)

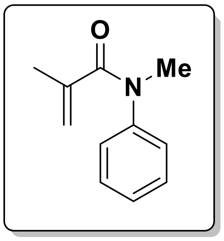

To an oven dried flask kept under argon were added methylaniline (1.1 mL, 10 mmol, 1 equiv.) and triethylamine (1.7 mL, 12 mmol, 1.2 equiv.). Dry DCM (30 mL) was added with a syringe and the resulting solution was placed in an ice bath. Methacryloyl chloride (1.2 mL, 12 mmol, 1.2 equiv.) was added dropwise. The reaction mixture was allowed to stir at ambient temperature overnight. After completion, the reaction was quenched by the addition of sat. aq. $NaHCO_3$ (20 mL). The organic phase was separated, washed with 1 M HCl (2 x 20 mL), dried over Na_2SO_4, filtered and dried under reduced pressure. The crude residue was purified by flash column chromatography (SiO_2, DCM) to yield **S22** as a white solid (945 mg, 54%).

^{1}H NMR (400 MHz, $CDCl_3$): δ = 7.35 (td, *J* = 7.1, 1.7 Hz, 2H), 7.29 – 7.22 (m, 1H), 7.14 (dd, *J* = 8.4, 1.3 Hz, 2H), 5.03 (s, 1H), 4.98 (s, 1H), 3.35 (s, 3H), 1.76 (s, 3H) ppm; analytical data in agreement with literature.[21]

Experimental Setup for Photoreactions

The photocatalyst-free reactions yielding boronic esters were carried out using a Kessil PR160L-390 (390 nm) LED lamps with the producer´s power settings.[22] The lamps was placed 4.5 cm away from the reaction vial, that was placed in the middle of a stirring plate (Roth Rotilabo-Mini-Magnetrührer M3 Stirring speed 550 rpm). To regulate temperature, a fan was used for cooling.

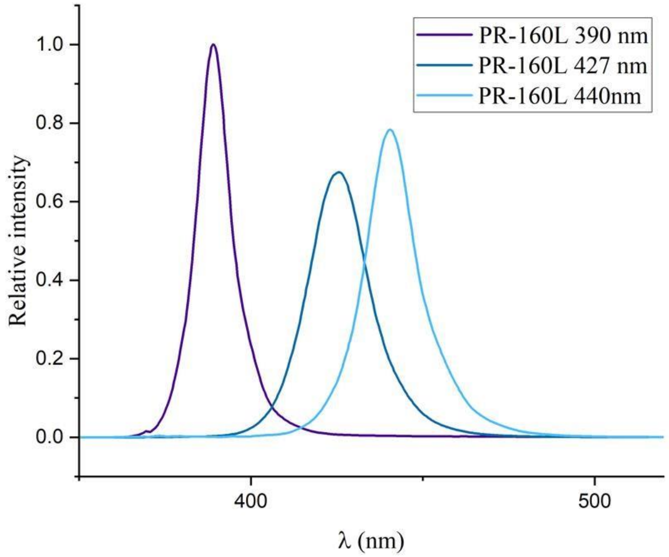

Figure S1. Emission spectra of Kessil PR160L-390, Kessil PR160L-427 and Kessil PR160L-440.

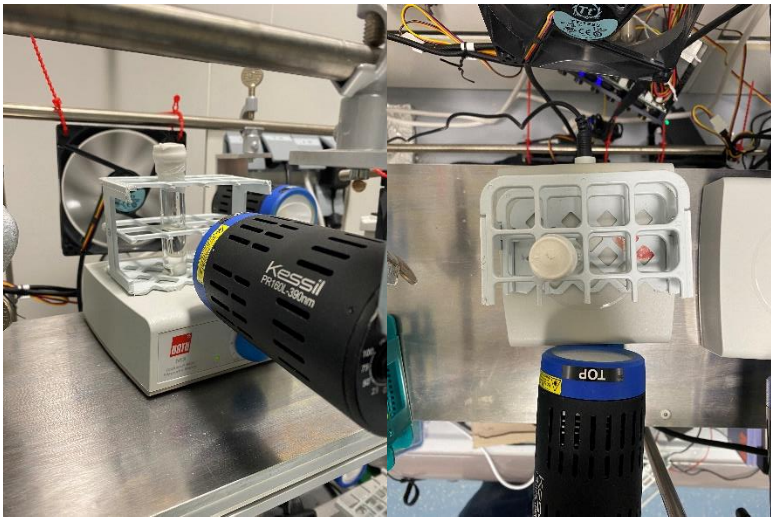

Figure S2. Reaction set up for non-photocatalyst reaction; Kessil PR160L-390. (Left: side view, right: top view)

The reactions yielding diene boronic esters were carried out using 2 Kessil PR160L-427 (427 mm) LED lamps with the producer´s power settings. The lamps were placed 12 cm apart, with the reaction vial placed

in the middle on a stirring plate (Roth Rotilabo-Mini-Magnetrührer M3 Stirring speed 550 rpm). To regulate temperature, a fan was used for cooling.

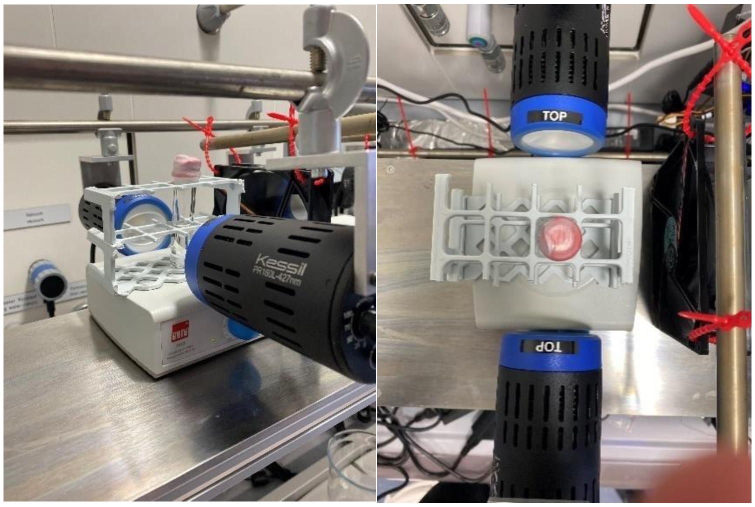

Figure S3. Reaction set up for *E*-selective reaction; Kessil PR160L-427. (Left: side view, right: top view)

The reactions yielding *Z*-allylic boronic esters were carried out using a Kessil PR160L-440 (440 mm) LED lamps with the producer´s power settings. The lamp was placed 4.5 cm away from the reaction vial, that was placed in the middle of a stirring plate (Roth Rotilabo-Mini-Magnetrührer M3 Stirring speed 550 rpm). To regulate temperature, a fan was used for cooling.

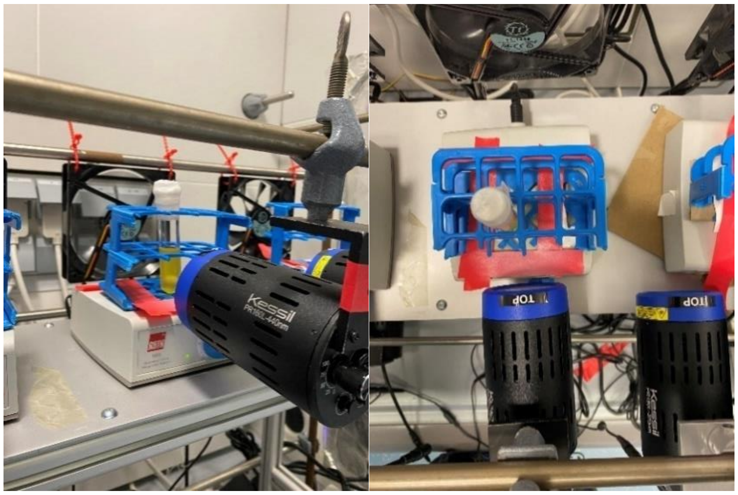

Figure S4. Reaction set up for *Z*-selective reaction; Kessil PR160L-440. (Left: side view, right: top view)

Reaction Optimization: *E*-Allylic Boronic Esters

Table S1. Effect of light source.

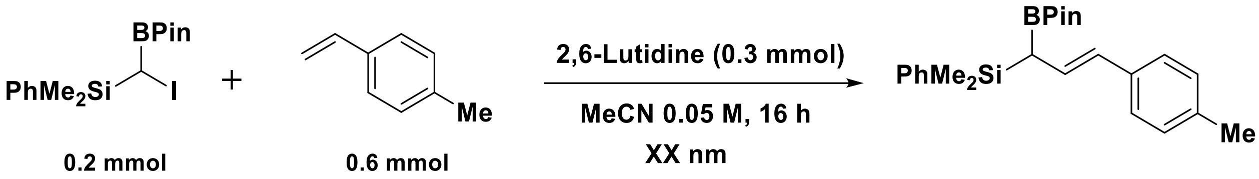

Entry	Light source (nm)	Yield[a] (%)	*E*:*Z* ratio[a]
1	370	37	86:14
**2**	**390**	**69**	**89:11**
3	2x427	43	86:14

[a] Determined by ^{1}H NMR spectroscopy against a known internal standard (1,3,5-trimethoxybenzene).

Table S2. Effect of concentration.

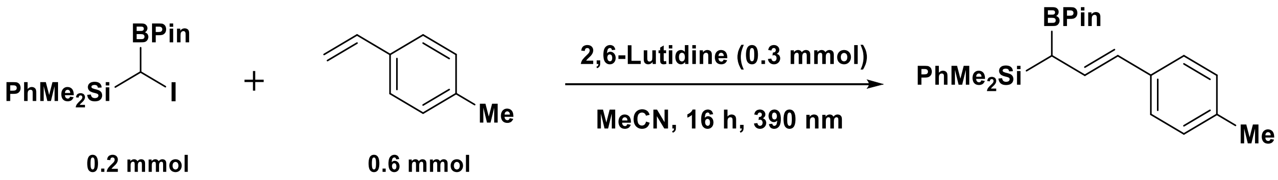

Entry	Concentration (M)	Yield[a] (%)	*E*:*Z* ratio[a]
4	0.1	67	86:14
5	0.025	25	88:12
**2**	**0.05**	**69**	**89:11**

[a] Determined by ^{1}H NMR spectroscopy against a known internal standard (1,3,5-trimethoxybenzene).

Table S3. Solvent screening.

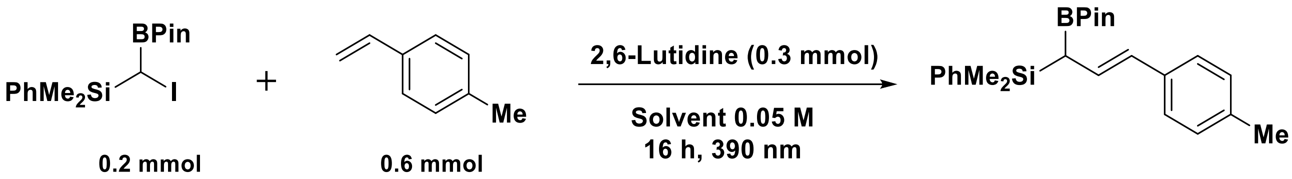

Entry	Solvent	Yield[a] (%)	*E*:*Z* ratio[a]
6	DMF	0	-
7	DCM	55	87:13
8	Toluene	64	94:6
9	THF	3	>95:5
**2**	**MeCN**	**69**	**89:11**

[a] Determined by ^{1}H NMR spectroscopy against a known internal standard (1,3,5-trimethoxybenzene).

Table S4. Additive screening.

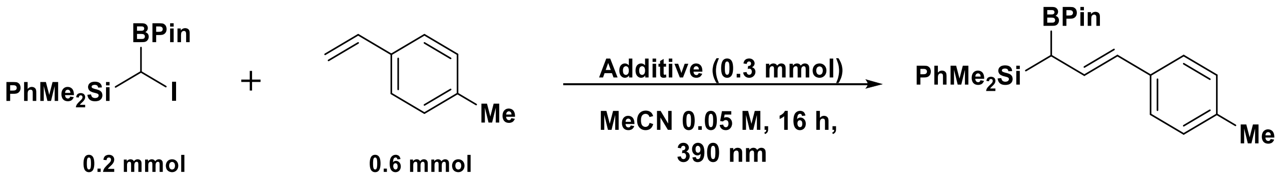

Entry	Additive	Yield[a] (%)	*E*:*Z* ratio[a]
10	2,6-Di-*tert*-butylpyridine	66	86:14
11	2,6-Dimethyl-4-dimethylaminopyridine	46	74:26
12	DMAP	34	38:62
13	Pyridine	28	79:21
14	3-Quinuclidinol	55	64:36
15	Guanidine	38	47:53
16	Et_3N	0	-
17	DIPEA	0	-
18	TMEDA	0	-
19	PPh_3	20	>95:5
20	KOAc	0	-
21	Cs_2CO_3	11	>95:5
**2**	**2,6-Lutidine**	**69**	**89:11**

[a] Determined by ^1H NMR spectroscopy against a known internal standard (1,3,5-trimethoxybenzene).

Comment: The additive screening showcased that both Lewis basicity and steric hindrance were important for efficient reactivity to take place. Less steric hindrance around the nitrogen (Entry 12 and 13) resulted in a drop in yield compared to more sterically hindered Lewis bases (Entry 2, 10 and 11). An increase in Lewis basicity (Entry 11, 14 and 15) also resulted in a drop in yield and selectivity. These results could be due to competing boron speciation occurring between substrate and Lewis base.

Table S5. Effect of styrene equivalents.

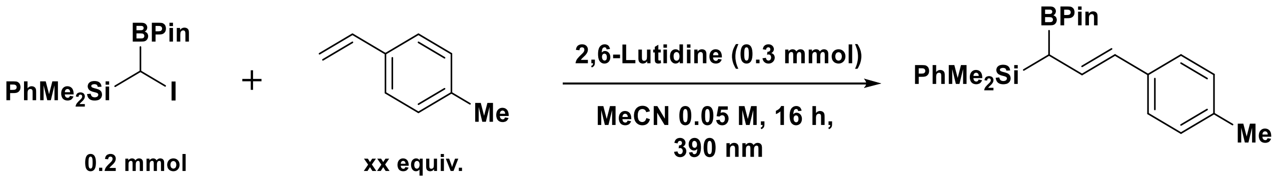

Entry	Styrene equivalents	Yield[a] (%)	*E*:*Z* ratio[a]
22	2	37	84:16
23	4	55	89:11
**2**	**3**	**69**	**89:11**

[a] Determined by ^1H NMR spectroscopy against a known internal standard (1,3,5-trimethoxybenzene).

Substrate Scope

General Procedure C: Light-mediated generation of *E*-allyl boron pinacol esters

To an oven-dried 5 mL microwave vial, the specified α-iodo boronic acid, pinacol ester (0.2 mmol, 1 equiv.) was added. The vial was sealed with a septum and purged with nitrogen before the sequential addition of degassed MeCN (4 mL), 2,6-lutidine (35 μL, 0.3 mmol, 1.5 equiv.) and styrene (0.6 mmol, 3 equiv.) via syringe. The styrene, when solid, was weighed directly prior to sealing and purging. The reaction mixture was stirred under light irradiation (390 nm, PR-160L) for 16 h. After completion, internal standard (1,3,5-trimethoxybenzene), as a solution in MeCN, was added and the reaction was washed with a 1 M sol. of K_2CO_3 (10 mL). Organics were extracted with EtOAc (3 x 10 mL). The combined organic phases were dried over Na_2SO_4, filtered and concentrated under reduced pressure. The crude residue was purified by flash column chromatography (SiO_2, specified combination of solvents). Borylated products purified by SiO_2 were exposed to the minimum amount of SiO_2 for as little time as possible to limit degradation.

General Procedure D: Light-mediated generation of aryl ketone boron pinacol esters

To an oven-dried 5 mL microwave vial, the specified α-iodo boronic acid, pinacol ester (0.2 mmol, 1 equiv.) and TBS-silyl enol ether (0.6 mmol, 3 equiv.) was added. The vial was sealed with a septum and purged with nitrogen before the sequential addition of degassed MeCN (2 mL), degassed water (72 μL, 4 mmol, 20 equiv.) and 2,6-lutidine (35 μL, 0.3 mmol, 1.5 equiv.) via syringe. The reaction mixture was stirred under light irradiation (390 nm, PR-160L) for 16 h. After completion, internal standard (1,3,5-trimethoxybenzene), as a solution in MeCN, was added and the reaction was washed with a 1 M sol. of K_2CO_3 (10 mL). Organics were extracted with EtOAc (3 x 10 mL). The combined organic phases were dried over Na_2SO_4, filtered and concentrated under reduced pressure. The crude residue was purified by flash column chromatography (SiO_2, specified combination of solvents). Borylated products purified by SiO_2 were exposed to the minimum amount of SiO_2 for as little time as possible to limit degradation.

General Procedure E: Light-mediated generation of diene boron pinacol esters

To an oven-dried 5 mL microwave vial, the specified α-iodo boronic acid, pinacol ester (0.2 mmol, 1 equiv.) was added. The vial was sealed with a septum and purged with nitrogen before the sequential addition of degassed MeCN (4 mL), 2,6-di-tert-butylpyridine (67 μL, 0.3 mmol, 1.5 equiv.) and 2,3-dimethyl-1,3-butadiene (68 μL, 0.6 mmol, 3 equiv.) via syringe. The reaction mixture was stirred under light irradiation (427 nm, 2 x PR-160L) for 48 h. After completion, internal standard (1,3,5-trimethoxybenzene), as a solution in MeCN, was added and the reaction was washed with a 1 M sol. of K_2CO_3 (10 mL). Organics were extracted with EtOAc (3 x 10 mL). The combined organic phases were dried over Na_2SO_4, filtered and concentrated under reduced pressure. The crude residue was purified by flash column chromatography (SiO_2, specified combination of solvents). Borylated products purified by SiO_2 were exposed to the minimum amount of SiO_2 for as little time as possible to limit degradation.

General Procedure F: Light-mediated generation of oxindole boron pinacol esters

To an oven-dried 5 mL microwave vial, the specified α-iodo boronic acid, pinacol ester (0.2 mmol, 1 equiv.) and *N*-methyl-*N*-phenylmethacrylamide (70.1 mg, 0.4 mmol. 2 equiv.) was added. The vial was sealed with a septum and purged with nitrogen before the sequential addition of degassed MeCN (4 mL) and 2,6-lutidine (35 μL, 0.3 mmol, 1.5 equiv.) via syringe. The reaction mixture was stirred under light irradiation (390 nm, PR-160L) for 16 h. After completion, internal standard (1,3,5-trimethoxybenzene), as a solution

in MeCN, was added and the reaction was washed with a 1 M sol. of K_2CO_3 (10 mL). Organics were extracted with EtOAc (3 x 10 mL). The combined organic phases were dried over Na_2SO_4, filtered and concentrated under reduced pressure. The crude residue was purified by flash column chromatography (SiO_2, specified combination of solvents). Borylated products purified by SiO_2 were exposed to the minimum amount of SiO_2 for as little time as possible to limit degradation.

(*E*)-(1-(Dimethyl(phenyl)silyl)-3-(*p*-tolyl)allyl)boronic acid, pinacol ester (3)

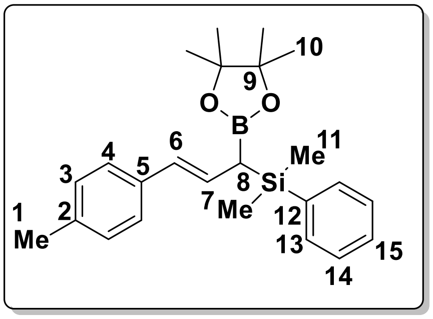

Prepared according to General Procedure **C**, **1a** (80.4 mg, 0.2 mmol), and 4-methylstyrene (79 µL, 0.6 mmol) were converted to **3** yielding a yellow oil (69% NMR yield, 89:11 (*E*:*Z*), 37.0 mg, 90:10 (*E*:*Z*) 47%), after purification by flash column chromatography (SiO_2, 0→0.5% EtOAc/*n*-hexane; C18, 20→100% MeCN/H_2O).

R_f (10% EtOAc/*n*-hexane) = 0.62; **^{1}H NMR** (400 MHz, $CDCl_3$) δ = 7.58 – 7.53 (m, 2H, H14), 7.39 – 7.30 (m, 3H, H13 and H15), 7.18 (d, *J* = 7.8 Hz, 2H, H4), 7.10 – 7.05 (m, 2H, H3), 6.26 (dd, *J* = 15.8, 10.5 Hz, 1H, H7), 6.07 (d, *J* = 15.7 Hz, 1H, H6), 2.32 (s, 3H, H1), 1.91 (d, *J* = 10.5 Hz, 1H, H8), 1.18 (s, 6H, H10), 1.14 (s, 6H, H10), 0.40 (s, 3H, H11), 0.38 (s, 3H, H11) ppm; **^{13}C NMR** (101 MHz, $CDCl_3$) δ = 138.2 (C12), 136.1 (C5), 135.7 (C2), 134.2 (C14), 129.2 (C3), 129.1 (C15), 127.7 (C6), 127.7 (C13), 127.4 (C7), 125.6 (C4), 83.2 (C9), 25.1 (C10), 25.0 (C10), 21.2 (C1), -2.9 (C11) ppm.; **^{11}B NMR** (128 MHz, $CDCl_3$) δ = 33.54 ppm.; **IR** (ATR) ṽ = 3072, 3050, 2979, 2925, 1899, 1636, 1514, 1441, 1428, 1411, 1372, 1352, 1316, 1300, 1265, 1249, 1213, 1166, 1140, 1113, 1027, 970, 857, 835, 817, 803, 778, 750, 737, 726, 699, 665 cm^{-1}; **HRMS** (EI) calcd. for $C_{24}H_{33}BO_2Si$ $[M]^+$ 392.2343, found 392.2324.

(*E*)-(1-(Dimethyl(phenyl)silyl)-3-(4-fluorophenyl)allyl)boronic acid, pinacol ester (4)

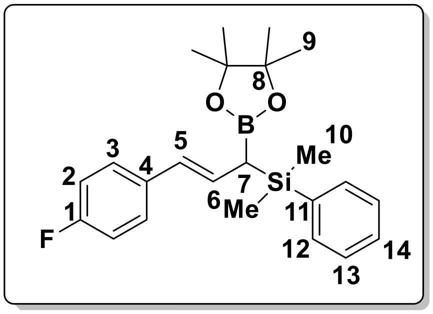

Prepared according to a modified version of General Procedure **C**, in which the reaction was stirred under 2 x 427 nm lamps and 2,6-*tert*-butyl pyridine was used as base, **1a** (80.4 mg, 0.2 mmol), and 4-fluorostyrene (72 µL, 0.6 mmol) were converted to **4** yielding a yellow oil (78% NMR yield, 85:15 (*E*:*Z*), 31.3 mg, >95:5 (*E*:*Z*) 40%), after purification by flash column chromatography (C18, 20→100% MeCN/H_2O).

R_f (10% EtOAc/*n*-hexane) = 0.48; **^{1}H NMR** (400 MHz, $CDCl_3$) δ = 7.56 – 7.51 (m, 2H, H13), 7.38 – 7.31 (m, 3H, H12 and H14), 7.23 – 7.17 (m, 2H, H2), 6.94 (t, *J* = 8.7 Hz, 2H, H3), 6.21 (dd, *J* = 15.8, 10.5 Hz, 1H, H6), 6.03 (d, *J* = 15.7 Hz, 1H, H5), 1.90 (d, *J* = 10.5 Hz, 1H, H7), 1.18 (s, 6H, H9), 1.15 (s, 6H, H9), 0.40 (s, 3H, H10), 0.38 (s, 3H, H10) ppm; **^{13}C NMR** (101 MHz, $CDCl_3$) δ = 161.5 (d, J_{CF} = 244.4 Hz, C1) , 138.0 (C11), 135.0 (d, J_{CF} = 3.3 Hz, C4) , 134.2 (C13), 129.2 (C14), 128.3 (d, C6), 127.7 (C12), 127.0 (d, J_{CF} = 7.7 Hz, C3) , 126.7 (C5), 115.2 (d, J_{CF} = 21.3 Hz, C2), 83.2 (C8), 25.0 (d, C9), -2.9 (d, C10) ppm; **^{11}B NMR** (128 MHz, $CDCl_3$) δ = 32.57 ppm; **^{19}F NMR** (376 MHz, $CDCl_3$) δ = 117.1 (m) ppm; **IR** (ATR): ῦ = 3070, 2981, 2933, 1901, 1736, 1639, 1593, 1509, 1481, 1462, 1428, 1414, 1383, 1372, 1354, 1308, 1298, 1264, 1251, 1225, 1162, 1138, 1113, 1082, 1027, 999, 972, 943, 906, 860, 829, 810, 785, 773, 750, 725, 708, 698 cm^{-1}; **HRMS** (EI) cacld. for $C_{23}H_{30}BFO_2Si$ $[M]^+$ 396.2092, found 396.2052.

(1-(Dimethyl(phenyl)silyl)-3,3-diphenylallyl)boronic acid, pinacol ester (5)

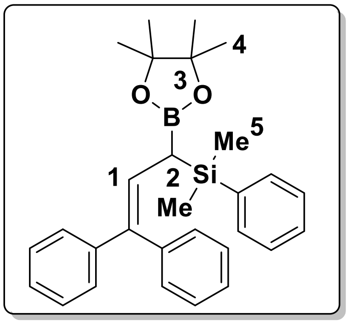

Prepared according to General Procedure **C**, **1a** (80.4 mg, 0.2 mmol), and diphenylethylene (106 µL, 0.6 mmol) were converted to **5** yielding a yellow oil (90% NMR yield, 74.5 mg, 86%), after purification by flash column chromatography (SiO_2, 0→1% EtOAc/*n*-hexane; C18, 20→100% $MeCN/H_2O$).

R*f* (10% EtOAc/*n*-hexane) = 0.46; **^{1}H NMR** (400 MHz, $CDCl_3$) δ = 7.42 (dd, *J* = 7.6, 1.9 Hz, 2H), 7.34 – 7.16 (m, 8H), 7.15 – 7.06 (m, 3H), 6.89 (dd, *J* = 8.1, 1.5 Hz, 2H), 6.22 (d, *J* = 12.5 Hz, 1H, H1), 2.10 (d, *J* = 12.5 Hz, 1H, H2), 1.18 (s, 6H, H4), 1.15 (s, 6H, H4), 0.33 (s, 3H, H5), 0.31 (s, 3H, H5) ppm; **^{13}C NMR** (101 MHz, $CDCl_3$) δ = 143.7, 140.4, 138.5, 138.0, 134.1, 130.4, 129.1, 128.3, 128.0, 127.7, 127.2 (C1), 127.1, 126.5, 126.2, 83.1 (C3), 25.1 (C4), 24.9 (C4), 20.7 (C2) -2.5 (C5), -2.8 (C5) ppm; **^{11}B NMR** (128 MHz, $CDCl_3$) δ = 33.95 ppm.; **IR** (ATR): ῦ = 3055, 3023, 2979, 1598, 1496, 1444, 1428, 1372, 1333, 1311, 1249, 1214, 1166, 1142, 1113, 1072, 1026, 1000, 969, 906, 846, 833, 814, 776, 764, 729, 698, 664 cm^{-1}; **HMRS** (EI) calcd. for $C_{29}H_{35}BO_2Si$ $[M]^+$ 454.2499, found 454.2489.

(*E*)-(1-(Dimethyl(phenyl)silyl)-3-phenylallyl)boronic acid, pinacol ester (6)

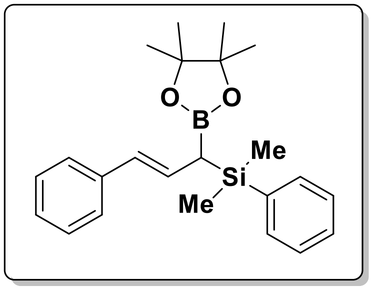

Prepared according to a modified version of General Procedure **C**, in which the reaction was stirred for 24 h, **1a** (80.4 mg, 0.2 mmol), and styrene (69 µL, 0.6 mmol) were converted to **6** yielding a yellow oil (52% NMR yield, 85:15 (*E*:*Z*), 29.8 mg, 87:13 (*E*:*Z*) 39%), after purification by flash column chromatography (SiO_2, 0→0.5% EtOAc/*n*-hexane; C18, 20→100% $MeCN/H_2O$).

^{1}H NMR (400 MHz, $CDCl_3$) δ = 7.55 – 7.52 (m, 2H), 7.37 – 7.29 (m, 3H), 7.28 – 7.23 (m, 4H), 7.16 – 7.10 (m, 1H), 6.34 – 6.26 (m, 1H), 6.09 (d, *J* = 15.7 Hz, 1H), 1.92 (d, *J* = 10.6 Hz, 1H), 1.17 (s, 6H), 1.13 (s, 6H), 0.40 (s, 3H), 0.37 (s, 3H) ppm; analytical data in agreement with literature.[2]

(*E*)-(3-(Benzofuran-5-yl)-1-(dimethyl(phenyl)silyl)allyl)boronic acid, pinacol ester (7)

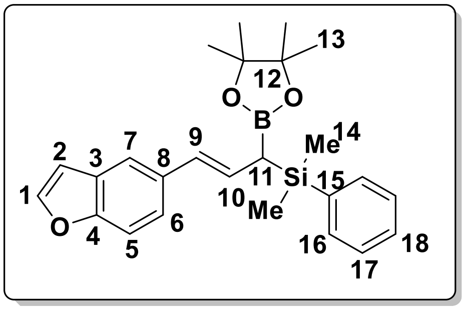

Prepared according to General Procedure **C**, **1a** (80.4 mg, 0.2 mmol), and **S5** (86.5 mg, 0.6 mmol) were converted to **7** yielding a pale yellow oil (66% NMR yield, 86:14 (*E*:*Z*), 42.0 mg, 94:6 (*E*:*Z*), 50%), after purification by flash column chromatography (SiO_2, 0→2% EtOAc/*n*-hexane; C18, 20→100% $MeCN/H_2O$).

R*f* (10% EtOAc/*n*-hexane) = 0.50; **^{1}H NMR** (400 MHz, $CDCl_3$) δ = 7.62 – 7.55 (m, 3H, H1, H17), 7.49 (m, 1H, H7), 7.43 – 7.33 (m, 5H, H5, H16 and H18), 7.28 (s, 1H, H6), 6.73 (s, 1H, H2), 6.31 (dd, *J* = 15.7, 10.1 Hz, 1H, H10), 6.21 (m, 1H, H9), 1.96 (d, *J* = 10.1 Hz, 1H, H11), 1.22 (s, 6H, H13), 1.18 (s, 6H, H13), 0.45 (s, 3H, H14), 0.42 (s, 3H, H14) ppm; **^{13}C NMR** (101 MHz, $CDCl_3$) δ = 154.0 (C4), 145.3 (C1), 138.2 (C15), 134.2 (C17), 134.0 (C8), 129.1 (C18), 127.9 (C9), 127.7 (C3), 127.7 (C16), 127.2 (C10), 122.5 (C6), 117.9 (C7), 111.2 (C5), 106.7 (C2), 83.2 (C12), 25.1 (C13), 25.0 (C13), -2.8 (C14), -2.9 (C14) ppm; **^{11}B NMR** (128 MHz, $CDCl_3$) δ = 33.12 ppm; **IR** (ATR) ῦ = 3115, 3073, 3052, 2981, 2344, 2302, 1815, 1635, 1537, 1468, 1441, 1429, 1380, 1372, 1356, 1329, 1316, 1290, 1264, 1247, 1211, 1196, 1166, 1137, 1126, 1110, 1032, 1019, 969, 879, 855, 845, 832,

807, 782, 766, 738, 715, 696, 660 cm^{-1}; **HRMS** (EI) calcd. for $C_{25}H_{31}BO_3Si$ $[M]^+$ 418.2136, found 418.2178.

(*E*)-(3-(*p*-Tolyl)-1-(triethylgermyl)allyl)boronic acid, pinacol ester (8)

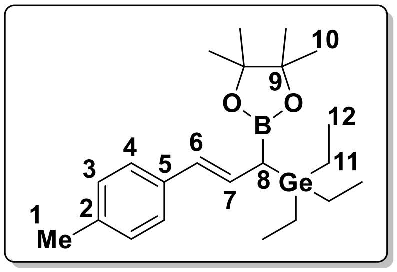

Prepared according to General Procedure **C**, **1b** (113.8 mg, 0.2 mmol), and p-Methyl Styrene (79 µL, 0.6 mmol) were converted to **8** yielding a pale yellow oil (29% NMR yield, 79:21 (E:Z), 20.9 mg 85:15 (E:Z), 25%), after purification by flash column chromatography (SiO_2, 0→1% EtOAc/*n*-hexane; C18, 20→100% MeCN/H_2O).

$\mathbf{R}_f$ (10% EtOAc/*n*-hexane) = 0.57; **^{1}H NMR** (400 MHz, $CDCl_3$) δ 7.21 (d, *J* = 8.2 Hz, 2H, H4), 7.07 (d, *J* = 8.0 Hz, 2H, H3), 6.36 (dd, *J* = 15.6, 11.3 Hz, 1H, H7), 6.11 (d, *J* = 15.7 Hz, 1H, H6), 2.31 (s, 3H, H1), 1.93 (d, *J* = 11.3 Hz, 1H, H8), 1.25 (s, 6H, H10), 1.24 (s, 6H, H10), 1.08 – 1.03 (m, 9H, H12), 0.89 – 0.83 (m, 6H, H11) ppm; **^{13}C NMR** (101 MHz, $CDCl_3$) δ 136.3 (C5), 135.5 (C2), 129.5 (C7), 129.2 (C3), 125.7 (C6), 125.4 (C4), 83.0 (C9), 25.2 (C10), 25.1 (C10), 21.2 (C1), 9.2 (C12), 4.6 (C11) ppm; **^{11}B NMR** (128 MHz, $CDCl_3$) δ = 33.10 ppm; **IR** (ATR): ῦ = 2980, 2952, 2931, 2908, 2872, 2385, 2359, 2344, 2299, 1636, 1513, 1459, 1425, 1381, 1359, 1314, 1300, 1264, 1215, 1166, 1140, 1110, 1084, 1023, 1004, 970, 902, 857, 843, 805, 772, 702, 664 cm^{-1}; **HMRS** (EI) calcd. for $C_{22}H_{37}BGeO_2$ $[M]^+$ 418.2098, found 418.2074.

(*E*)-(3-(*p*-Tolyl)prop-2-ene-1,1-diyl)diboronic acid, pinacol ester (9)

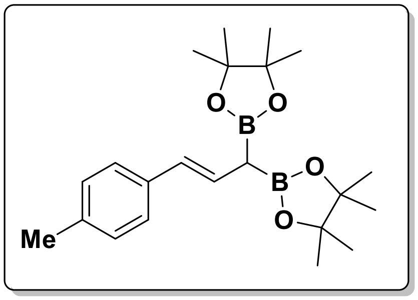

Prepared according to a modified version of General Procedure **C**, in which the reaction was washed with sat. aq. NH_4Cl instead of 1 M K_2CO_3, **1c** (78.8 mg, 0.2 mmol), and 4-methylstyrene (79 µL, 0.6 mmol) were converted to **9** yielding a white solid (81% NMR yield, 90:10 (*E*:*Z*), 31.7 mg, >95:5 (*E*:*Z*) 41%), after purification by flash column chromatography (C18, 20→100% MeCN/H_2O (0.1% Formic Acid)) and recrystallization in *n*-hexane. (Note: Basic work-up lead to protodeboronation of desired product)

^{1}H NMR (400 MHz, $CDCl_3$) δ = 7.24 (d, *J* = 8.1 Hz, 2H), 7.07 (d, *J* = 7.8 Hz, 2H), 6.36 (dd, *J* = 15.7, 9.8 Hz, 1H), 6.24 (d, *J* = 15.7 Hz, 1H), 2.30 (s, 3H), 1.98 (d, *J* = 9.8 Hz, 1H), 1.24 (s, 12H), 1.23 (s, 12H) ppm; analytical data in agreement with literature.[23]

(*E*)-(3-(4-Methoxyphenyl)prop-2-ene-1,1-diyl)diboronic acid, pinacol ester (10)

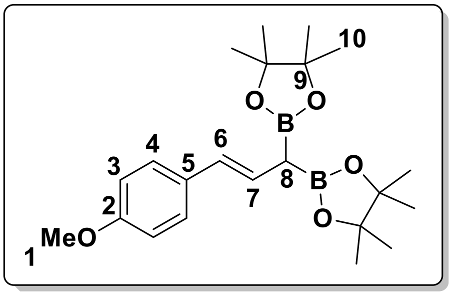

Prepared according to a modified version of General Procedure **C**, in which the reaction was washed with sat. aq. NH_4Cl instead of 1 M K_2CO_3, **1c** (78.8 mg, 0.2 mmol), and 4-methoxystyrene (80 µL, 0.6 mmol) were converted to **10** yielding an off-white solid (70% NMR yield, >95:5 (*E*:*Z*), 42.4 mg, 53%), after purification by flash column chromatography (C18, 20→100% MeCN/H_2O (0.1% formic acid)). (Note: Basic work-up lead to protodeboronation of desired product)

^{1}H NMR (400 MHz, $CDCl_3$) δ = 7.27 (d, *J* = 8.7 Hz, 2H, H4), 6.80 (d, *J* = 8.7 Hz, 2H, H3), 6.29 – 6.20 (m, 2H, H6 and H7), 3.78 (s, 3H, H1), 1.95 (d, *J* = 8.9 Hz, 1H, H8), 1.24 (s, 12H, H10), 1.23 (s, 12H, H10) ppm; **^{13}C NMR** (101 MHz, $CDCl_3$) δ = 158.3 (C2), 131.7 (C5), 128.0 (C6), 127.0 (C4), 125.5 (C7), 113.8 (C3), 83.5 (C9), 55.4 (C1), 24.8 (C10) ppm; **^{11}B NMR** (128 MHz, $CDCl_3$) δ = 32.88 ppm; **IR** (ATR): ῦ =

3401, 3035, 2981, 2932, 2839, 1646, 1609, 1577, 1511, 1462, 1444, 1381, 1364, 1329, 1299, 1282, 1263, 1244, 1213, 1170, 1134, 1110, 1058, 1034, 1006, 967, 906, 855, 822, 811, 768, 737, 670 cm^{-1}; **HRMS** (EI) calcd. for $C_{22}H_{34}B_2O_5$ $[M]^+$ 400.2592, found 400.2607.

(3-(4-Bromophenyl)-1-(dimethyl(phenyl)silyl)-3-oxopropyl)boronic acid, pinacol ester (11)

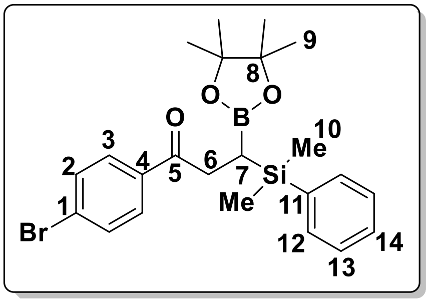

Prepared according to General Procedure **D**, **1a** (80.4 mg, 0.2 mmol), and **S12** (188 mg, 0.6 mmol) were converted to **11** yielding a white solid (72% NMR yield, 46.2 mg, 49%), after purification by flash column chromatography (SiO_2, 0→2% EtOAc/*n*-hexane; C18, 20→100% MeCN/H_2O).

R_f (10% EtOAc/*n*-hexane) = 0.39; **^{1}H NMR** (400 MHz, $CDCl_3$) δ = 7.72 (d, *J* = 8.6 Hz, 2H, H3), 7.58 – 7.54 (m, 2H, H13), 7.52 (d, *J* = 8.4 Hz, 2H, H2), 7.40 – 7.32 (m, 3H, H12 and H14), 3.14 (dd, *J* = 18.4, 12.1 Hz, 1H, H6), 2.88 (dd, *J* = 18.3, 3.5 Hz, 1H, H6), 1.24 (s, 6H, H9), 1.15 (s, 6H, H9), 1.08 (dd, *J* = 12.1, 3.5 Hz, 1H, H7), 0.40 (s, 3H, H10), 0.38 (s, 3H, H10) ppm; **^{13}C NMR** (101 MHz, $CDCl_3$) δ = 199.5 (C5), 138.3 (C11), 135.8 (C1), 133.9 (C13), 131.8 (C2), 129.6 (C3), 129.2 (C14), 128.0 (C12), 127.8 (C4), 83.1 (C8), 36.2 (C6), 25.1 (C9), 24.8 (C9), -2.0 (C10), -3.5 (C10) ppm; **^{11}B NMR** (128 MHz, $CDCl_3$) δ = 33.54 ppm; **IR** (ATR): ῦ = 3729, 3107, 3068, 2975, 2930, 2893, 2345, 2297, 1684, 1586, 1483, 1464, 1428, 1399, 1382, 1371, 1351, 1307, 1250, 1212, 1180, 1165, 1144, 1112, 1070, 1034, 1010, 981, 970, 937, 879, 853, 838, 814, 769, 738, 702, 672, 665 cm^{-1}; **HRMS** (EI) calcd. for $C_{23}H_{30}BBrO_3Si$ $[M]^+$ 472.1241, found 472.1201.

(1-(Dimethyl(phenyl)silyl)-3-(naphthalen-2-yl)-3-oxopropyl)boronic acid, pinacol ester (12)

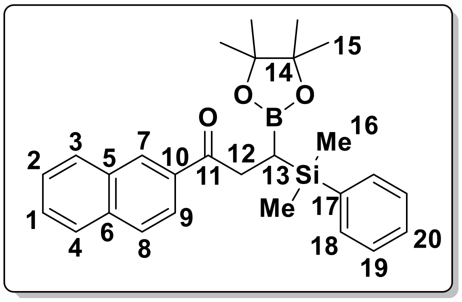

Prepared according to General Procedure **D**, **1a** (80.4 mg, 0.2 mmol), and **S11** (171 mg, 0.6 mmol) were converted to **12** yielding a white solid (78% NMR yield, 50.6 mg, 57%), after purification by flash column chromatography (SiO_2, 0→2% EtOAc/*n*-hexane; C18, 20→100% MeCN/H_2O).

R_f (10% EtOAc/*n*-hexane) = 0.38; **^{1}H NMR** (400 MHz, $CDCl_3$) δ = 8.37 (s, 1H, H7), 7.96 (dd, *J* = 8.6, 1.7 Hz, 1H), 7.92 (d, *J* = 1.4 Hz, 1H), 7.86 – 7.81 (m, 2H), 7.66 – 7.60 (m, 2H, H19), 7.60 – 7.49 (m, 2H), 7.42 – 7.35 (m, 3H, H18 and H20), 3.34 (dd, *J* = 18.2, 12.1 Hz, 1H, H12), 3.10 (dd, *J* = 18.2, 3.5 Hz, 1H, H12), 1.26 (s, 6H, H15), 1.18 (s, 7H, H13 and H15), 0.46 (s, 3H, H16), 0.42 (s, 3H, H16) ppm; **^{13}C NMR** (101 MHz, $CDCl_3$) δ = 200.4 (C11), 138.5 (C17), 135.5 (C10), 134.4, 133.9 (C19), 132.6, 129.6, 129.4 (C7), 129.2 (C20), 128.3, 128.2, 128.0 (C18), 127.8, 126.7, 124.2, 83.1 (C14), 36.2 (C12), 25.1 (C15), 24.8 (C15), -1.9 (C16), -3.4 (C16) ppm; **^{11}B NMR** (128 MHz, $CDCl_3$) δ = 34.01 ppm; **IR** (ATR): ῦ = 3075, 3056, 2979, 2958, 2926, 2385, 2351, 2344, 2328, 2230, 1980, 1680, 1629, 1597, 1481, 1467, 1429, 1411, 1388, 1351, 1328, 1310, 1277, 1250, 1212, 1183, 1164, 1141, 1125, 1113, 1065, 1026, 1001, 982, 972, 938, 895, 878, 832, 819, 760, 737, 715, 702, 672, 665 cm^{-1}; **HRMS** (EI) calcd. for $C_{27}H_{33}BO_3Si$ $[M]^+$ 444.2292, found 444.2262.

(1-(Dimethyl(phenyl)silyl)-3-oxo-3-(p-tolyl)propyl)boronic acid, pinacol ester (13)

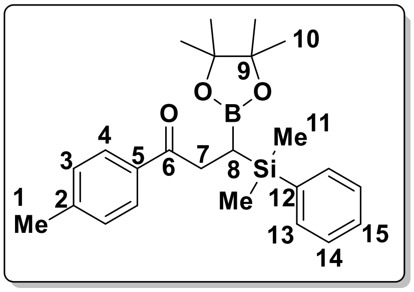

Prepared according to General Procedure **D**, **1a** (80.4 mg, 0.2 mmol), and **S13** (149 mg, 0.6 mmol) were converted to **13** yielding a yellow oil (89% NMR yield, 64.1 mg, 78%), after purification by flash column chromatography (C18, 20→100% MeCN/H_2O).

R$_f$ (10% EtOAc/*n*-hexane) = 0.33; **^{1}H NMR** (400 MHz, $CDCl_3$) δ = 7.81 – 7.74 (m, 2H, H4), 7.61 – 7.55 (m, 2H, H14), 7.37 (m, 3H, H13 and H15), 7.19 (d, *J* = 7.9 Hz, 2H, H3), 3.17 (dd, *J* = 18.3, 12.2 Hz, 1H, H7), 2.95 (dd, *J* = 18.3, 3.6 Hz, 1H, H7), 2.38 (s, 3H, H1), 1.25 (s, 6H, H10), 1.17 (s, 6H, H10), 1.08 (dd, *J* = 12.2, 3.6 Hz, 1H, H8), 0.41 (s, 3H, H11), 0.39 (s, 3H, H11) ppm; **^{13}C NMR** (101 MHz, $CDCl_3$) δ = 200.0 (C6), 143.3 (C5), 138.5 (C12), 134.6 (C2), 133.9 (C14), 129.1 (C3 and C15), 128.2 (C4), 127.9 (C13), 83.0 (C9), 36.1 (C7), 25.1 (C10), 24.8 (C10), 21.7 (C1), -2.0 (C11), -3.5 (C11) ppm; **^{11}B NMR** (128 MHz, $CDCl_3$) δ = 33.67 ppm; **IR** (ATR): ῦ = 3069, 2981, 2928, 1740, 1677, 1609, 1576, 1483, 1461, 1428, 1409, 1380, 1372, 1350, 1310, 1251, 1219, 1210, 1178, 1165, 1142, 1113, 1067, 1026, 969, 914, 880, 853, 838, 808, 765, 742, 722, 702, 674 cm^{-1}; **HRMS** (EI) cacld. for $C_{24}H_{33}BO_3Si$ $[M]^+$ 408.2292, found 408.2281.

(3-(4-Cyanophenyl)-1-(dimethyl(phenyl)silyl)-3-oxopropyl)boronic acid, pinacol ester (14)

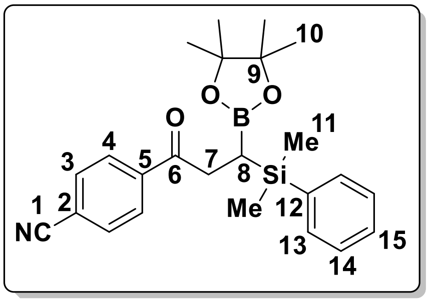

Prepared according to General Procedure **D**, **1a** (80.4 mg, 0.2 mmol), and **S10** (156 mg, 0.6 mmol) were converted to **14** yielding a white solid (66% NMR yield, 35.9 mg, 43%), after purification by flash column chromatography (SiO_2, 0→2% EtOAc/*n*-hexane; C18, 20→100% MeCN/H_2O).

R$_f$ (10% EtOAc/*n*-hexane) = 0.32; **^{1}H NMR** (400 MHz, $CDCl_3$) δ = 7.92 (d, *J* = 8.3 Hz, 2H, H4), 7.69 (d, *J* = 8.3 Hz, 2H, H3), 7.57 – 7.52 (m, 2H, H14), 7.39 – 7.34 (m, 3H, H13 and H15), 3.18 (dd, *J* = 18.5, 12.1 Hz, 1H, H7), 2.89 (dd, *J* = 18.5, 3.4 Hz, 1H, H7), 1.23 (s, 6H, H10), 1.15 (s, 6H, H10), 1.09 (dd, *J* = 12.1, 3.4 Hz, 1H, H8), 0.40 (s, 3H, H11), 0.39 (s, 3H, H11) ppm; **^{13}C NMR** (101 MHz, $CDCl_3$) δ = 199.3 (C6), 140.1 (C5), 138.0 (C12), 133.9 (C14), 132.4 (C3), 129.3 (C15), 128.5 (C4), 128.0 (C13), 118.2 (C1), 116.0 (C2), 83.2 (C9), 36.6 (C7), 25.1 (C10), 24.8 (C10), -2.1 (C11), -3.6 (C11) ppm; **^{11}B NMR** (128 MHz, $CDCl_3$) δ = 34.06 ppm; **IR** (ATR): ῦ = 3074, 3057, 2978, 2386, 2344, 2298, 2230, 1947, 1737, 1688, 1608, 1568, 1481, 1427, 1409, 1383, 1374, 1353, 1313, 1307, 1248, 1215, 1179, 1166, 1142, 1114, 1071, 1033, 1000, 983, 971, 938, 880, 856, 826, 812, 785, 762, 735, 724, 711, 697, 673, 664 cm^{-1}; **HRMS** (EI) calcd. for $C_{24}H_{30}BNO_3Si$ $[M]^+$ 419.2088, found 419.2103.

(1-(Dimethyl(phenyl)silyl)-3-oxo-3-(o-tolyl)propyl)boronic acid, pinacol ester (15)

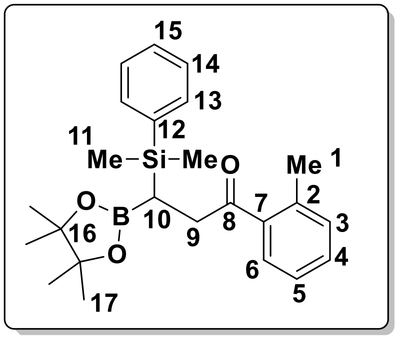

Prepared according to General Procedure **D**, **1a** (80.4 mg, 0.2 mmol) and **S18** (149 mg, 0.6 mmol) were converted to **15** yielding a white solid (86% NMR yield, 65.3 mg, 80%) after purification by flash column chromatography (SiO_2, EtOAc/*n*-hexane 0→20%).

***R**_f* (EtOAc/*n*-hexane 10%) = 0.2; **^{1}H NMR** (400 MHz, $CDCl_3$): δ = 7.60 – 7.51 (m, 2H, H13), 7.47 (dd, *J* = 8.0, 1.5 Hz, 1H, H6), 7.38 – 7.34 (m, 3H, H14 and H15), 7.30 (td, *J* = 7.5, 1.4 Hz, 1H, H3), 7.22 – 7.13 (m, 2H, H4 and H5), 3.08 (dd, *J* = 18.6, 12.4 Hz, 1H, H9), 2.83 (dd, *J* = 18.6, 3.4 Hz, 1H, H9), 2.41 (s, 3H, H1), 1.25 (s, 6H, H17), 1.18 (s, 6H, H17), 1.07 (dd, *J* = 12.3, 3.4 Hz, 1H, H10), 0.38 (m, 6H, H11) ppm; **^{13}C NMR** (101 MHz, $CDCl_3$) δ = 205.0 (C8), 138.7 (C7), 138.4 (C12), 137.4 (C2), 134.0 (C13), 131.6 (C4), 130.8 (C3), 129.1 (C15), 128.1 (C6), 127.9 (C14), 125.6 (C5), 83.1 (C16), 39.4 (C9), 25.1 (C17), 25.0 (C17), 21.0 (C1), -2.1 (C11), -3.5 (C11) ppm; **^{11}B NMR** (192.5 MHz, $CDCl_3$): δ = 34.55 ppm; **IR** (ATR) $\tilde{\nu}$ = 3070, 2977, 2931, 1687, 1601, 1572, 1483, 1456, 1428, 1404, 1380, 1372, 1352, 1329, 1305, 1249, 1211, 1141, 1112, 1024, 965, 879, 850, 839, 815, 762, 735, 721, 701 cm^{-1}; **HRMS** (EI) calcd. for $C_{24}H_{33}BO_3Si$ $[M]^+$ 408.2292, found 408.2274.

(1-(Dimethyl(phenyl)silyl)-3-(4-(morpholinomethyl)phenyl)-3-oxopropyl)boronic acid, pinacol ester (16)

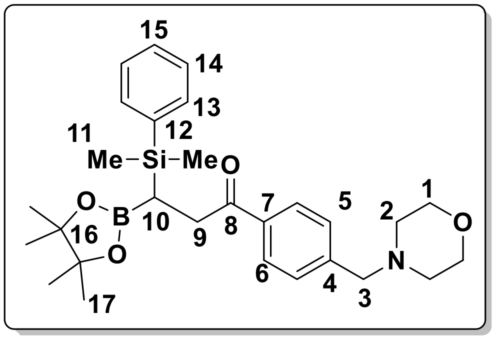

Prepared according to General Procedure **D**, **1a** (80.4 mg, 0.2 mmol) and **S16** (200 mg, 0.6 mmol) were converted to **16** yielding a colourless oil (89% NMR yield, 76.9 mg, 78%) after purification by flash column chromatography (C18, MeCN/H_2O 20→100%).

***R**_f* (EtOAc) = 0.65; **^{1}H NMR** (400 MHz, $CDCl_3$): δ = 7.81 (d, *J* = 8.3 Hz, 2H, H6), 7.59 – 7.51 (m, 2H, H13), 7.40 – 7.31 (m, 5H, H5, H14 and H15), 3.73 – 3.67 (m, 4H, H1), 3.51 (s, 2H, H3), 3.17 (dd, *J* = 18.3, 12.2 Hz, 1H, H9), 2.94 (dd, *J* = 18.3, 3.5 Hz, 1H, H9), 2.48 – 2.35 (m, 4H, H2), 1.24 (s, 6H, H17), 1.16 (s, 6H, H17), 1.07 (dd, *J* = 12.2, 3.4 Hz, 1H, H10), 0.40 (s, 3H, H11), 0.38 (s, 3H, H11) ppm; **^{13}C NMR** (101 MHz, $CDCl_3$) δ = 200.0 (C8), 143.0 (C4), 138.4 (C12), 136.2 (C7), 133.9 (C13), 129.1 (C15), 129.1 (C14), 128.1 (C6), 128.0 (C5), 83.0 (C16), 67.0 (C1), 63.1 (C3), 53.7 (C2), 36.2 (C9), 25.1 (C17), 24.8 (C17), -2.0 (C11), -3.5 (C11) ppm; **^{11}B NMR** (192.5 MHz, $CDCl_3$): δ = 34.32 ppm; **IR** (ATR) $\tilde{\nu}$ = 2977, 2857, 2808, 2247, 1686, 1609, 1574, 1456, 1428, 1414, 1380, 1372, 1351, 1308, 1250, 1216, 1142, 1116, 1071, 1035, 1009, 970, 914, 867, 835, 816, 768, 731, 701, 664 cm^{-1}; **HRMS** (ESI) calcd. for $C_{28}H_{41}BNO_4Si^+$ $[M+H]^+$ 494.2892, found 494.2904.

(1-(Dimethyl(phenyl)silyl)-3-(2-methoxyphenyl)-3-oxopropyl)boronic acid, pinacol ester (17)

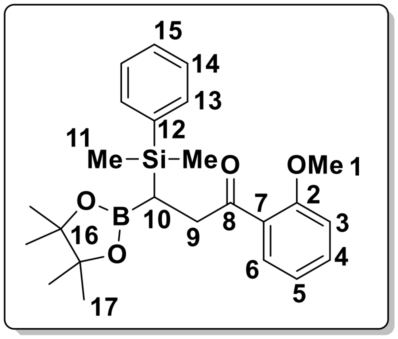

Prepared according to General Procedure **D**, **1a** (80.4 mg, 0.2 mmol) and **S17** (159 mg, 0.6 mmol) were converted to **17** yielding a white solid (82% NMR yield, 62.8 mg, 74%) containing a 16% protodeboronated impurity after purification by flash column chromatography (SiO_2, EtOAc/*n*-hexane 0→8%).

R_f (EtOAc/*n*-hexane 10%) = 0.12 ; **^{1}H NMR** (400 MHz, $CDCl_3$): δ = 7.68 (dd, *J* = 7.6, 1.9 Hz, 1H, H6), 7.63 – 7.61 (m, 2H, H13), 7.47 – 7.42 (m, 1H, H4), 7.42 – 7.39 (m, 3H, H14 and H15), 6.99 (td, *J* = 7.5, 1.0 Hz, 1H, H5), 6.92 (d, *J* = 8.4, 1.0 Hz, 1H, H3), 3.79 (s, 3H, H1), 3.21 – 3.05 (m, 2H, H9), 1.30 (s, 6H, H17), 1.23 (s, 6H, H17), 1.08 (dd, *J* = 11.7, 4.3 Hz, 1H, H10), 0.44 (m, 6H, H11) ppm; **^{13}C NMR** (101 MHz, $CDCl_3$) δ = 202.7 (C8), 158.5 (C7), 138.8 (C12), 134.0 (C13), 133.7 (C2) 133.0 (C4), 130.2 (C6), 129.0 (C15), 127.7 (C14), 120.5 (C5), 111.5 (C3), 82.9 (C16), 55.4 (C1), 41.5 (C9), 25.1 (C17), 24.8 (C17), -2.3 (C11), -3.4 (C11) ppm; **^{11}B NMR** (192.5 MHz, $CDCl_3$): δ = 34.19 ppm; **IR** (ATR) $\tilde{\nu}$ = 3072, 2978, 2841, 2248, 1673, 1598, 1485, 1466, 1438, 1429, 1379, 1372, 1351, 1305, 1284, 1243, 1198, 1181, 1163, 1143, 1112, 1053, 1025, 983, 969, 909, 877, 851, 838, 814, 756, 731, 700, 662 cm^{-1}; **HRMS** (EI) calcd. for $C_{24}H_{33}BO_4Si$ $[M]^+$ 424.2241, found 424.2257.

(3-(4-Bromophenyl)-3-oxopropane-1,1-diyl)diboronic acid, pinacol ester (18)

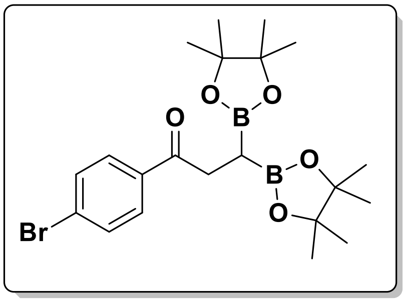

Prepared according to General Procedure **D**, **1c** (78.8 mg, 0.2 mmol), and **S12** (188 mg, 0.6 mmol) were converted to **18** yielding a white solid (52% NMR yield, 44.6 mg, 48%), after purification by flash column chromatography (SiO_2, 0→7.5% EtOAc/*n*-hexane).

^{1}H NMR (400 MHz, $CDCl_3$) δ = 7.85 (d, *J* = 8.3 Hz, 2H), 7.56 (d, *J* = 8.2 Hz, 2H), 3.24 (d, *J* = 7.9 Hz, 2H), 1.25 (s, 12H), 1.21 (s, 12H), 1.12 (t, *J* = 7.9 Hz, 1H) ppm; analytical data in agreement with literature.[24]

(3-Oxo-3-(thiophen-2-yl)propane-1,1-diyl)diboronic acid, pinacol ester (19)

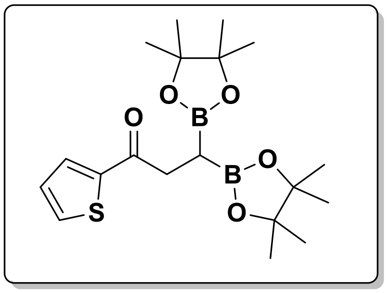

Prepared according to General Procedure **D**, **1c** (78.8 mg, 0.2 mmol), and **S21** (144 mg, 0.6 mmol) were converted to **19** yielding a pale yellow oil (85% NMR yield, 50.1 mg, 64%), after purification by flash column chromatography (C18, 20→100% MeCN/H_2O).

^{1}H NMR (400 MHz, $CDCl_3$) δ = 7.75 (dd, *J* = 3.8, 1.2 Hz, 1H), 7.56 (dd, *J* = 5.0, 1.2 Hz, 1H), 7.08 (dd, *J* = 5.0, 3.8 Hz, 1H), 3.22 (d, *J* = 8.0 Hz, 2H), 1.24 (s, 12H), 1.21 (s, 12H), 1.13 (t, *J* = 8.0 Hz, 1H) ppm; analytical data in agreement with literature.[24]

(3-(4-Acetamidophenyl)-3-oxopropane-1,1-diyl)diboronic acid, pinacol ester (20)

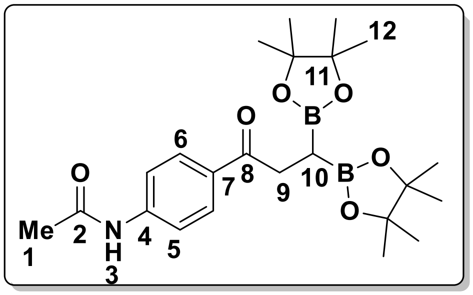

Prepared according to General Procedure **D**, **1c** (78.8 mg, 0.2 mmol) and **S19** (175 mg, 0.6 mmol) were converted to **20** yielding a white solid (74% NMR yield, 49.6 mg, 56%) after purification by flash column chromatography (SiO_2, EtOAc/*n*-hexane 0→30%).

R$_f$ (EtOAc/*n*-hexane 20%) = 0.10; **^{1}H NMR** (600 MHz, $CDCl_3$): δ = 8.16 (s, 1H, H3), 7.90 (d, *J* = 8.7 Hz, 2H, H6), 7.59 (d, *J* = 8.7 Hz, 2H, H5), 3.23 (d, *J* = 8.0 Hz, 2H, H9), 2.19 (s, 3H, H1), 1.23 (s, 12H, H12), 1.20 (s, 12H, H12), 1.08 (t, *J* = 8.0 Hz, 1H, H10) ppm; **^{13}C NMR** (150.9 MHz, $CDCl_3$): δ = 199.6 (C8), 169.0 (C2), 142.3 (C7), 132.5 (C4), 129.5 (C6), 118.9 (C5), 83.3 (C11), 35.9 (C9), 24.9 (C12), 24.9 (C1), 24.7 (C12) ppm; **^{11}B NMR** (192.5 MHz, $CDCl_3$): δ = 34.98 ppm; **IR** (ATR) $\tilde{\nu}$ = 3315, 3275, 3203, 3130, 2979, 2934, 1684, 1675, 1602, 1533, 1482, 1468, 1408, 1389, 1368, 1343, 1305, 1267, 1212, 1173, 1138, 1111, 1079, 1044, 1015, 989, 973, 936, 903, 874, 854, 832, 822, 738, 689, 672 cm^{-1}; **HRMS** (ESI) calcd. for $C_{23}H_{36}B_2NO_6^+$ $[M+H]^+$ 444.2723, found 444.2711.

(3-(4-Bromophenyl)-3-oxo-1-(triethylgermyl)propyl)boronic acid, pinacol ester (21)

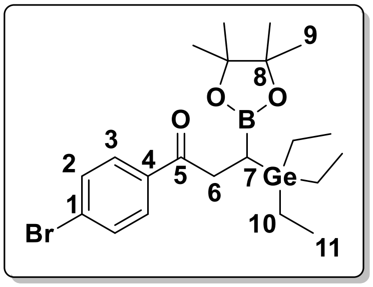

Prepared according to General Procedure **D**, **1b** (85.3 mg, 0.2 mmol), and **S12** (0.188 g, 0.6 mmol) were converted to **21** yielding a white solid (37.9 mg, 38%), after purification by flash column chromatography (SiO_2, 0→1% EtOAc/*n*-hexane; C18, 20→100% MeCN/H_2O).

R$_f$ (10% EtOAc/*n*-hexane) = 0.58; **^{1}H NMR** (400 MHz, $CDCl_3$) δ 7.82 – 7.79 (m, 2H, H3), 7.59 – 7.56 (m, 2H, H2), 3.22 (dd, *J* = 18.1, 12.2 Hz, 1H, H6), 2.98 (dd, *J* = 18.1, 3.8 Hz, 1H, H6), 1.23 (s, 6H, H9), 1.17 (s, 6H, H9), 1.07 (t, *J* = 7.9 Hz, 9H, H11), 1.03 (dd, *J* = 12.2, 3.8 Hz, 1H, H7), 0.85 (qd, *J* = 7.9, 3.1 Hz, 6H, H10) ppm; **^{13}C NMR** (101 MHz, $CDCl_3$) δ 200.0 (C5), 136.2 (C4), 131.8 (C2), 129.7 (C3), 127.7 (C1), 82.9 (C8), 36.6 (C6), 25.2 (C9), 24.9 (C9), 9.2 (C11), 4.3 (C10) ppm; **^{11}B NMR** (128 MHz, $CDCl_3$) δ = 34.11 ppm; **IR** (ATR): ῦ = 3099, 2958, 2927, 2907, 2874, 2197, 1966, 1684, 1585, 1572, 1482, 1462, 1426, 1389, 1358, 1331, 1299, 1254, 1209, 1178, 1144, 1111, 1069, 1021, 1008, 970, 878, 845, 816, 757, 731, 707, 671 cm^{-1}; **HMRS** (ESI) calcd. for $C_{21}H_{34}BBrGeO_3Na^+$ $[M+Na]^+$ 521.0888, found 521.0912.

(1-(Dimethyl(phenyl)silyl)-4-methyl-3-methylenepent-4-en-1-yl)boronic acid, pinacol ester (22)

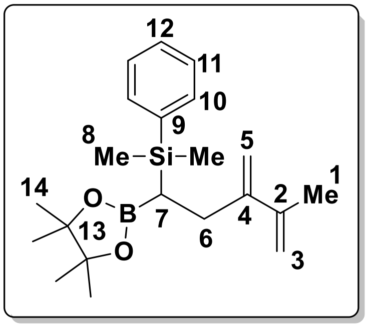

Prepared according to General Procedure **E**, **1a** (80.4 mg, 0.2 mmol) and 2,3-dimethyl-1,3-butadiene (68 µL, 0.6 mmol) were converted to **22** yielding a colourless oil (63% NMR yield, 37.1 mg, 52%) after purification by flash column chromatography (SiO_2, EtOAc/*n*-hexane 0→5%).

R$_f$ (5% EtOAc/*n*-hexane) = 0.25; **^{1}H NMR** (400 MHz, $CDCl_3$): δ = 7.58 – 7.53 (m, 2H), 7.37 – 7.32 (m, 3H), 5.00 – 4.98 (m, 2H, H5), 4.87 – 4.82 (m, 2H, H3), 2.44 (dd, *J* = 14.9, 12.2, 1.0 Hz, 1H, H6), 2.32 (dd, *J* = 14.9, 3.0, 1.2 Hz, 1H, H6), 1.85 – 1.80 (m, 3H, H1), 1.17 (s, 6H, H14), 1.12 (s, 6H, H14), 0.95 (dd, *J* = 12.2, 3.0 Hz, 1H, H7), 0.37 (s, 3H, H8), 0.35 (s, 3H, H8) ppm; **^{13}C NMR** (101 MHz, $CDCl_3$) δ = 150.0 (C4), 142.6 (C2), 138.7 (C9), 134.0 (ArCH), 129.0 (ArCH), 127.8 (ArCH), 112.6

(C3), 111.2 (C5), 82.88 (C13), 29.5 (C6), 25.1 (C14), 25.0 (C14), 21.5 (C1), -2.4 (C8), -3.4 (C8) ppm; **^{11}B NMR** (192.5 MHz, $CDCl_3$): δ = 34.32 ppm; **IR** (ATR) $\tilde{\nu}$ = 2980, 2840, 1597, 1480, 1460, 1428, 1379, 1372, 1343, 1318, 1300, 1248, 1206, 1195, 1151, 1113, 1069, 993, 972, 944, 893, 873, 841, 818, 769, 734, 701, 681, 660 cm^{-1}; **HRMS** (EI) calcd. for $C_{21}H_{33}BO_2Si$ $[M]^+$ 356.2343, found 356.2297.

(4-Methyl-3-methylenepent-4-ene-1,1-diyl)diboronic acid, pinacol ester (23)

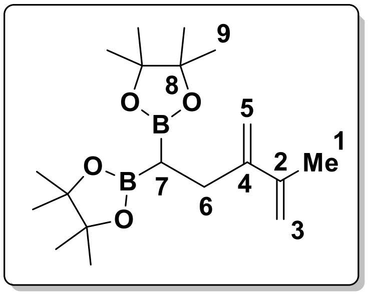

Prepared according to General Procedure **E**, **1c** (78.8 mg, 0.2 mmol) and 2,3-dimethyl-1,3-butadiene (68 µL, 0.6 mmol) were converted to **23** yielding a colourless oil (49% NMR yield, 29.2 mg, 42%) after purification by flash column chromatography (SiO_2, EtOAc/*n*-hexane 0→12%).

R$_f$ (5% EtOAc/*n*-hexane) = 0.06; **^{1}H NMR** (400 MHz, $CDCl_3$): δ = 5.09 (s, 1H, H3), 5.01 (s, 1H, H5), 4.99 (s, 1H, H5), 4.92 (s, 1H, H3), 2.52 (d, *J* = 7.8 Hz, 2H, H6), 1.87 (s, 3H, H1), 1.21 (s, 12H, H9), 1.20 (s, 12H, H9), 1.03 (t, *J* = 8.0 Hz, 1H, H7) ppm; **^{13}C NMR** (101 MHz, $CDCl_3$): δ = 149.7 (C2), 142.9 (C4), 112.7 (C3), 111.0 (C5), 83.1 (C8), 29.0 (C6), 24.9 (C9), 24.7 (C9), 21.5 (C1) ppm; **^{11}B NMR** (192.5 MHz, $CDCl_3$): δ = 33.49 ppm; **IR** (ATR) $\tilde{\nu}$ = 3095, 2980, 2931, 1560, 1469, 1379, 1371, 1356, 1317, 1271, 1248, 1216, 1166, 1140, 1111, 1084, 1006, 972, 890, 869, 851, 736, 670 cm^{-1}; **HRMS** (ESI) calcd. for $C_{19}H_{35}B_2O_4^+$ $[M+H]^+$ 349.2716, found 349.2711.

(2-(1,3-Dimethyl-2-oxoindolin-3-yl)ethane-1,1-diyl)diboronic acid, pinacol ester (24)

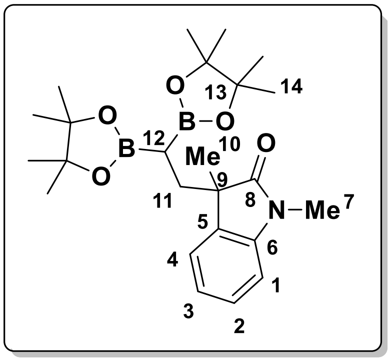

Prepared according to General Procedure **F**, **1c** (78.8 mg, 0.2 mmol) and **S22** (70.1 mg, 0.4 mmol) were converted to **24** yielding a colourless oil (85% NMR yield, 66.2 mg, 75%) after purification by flash column chromatography (SiO_2, DCM).

R$_f$ (EtOAc) = 0.78; **^{1}H NMR** (400 MHz, $CDCl_3$): δ = 7.25 – 7.19 (m, 2H), 7.05 – 6.98 (m, 1H), 6.81 – 6.76 (m, 1H), 3.18 (s, 3H, H7), 2.24 – 2.09 (m, 2H, H11), 1.33 (s, 3H, H10), 1.21 (s, 12H, H14), 1.13 (s, 6H, H14), 1.06 (s, 6H, H14), 0.38 (t, *J* = 6.2 Hz, 1H, H12) ppm; **^{13}C NMR** (101 MHz, $CDCl_3$): δ = 180.5 (C8), 143.7 (C6), 134.0 (C5), 127.6 (CH), 123.4 (CH), 122.2 (CH), 107.8 (CH), 83.2 (C13), 83.0 (C13), 49.5 (C9), 33.7 (C11), 26.1 (C7), 25.0 (C14), 24.9 (C14), 24.7 (C14), 24.7 (C14), 23.4 (C10) ppm; **^{11}B NMR** (192.5 MHz, $CDCl_3$): δ = 33.53 ppm; **IR** (ATR) $\tilde{\nu}$ = 2979, 2931, 2248, 1711, 1614, 1494, 1471, 1372, 1359, 1312, 1264, 1214, 1137, 1098, 1065, 1022, 968, 922, 868, 849, 789, 753, 731, 700, 671 cm^{-1}; **HRMS** (ESI) calcd. for $C_{24}H_{37}B_2NO_5Na^+$ $[M+Na]^+$ 464.1712, found 464.1763.

(2-(1,3-Dimethyl-2-oxoindolin-3-yl)-1-(triethylgermyl)ethyl)boronic acid, pinacol ester (25)

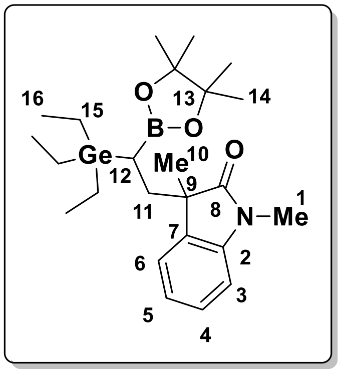

Prepared according to General Procedure **F**, **1b** (85.3 mg, 0.2 mmol), and **S22** (70.1 mg, 0.4 mmol) were converted to **25** yielding an off-white solid (45% NMR yield, 40.9 mg, 43%, 1:1.3 *dr*), after purification by flash column chromatography (SiO_2, 0→14% EtOAc/*n*-hexane).

$\mathbf{R_f}$ (10% EtOAc/*n*-hexane) = 0.29; **^{1}H NMR** (400 MHz, $CDCl_3$, *major diastereomer*) δ 7.21 (tdd, *J* = 7.7, 3.5, 1.3 Hz, 1H, H4), 7.14 (dd, *J* = 7.3, 1.3 Hz, 1H, H6), 7.01 (tdd, *J* = 7.5, 3.6, 1.0 Hz, 1H, H5), 6.78 (t, *J* = 8.4 Hz, 1H, H3), 3.17 (s, 3H, H1), 2.28 (dd, *J* = 13.6, 11.2 Hz, 1H, H11), 1.80 (d, *J* = 13.5 Hz, 1H, H11), 1.32 (s, 3H, H10), 1.20 (s, 6H, H14), 1.18 (s, 6H, H14), 0.93 (t, *J* = 7.9 Hz, 9H, H16), 0.75 – 0.69 (m, 6H, H15), 0.28 (d, *J* = 11.2 Hz, 1H, H12) ppm; **^{1}H NMR** (400 MHz, $CDCl_3$, *minor diastereomer*) δ 7.24 (d, *J* = 1.2 Hz, 1H, H6'), 7.21 (m, 1H, H4'), 7.01 (m, 1H, H5'), 6.78 (t, *J* = 8.4 Hz, 1H, H3'), 3.17 (s, 3H, H1'), 2.22 (dd, *J* = 13.5, 11.8 Hz, 1H, H11'), 1.94 – 1.89 (m, 1H, H11'), 1.30 (s, 3H, H10'), 1.13 (s, 6H, H14'), 0.99 (t, *J* = 7.9 Hz, 9H, H16'), 0.96 (s, 6H, H14'), 0.81 – 0.76 (m, 6H, H15'), 0.36 – 0.32 (m, 1H, H12') ppm; **^{13}C NMR** (101 MHz, $CDCl_3$, mixture of diastereomers) δ 180.6, 180.4, 143.9, 143.8, 134.3, 134.0, 127.7, 127.6, 124.1, 122.9, 122.3, 122.2, 107.9, 107.8, 82.8, 82.7, 50.4, 49.8, 35.8, 35.2, 26.2, 26.2, 26.1, 25.6, 25.3, 24.8, 23.6, 22.9, 9.2, 9.1, 4.2, 4.0 ppm; **^{11}B NMR** (128 MHz, $CDCl_3$) δ = 33.65 ppm; **IR** (ATR): ῦ = 3057, 2930, 2874, 1711, 1615, 1494, 1471, 1454, 1377, 1352, 1336, 1307, 1281, 1267, 1256, 1206, 1165, 1143, 1125, 1097, 1057, 1024, 996, 974, 931, 872, 843, 790, 765, 751, 740, 729, 716, 699 cm^{-1}; **HMRS** (ESI) calcd. for $C_{24}H_{40}BGeNO_3Na^+$ $[M+Na]^+$ 498.2205, found 498.2222.

(3-(4-(3-(4-(6-Fluorobenzo[d]isoxazol-3-yl)piperidin-1-yl)propoxy)-3-methoxyphenyl)-3-oxopropane-1,1-diyl)diboronic acid, pinacol ester (26)

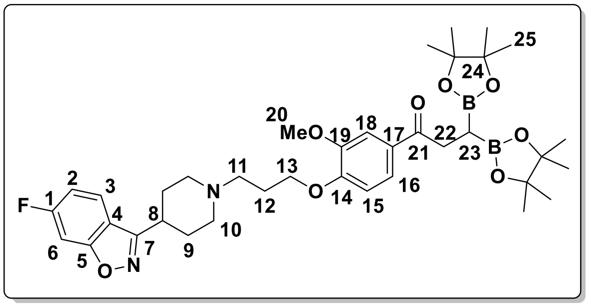

Prepared according to General Procedure **D**, **1c** (78.8 mg, 0.2 mmol) and **S20** (216 mg, 0.4 mmol) were converted to **26** yielding a yellow solid (47.1 mg, 34 %) after purification by flash column chromatography (C18, 20→100%, MeCN/H_2O).

$\boldsymbol{R_f}$ (EtOAc) = 0.28; **^{1}H NMR** (600 MHz, $CDCl_3$) δ = 7.69 (dt, *J* = 8.0, 4.0 Hz, 1H, H3), 7.62 (dd, *J* = 8.4, 2.0 Hz, 1H, H16), 7.52 (d, *J* = 2.1 Hz, 1H, H18), 7.23 (dd, *J* = 8.5, 2.2 Hz, 1H, H6), 7.05 (td, *J* = 8.8, 2.2 Hz, 1H, H2), 6.89 (d, *J* = 8.4 Hz, 1H, H15), 4.17 (t, *J* = 6.6 Hz, 2H, H13), 3.90 (s, 3H, H20), 3.24 (d, *J* = 7.9 Hz, 2H, H22), 3.11 – 3.08 (m, 3H, H8, H10), 2.60 (t, *J* = 7.3 Hz, 2H, H11), 2.22 – 2.16 (m, 2H, H10), 2.14 – 2.02 (m, 6H, H9, H12), 1.25 (s, 12H, H25), 1.22 (s, 12H, H25), 1.10 (t, *J* = 7.9 Hz, 1H, H23) ppm; **^{13}C NMR** (150.9 MHz, $CDCl_3$) : δ = 199.3 (C21), 164.2 (d, J_{CF} = 250.3 Hz, C1), 164.0 (d, J_{CF} = 13.7 Hz, C5), 161.2 (C7), 152.5 (C14), 149.2 (C19), 130.3 (C17), 122.8 (C16), 122.7 (d, J_{CF} = 10.2 Hz, C3), 117.4 (C4), 112.5 (d, J_{CF} = 25.6 Hz, C2), 111.4 (C15), 110.9 (C18), 97.6 (d, J_{CF} = 26.7 Hz, C6), 83.2 (C24), 67.4 (C13), 56.2 (C20), 55.3 (C11), 53.6 (C10), 35.6 (C22), 34.7 (C8), 30.6 (C9), 26.7 (C12), 25.0 (C25), 24.7 (C25) ppm; **^{11}B NMR** (192.5 MHz, $CDCl_3$) : δ = 34.76 ppm; **^{19}F NMR** (564.7 MHz, $CDCl_3$) : δ = 109.6 ppm; **IR** (ATR) $\tilde{\nu}$ = 2979, 2933, 2250, 1675, 1616, 1596, 1514, 1497, 1467, 1417, 1390, 1372, 1312, 1266, 1216, 1199, 1167, 1138, 1035, 1020, 970, 957, 910, 851, 840, 814, 776, 731, 670 cm^{-1}; **HRMS** (ESI) calcd. for $C_{37}H_{51}B_2FN_2O_8Na^+$ $[M+Na]^+$ 715.3708, found 715.3747.

Additional Substrate

(4-Methyl-3-methylene-1-(triethylgermyl)pent-4-en-1-yl)boronic acid, pinacol ester (S23)

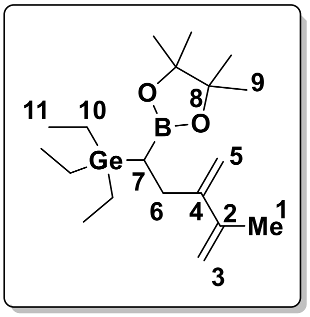

Prepared according to General Procedure **E**, **1b** (85.3 mg, 0.2 mmol), and 2,3-dimethyl-1,3-butadiene (68 μL, 0.6 mmol) were converted to **S23** yielding a colourless oil (50% NMR yield, 24.4 mg, 32%), after purification by flash column chromatography (SiO_2, 0→1% Et_2O/*n*-pentane).

$\mathbf{R_f}$ (10% EtOAc/*n*-hexane) = 0.71; ^{1}**H NMR** (400 MHz, $CDCl_3$) δ 5.05 (s, 1H, H3), 5.03 (s, 2H, H5), 4.93 (s, 1H, H3), 2.51 (dd, *J* = 15.2, 12.5 Hz, 1H, H6), 2.38 (dd, *J* = 15.2, 3.2 Hz, 1H, H6), 1.88 (s, 3H, H1), 1.20 (s, 6H, H9), 1.18 (s, 6H, H9), 1.06 (t, *J* = 7.9 Hz, 9H, H11), 0.91 (dd, *J* = 12.6, 3.2 Hz, 1H, H7), 0.86 – 0.80 (m, 6H, H10) ppm; ^{13}**C NMR** (101 MHz, $CDCl_3$) δ 150.4 (C4), 143.1 (C2), 112.4 (C3), 111.1 (C5), 82.7 (C8), 30.2 (C6), 25.3 (C9), 25.0 (C9), 21.6 (C1), 9.2 (C11), 4.3 (C10) ppm; ^{11}**B NMR** (128 MHz, $CDCl_3$) δ = 34.59 ppm; **IR** (ATR): ṽ = 3095, 2949, 2932, 2874, 1712, 1616, 1601, 1495, 1470, 1456, 1378, 1371, 1352, 1312, 1255, 1212, 1165, 1143, 1098, 1058, 1023, 973, 932, 889, 873, 842, 791, 766, 751, 740, 717, 699, 674 cm^{-1}; **HMRS** (EI) calcd. for $C_{19}H_{37}BGeO_2$ $[M-C_2H_5]^+$ 353.1707, found 353.1716.

Failed Substrates

Silyl Enol Ethers

All reactions were performed following General Procedure **D**.

Table S6. Failed Silyl Enol Ether Substrates

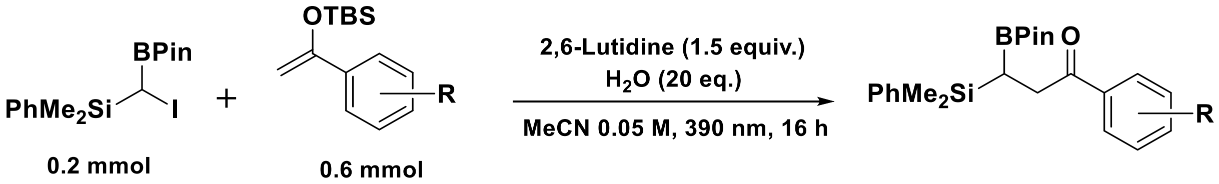

R	Yield[a] (%)	1a Retention[a] (%)
4-NO_2	0	74
2-CF_3	0	95

[a] Determined by ^{1}H NMR spectroscopy against a known internal standard (1,3,5-trimethoxybenzene).

Alkenes

All reactions were performed following General Procedure **C**.

Table S7. Failed Alkene Substrates

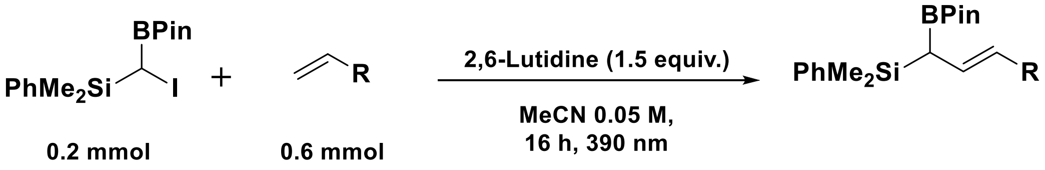

R	Yield[a] (%)	1a Retention[a] (%)
^{t}Bu	0	95

BMIDA	0	85

[a] Determined by ^{1}H NMR spectroscopy against a known internal standard (1,3,5-trimethoxybenzene).

Allylic Sulfones

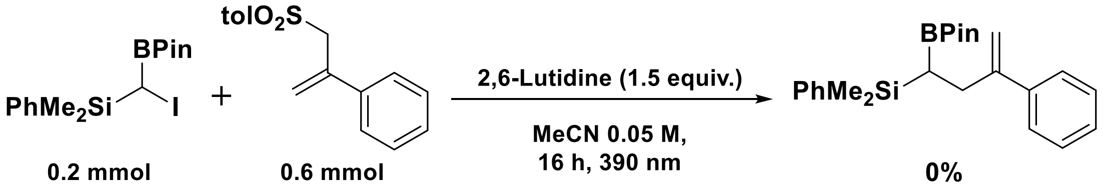

Reaction was prepared according to General Procedure **C**. After completion, internal standard (1,3,5-trimethoxybenzene), as a solution in MeCN, was added. Crude NMR analysis against the internal standard revealed no formation of product had occurred. 98% of **1a** was retained.

α-CF_3 Styrenes

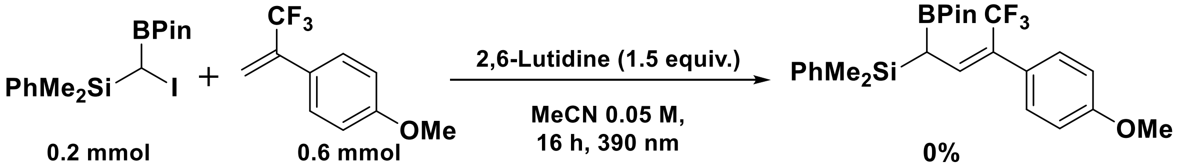

Reaction was prepared according to General Procedure **C**. After completion, internal standard (1,3,5-trimethoxybenzene), as a solution in MeCN, was added. Crude NMR analysis against the internal standard revealed no formation of product had occurred. 86% of **1a** was retained.

Heterocycles

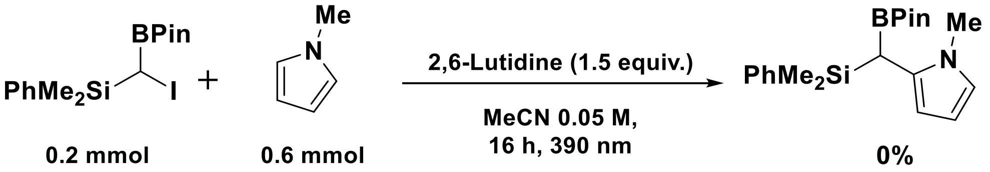

Reaction was prepared according to General Procedure **C**. After completion, internal standard (1,3,5-trimethoxybenzene), as a solution in MeCN, was added. Crude NMR analysis against the internal standard revealed no formation of product had occurred. 73% of **1a** was retained.

[1.1.1]Propellanes

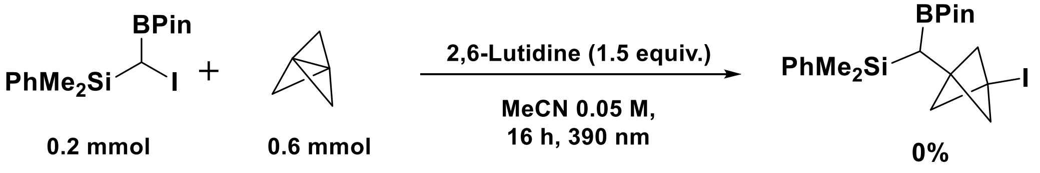

Reaction was prepared according to General Procedure **C**. After completion, internal standard (1,3,5-trimethoxybenzene), as a solution in MeCN, was added. Crude NMR analysis against the internal standard revealed no formation of product had occurred. 0% of **1a** was retained. This suggests *in situ* degradation or side-reactions are occurring under model reaction conditions.

Mechanistic Studies

Control Reactions

Control Reactions (Styrene SOMOphile)

All reactions were performed following General Procedure **C**, with the described deviations.

Table S8. Control Reactions for Photocatalyst-Free System (Styrene SOMOphile)

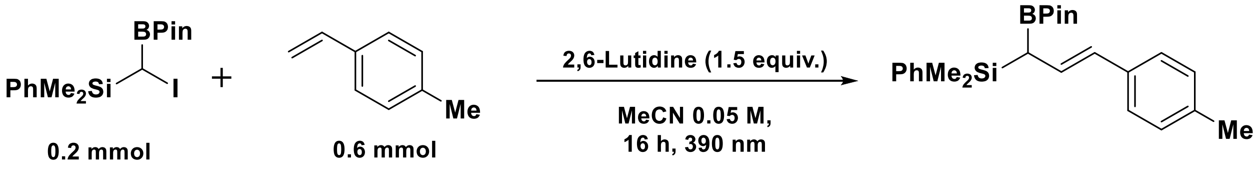

Entry	Deviation from Standard Conditions	Yield[a] (%)	1a Retention[a] (%)
24	No Light Irradiation	0	100
25	No Additive	0	90
26	Presence of O_2	0	80
27	Presence of TEMPO (1 equiv.)	0	74

[a] Determined by ^1H NMR spectroscopy against a known internal standard (1,3,5-trimethoxybenzene).

Control Reactions (Silyl Enol Ether SOMOphile)

All reactions were performed following General Procedure **D**, with the described deviations.

Table S9. Control Reactions for Photocatalyst-Free System (Silyl Enol Ether SOMOphile)

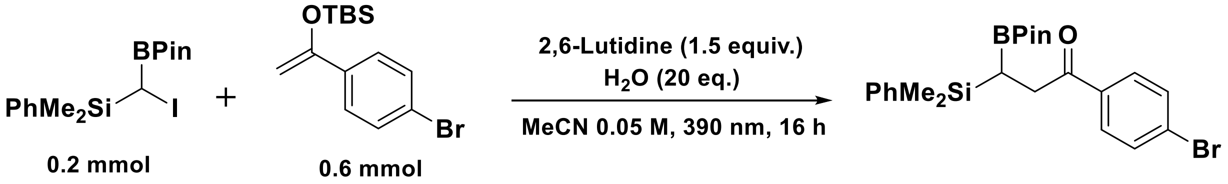

Entry	Deviation from Standard Conditions	Yield[a] (%)	1a Retention[a] (%)
28	No Additive	0	97
29	No Light + Heating to 50 °C	0	94

[a] Determined by ^1H NMR spectroscopy against a known internal standard (1,3,5-trimethoxybenzene).

Influence of Boron p-orbital

All reactions were performed following General Procedure **C**.

Table S10. Influence of Boron p-orbital

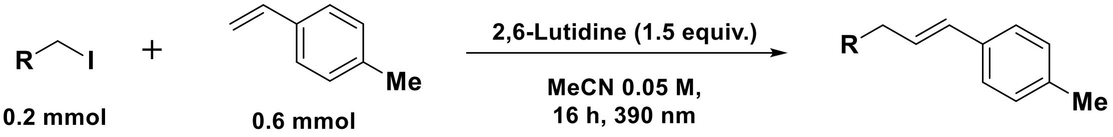

Entry	R	Yield[a] (%) (*E*:*Z*)	α-Iodo Retention[a] (%)
30	BPin	36 (>95:5)	0
31	$SiMe_2Ph$	<5	73
32	$GeEt_3$	<5	55

[a] Determined by ^1H NMR spectroscopy against a known internal standard (1,3,5-trimethoxybenzene).

Reaction with Alternative Light Source

All reactions were performed following General Procedure **C**, with the described deviation of light source.

Table S11. Reaction with Alternative Light Source

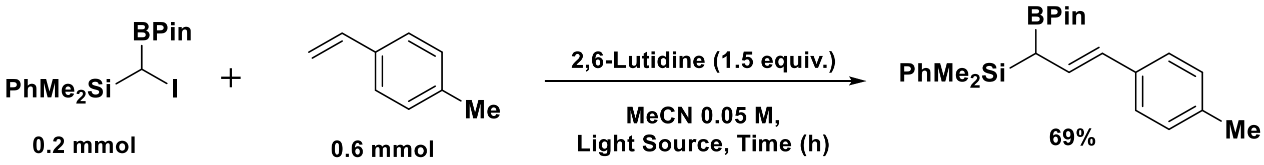

Entry	Light source (nm)	Wattage (W)	Time (h)	Yield[a] (%)	*E*:*Z* ratio[a]
**2**	390	40	16	69	89:11
33	405[b]	18	48	69	88:12

[a] Determined by ^1H NMR spectroscopy against a known internal standard (1,3,5-trimethoxybenzene). [b] Ran with EvoluChem™ LED (405 nm, 18 W, HCK1012-01-010).

Reaction with Presence of Triiodide

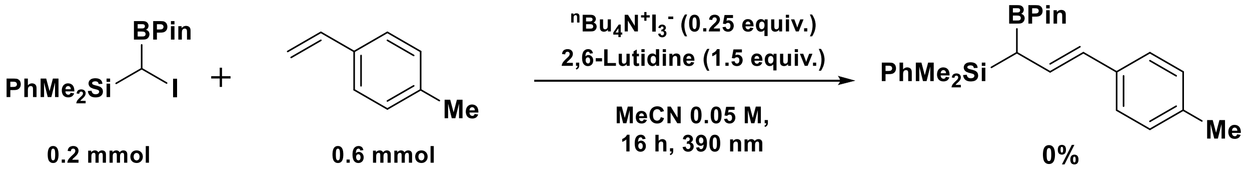

Reaction was performed prepared according to General Procedure **C**, with the described deviation. After completion, internal standard (1,3,5-trimethoxybenzene), as a solution in MeCN, was added. Crude NMR analysis against the internal standard revealed no formation of product had occurred. 98% of **1a** was retained.

UV/Vis Analysis

All absorption spectra were recorded on a Shimadzu UV-1900 I UV/Vis Spectrophotometer, at medium speed with 2 nm steps in the 330-600 nm range using a 1 cm path quartz cuvette. All concentrations are based on the concentration of model reaction conditions unless stated otherwise.

Absorption spectra of starting materials

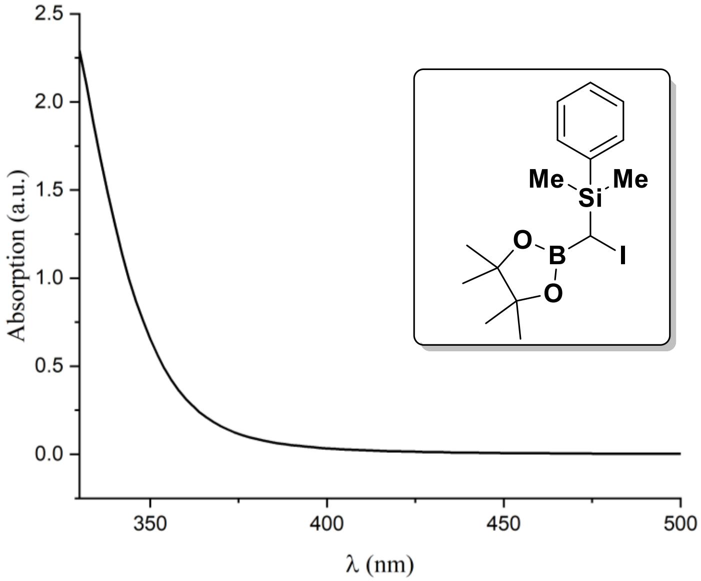

Figure S5. Absorption spectrum of **1a** in MeCN (0.05 M).

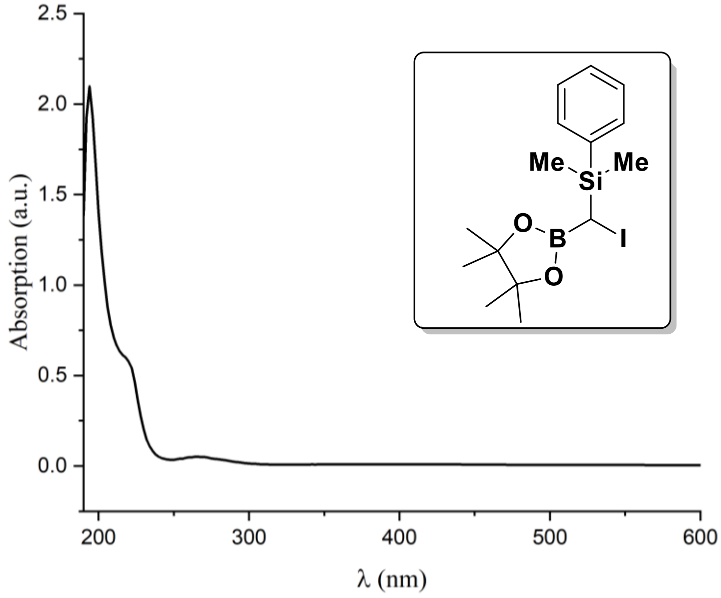

Figure S6. Absorption spectrum of **1a** in MeCN (0.5 mM).

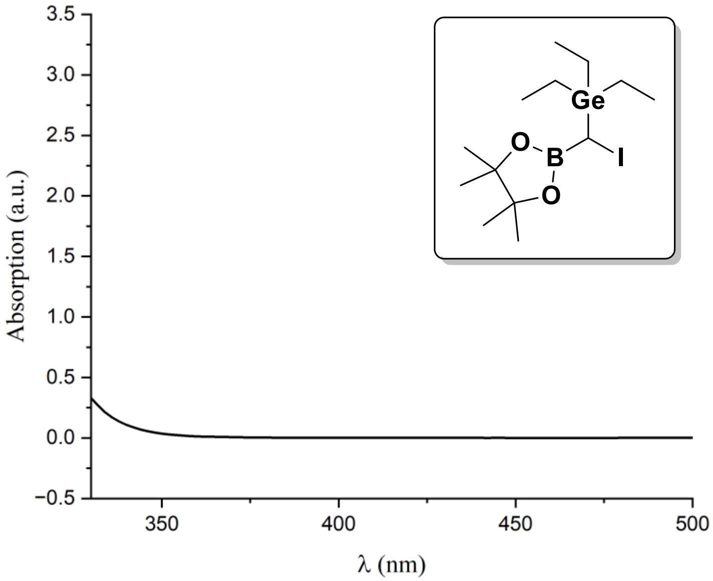

Figure S7. Absorption spectrum of **1b** in MeCN (0.05 M).

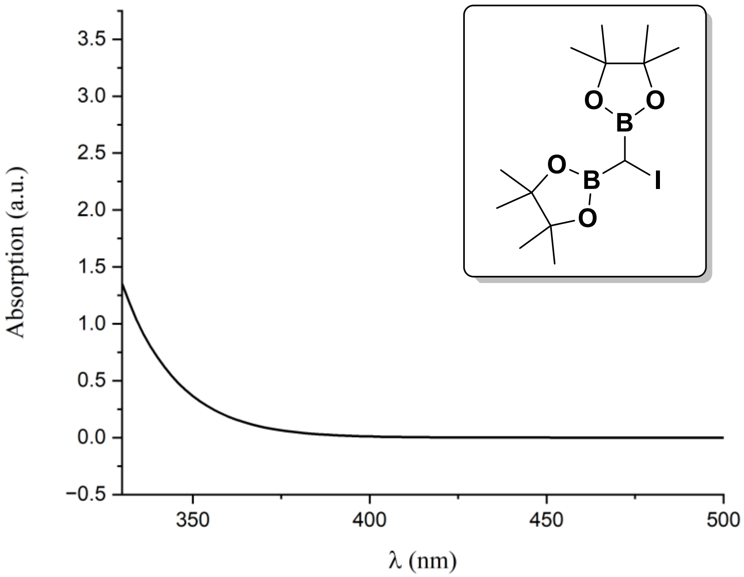

Figure S8. Absorption spectrum of **1c** in MeCN (0.05 M).

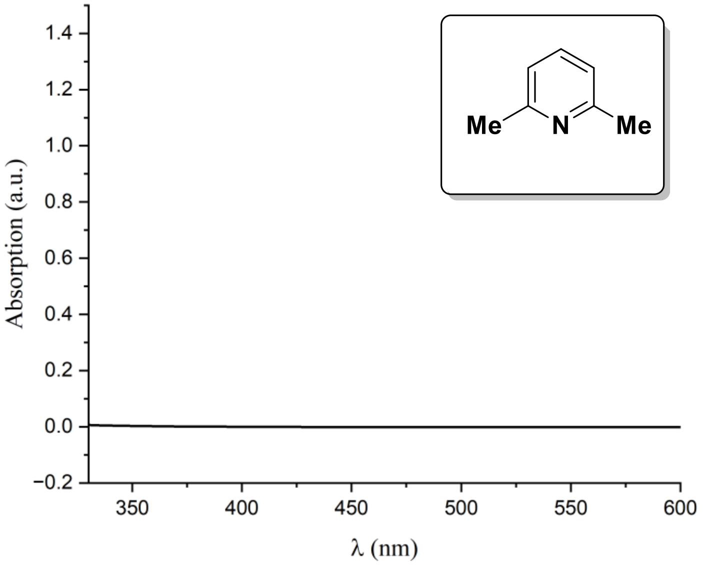

Figure S9. Absorption spectrum of 2,6-lutidine in MeCN (0.075 M).

Comment: The absorption spectra of sample **1a** at standard low UV-VIS concentration (Figure S6) showed a $\lambda_{max} \approx 194$ nm and no bands above 300 nm. An increase in concentration (Figure S5) led to a broader spectrum resulting in the tail of the broadening, moving towards the visible light region (>300 nm). The absorption of **1a**, **1b**, and **1c** at reaction concentration begins weakly at 360, 341, and 360 nm respectively. Control absorption of 2,6-lutidine showed no absorption in the visible light region.

Absorption spectra of mixtures

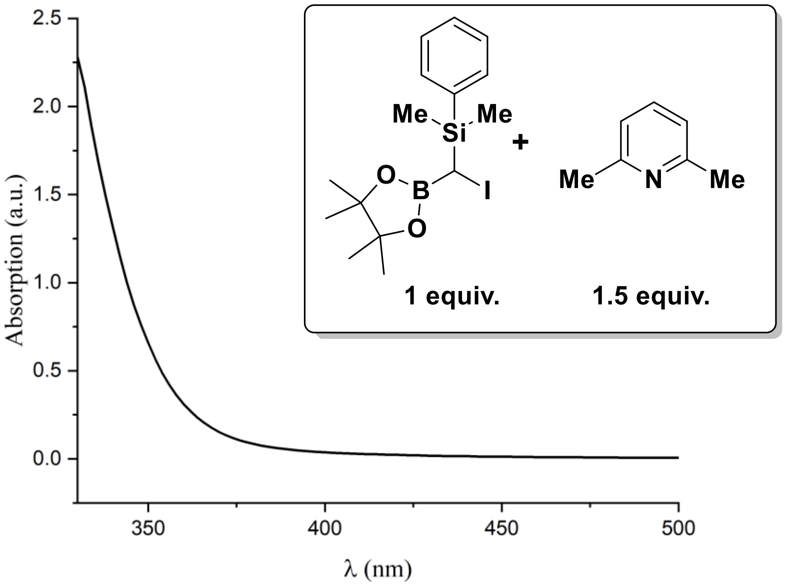

Figure S10. Absorption spectrum of **1a** (0.05 M) and 2,6-lutidine (0.075 M) in MeCN. Uv-Vis recorded after 15 min of stirring in the dark.

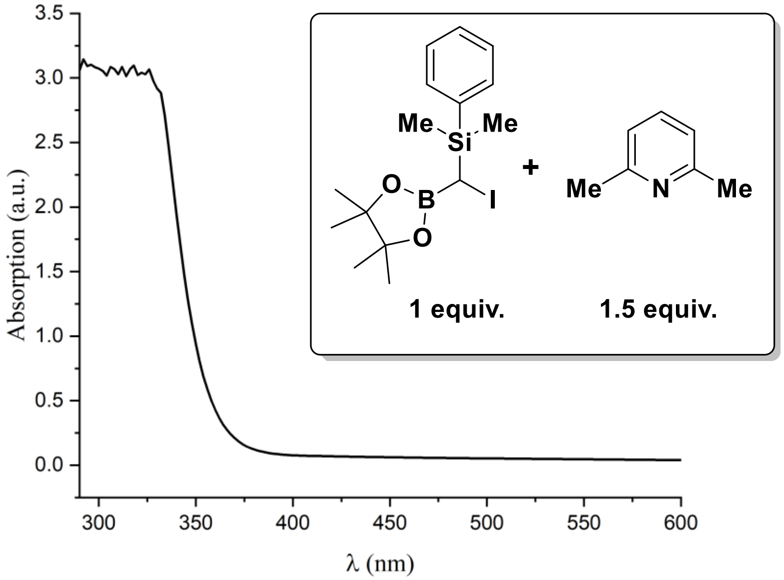

Figure S11. Absorption spectrum of **1a** (0.1 M) and 2,6-lutidine (0.15 M) in MeCN. UV-Vis recorded after 15 min of stirring in the dark.

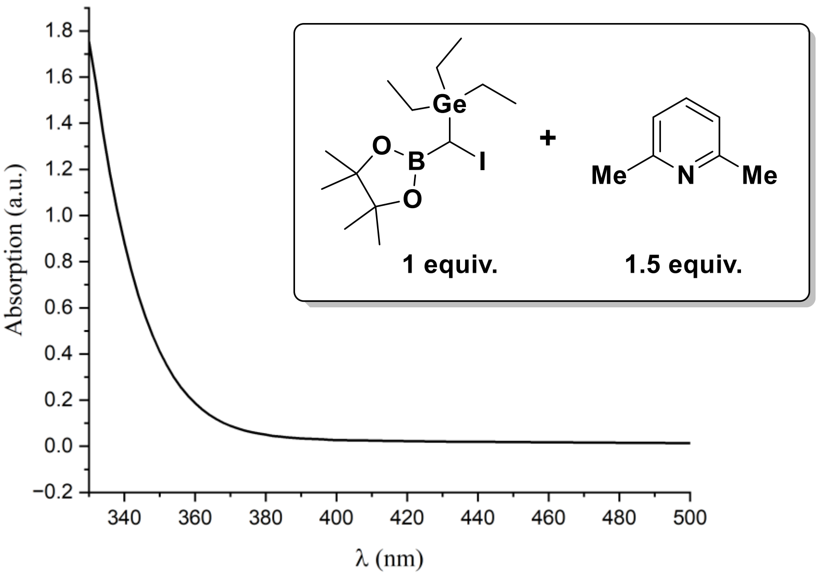

Figure S12. Absorption spectrum of **1b** (0.05 M) and 2,6-lutidine (0.075 M) in MeCN. UV-Vis recorded after 15 min of stirring in the dark.

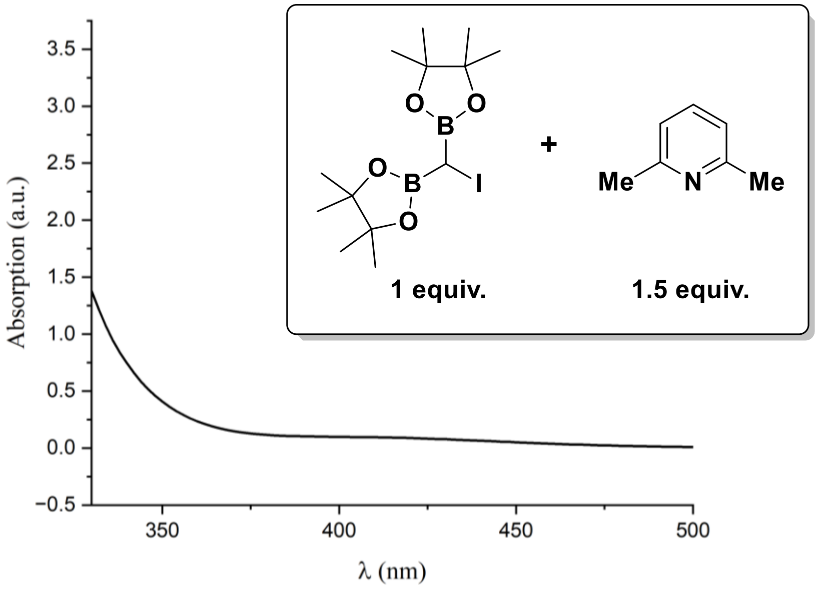

Figure S13. Absorption spectrum of **1c** (0.05 M) and 2,6-lutidine (0.075 M) in MeCN. Uv-Vis recorded after 15 min of stirring in the dark.

Comment: Mixtures of iodo compounds **1a - 1c** with 2,6-lutidine led to no detectable EDA formation in the visible light region. A bathochromic shift was observed for compounds **1b** and **1c** upon mixing with 2,6-lutidine, with absorption beginning at 369 and 396 nm respectively. The mixture containing **1a** and 2,6-lutidine did not showcase a change in absorbance at reaction concentration, and only a slight bathochromic shift at higher concentration (absorbance beginning at 385 nm).

Absorption spectra of mixtures under light irradiation

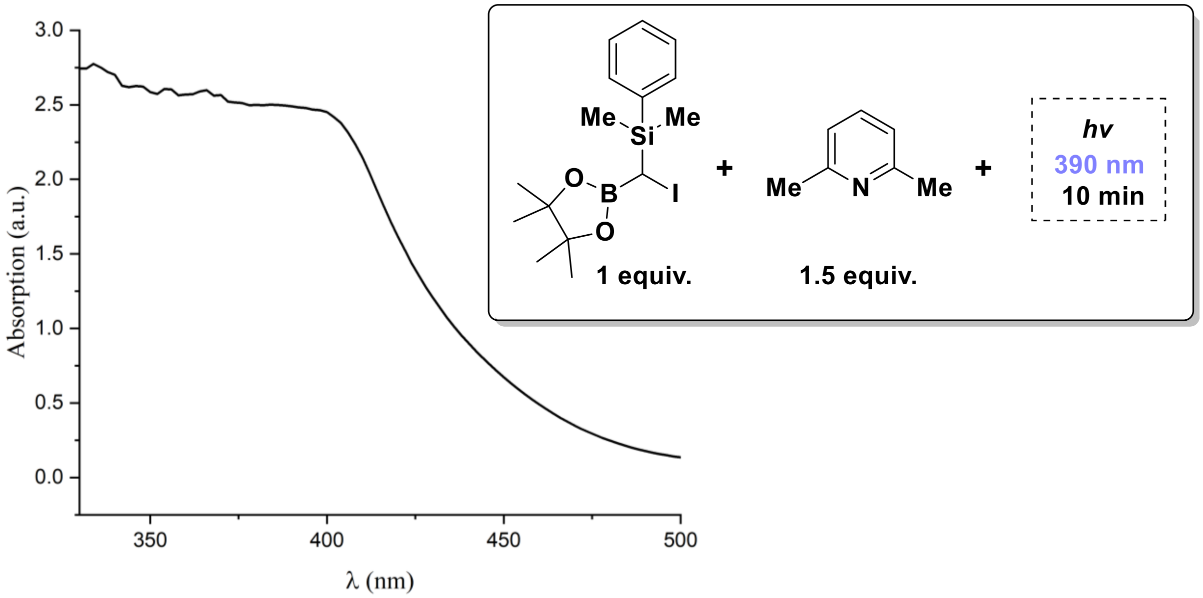

Figure S14. Absorption spectrum of **1a** (0.05 M) and 2,6-lutidine (0.075 M) in MeCN. Uv-Vis recorded after 10 min of irradiation (390 nm, PR-160L).

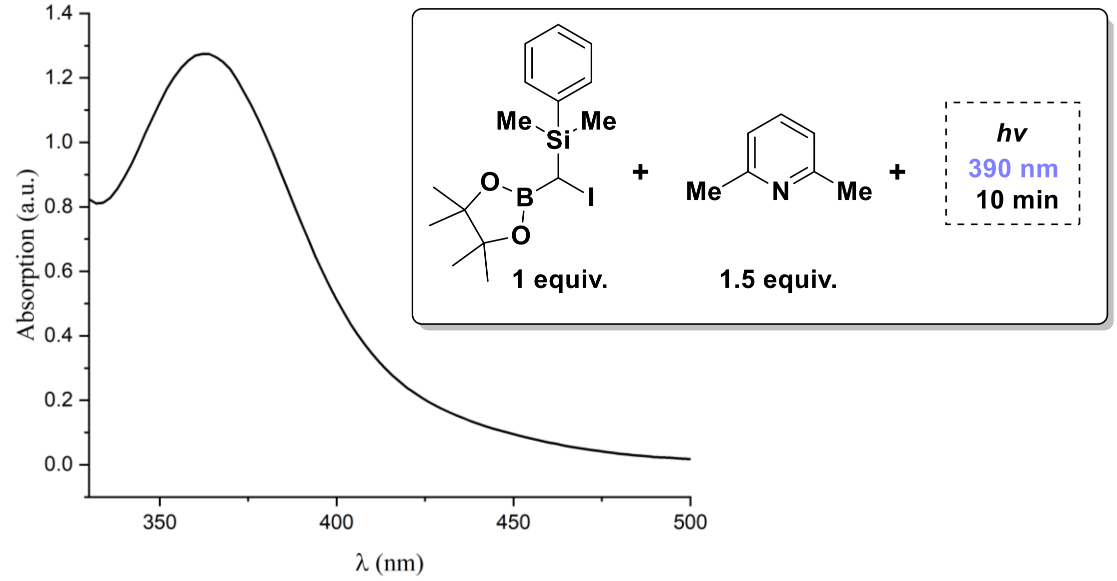

Figure S15. Absorption spectrum of **1a** (0.05 M) and 2,6-lutidine (0.075 M) in MeCN. Uv-Vis recorded after 10 min of irradiation (390 nm, PR-160L). Sample was subjected to 10-fold dilution.

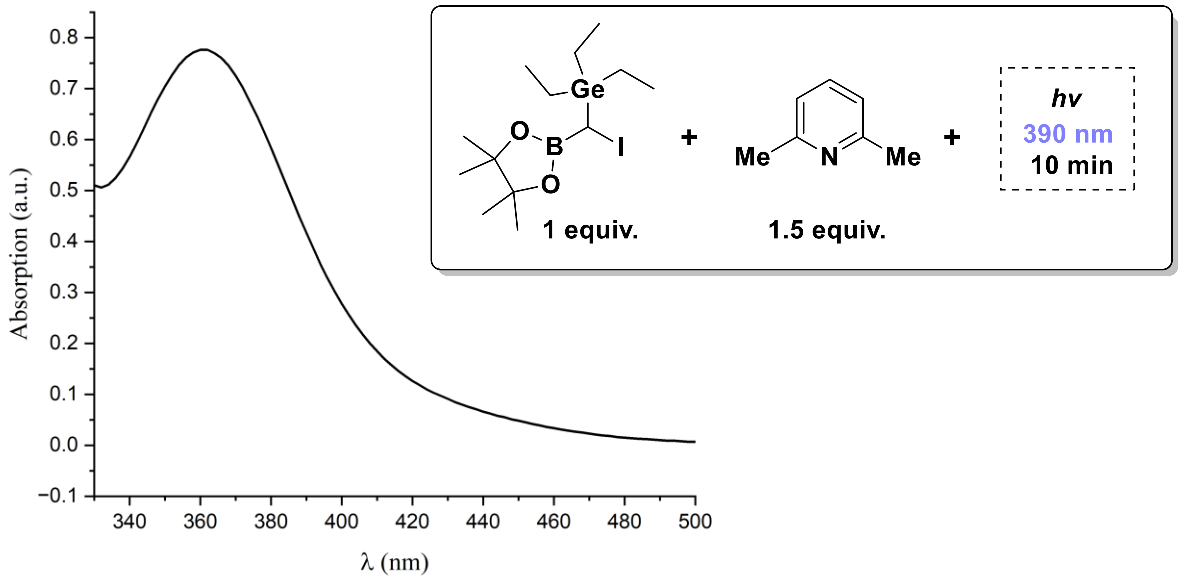

Figure S16. Absorption spectrum of **1b** (0.05 M) and 2,6-lutidine (0.075 M) in MeCN. Uv-Vis recorded after 10 min of irradiation (390 nm, PR-160L). Sample was subjected to 10-fold dilution.

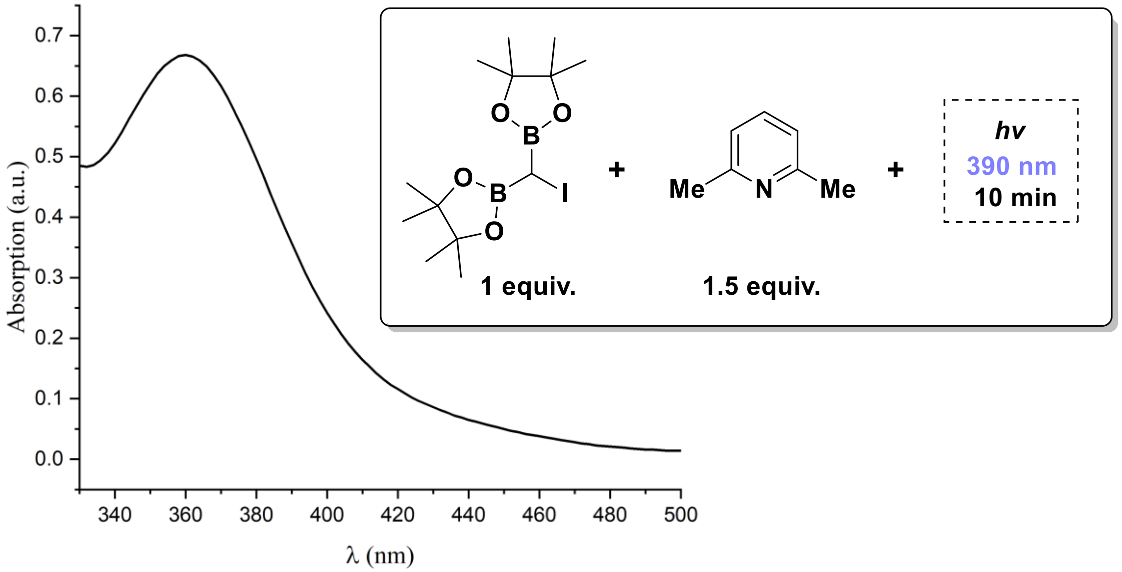

Figure S17. Absorption spectrum of **1c** (0.05 M) and 2,6-lutidine (0.075 M) in MeCN. Uv-Vis recorded after 10 min of irradiation (390 nm, PR-160L). Sample was subjected to 10-fold dilution.

Comment: The exposure of iodo compounds **1a-1c** with 2,6-lutidine resulted in the formation of a new band (λ_{max} = ~364 nm), which can be assigned as triiodide (see Figure S18). This demonstrates the homolytic cleavage of the C-I bond was possible.

Absorption spectra of control components – Evidence for triiodide formation

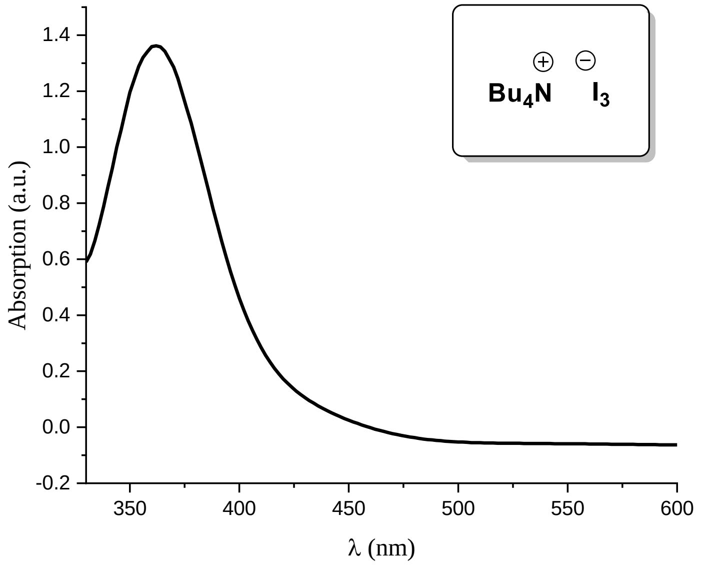

Figure S18. Absorption spectra of commercial $Bu_4N^+I_3^-$ in MeCN (≈6 x 10^{-5} M).

Cyclic Voltammetry Studies

Cyclic voltammetry was conducted on an Interface 1000 Gamry potentiostat using a 3-electrode cell configuration. A glassy carbon working electrode was employed alongside a platinum wire counter electrode and an Ag/Ag^+ reference electrode. The solution was degassed by bubbling nitrogen prior to measurements. The analysis was carried out on a 5 mM solution in MeCN along with 0.1 M of tetrabutylammonium hexafluorophosphate as supporting electrolyte and with 5 mM of ferrocene as internal standard. It was examined at a scan rate of 0.05 V s^{-1}. Potential value is given versus the saturated calomel electrode (SCE). An irreversible wave was obtained; therefore, the potential was estimated at half the maximum current.[25]

1a, E_{red} (V vs SCE) = -1.616 V

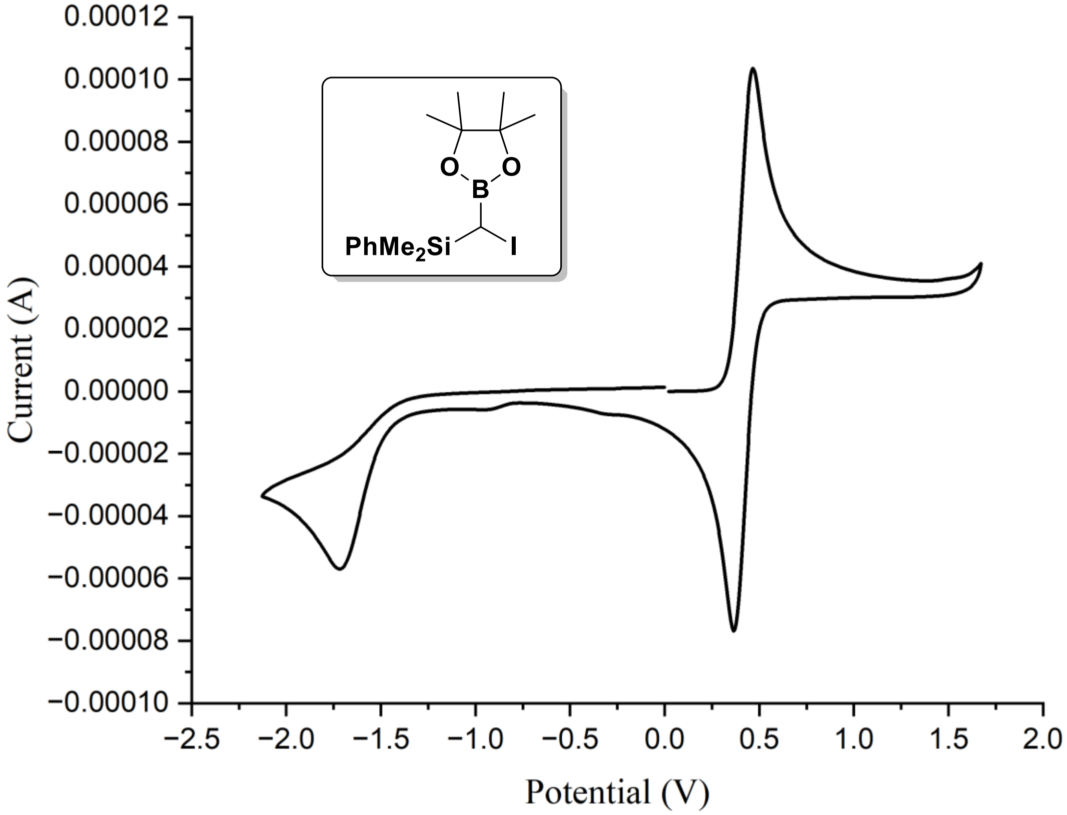

Figure S19. CV of **1a.**

E_{red} (V vs SCE) = -1.618 V

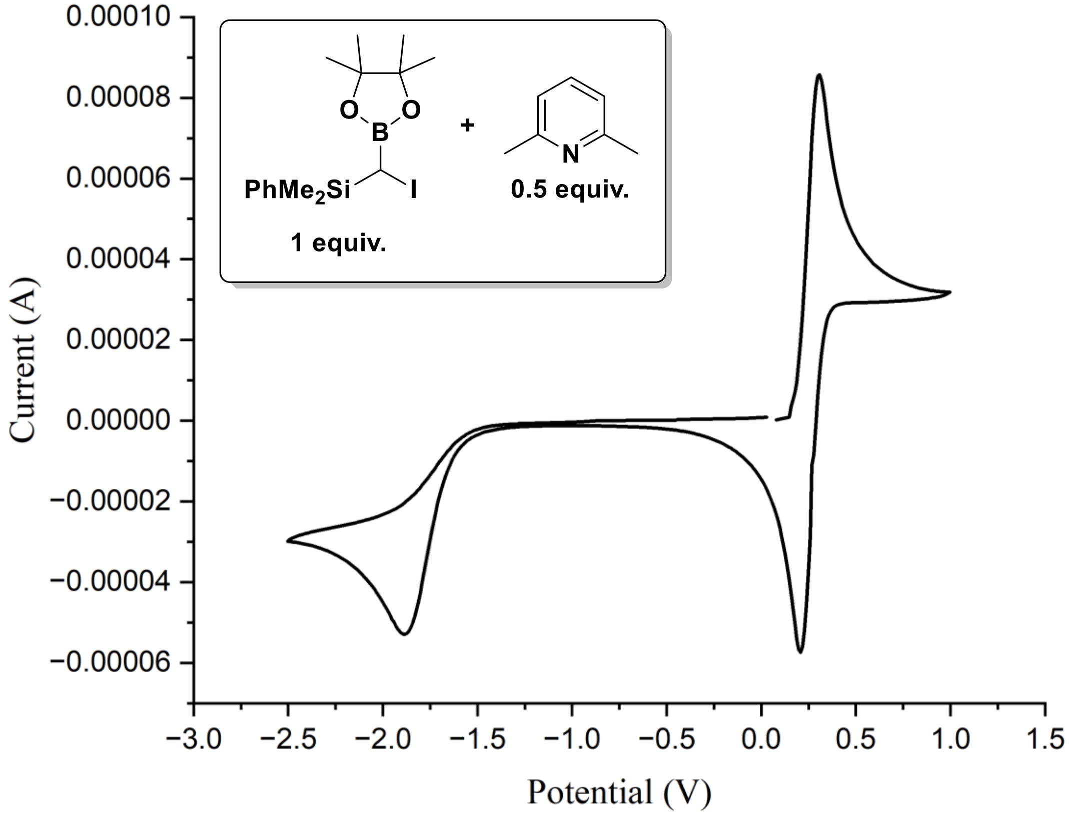

Figure S20. CV of **1a** (1 equiv.) and 2,6-lutidine (0.5 equiv.).

E_{red} (V vs SCE) = -1.608 V

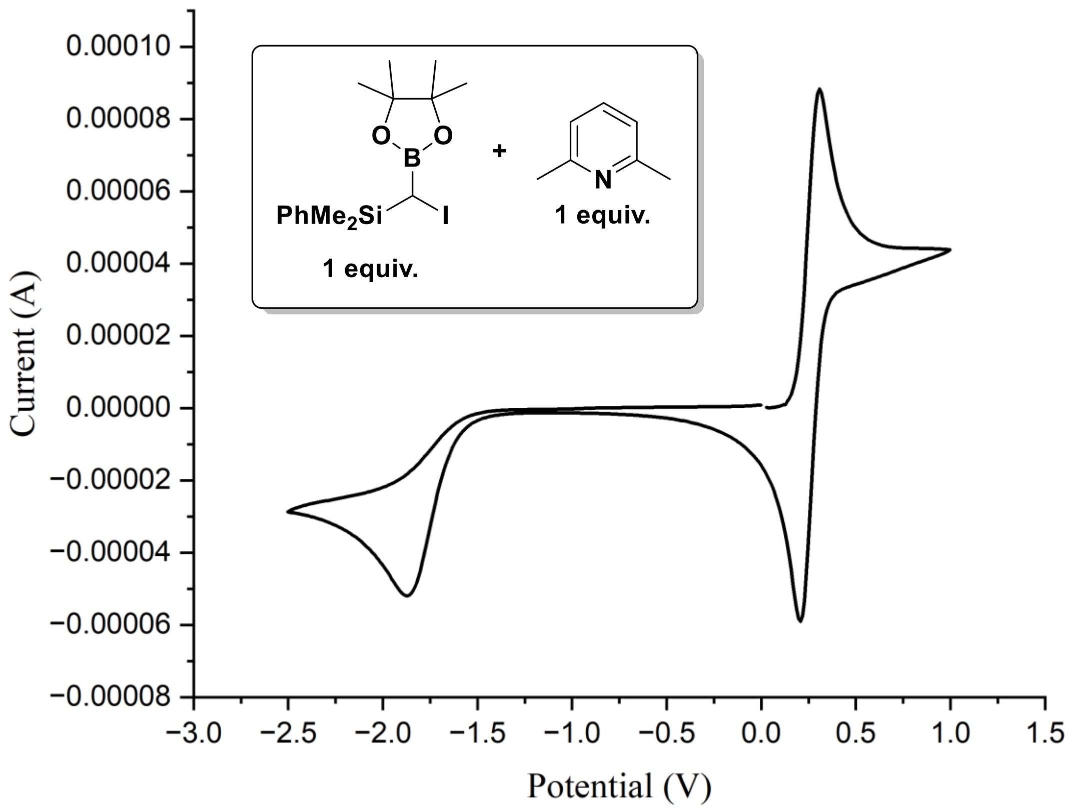

Figure S21. CV of **1a** (1 equiv.) and 2,6-lutidine (1 equiv.).

E_{red} (V vs SCE) = -1.620 V

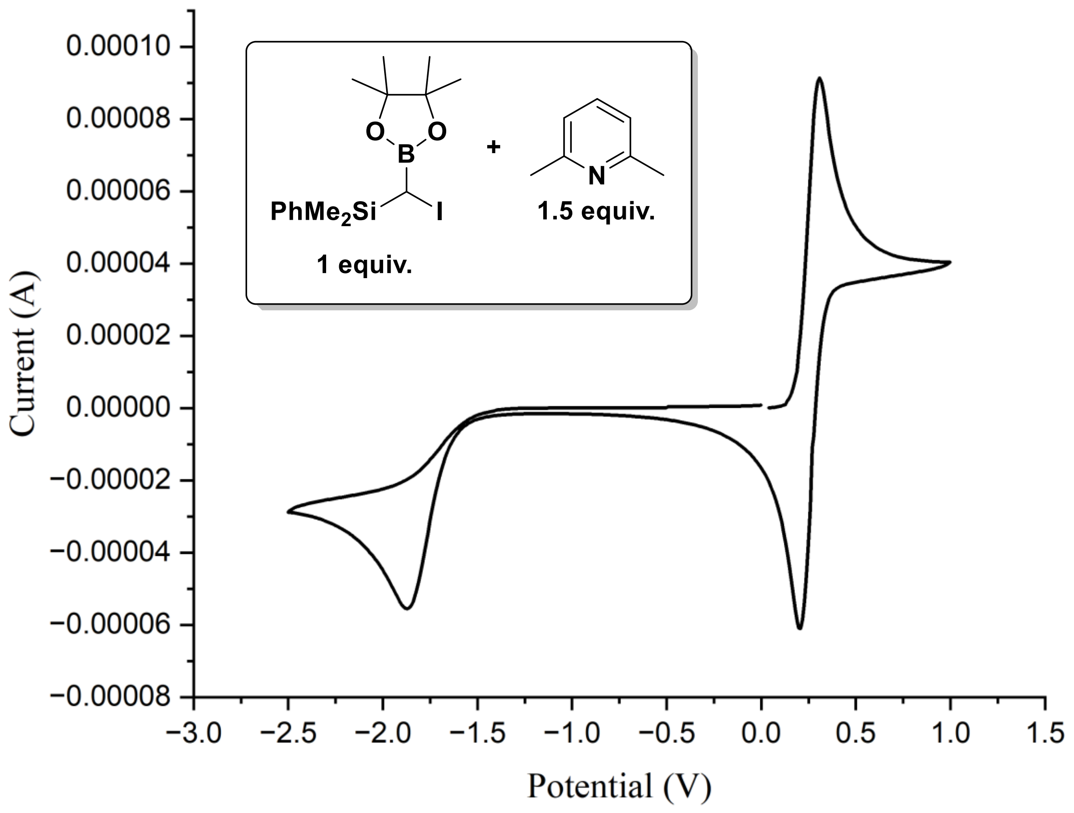

Figure S22. CV of **1a** (1 equiv.) and 2,6-lutidine (1.5 equiv.).

E_{red} (V vs SCE) = -1.616 V

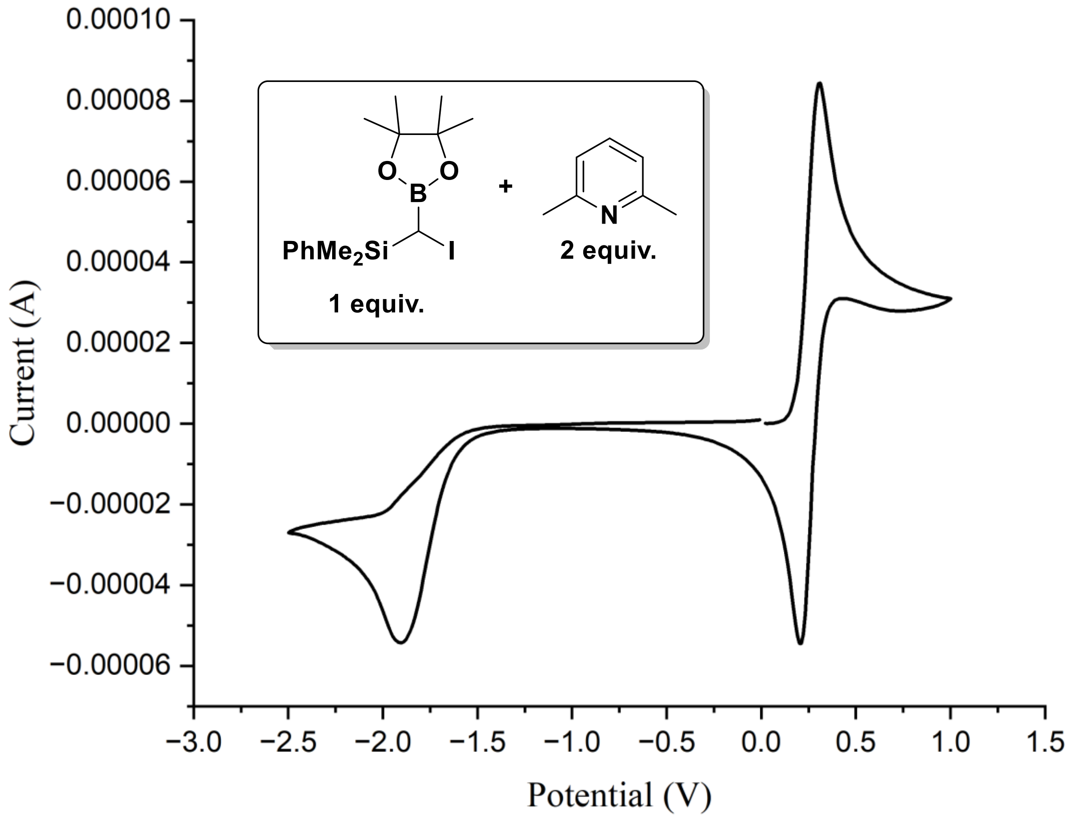

Figure S23. CV of **1a** (1 equiv.) and 2,6-lutidine (2 equiv.).

E_{red} (V vs SCE) = -1.595 V

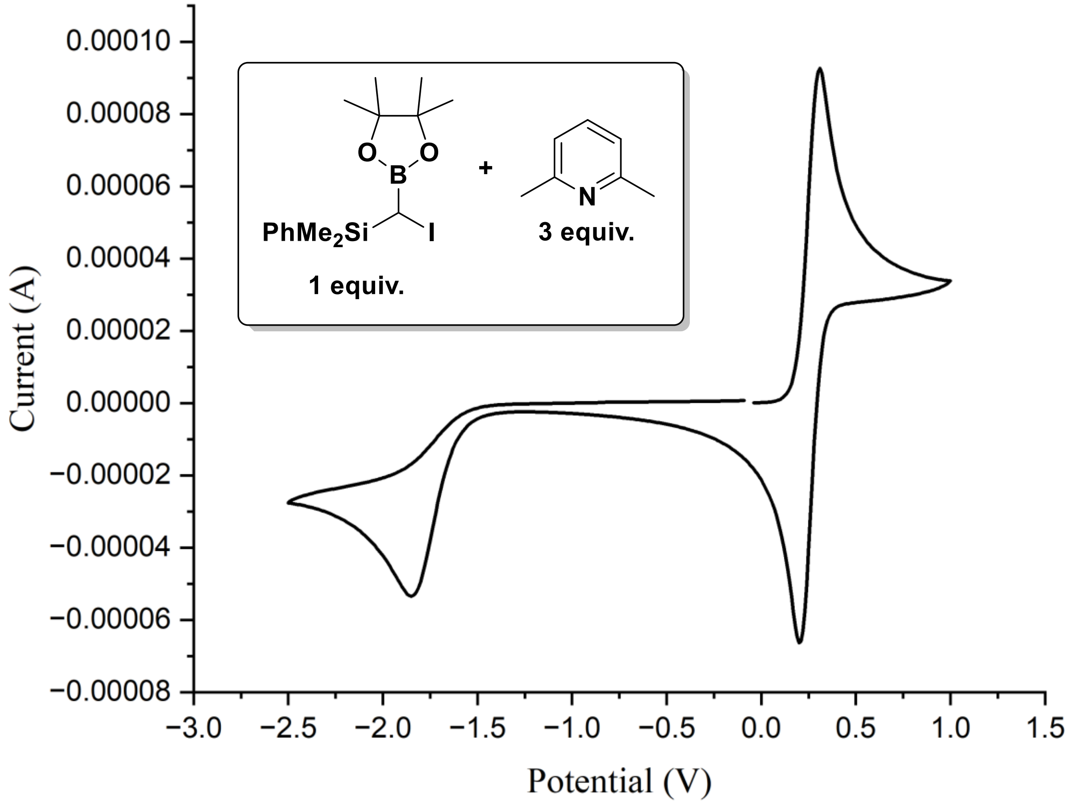

Figure S24. CV of **1a** (1 equiv.) and 2,6-lutidine (3 equiv.).

Comment: The increased addition of 2,6-lutidine to **1a** showed no significant variation to the measured reduction potential.

Time Interval Studies

On/Off Experiment

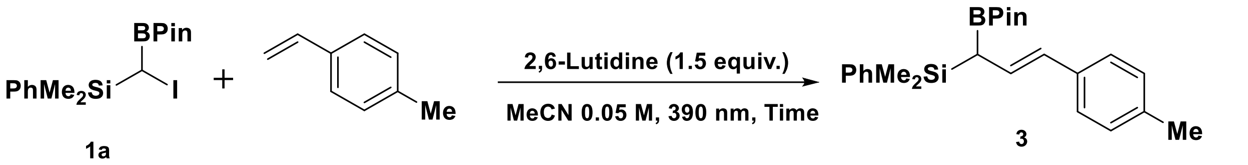

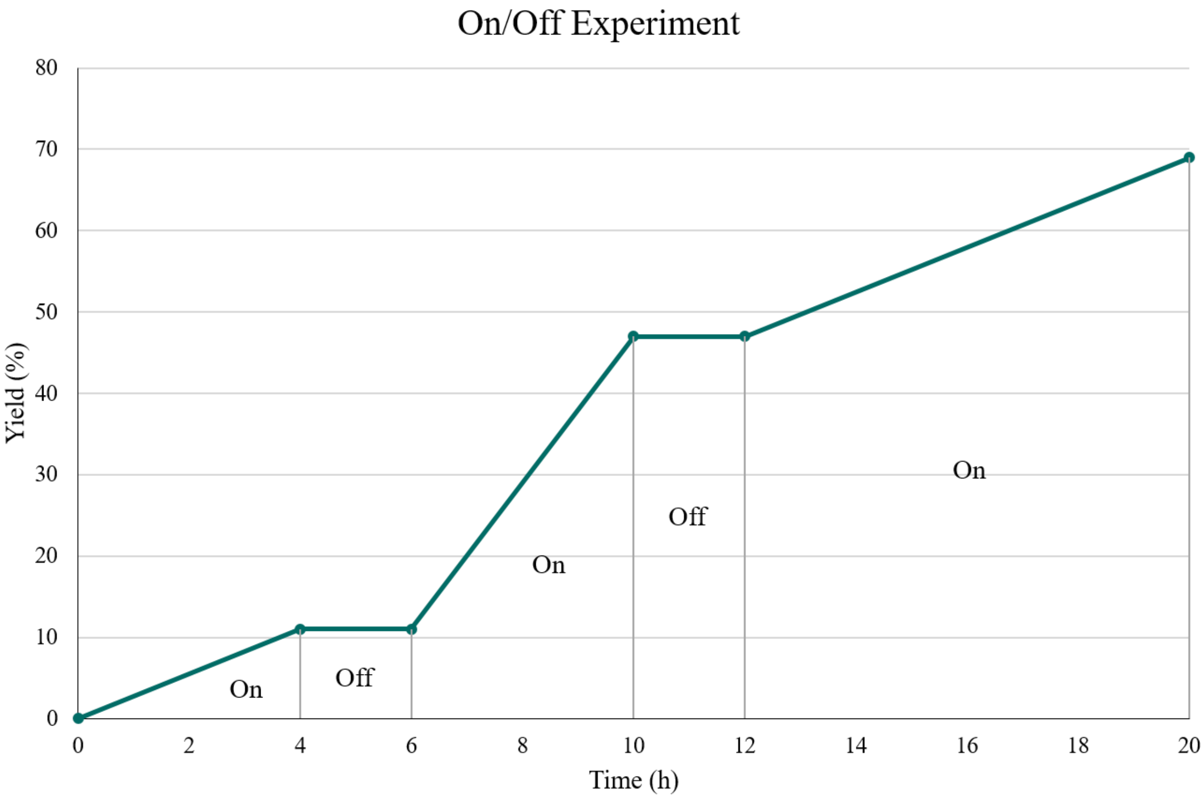

Figure S25. Time profile of the transformation of **3** with the irradiation on/off over time.

To 5 identical oven-dried 5 mL microwave vials, **1a** (80.4 mg, 0.2 mmol, 1 equiv.) was added. The vials were sealed with a septum and purged with nitrogen before the sequential addition of degassed MeCN (4 mL), 2,6-lutidine (35 µL, 0.3 mmol, 1.5 equiv.) and 4-methylstyrene (79 µL, 0.6 mmol, 3 equiv.) via syringe. The reaction mixtures were stirred under light irradiation (390 nm, PR-160L) for 4 h. One reaction vial was removed and the lights turned off for 2 h. To the removed vial was added internal standard (1,3,5-trimethoxybenzene), as a solution in MeCN, and the reaction was washed with a 1 M sol. of K_2CO_3 (10 mL). Organics were extracted with EtOAc (3 x 10 mL). The combined organic phases were dried over Na_2SO_4, filtered and concentrated under reduced pressure. The remaining reaction vials were stirred in the dark for 2 h. One reaction vial was removed and the lights turned on for another 4 h. This cycle was repeated for remaining samples.

Table S12. On/Off Experiment Results

Time (h)	Yield (%), (E:Z)
0	0
4	11 (82:18)
6	11 (82:18)
10	47 (89:11)
12	47 (89:11)
20	69 (89:11)

Comment: In the absence of light irradiation there was no increase in reaction yield observed. While this does give evidence against the reaction proceeding via a radical chain mechanism with a long propagation length, it cannot rule out the presence of a radical chain mechanism with short propagations.[26]

Time Interval Study

***E*-Reaction:**

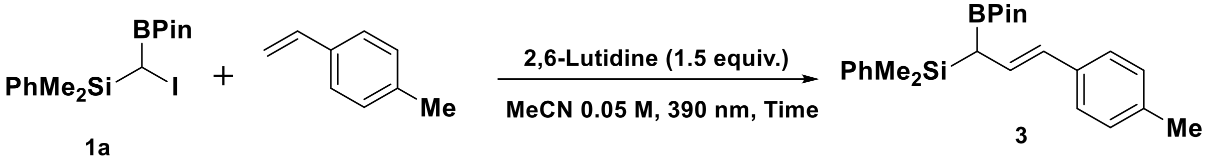

To 4 identical oven-dried 5 mL microwave vials, **1a** (80.4 mg, 0.2 mmol, 1 equiv.) was added. The vials were sealed with a septum and purged with nitrogen before the sequential addition of degassed MeCN (4 mL), 2,6-lutidine (35 µL, 0.3 mmol, 1.5 equiv.) and 4-methylstyrene (79 µL, 0.6 mmol, 3 equiv.) via syringe. The reaction mixtures were stirred under light irradiation (390 nm, PR-160L) with one reaction vial removed every 4 h. To the removed vial was added internal standard (1,3,5-trimethoxybenzene), as a solution in MeCN, and the reaction was washed with a 1 M sol. of K_2CO_3 (10 mL). Organics were extracted with EtOAc (3 x 10 mL). The combined organic phases were dried over Na_2SO_4, filtered and concentrated under reduced pressure.

***Z*-Reaction:**

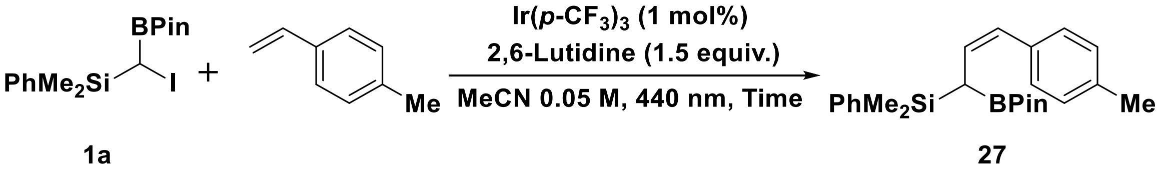

To 4 identical oven-dried 5 mL microwave vials, **1a** (80.4 mg, 0.2 mmol, 1 equiv.) and Ir(*p*-CF_3)$_3$ (1.7 mg, 1 mol%) were added. The vials were sealed with a septum and purged with nitrogen before the sequential addition of degassed MeCN (4 mL), 2,6-lutidine (35 µL, 0.3 mmol, 1.5 equiv.) and 4-methylstyrene (79 µL, 0.6 mmol, 3 equiv.) via syringe. The reaction mixtures were stirred under light irradiation (440 nm, PR-160L) with one reaction vial removed every 4 h. To the removed vial was added internal standard (1,3,5-trimethoxybenzene), as a solution in MeCN, and the reaction was washed with a 1 M sol. of K_2CO_3 (10 mL). Organics were extracted with EtOAc (3 x 10 mL). The combined organic phases were dried over Na_2SO_4, filtered and concentrated under reduced pressure.

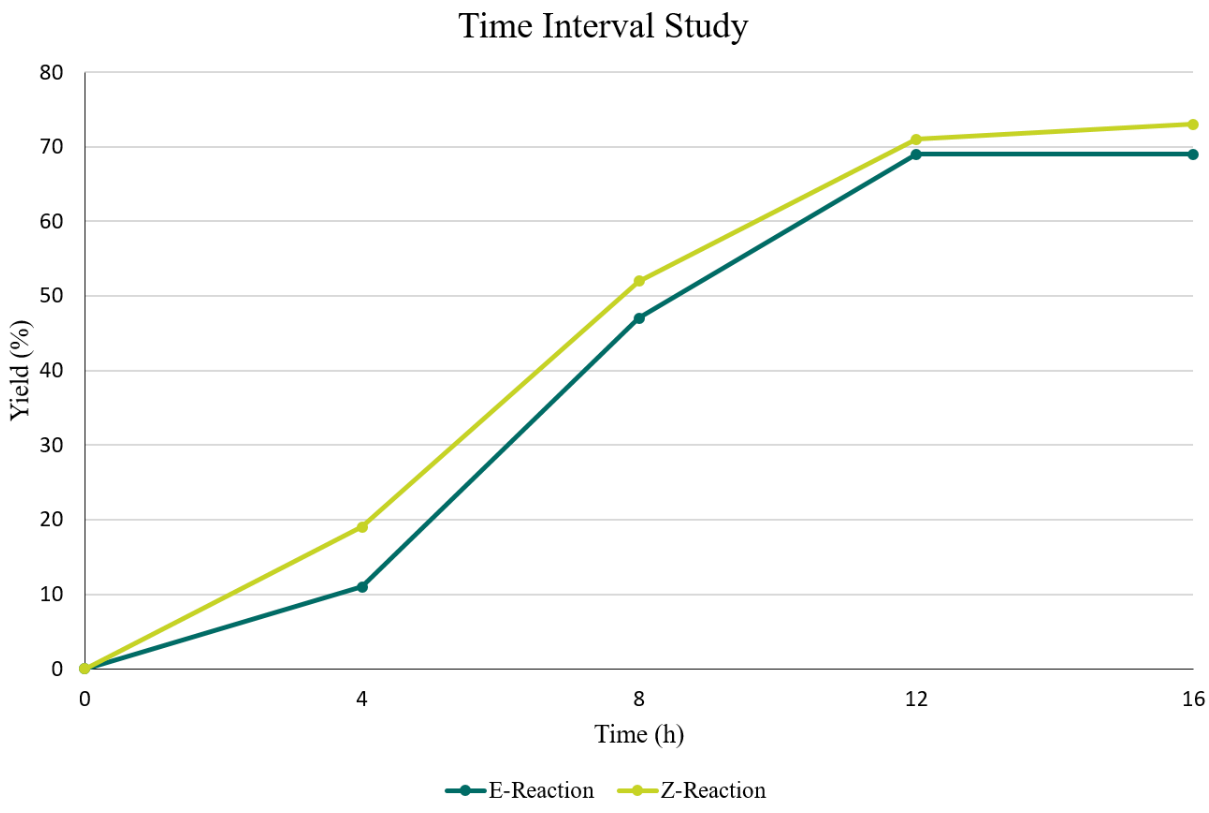

Figure S26. Time profile for product formation of **E-Reaction** (dark green) and **Z-Reaction** (light green) over time.

Table S13. Time Interval Study Results for E-Reaction and Z-Reaction

Time (h)	Yield of 3 (%) (E:Z)	Yield of 27 (%) (Z:E)
0	0	0
4	11 (82:18)	19 (>95:5)
8	47 (91:9)	52 (>95:5)
12	69 (89:11)	71 (>95:5)
16	69 (89:11)	73 (>95:5)

Comment: The results showcase that the reaction rate of both the E-reaction and Z-reaction are similar, with the most significant increase in yield occurring between the 4 and 8 h mark.

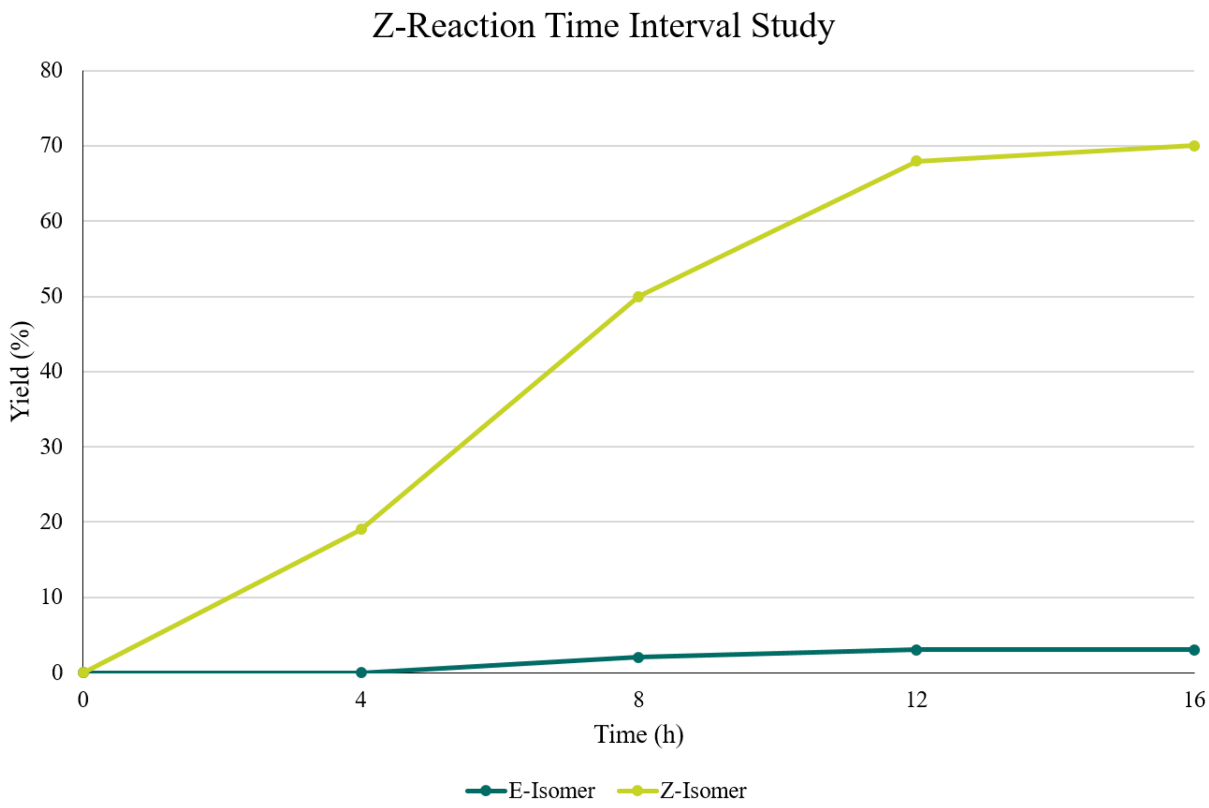

Figure S27. Time profile of product formation in **Z-Reaction**, with **E-isomer** (dark green) and **Z-Isomer** (light green).

Table S14. Time Interval Study Z-Reaction Results

Time (h)	E-Isomer Yield (%)	Z-Isomer Yield (%)
0	0	0
4	0	19
8	2	50
12	3	68
16	3	70

Comment: Results indicate the rate of the $E \rightarrow Z$ isomerization EnT process is greater than the formation of E-isomer **3**, with concentrations of **3** remaining consistently low while **27** increases.

NMR Analysis

NMR investigation of boronate formation

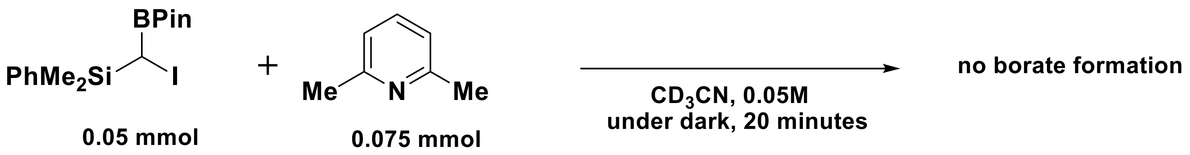

To an oven-dried 5 mL microwave vial, **1a** (20.1 mg, 0.05 mmol) was added. The vial was sealed with a septum and purged with nitrogen before the sequential addition of deuterated MeCN (1 mL) and 2,6-lutidine (9 µL, 0.075 mmol) via syringe. The mixture was stirred for 20 minutes in the dark and the respective NMR was compared to the parent starting material.

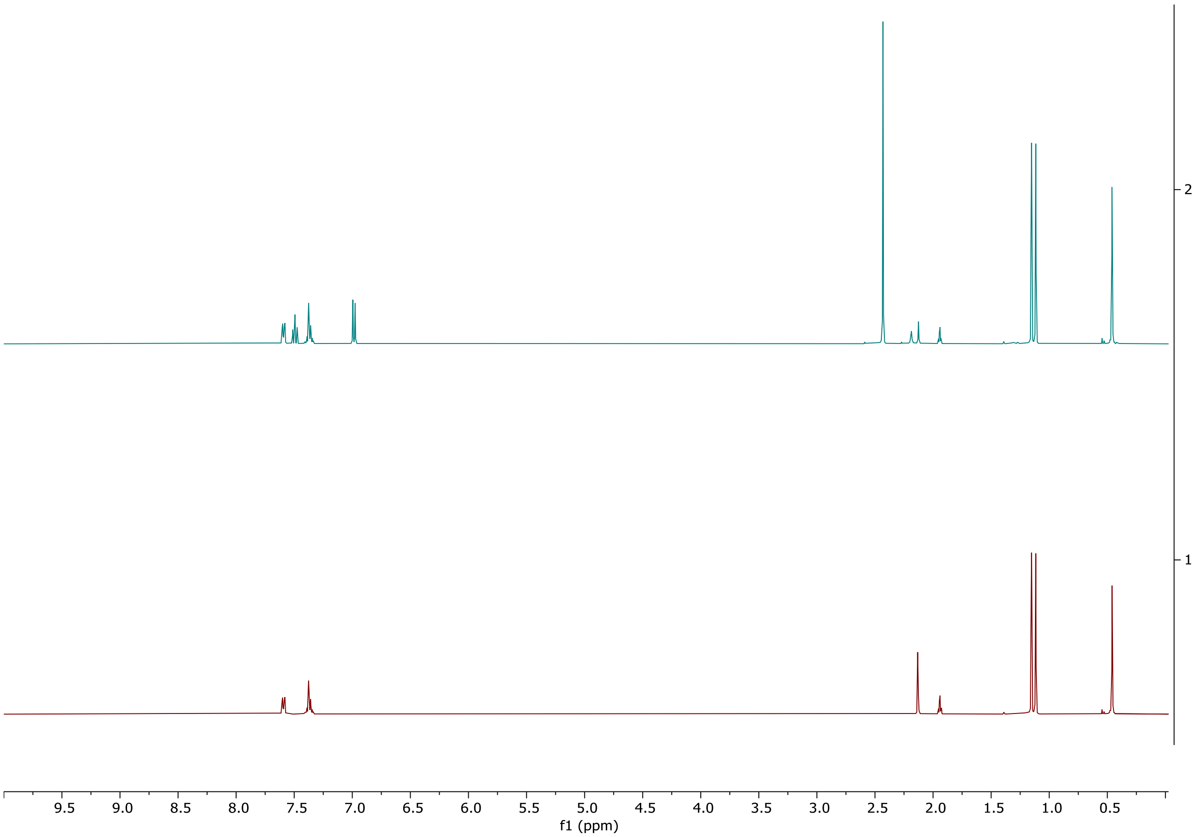

Figure S28. Top: **^{1}H NMR** spectrum of **1a** and 2,6-lutidine in CD_3CN after 20 min stirring; Bottom: **^{1}H NMR** spectrum of **1a** in CD_3CN;

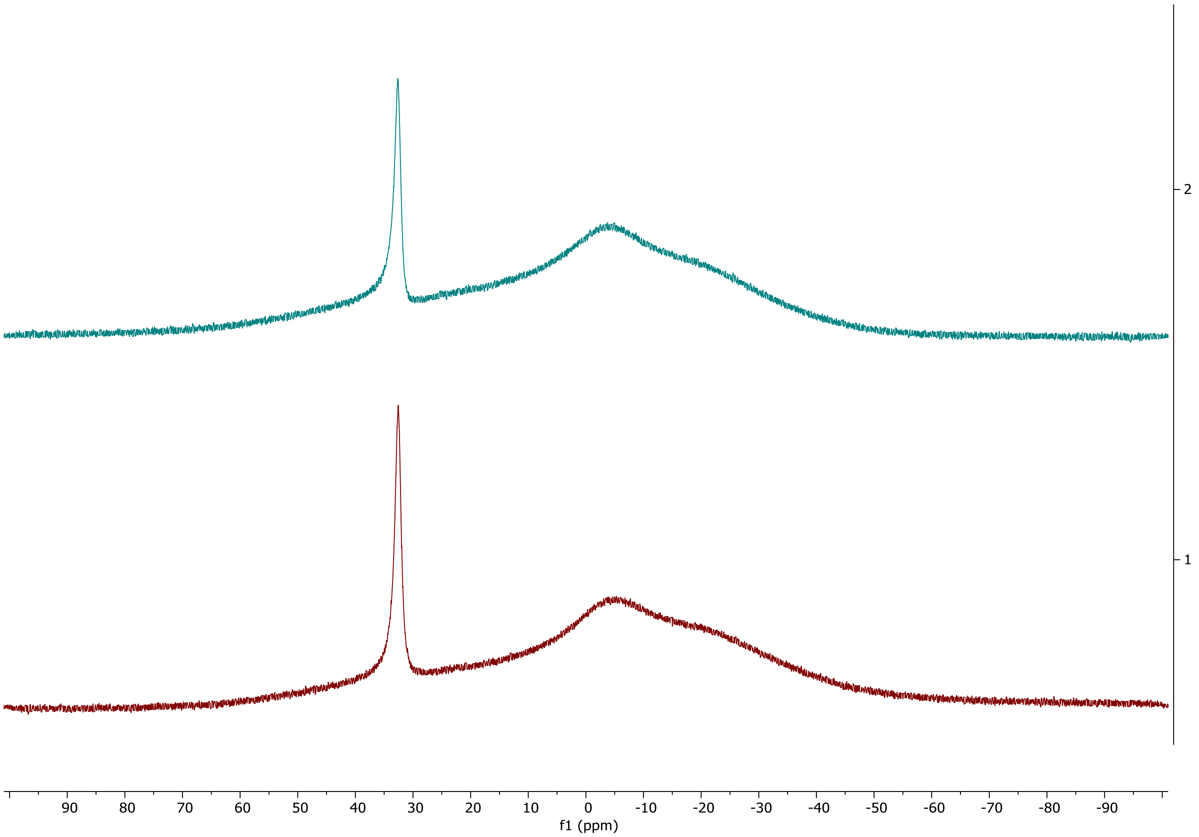

Figure S29. Top: **^{11}B NMR** spectrum of **1a** and 2,6-lutidine in CD_3CN after 20 min stirring; Bottom: **^{11}B NMR** spectrum of **1a** in CD_3CN.

Comment: Upon the mixing of **1a** and 2,6-lutidine, formation of a borate complex was not observed in ^{11}B NMR. This gives indication an interaction between the boron p-orbital and the nitrogen of 2,6-lutidine is unlikely.

There was no change observed in the ^{1}H NMR.

Reaction Optimization: *Z*-Allylic Boronic Esters

Table S15: Catalyst screening.

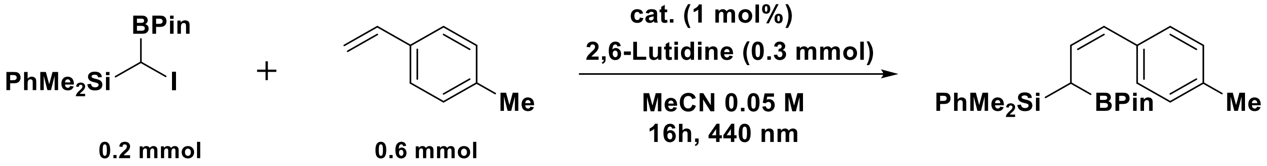

Entry	Catalyst	$E_{1/2}$(M*/M+)[27]	E_T (Kcal/mol)[27]	Yield[a]	*Z*:*E* ratio[a]
34[b]	Xanthone	-1.65 V	74.2	0%	-
35[c]	Thioxanthone	-1.62 V	65.5	10%	90:10
36	$[Ir(dF(Me)ppy)_2(dtbbpy)]PF_6$	-0.92 V	62.9	44%	77:23
**37**	**Ir(*p*-CF$_3$)$_3$**	**-1.695 V**	**56.4**	**73%**	**>95:5**
38	Ir(*p*-F-ppy)$_3$	-1.905 V	58.6	66%	85:15
39	$[Ru(dmbpy)_3](PF_6)_2$	-1.48 V	45.3	10%	5:>95
40[d]	Eosin Y	-1.08 V	45.4	0%	-
41[d]	4CzIPN	-1.04 V	58.3	<5%	5:>95

[a] Determined by 1H NMR spectroscopy against a known internal standard (1,3,5-trimethoxybenzene). [b] 5 mol% cat. loading and ran under 370 nm irradiation. [c] 5 mol% cat. loading and ran under 390 nm irradiation. [d] 5 mol% cat. loading.

Determining of the Origin of Diastereoselectivity

In order to establish that the reaction proceeds via formation of the E-isomer and subsequent isomerisation via selective energy transfer we looked to probe the E→Z isomerisation of our allylic system under representative reaction conditions.

E$_n$T enabled contra-thermodynamic E to Z isomerisation of **3** *to* **27**

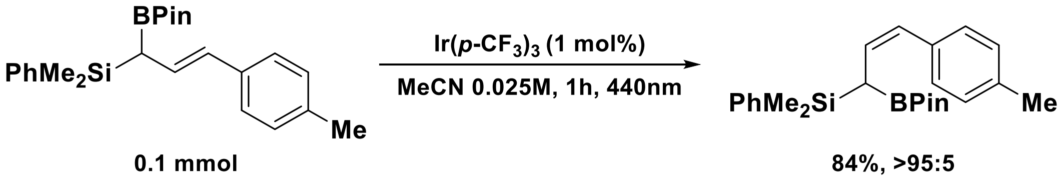

To an oven-dried 5 mL microwave vial, **3** (39.1 mg, 0.1 mmol, 1 equiv.) and Ir(p-CF$_3$)$_3$ (0.9 mg, 1 mol%) were added. The vial was sealed with a septum and purged with nitrogen before the addition of degassed MeCN (4 mL) via syringe. The reaction mixture was stirred under light irradiation (440 nm, PR-160L, 25% Intensity) for 1 h. After completion, internal standard (1,3,5-trimethoxybenzene), as a solution in MeCN, was added and the mixture was transferred to a flask and concentrated under reduced pressure. Crude NMR analysis against the internal standard revealed isomerisation to **27** had proceeded (84%, >95:5 *Z*:*E*).

Control Reactions

Table S16. Control Reactions for Photocatalysed Reaction.

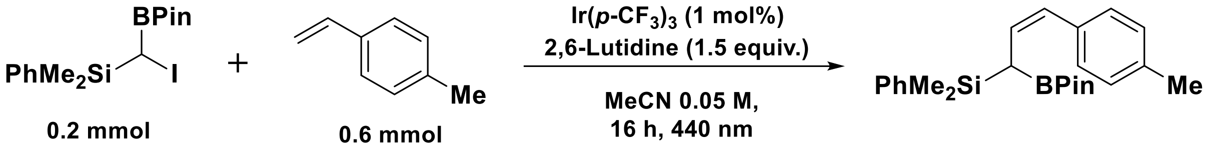

Entry	Deviation from Standard Conditions	Yield[a] (%) (Z:E)	1a Retention[a] (%)
42	No Photocatalyst	0	93
43	No Additive	19 (>95:5)	78
44	Presence of O_2	31 (>95:5)	43
45	Presence of TEMPO (1 equiv.)	0	63

[a] Determined by 1H NMR spectroscopy against a known internal standard (1,3,5-trimethoxybenzene).

Substrate Scope: *Z*-Allylic Boronic Esters

General Procedure G: Photocatalytic generation of *Z*-allyl boron pinacol esters.

To an oven-dried 5 mL microwave vial, Ir(*p*-CF_3)$_3$ (1.7 mg, 1 mol%) and the specified α-iodo boronic acid pinacol ester (0.2 mmol, 1 equiv.) were added. The vial was sealed with a septum and purged with nitrogen before the sequential addition of degassed MeCN (4 mL), heteroarene additive (0.3 mmol, 1.5 equiv.) and styrene (0.6 mmol, 3 equiv.) via syringe. The styrene, when solid, could also be weighed directly prior to sealing and purging. The reaction mixture was stirred under light irradiation (440 nm, PR-160L) for 16 h. After completion, internal standard (1,3,5-trimethoxybenzene), as a solution in MeCN, was added and the reaction was washed with a 1 M sol. of K_2CO_3 (10 mL). Organics were extracted with EtOAc (3 x 10 mL). The combined organic phases were dried over Na_2SO_4, filtered and concentrated under reduced pressure. The crude residue was purified by flash column chromatography (SiO_2, specified combination of solvents). Borylated products purified by SiO_2 were exposed to the minimum amount of SiO_2 for as little time as possible to limit degradation.

(*Z*)-(1-(Dimethyl(phenyl)silyl)-3-(p-tolyl)allyl)boronic acid, pinacol ester (27)

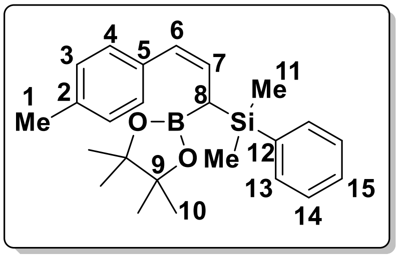

Prepared according to General Procedure **G**, **1a** (80.4 mg, 0.2 mmol), and 4-methylstyrene (79 µL, 0.6 mmol) were converted to **27** yielding a yellow oil (73% NMR yield, >95:5 (*Z*:*E*), 53.3 mg, 68%), after purification by flash column chromatography (SiO_2, 0→0.25% EtOAc/*n*-hexane).

R*f* (10% EtOAc/*n*-hexane) = 0.52; **^{1}H NMR** (400 MHz, $CDCl_3$) δ = 7.53 – 7.48 (m, 2H, H14), 7.36 – 7.28 (m, 3H, H13 and H15), 7.09 (m, 4H, H3 and H4), 6.27 (d, *J* = 11.5 Hz, 1H, H6), 5.79 (dd, *J* = 12.5, 11.5 Hz, 1H, H7), 2.58 (d, *J* = 12.7 Hz, 1H, H8), 2.34 (s, 3H, H1), 1.20 (s, 6H, H10), 1.17 (s, 6H, H10), 0.35 (m, 6H, H11) ppm; **^{13}C NMR** (101 MHz, $CDCl_3$) δ = 138.1 (C12), 135.5 (C2), 135.3 (C5), 134.1 (C14), 129.1 (C15), 128.9, 128.7 (C7), 128.6, 127.6 (C13), 126.0 (C6), 83.2 (C9), 25.0 (C10), 25.0 (C10), 21.3 (C1), -2.7 (C11), -2.8 (C11) ppm; **^{11}B NMR** (128 MHz, $CDCl_3$) δ = 32.71 ppm; **IR** (ATR): ṽ = 3071, 3050, 2979, 2926, 1623, 1512, 1483, 1448, 1427, 1417, 1372, 1315, 1248, 1213, 1166, 1141, 1113, 1053, 1007, 969, 948, 905, 833, 816, 763, 733, 700, 660 cm^{-1}; **HRMS** (EI) cacld. for $C_{24}H_{33}BO_2Si$ $[M]^+$ 392.2343, found 392.2335.

(*Z*)-(3-(*p*-tolyl)prop-2-ene-1,1-diyl)diboronic acid, pinacol ester (28)

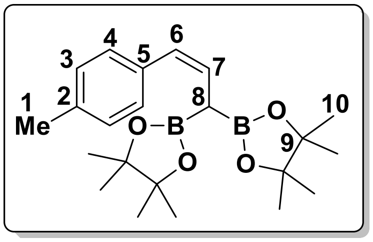

Prepared according to a modified version of General Procedure **G**, in which the reaction was washed with sat. aq. NH_4Cl instead of 1 M K_2CO_3, **1c** (78.8 mg, 0.2 mmol), and 4-methylstyrene (79 µL, 0.6 mmol) were converted to **28** yielding a yellow solid (64% NMR yield, >95:5 (*Z*:*E*), 42.4 mg, 55%), containing a 23% alkyl impurity after purification by flash column chromatography (C18, 20→100% MeCN/H2O (0.1% Formic Acid)). (Note: Basic work-up lead to protodeboronation of desired product)

^{1}H NMR (400 MHz, $CDCl_3$) δ = 7.22 (d, *J* = 8.1 Hz, 2H, H4), 7.10 (d, *J* = 7.9 Hz, 2H, H3), 6.33 (d, *J* = 11.4 Hz, 1H, H6), 5.92 (t, *J* = 11.3 Hz, 1H, H7), 2.47 (d, *J* = 11.1 Hz, 1H, H8), 2.32 (s, 3H, H1), 1.24 (s, 12H, H10), 1.23 (s, 12H, H10) ppm; **^{13}C NMR** (101 MHz, $CDCl_3$) δ = 135.8 (C2), 135.1 (C5), 128.9 (C3), 128.7 (C4), 128.0 (C7), 126.8 (C6), 83.5 (C9), 24.8 (C10), 24.8 (C10), 21.3 (C1) ppm; **^{11}B NMR** (128 MHz, $CDCl_3$) δ = 32.54 ppm; **IR** (ATR): ṽ = 2978, 2926, 1513, 1370, 1347, 1318, 1263, 1215, 1166, 1137, 1074, 1005, 968, 907, 850, 824, 758, 729, 701, 669 cm^{-1}; **HRMS** (EI) cacld. for $C_{22}H_{34}B_2O_4$ $[M]^+$ 384.2643, found 384.2639.

(*Z*)-(1-(Dimethyl(phenyl)silyl)-3-(2-(2-methoxy-2-oxoethyl)phenyl)allyl)boronic acid, pinacol ester (30)

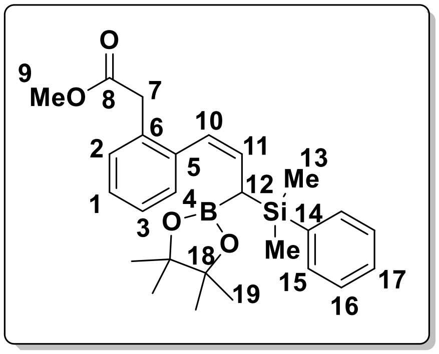

Prepared according to General Procedure **G**, **1a** (80.4 mg, 0.2 mmol), and **S7** (97.3 mg, 0.6 mmol) were converted to **30** yielding a yellow oil (43% NMR yield, >95:5 (*Z*:*E*), 35 mg, 39%), after purification by flash column chromatography (C18, 20→100% MeCN/H_2O).

R_f (10% EtOAc/*n*-hexane) = 0.28; **^{1}H NMR** (400 MHz, $CDCl_3$) δ = 7.51 – 7.46 (m, 2H), 7.45 – 7.40 (m, 1H), 7.39 – 7.34 (m, 2H), 7.28 – 7.23 (m, 3H), 7.08 – 7.04 (m, 1H), 6.35 (d, *J* = 11.3 Hz, 1H, H10), 6.00 (dd, *J* = 12.6, 11.2 Hz, 1H, H11), 3.74 (s, 3H, H9), 3.49 (d, *J* = 2.7 Hz, 2H, H7), 2.28 (d, *J* = 12.6 Hz, 1H, H12), 1.31 (s, 6H, H19), 1.29 (s, 6H, H19), 0.42 (s, 3H, H13), 0.39 (s, 3H, H13) ppm; **^{13}C NMR** (101 MHz, $CDCl_3$) δ = 172.3 (C8), 138.0 (C14), 137.5, 134.0, 132.5, 130.7 (C11), 129.9, 129.7, 129.1 (C17), 127.7, 127.0, 126.7, 124.4 (C10), 83.2 (C18), 52.0 (C9), 38.7 (C7), 25.0 (C19), 24.9 (C19), -2.5 (C13), -3.3 (C13) ppm; **^{11}B NMR** (128 MHz, $CDCl_3$) δ = 32.55 ppm; **IR** (ATR): ṽ = 2926, 2855, 2369, 2302, 1737, 1600, 1457, 1363, 1251, 1212, 1190, 1178, 1142, 1113, 1097, 1020, 993, 850, 815, 764, 701, 688, 660 cm^{-1}; **HRMS** (EI) cacld. for $C_{26}H_{35}BO_4Si$ $[M]^+$ 450.2398, found 450.2458.

(*Z*)-(1-(Dimethyl(phenyl)silyl)-3-(4-methoxyphenyl)allyl)boronic acid, pinacol ester (31)

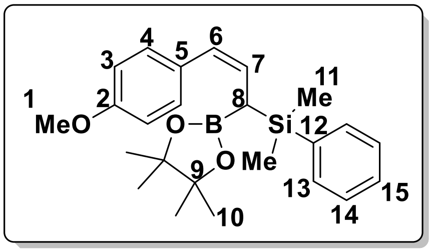

Prepared according to General Procedure **G**, **1a** (80.4 mg, 0.2 mmol), and 4-methoxystyrene (80 µL, 0.6 mmol) were converted to **31** yielding a yellow oil (88% NMR yield, 93:7 (*Z*:*E*), 58.6 mg, 67%), after purification by flash column chromatography (SiO_2, 0→2% EtOAc/*n*-hexane).

R_f (10% EtOAc/*n*-hexane) = 0.40; **^{1}H NMR** (400 MHz, $CDCl_3$) δ = 7.60 – 7.55 (m, 2H, H14), 7.45 – 7.33 (m, 3H, H13 and H15),

7.20 (d, *J* = 8.2 Hz, 2H, H4), 6.89 (d, *J* = 8.6 Hz, 2H, H3), 6.31 (d, *J* = 11.5 Hz, 1H, H6), 5.83 (t, *J* = 12.0 Hz, 1H, H7), 3.89 (s, 3H, H1), 2.63 (d, *J* = 12.5 Hz, 1H, H8), 1.28 (s, 6H, H10), 1.25 (s, 6H, H10), 0.43 (s, 6H, H11) ppm; **^{13}C NMR** (101 MHz, $CDCl_3$) δ = 157.8 (C2), 138.1 (C12), 134.1 (C14), 130.9 (C5), 129.8 (C4), 129.1 (C15), 127.9 (C7), 127.6 (C13), 125.6 (C6), 113.6 (C3), 83.2 (C9), 55.3 (C1), 25.0 (C10), -2.7 (C11), -2.8 (C11) ppm; **^{11}B NMR** (128 MHz, $CDCl_3$) δ = 32.83 ppm; **IR** (ATR): ῦ = 2979, 2837, 1608, 1574, 1510, 1465, 1443, 1428, 1391, 1372, 1312, 1247, 1176, 1141, 1112, 1036, 1005, 969, 909, 833, 815, 773, 731, 700, 662 cm^{-1}; **HRMS** (EI) calcd. for $C_{24}H_{33}BO_3Si$ $[M]^+$ 408.2292, found 408.2292.

(*Z*)-(1-(Dimethyl(phenyl)silyl)-3-(4-fluorophenyl)allyl)boronic acid, pinacol ester (32)

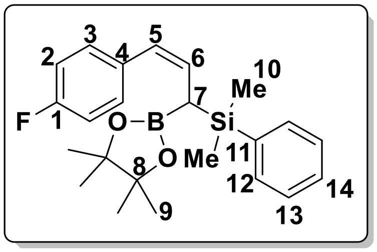

Prepared according to General Procedure **G**, **1a** (80.4 mg, 0.2 mmol), and 4-fluorostyrene (72 µL, 0.6 mmol) were converted to **32** yielding a yellow oil (76% NMR yield, 92:8 (*Z*:*E*), 50 mg, >95:5 (*Z*:*E*), 63%), after purification by flash column chromatography (SiO_2, 0→0.25% EtOAc/*n*-hexane).

R_f (10% EtOAc/*n*-hexane) = 0.46; **^{1}H NMR** (400 MHz, $CDCl_3$) δ = 7.46 – 7.42 (m, 2H, H13), 7.33 – 7.24 (m, 3H, H12 and H14), 7.09 – 7.02 (m, 2H, H3), 6.95 – 6.89 (m, 2H, H2), 6.21 (d, *J* = 11.5 Hz, 1H, H5), 5.80 (dd, *J* = 12.6, 11.5 Hz, 1H, H6), 2.47 (d, *J* = 12.6 Hz, 1H, H7), 1.20 (s, 6H, H9), 1.17 (s, 6H, H9), 0.33 (m, 6H, H10) ppm; **^{13}C NMR** (101 MHz, $CDCl_3$) δ = 161.1 (d, J_{CF} = 245 Hz, C1), 137.8 (C11), 134.2 (d, J_{CF} = 3 Hz, C4), 134.0 (C13), 130.1 (d, J_{CF} = 8 Hz, C3), 129.4 (C6), 129.2 (C14), 127.7 (C12), 124.9 (C5), 115.0 (d, J_{CF} = 22 Hz, C2), 83.3 (C8), 25.0 (C9), -2.7 (C10), -3.0 (C10) ppm; **^{11}B NMR** (128 MHz, $CDCl_3$) δ = 33.29 ppm; **^{19}F NMR** (376 MHz, $CDCl_3$) δ = 116.7 (m) ppm; **IR** (ATR): ῦ = 3072, 2980, 2931, 1704, 1632, 1603, 1591, 1509, 1469, 1428, 1412, 1391, 1372, 1312, 1249, 1221, 1191, 1141, 1113, 1013, 968, 901, 835, 816, 773, 732, 698, 661 cm^{-1}; **HMRS** (EI) calcd. for $C_{23}H_{30}BFO_2Si$ $[M]^+$ 396.2092, found 396.2134.

(*Z*)-(1-(Dimethyl(phenyl)silyl)-3-phenylallyl)boronic acid, pinacol ester (33)

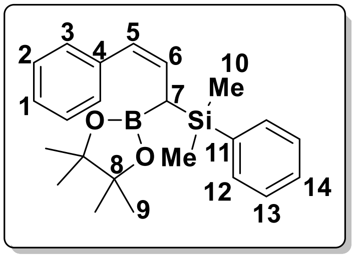

Prepared according to General Procedure **G**, **1a** (80.4 mg, 0.2 mmol), and styrene (69 µL, 0.6 mmol) were converted to **33** yielding a yellow oil (65% NMR yield, >95:5 (*Z*:*E*), 47 mg, 93:7 (*Z*:*E*) 62%), after purification by flash column chromatography (SiO_2, 0→0.25% EtOAc/*n*-hexane).

R_f (10% EtOAc/*n*-hexane) = 0.52; **^{1}H NMR** (400 MHz, $CDCl_3$) δ = 7.48 – 7.45 (m, 2H), 7.31 – 7.22 (m, 5H), 7.15 – 7.13 (m, 3H), 6.27 (d, *J* = 11.5 Hz, 1H, H5), 5.84 – 5.78 (m, 1H, H6), 2.56 (d, *J* = 12.6 Hz, 1H, H7), 1.18 (s, 6H, H9), 1.15 (s, 6H, H9), 0.33 (m, 6H, H10) ppm; **^{13}C NMR** (101 MHz, $CDCl_3$) δ = 138.2 (C4), 138.0 (C11), 134.1, 129.5 (C6), 129.1, 128.7, 128.2, 127.6, 126.1 (C5), 126.0, 83.2 (C8), 25.0 (C9), -2.7 (C10), -2.9 (C10) ppm; **^{11}B NMR** (128 MHz, $CDCl_3$) δ = 32.78 ppm; **IR** (ATR): ῦ = 3054, 2980, 2932, 1628, 1601, 1576, 1493, 1481, 1469, 1447, 1428, 1405, 1390, 1372, 1312, 1249, 1214, 1191, 1166, 1141, 1113, 1074, 1005, 969, 902, 850, 834, 811, 780, 730, 697, 664 cm^{-1}; **HRMS** (EI) calcd. for $C_{23}H_{31}BO_2Si$ $[M]^+$ 378.2186, found 378.2230.

(*Z*)-(3-(Benzofuran-5-yl)-1-(dimethyl(phenyl)silyl)allyl)boronic acid, pinacol ester (34)

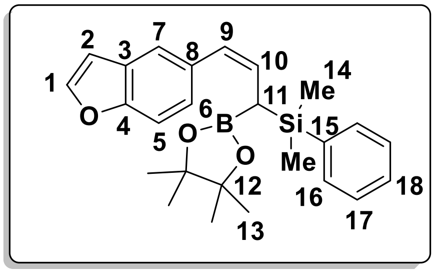

Prepared according to General Procedure **G**, **1a** (80.4 mg, 0.2 mmol), and **S5** (86.5 mg, 0.6 mmol) were converted to **34** yielding a yellow oil (82% NMR yield, >95:5 (*Z*:*E*), 58.6 mg, 70%), after purification by flash column chromatography (C18, 20→100% $MeCN/H_2O$).

R*f* (5% EtOAc/*n*-hexane) = 0.29; **^{1}H NMR** (400 MHz, $CDCl_3$) δ = 7.58 (d, *J* = 2.2 Hz, 1H, H1), 7.48 – 7.44 (m, 2H, H16), 7.36 (d, *J* = 8.4 Hz, 1H, H5), 7.31 – 7.23 (m, 4H, H7, H17 and H18), 7.06 (dd, *J* = 8.5, 1.8 Hz, 1H, H6), 6.68 (dd, *J* = 2.2, 1.0 Hz, 1H, H2), 6.37 (d, *J* = 11.5 Hz, 1H, H9), 5.81 (dd, *J* = 12.5, 11.4 Hz, 1H, H10), 2.57 (d, *J* = 12.9 Hz, 1H, H11), 1.20 (s, 6H, H13), 1.18 (s, 6H, H13), 0.34 (s, 3H, H14), 0.33 (s, 3H, H14) ppm; **^{13}C NMR** (101 MHz, $CDCl_3$) δ = 153.5 (C4), 145.1 (C1), 138.1 (C15), 134.1 (C17), 133.0 (C8), 129.1 (C18), 128.6 (C10), 127.6 (C16), 127.4 (C3), 126.2 (C9), 125.5 (C6), 120.8 (C7), 110.9 (C5), 106.8 (C2), 83.2 (C12), 25.1 (C13), 25.0 (C13), -2.6 (C14), -3.0 (C14) ppm; **^{11}B NMR** (128 MHz, $CDCl_3$) δ = 32.80 ppm; **IR** (ATR): ṽ = 3071, 2979, 2250, 1628, 1590, 1539, 1468, 1440, 1428, 1391, 1372, 1312, 1249, 1214, 1196, 1166, 1141, 1125, 1111, 1032, 1006, 969, 909, 882, 850, 815, 765, 732, 699, 665 cm^{-1}; **HMRS** (EI) cacld. for $C_{25}H_{31}BO_3Si$ $[M]^+$ 418.2136, found 418.2056.

((*Z*)-1-(Dimethyl(phenyl)silyl)-3-((8R,9S,13S,14S)-13-methyl-17-oxo-7,8,9,11,12,13,14,15,16,17-decahydro-6H-cyclopenta[a]phenanthren-3-yl)allyl)boronic acid, pinacol ester (35)

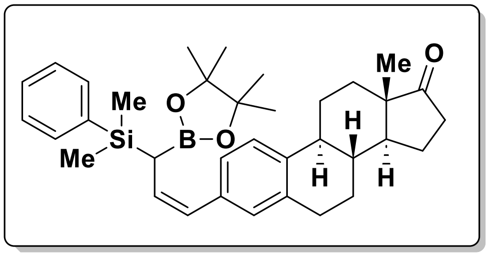

Prepared according to General Procedure **G**, **1a** (80.4 mg, 0.2 mmol), and **S9** (168 mg, 0.6 mmol) were converted to **35** yielding a yellow oil (64% NMR yield, 90:10 (*Z*:*E*), 62.1 mg, 93:7 (*Z*:*E*) 55%), after purification by flash column chromatography (SiO_2, 0→10% EtOAc/*n*-hexane).

R*f* (10% EtOAc/*n*-hexane) = 0.19; **^{1}H NMR** (400 MHz, $CDCl_3$) δ = 7.54 – 7.49 (m, 2H), 7.33 – 7.28 (m, 3H), 7.17 (dd, *J* = 8.2, 3.4 Hz, 1H), 7.00 – 6.96 (m, 1H), 6.88 – 6.81 (m, 1H), 6.22 (dd, *J* = 11.4, 2.0 Hz, 1H), 5.79 (dd, *J* = 12.5, 11.5 Hz, 1H), 2.84 – 2.80 (m, 2H), 2.59 – 2.47 (m, 2H), 2.45 – 2.39 (m, 1H), 2.34 – 2.24 (m, 1H), 2.21 – 1.92 (m, 4H), 1.71 – 1.37 (m, 6H), 1.18, (s, 6H), 1.17 (s, 6H), 0.92 (d, *J* = 3.2 Hz, 3H), 0.38 (s, 3H), 0.37 (s, 3H) ppm; **^{13}C NMR** (101 MHz, $CDCl_3$, mixture of diastereomers) δ = 221.2, 138.1, 137.5, 136.0, 135.8, 134.2, 134.1, 129.2, 129.2, 129.1, 129.0, 127.6, 126.1, 126.1, 125.8, 125.8, 125.1, 125.1, 83.2, 50.6, 48.2, 44.5, 44.5, 38.3, 38.3, 36.0, 31.7, 29.5, 26.7, 25.8, 25.8, 25.1, 25.0, 21.7, 14.0, 14.0, -2.5, -2.8, -2.8 ppm; **^{11}B NMR** (128 MHz, $CDCl_3$) δ = 33.36 ppm; **IR** (ATR): ṽ = 2979, 2931, 1739, 1627, 1499, 1455, 1428, 1408, 1391, 1372, 1313, 1250, 1216, 1166, 114z1, 1113, 1084, 1053, 1007, 968, 896, 850, 835, 818, 753, 734, 700, 667 cm^{-1}; **HRMS** (EI) calcd. for $C_{35}H_{47}BO_3Si$ $[M]^+$ 554.3388, found 554.3401.

Derivatization of Products

(*E*)-(4-Methoxy-3-(*p*-tolyl)pent-1-en-1-yl)boronic acid, pinacol ester (36)

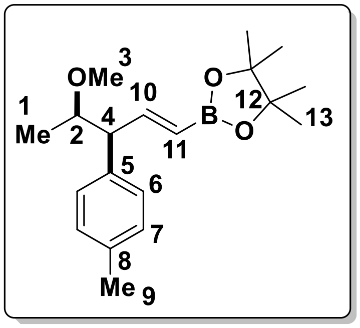

Prepared according to a literature procedure of Gevorgyan *et al.*[2] To an oven-dried microwave vial was added **3** (47.3 mg, 0.1 mmol, 1.0 equiv.). The tube was sealed and purged with argon before the addition of 1,1-dimethoxyethane (13 μL, 1.03 mmol, 1.03 equiv.) and anhydrous DCM (0.5 mL, 0.2 M). The reaction mixture was cooled to -78 ºC, followed by the addition of $TiCl_4$ (120 μL, 1 M DCM, 1.2 equiv.). After 15 minutes, the mixture was warmed to ambient temperature, diluted with DCM (10 mL) and filtered through a layer of Celite. The filtrate was concentrated *in vacuo*. The crude residue was purified by flash column chromatography (SiO_2, 0→20% EtOAc/*n*-hexane) to yield **36** as a colourless oil (18.9 mg, 60%, *d.r.* > 20:1).

R_f (10% EtOAc/*n*-hexane) = 0.35; **^{1}H NMR** (400 MHz, $CDCl_3$) δ = 7.14 – 7.08 (m, 4H, H6 and H7), 6.78 (dd, *J* = 17.9, 8.2 Hz, 1H, H10), 5.46 (d, *J* = 1.2 Hz, 1H, H11), 3.66 – 3.58 (m, 1H, H2), 3.39 (t, *J* = 7.2 Hz, 1H, H4), 3.24 (s, 3H, H3), 2.31 (s, 3H, H9), 1.24 (s, 12H, H13), 1.15 (d, *J* = 6.2 Hz, 3H, H1) ppm; **^{13}C NMR** (101 MHz, $CDCl_3$) δ = 153.6 (C10), 138.2 (C5), 136.0 (C8), 129.2 (C7), 128.6 (C6), 83.2 (C12), 79.5 (C2), 58.3 (C4), 56.8 (C3), 24.9 (C13), 21.2 (C9), 17.3 (C1) ppm; **^{11}B NMR** (128 MHz, $CDCl_3$) δ = 29.96 ppm; **IR** (ATR): ṽ = 2980, 2930, 2824, 1729, 1635, 1515, 1459, 1391, 1361, 1321, 1259, 1215, 1188, 1166, 1144, 1112, 997, 970, 926, 901, 851, 812, 756, 722, 666 cm^{-1}; **HRMS** (ESI) cacld. for $C_{19}H_{29}BO_3Na^+$ $[M+Na]^+$ 339.2102, found 339.2132. The stereochemical outcome was rationalized by Hayashi and Kumada's report on the Sakurai reaction of (E)-cinnamyltrimethylsilane (3a) with aldehydes under the same reactions conditions.[28]

(*E*)-4-(Dimethyl(phenyl)silyl)-1-(4-nitrophenyl)-2-(*p*-tolyl)but-3-en-1-ol (37)

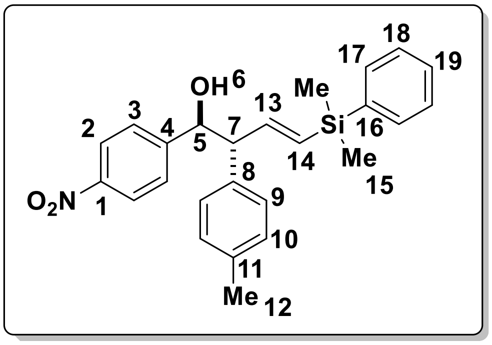

To an oven-dried microwave vial was added **3** (47.3 mg, 0.1 mmol, 1.0 equiv.) and 4-nitrobenzaldehyde (15.1 mg, 0.1 mmol, 1 equiv.). The tube was sealed and purged with argon before the addition of anhydrous MeCN (0.5 mL, 0.2 M). The reaction mixture was heated to 50 ºC and stirred overnight. The reaction mixture was gradually cooled to ambient temperature and the solvent was removed *in vacuo*. The crude residue was purified by flash column chromatography (SiO_2, 0→20% EtOAc/*n*-hexane) to yield **37** as a pale yellow oil (28.2 mg, 68%, *d.r.* = 15:1).

R_f (10% EtOAc/*n*-hexane) = 0.36; **^{1}H NMR** (400 MHz, $CDCl_3$) δ = 7.97 – 7.93 (m, 2H, H2), 7.56 – 7.49 (m, 2H, H18), 7.46 – 7.34 (m, 3H, H17 and H19), 7.01 – 6.92 (m, 4H, H3 and H10), 6.80 (dd, *J* = 13.9, 10.7 Hz, 1H, H13), 6.66 (d, *J* = 8.1 Hz, 2H, H9), 6.02 (d, *J* = 13.9 Hz, 1H, H14), 4.74 (d, *J* = 8.0 Hz, 1H, H5), 3.46 (dd, *J* = 10.7, 8.1 Hz, 1H, H7), 2.27 (s, 3H, H12), 0.41 (s, 3H, H15), 0.35 (s, 3H, H15) ppm; **^{13}C NMR** (101 MHz, $CDCl_3$) δ = 149.2 (C4), 147.4 (C13), 147.1 (C1), 139.0 (C16), 136.7 (C11), 136.5 (C8), 134.1 (C17), 133.3 (C14), 129.5 (C19), 129.4 (C10), 128.3 (C18), 127.9 (C9), 127.5 (C3), 123.1 (C2), 77.0 (C5), 57.3 (C7), 21.2 (C12), -0.8 (C15), -1.2 (C15) ppm; **IR** (ATR): ṽ = 3556, 3070, 3022, 2956, 2925, 1605, 1516, 1429, 1345, 1249, 1183, 1111, 1057, 1015, 999, 853, 820, 787, 755, 731, 700 cm^{-1}; **HRMS** (ESI) cacld. for $C_{25}H_{27}NO_3SiNa^+$ $[M+Na]^+$ 440.1652 , found 440.1672.

(*E*)-(4-Hydroxy-4-(4-nitrophenyl)-3-(*p*-tolyl)but-1-en-1-yl)boronic acid, pinacol ester (38)

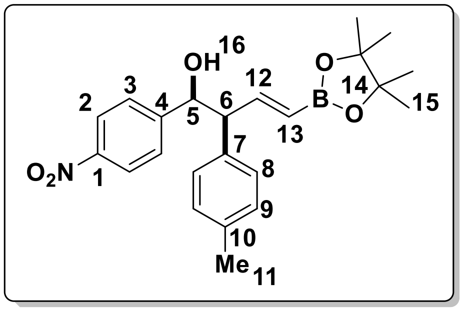

To an oven-dried 5 mL microwave vial, Ir(*p*-CF_3)$_3$ (3.4 mg, 1 mol%) and **1c** (158 mg, 0.4 mmol, 1 equiv.) were added. The vial was sealed with a septum and purged with nitrogen before the sequential addition of degassed MeCN (8 mL), 2,6-lutidine (70 μL, 0.6 mmol, 1.5 equiv.) and 4-methylstyrene (158 μL, 1.2 mmol, 3 equiv.) via syringe. The reaction mixture was stirred under light irradiation (440 nm, PR-160L) for 16 h. After completion, the reaction mixture was washed with sat. aq. NH_4Cl (10 mL). Organics were extracted with EtOAc (3 x 10 mL). The combined organic phase was dried over Na_2SO_4 and concentrated under reduced pressure. To an oven-dried microwave vial was added crude **28** and 4-nitrobenzaldehyde (39.3 mg, 0.26 mmol, 1 equiv.). The tube was sealed and purged with argon before the addition of anhydrous MeCN (1.3 mL, 0.2 M). The reaction mixture was heated to 50 ºC and stirred overnight. The reaction mixture was gradually cooled to ambient temperature and the solvent was removed *in vacuo*. The crude residue was purified by flash column chromatography (SiO_2, 0→20% EtOAc/*n*-hexane) to yield **38** containing a 10% impurity from starting **1c** material as a pale yellow oil (51.3 mg, 31% over two-steps, *d.r.* >20:1).

R*f* (20% EtOAc/*n*-hexane) = 0.18; **^{1}H NMR** (400 MHz, $CDCl_3$) δ = 8.14 (d, *J* = 8.7 Hz, 2H, H2), 7.43 (d, *J* = 8.7 Hz, 2H, H3), 7.12 (q, *J* = 8.2 Hz, 4H, H8 and H9), 6.68 (dd, *J* = 17.9, 7.9 Hz, 1H, H12), 5.28 (dd, *J* = 17.9, 1.2 Hz, 1H, H13), 5.05 (d, *J* = 7.6 Hz, 1H, H5), 3.57 (t, *J* = 1.2 Hz, 1H, H6), 2.33 (s, 3H, H11), 1.20 (s, 12H, H15) ppm; **^{13}C NMR** (100 MHz, CDCl3) δ = 151.2 (C12), 149.5 (C4), 147.5 (C1), 137.4 (C7), 135.3 (C10), 129.7 (ArC-H), 128.9 (ArC-H), 127.8 (C3), 123.3 (C2), 83.4 (C14), 76.3 (C5), 60.4 (C6), 24.8 (C15), 21.2 (C11) ppm; **^{11}B NMR** (128 MHz, $CDCl_3$) δ = 30.05 ppm; **IR** (ATR): ῦ = 3397, 2980, 2926, 1634, 1608, 1513, 1390, 1344, 1324, 1255, 1215, 1166, 1140, 1108, 1082, 1039, 993, 968, 907, 847, 807, 752, 732, 701, 666, 646 cm^{-1}; **HRMS** (ESI) calcd. For $C_{23}H_{28}BNO_5Na^+$ $[M+Na]^+$ 432.1953, found 432.1976.

(3-([1,1'-Biphenyl]-4-yl)-1-(dimethyl(phenyl)silyl)-3-oxopropyl)boronic acid, pinacol ester (39)

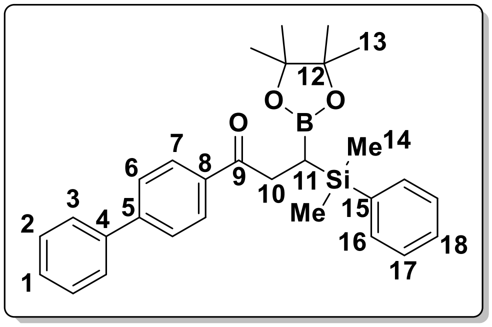

To an oven-dried microwave vial was added $Pd(OAc)_2$ (1.1 mg, 0.005 mmol, 0.05 equiv.), SPhos (4.1 mg, 0.01 mmol, 0.1 equiv.), phenylboronic acid (18.3 mg, 0.15 mmol, 1.5 equiv.), **11** (47.3 mg, 0.1 mmol, 1.0 equiv.), K_3PO_4 (63.7 mg, 0.3 mmol, 3 equiv.). The tube was sealed and purged with argon before the addition of 1,4-dioxane (0.4 mL, 0.25 M) and H_2O (9 μL, 1.25 mmol, 5 equiv.). The reaction mixture was stirred at 80 ºC overnight. After the reaction was complete, the tube was gradually cooled to ambient temperature and the reaction mixture was diluted with EtOAc (5 mL) and filtered through a layer of Celite, eluting the product with EtOAc (2 x 10 mL). The filtrate was washed with H_2O (25 mL), brine (25 mL), dried over $MgSO_4$, and concentrated *in vacuo*. The crude residue was purified by flash column chromatography (SiO_2, 0→5% EtOAc/*n*-hexane) to yield **39** as a colourless oil (26.3 mg, 56%).

R*f* (10% EtOAc/*n*-hexane) = 0.48; **^{1}H NMR** (400 MHz, $CDCl_3$) δ = 7.96 – 7.92 (m, 2H), 7.64 – 7.56 (m, 6H), 7.49 – 7.43 (m, 2H), 7.41 – 7.35 (m, 4H), 3.23 (dd, *J* = 18.3, 12.2 Hz, 1H, H10), 2.98 (dd, *J* = 18.3, 3.5 Hz, 1H, H10), 1.26 (s, 6H, H13), 1.17 (s, 6H, H13), 1.11 (dd, *J* = 12.2, 3.5 Hz, 1H, H11), 0.42 (s, 3H, H14), 0.40 (s, 3H, H14) ppm; **^{13}C NMR** (101 MHz, $CDCl_3$) δ = 200.0 (C9), 145.4 (C5), 140.2 (C4), 138.4

(C15), 135.8 (C8), 133.9 (ArC-H), 129.2 (ArC-H), 129.0 (ArC-H), 128.7 (ArC-H), 128.2 (ArC-H), 128.0, 127.4 (ArC-H), 127.2 (ArC-H), 83.1 (C12), 36.3 (C10), 25.1 (C13), 24.8 (C13), -2.0 (C14), -3.4 (C14) ppm; ^{11}**B NMR** (128 MHz, $CDCl_3$) δ = 34.18 ppm; **IR** (ATR): ṽ = 3070, 2978, 2930, 1684, 1605, 1561, 1516, 1487, 1450, 1428, 1405, 1379, 1372, 1352, 1305, 1249, 1216, 1191, 1142, 1112, 1075, 1026, 1008, 998, 981, 970, 912, 879, 853, 831, 815, 763, 731, 698, 675, 664 cm^{-1}; **HRMS** (EI) cacld. for $C_{29}H_{35}BO_3Si$ $[M]^+$ 470.2449, found 470.2466.

2-(1,3-Dimethyl-2-oxoindolin-3-yl)acetaldehyde (40)

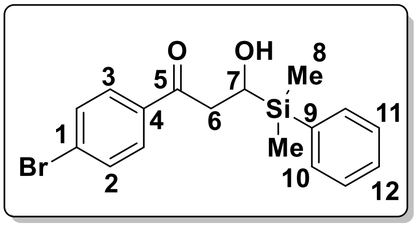

To a solution of **11** (23.7 mg, 0.05 mmol, 1 equiv.) in THF (250 µl) and H_2O (250 µl, KH_2PO_4-NaOH buffer pH 7) kept at 0°C was added $NaBO_3 \cdot H_2O$ (15 mg, 0.15 mmol, 3 equiv.). The reaction was stirred for 1 h, before being gradually warmed to ambient temperature and stirred overnight. The reaction mixture was washed with sat. aq. $Na_2S_2O_3$ (4 mL) and organic phase extracted (EtOAc 3 x 4 mL). The combined organic phases were dried over Na_2SO_4, filtered and the solvent removed under reduced pressure. The crude was purified by flash column chromatography (SiO_2, DCM) to yield **40** as a colourless oil (16.7 mg, 92%).

***R**$_f$* (DCM) = 0.50; ^{1}**H NMR** (400 MHz, $CDCl_3$): δ = 7.73 (dd, *J* = 6.4, 2.1 Hz, 2H), 7.61 – 7.56 (m, 4H), 7.43 – 7.37 (m, 3H), 4.19 (dd, *J* = 11.0, 2.3 Hz, 1H, H7), 3.10 (dd, *J* = 17.7, 11.0 Hz, 1H, H6), 2.97 (dd, *J* = 17.7, 2.3 Hz, 1H, H6), 0.42 (s, 6H, H8) ppm; ^{13}**C NMR** (151 MHz, $CDCl_3$) δ = 200.5 (C5), 136.3, 135.6, 134.2, 132.1, 130.5, 129.7, 128.8, 128.2, 60.7 (C7), 41.1 (C6), -5.1 (C8), -5.7 (C8) ppm; **IR** (ATR); ṽ = 2955, 2926, 1677, 1585, 1568, 1484, 1427, 1395, 1352, 1249, 1206, 1177, 1143, 1111, 1071, 1008, 982, 876, 810, 772, 732, 701, 623 cm^{-1}; **HRMS** (ESI) calcd. for $C_{17}H_{18}BrO_2Si^-$ $[M-H^+]^-$ 361.0265, found 361.0282.

3-(2,2-Difluoroethyl)-5-fluoro-1,3-dimethylindolin-2-one (41)

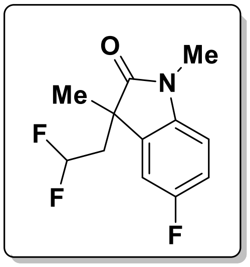

Prepared according to a literature procedure of Li *et al.*[29] To an oven-dried microwave vial was added **24** (44.2 mg, 0.1 mmol, 1 equiv.), $AgNO_3$ (3.4 mg, 0.02 mmol, 0.2 equiv.), and Selectfluor (106 mg, 0.3 mmol, 3 equiv.). The tube was sealed and purged with argon before the addition of degassed DCM (0.3 mL), degassed H_2O (0.23 mL), TFA (0.2 mL), and H_3PO_4 (0.05 mL) at ambient temperature. The reaction mixture was heated to 50 ºC and stirred for 16 h. The reaction mixture was gradually cooled to ambient temperature and extracted with DCM (3 x 10 mL). Combined organics were washed with brine, dried over Na_2SO_4, and concentrated *in vacuo*. The crude was purified by flash column chromatography (SiO_2, 0→20% EtOAc/*n*-hexane) to give yield **41** as a colourless oil (9.6 mg, 39%).

^{1}**H NMR** (600 MHz, $CDCl_3$) δ = 7.04 – 6.96 (m, 2H), 6.80 (dd, *J* = 8.5, 4.1 Hz, 1H), 5.62 (tdd, *J* = 55.9, 6.1, 3.6 Hz, 1H), 3.22 (s, 3H), 2.50 (qd, *J* = 14.5, 6.1 Hz, 1H), 2.32 – 2.22 (m, 1H), 1.41 (s, 3H) ppm; ^{13}**C NMR** (151 MHz, $CDCl_3$) δ = 178.9, 159.5 (d, $^1J_{C-F}$ = 241.4 Hz), 138.9, 133.8 (d, $^3J_{C-F}$ = 7.7 Hz), 115.0 (t, $^1J_{C-F}$ = 240 Hz), 114.9 (d, $^2J_{C-F}$ = 23.4 Hz), 111.3 (d, $^2J_{C-F}$ = 24.8 Hz), 109.1 (d, $^3J_{C-F}$ = 8.2 Hz), 45.2, 41.3 (t, $^2J_{C-F}$ = 21.9 Hz), 26.7, 24.5 ppm; ^{19}**F NMR** (376 MHz, $CDCl_3$) δ = -114.16 – -114.34 (m), -120.10 ppm; analytical data in agreement with literature.[30]

2-(1,3-Dimethyl-2-oxoindolin-3-yl)acetaldehyde (42)

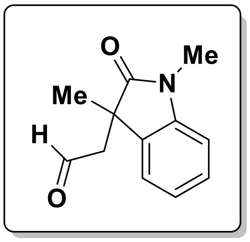

To a solution of **24** (22.1 mg, 0.05 mmol, 1 equiv.) in THF (250 µl) and H_2O (250 µl, KH_2PO_4-NaOH buffer pH 7) kept at 0°C was added $NaBO_3 \cdot H_2O$ (13 mg, 0.13 mmol, 2.6 equiv.). The reaction was stirred for 1 h, before being gradually warmed to ambient temperature and stirred overnight. The reaction mixture was washed with sat. aq. $Na_2S_2O_3$ (4 mL) and organic phase extracted (EtOAc 3 x 4 mL). The combined organic phases were dried over Na_2SO_4, filtered and the solvent removed under reduced pressure. The crude was purified by flash column chromatography (SiO_2, EtOAc/*n*-hexane 50%) to yield **42** as a colourless oil (9 mg, 89%).

^{1}H NMR (600 MHz, $CDCl_3$) δ = 9.52 (s, 1H), 7.29 (t, *J* = 7.7 Hz, 1H), 7.18 (d, *J* = 7.4 Hz, 1H), 7.05 (t, *J* = 7.5 Hz, 1H), 6.88 (d, *J* = 7.8 Hz, 1H), 3.27 (s, 3H), 3.02 – 2.93 (m, 2H), 1.42 (s, 3H) ppm; **^{13}C NMR** (151 MHz, $CDCl_3$) δ = 198.9, 179.7, 143.3, 132.9, 128.5, 122.8, 122.6, 108.5, 50.7, 45.1, 26.6, 24.1 ppm; analytical data in agreement with literature.[31]

Computational Investigation

1. Computational Details

All of the calculations were carried out using density functional theory (DFT) calculations in the Gaussian 16[32] program package. Optimizations were performed using the ωB97xD functional[33] with the Def2SVP[34] basis set for all the atoms. All the stationary points were characterized by frequency calculations. Additional single point energy calculations at ωB97xD/Def2TZVPP were carried out to refine the potential energies. Final enthalpies and free energies were calculated by adding the thermodynamic corrections calculated at low-theory level to the single point energies at high-theory level. Also, the standard state was corrected from 1atm to 1M by adding 1.89 kcal/mol. Solvent was considered implicitly for both optimizations/frequencies and single point calculations, using the SMD implicit solvation model and acetonitrile as the solvent.[35] TD-DFT calculations were carried out when needed using the optimized structures and the ωB97xD/Def2TZVPP level of theory, including the first 50 singlet excited states (NStates=50).

NBO calculations were performed with nbo (version 3) program.[36] Quantitative analysis of the Electrostatic potentential surface was carried out with MultiWFN program package.[37]

Some of the 3D images calculated structures were prepared using CYLview.[38]

2. DFT Method Benchmarking

1a

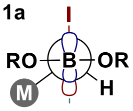

M = $SiMe_2Ph$	RSE	BDE	ΔG^{o}_{Add}
wB97xD/Def2TZVPP	6.1	57.6	6.0
B3LYP(D3BJ)/Def2TZVPP	7.1	54.6	5.7
PBE0(D3BJ)/Def2TZVPP	6.1	59.9	5.4
M062X/Def2TZVPP	6.4	52.6	6.7
CBS-4M	5.9	-	-

1b

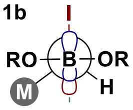

M = $GeEt_3$	RSE	BDE	ΔG^{o}_{Add}
wB97xD/Def2TZVPP	6.0	57.2	5.3
B3LYP(D3BJ)/Def2TZVPP	6.8	54.6	5.1
PBE0(D3BJ)/Def2TZVPP	6.3	59.1	4.7
M062X/Def2TZVPP	6.7	52.2	5.8
CBS-4M	4.6	-	-

1c

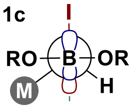

M = BPin	RSE	BDE	ΔG^{o}_{Add}
wB97xD/Def2TZVPP	7.6	55.3	6.2
B3LYP(D3BJ)/Def2TZVPP	8.5	52.0	5.8
PBE0(D3BJ)/Def2TZVPP	7.6	57.1	5.5
M062X/Def2TZVPP	8.3	50.1	6.5
CBS-4M	6.6	-	-

1d

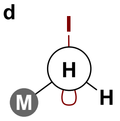

M = $SiMe_2Ph$	RSE	BDE	ΔG^{o}_{Add}
wB97xD/Def2TZVPP	3.5	60.5	6.6
B3LYP(D3BJ)/Def2TZVPP	3.9	57.9	6.4
PBE0(D3BJ)/Def2TZVPP	3.5	62.5	6.1
M062X/Def2TZVPP	4.0	55.5	7.2
CBS-4M	3.1	-	-

1e

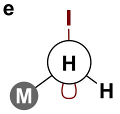

M = $GeEt_3$	RSE	BDE	ΔG^{o}_{Add}
wB97xD/Def2TZVPP	3.5	61.0	5.4
B3LYP(D3BJ)/Def2TZVPP	3.8	58.6	5.3
PBE0(D3BJ)/Def2TZVPP	3.5	62.7	5.0
M062X/Def2TZVPP	3.9	56.0	5.7
CBS-4M	2.5	-	-

Figure S30. Comparison of Radical Stabilization Energy (RSE), Bond Dissociation Enthalpy (BDE), and substrate-lutidine adduct formation energy (ΔG^{o}_{Add}). All the energies are in kcal/mol.

The relative energies calculated with the four different methods showed minor deviations. The absolute BDE showed more deviation but the relative bond strength was not affected by the method (Si and Ge showed a ~2kcal/mol stronger C-I bond than B substituted). We selected the ωB97xD method to minimize errors between optimized structures (small basis-set) and SP calculation (large basis set), and due to the level of accuracy for BDE calculations, which were close to the DLPNO-CCSD(T) method and RSE43 benchmark dataset.[39,40]

3. Relaxed Potential Energy Scan for C-I Bond Breaking in the Triplet State

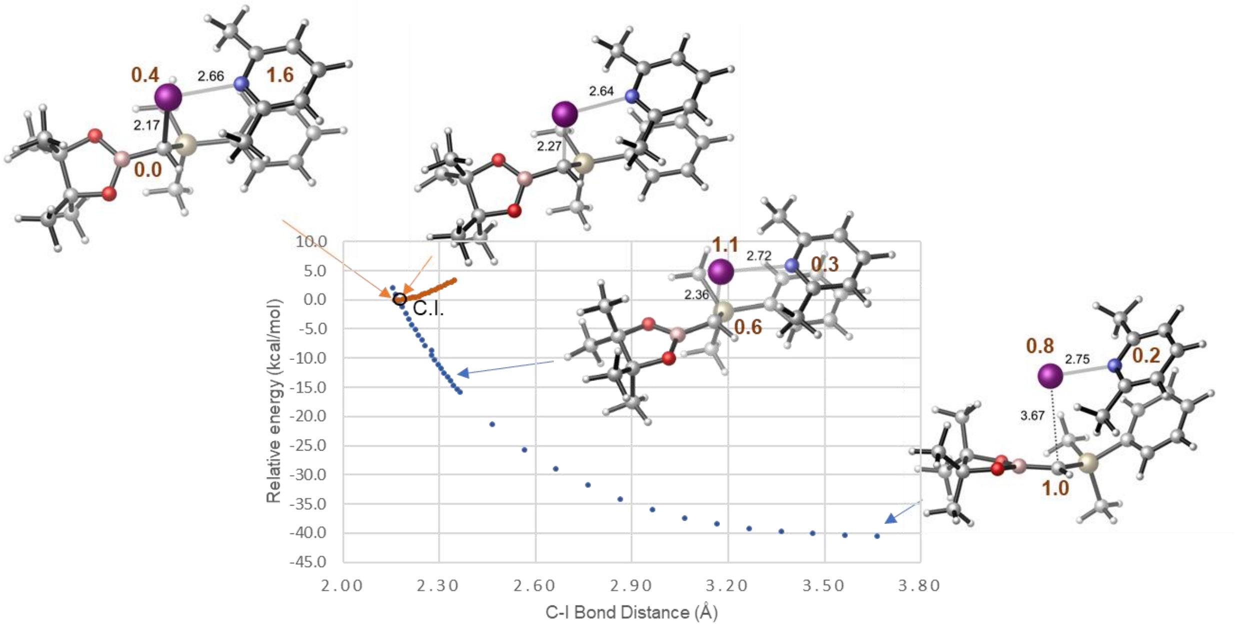

Figure S31. Relaxed potential energy scan of the C-I coordinate in the triplet state electronic structures, including the crossing point (approximate conical intersection). Energy referred to **1a-lut** adduct in the triplet state (kcal/mol). Spin densities are shown in brown.

4. Relaxed Potential Energy Scan for N-B Coordination

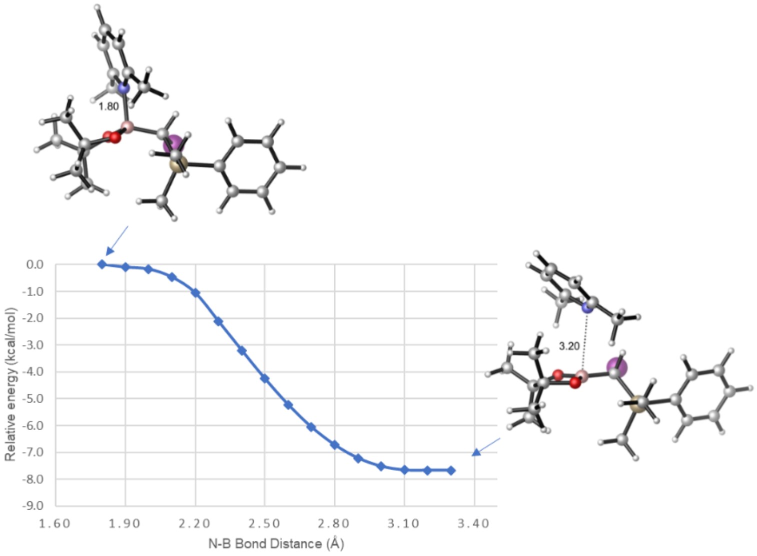

Figure S32. Relaxed potential energy scan of the **1a-lut** adduct through N-B coordination. Energy referred to the first point (1.80 Å).

5. HOMO and LUMO Orbitals of 1a

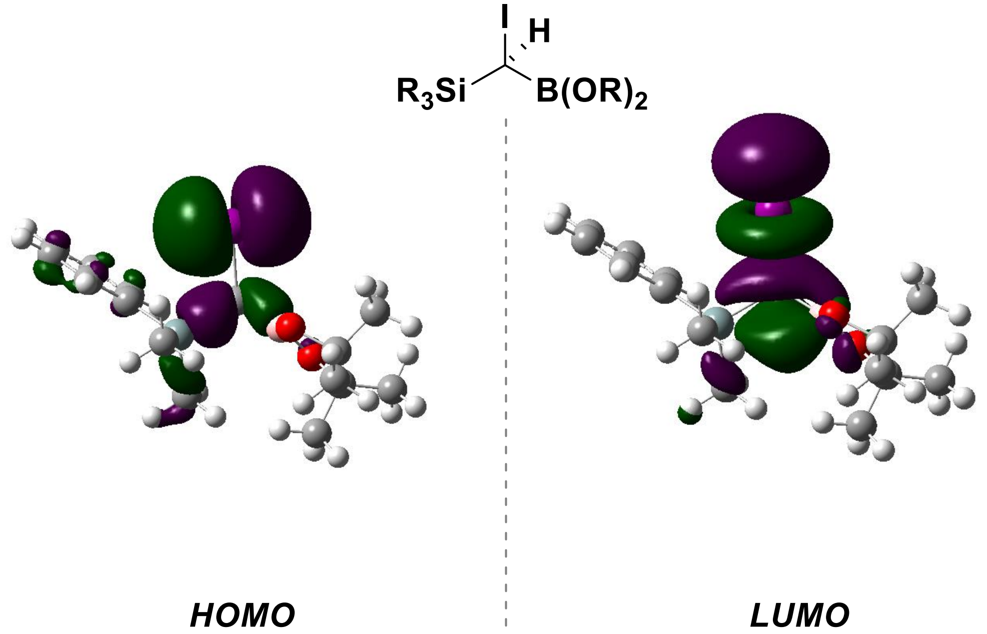

Figure S33. 3D representation of the HOMO and LUMO orbitals of **1a**.

6. Calculated UV-VIS Spectra of 1a and 1a-lut Adduct

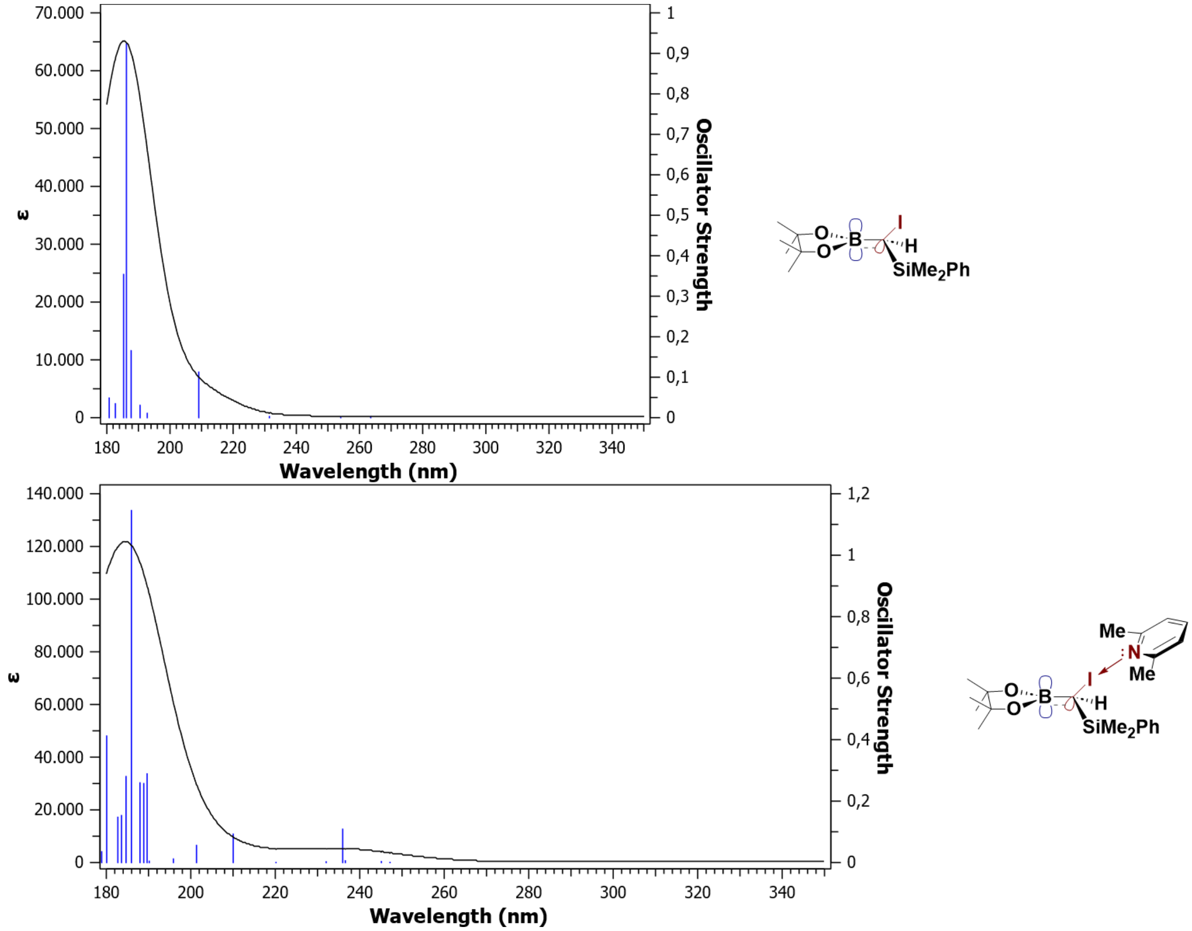

Figure S34. Calculated UV-VIS spectra (50 singlet excited states) using TD-DFT of **1a** (top) and **1a-lut** (bottom).

The excitation associated with the small peak around 230 nm in the **1a-lut** adduct is defined by the following orbital transitions, showing the clear impact of adduct in the bathochromic shift of the absorption.

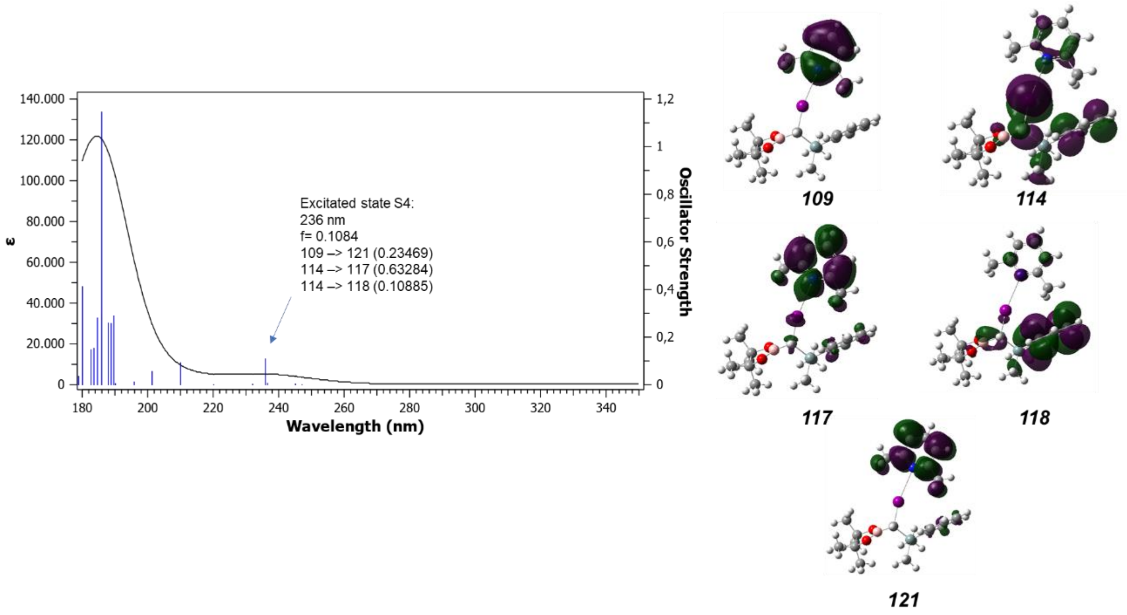

Figure S35. Orbitals involved in the red-shifted absorption band of **1a-lut** adduct.

7. Characterization of sigma hole in 1a and N-I donation (nbo analysis)

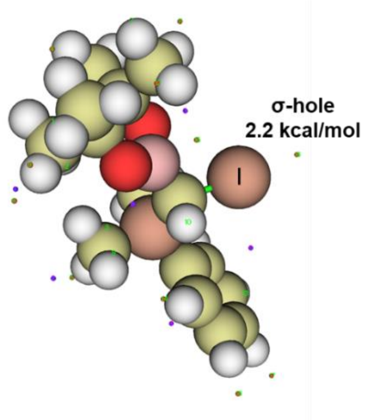

Figure S36. Quantitative analysis of the σ-hole in the electrostatic potential surface of **1a**.

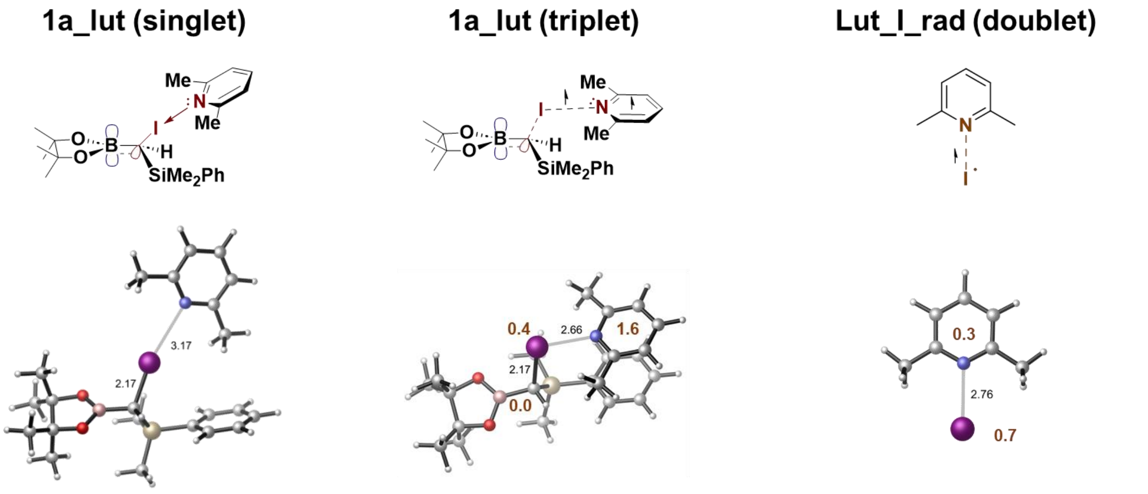

NBO analysis:	NBO analysis:	NBO analysis:
Donor-Aceptor NBO pairs (kcal/mol):	Donor-Aceptor NBO pairs (kcal/mol):	Donor-Aceptor NBO pairs (kcal/mol):
N Lone-pair → C-I σ* (5.54)	α-electrons:	α-electrons:
	None above 1kcal/mol	None above 1kcal/mol
	β-electrons:	β-electrons:
	There is a σ-bond between I-N centers	N Lone-pair → Lone-pair*(empty) I (53.37)

Figure S37. Second order perturbation analysis of NBO electronic structure distribution. Energies in kcal/mol (above 1 kcal/mol threshold), bond lengths in Å, spin densities shown in brown.

The NBO analysis of the acceptor-donor orbital pairs shows a clear σ-hole interaction in **1a_lut** adduct, with an interaction of 5.54 kcal/mol, further supporting the σ-hole characterization by electrostatic potential surface maxima-minima analysis (Figure S36). In the triplet adduct, the NBO analysis detect a bonding interaction between N and I centers beta-electron, suggesting an actual bond between those fragments, and further explaining the small distance observed (2.66 Å). Finally, once the iodine radical is formed, there is a clear stabilization by β-electron donation of the lone-pair of N in lutidine to the empty β lone pair (SOMO) of iodine radical (53.37 kcal/mol).

8. Free energy profile for product formation from C-radical intermediate

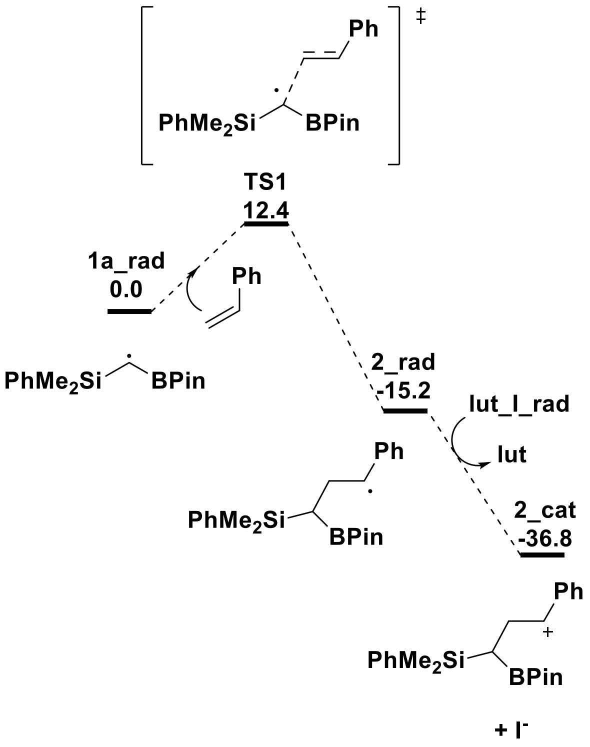

Figure S38. Free energy profile of product formation after C-I bond activation. Free energies in kcal/mol.

9. XYZ Coordinates and final energies of the calculated species

Final free energies of the main text are calculated as the sum of E (wB97xD/def2TZVPP) + G_{Corr} and final enthalpies as the sum of E (wB97xD/def2TZVPP) + H_{Corr}

·I_{rad}

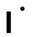

E (ωB97xD) = -297.7619585
E (B3LYP-D3(BJ)) = -297.7806623
E (PBE0-D3(BJ)) = -297.6953301
E (M062X) = -297.6142761
H_{Corr}= 0.00236
G_{Corr}= -0.017503

I	1.71526300	-4.53439900	1.23403600

I^-_{an}

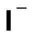

E (ωB97xD) = -297.9697315
G_{Corr}= -0.016848

I	1.71526300	-4.53439900	1.23403600

CH_4

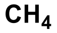

E (ωB97xD) = -40.52009414
E (B3LYP-D3(BJ)) = -40.53837596
E (PBE0-D3(BJ)) = -40.4755937
E (M062X) = -40.50095338
H_{Corr}= 0.04834
H (CBS-4M) = -40.425069

C	0.10905500	2.02492600	0.00000000
H	0.47544400	0.98848700	0.00000100
H	0.47546200	2.54313000	0.89759000
H	0.47546100	2.54313100	-0.89759100
H	-0.99025100	2.02493600	0.00000000

·CH_3

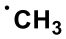

E (ωB97xD) = -39.83790429
E (B3LYP-D3(BJ)) = -39.85797395
E (PBE0-D3(BJ)) = -39.7987687
E (M062X) = -39.82303739
H_{Corr}= 0.033466
H (CBS-4M) = -39.754999

C	0.01998200	2.27674700	0.00000000
H	0.53456400	2.45967400	0.94582700
H	0.53456400	2.45967400	-0.94582700
H	-1.00962700	1.91240200	0.00000000

Lut

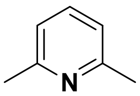

E (ωB97xD) = -326.9426466
E (B3LYP-D3(BJ)) = -327.0713696
E (PBE0-D3(BJ)) = -326.672975
E (M062X) = -326.9062814
G_{Corr}= 0.110866

C	1.33105000	2.10241500	0.00033700
C	2.73390600	2.10474600	-0.00025100
C	3.40090500	3.32281500	-0.00108500
C	2.65716000	4.50248600	-0.00126500
C	1.26274300	4.41785600	-0.00055100
N	0.62656600	3.23666900	0.00016600
H	4.49322100	3.35765600	-0.00160900
H	3.28274100	1.16035600	-0.00008500
H	3.14925100	5.47717900	-0.00194600
C	0.56771800	0.80615700	0.00095400
H	0.81651300	0.20301200	-0.88618800
H	0.81995800	0.20167600	0.88618800
H	-0.51230000	1.00327300	0.00310600
C	0.39565400	5.64592900	-0.00036200
H	-0.26094500	5.64937200	-0.88439700
H	-0.25633900	5.65221500	0.88710500
H	0.99317900	6.56741700	-0.00322400

Lut_I_rad

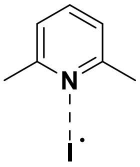

E (ωB97xD) = -624.7213148
E (B3LYP-D3(BJ)) = -624.8725053
E (PBE0-D3(BJ)) = -624.3893292
E (M062X) = -624.5325941
G_{Corr}= 0.107452

C	1.36639300	2.08143300	0.02463000
C	2.76289200	2.10572600	0.09331400
C	3.42209300	3.32965200	0.10178100
C	2.67807200	4.50247300	0.04201900
C	1.28350000	4.42488000	-0.02580800
N	0.67728100	3.22957400	-0.03231500
H	4.51237100	3.36943100	0.15483800
H	3.31601200	1.16618100	0.13884700
H	3.16326900	5.47983600	0.04711000
C	0.61526100	0.78327700	0.01346400
H	0.01227400	0.69277700	-0.90272300
H	1.30859500	-0.06594400	0.06390600
H	-0.07391900	0.72760200	0.86973300

C	0.44232300	5.66472700	-0.09265200
H	-0.16257900	5.67280700	-1.01204100
H	-0.25216900	5.70759600	0.76004600
H	1.07354100	6.56243600	-0.07843000
I	-2.07655200	3.12924000	-0.16903800

1a

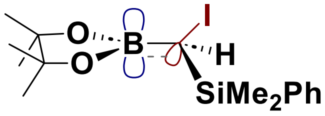

E (ωB97xD) = -1348.883703

E (B3LYP-D3(BJ)) = -1349.207822

E (PBE0-D3(BJ)) = -1348.067013

E (M062X) = -1348.61495

H_{Corr}= 0.390508

G_{Corr}= 0.311157

C	1.25921800	-4.35289900	1.24730600
C	1.25344300	-2.85301400	1.72375100
B	2.44017100	-3.06075100	-0.19557100
O	1.73477200	-2.16192000	0.55056600
O	2.29743200	-4.34680100	0.24074700
C	-0.03628800	-4.76319000	0.55139100
H	0.11396500	-5.73556900	0.05967700
H	-0.86135600	-4.86493200	1.27066000
H	-0.33144600	-4.03289400	-0.21669100
C	-0.11606900	-2.30946300	2.09383800
H	-0.54624100	-2.88855500	2.92455100
H	-0.02448600	-1.26260800	2.41921400
H	-0.81114100	-2.34326300	1.24472400
C	2.25333100	-2.57538600	2.84387600
H	2.34038100	-1.48772400	2.98259000
H	1.92442200	-3.01838600	3.79464000
H	3.25197700	-2.96843100	2.60073600
C	1.61974000	-5.36060900	2.32529700
H	0.89679400	-5.31018100	3.15315300
H	1.58990500	-6.37815300	1.90818300
H	2.62651900	-5.18516600	2.72645000
C	3.26688000	-2.69631500	-1.47785900
H	3.96464500	-3.51357600	-1.71477700
I	4.52970800	-0.97287600	-1.12453900
Si	2.13347300	-2.44619200	-2.98854800
C	0.91789500	-1.05876600	-2.67121500
C	1.23535500	-4.07512000	-3.23986700
C	3.21272900	-2.07058100	-4.48983700
H	0.23682900	-0.93103700	-3.52816200
H	0.31009700	-1.29266900	-1.78278200
H	1.42448700	-0.09802400	-2.48836000
H	0.60705600	-4.03397700	-4.14464100
H	1.94512100	-4.91000000	-3.35480700
H	0.58279500	-4.30231400	-2.38200000
C	4.12635500	-3.03131000	-4.95744600
C	3.15254400	-0.83944400	-5.16171400
C	4.94961300	-2.77312200	-6.05318700
H	4.20289200	-4.00280900	-4.45815800
C	3.97332600	-0.57489300	-6.26023600
H	2.45530500	-0.06624200	-4.82595300
C	4.87369300	-1.54174800	-6.70736600
H	5.65326100	-3.53437600	-6.39943200
H	3.90982900	0.39077900	-6.76812500
H	5.51760100	-1.33643600	-7.56624300

1a_T

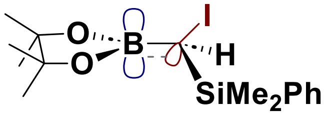

E (ωB97xD) = -1348.796275

E (B3LYP-D3(BJ)) = -1349.125707

E (PBE0-D3(BJ)) = -1347.975887

E (M062X) = -1348.535623

G_{Corr}= 0.305184

C	1.41903200	-4.74989600	1.40676200
C	0.89114300	-3.26628400	1.41168500
B	2.00959500	-3.70184300	-0.52358500
O	1.05797200	-2.88124900	0.03374800
O	2.34567200	-4.74336000	0.30201900
C	0.33260800	-5.76982500	1.07051500
H	0.80448000	-6.74653400	0.88758800
H	-0.38321300	-5.88315000	1.89721000
H	-0.22175600	-5.48415000	0.16399200
C	-0.57081900	-3.11448900	1.79871700
H	-0.74041900	-3.49523100	2.81702600
H	-0.85070500	-2.05059800	1.78148300
H	-1.23272700	-3.65235400	1.10738400
C	1.75599800	-2.32479200	2.24760900
H	1.45125500	-1.28685700	2.04726900
H	1.63541300	-2.51659800	3.32341300
H	2.82156900	-2.42165600	1.99132700
C	2.15367500	-5.16471900	2.67099200
H	1.49030700	-5.08420400	3.54515800
H	2.47870500	-6.21233400	2.58531200
H	3.04322900	-4.54551400	2.84631900
C	2.60589300	-3.45312800	-1.92618800
H	3.32590300	-4.18681100	-2.31317700
I	5.48527300	-1.82306300	-0.09883900
Si	2.11218500	-1.99121300	-2.97582500
C	2.42892300	-0.39858300	-2.02146500
C	0.28287700	-2.10929500	-3.39381300
C	3.14846400	-1.97388800	-4.55917400
H	2.03282200	0.47005800	-2.57354800
H	1.93682600	-0.44150900	-1.03690400
H	3.50811500	-0.22982100	-1.86459000
H	-0.05644100	-1.22891900	-3.96392200
H	0.05509400	-3.01233300	-3.98259000
H	-0.29989100	-2.15577700	-2.45990900
C	4.54839900	-2.08283000	-4.48112100
C	2.57442800	-1.83273200	-5.83271300
C	5.34381600	-2.05207400	-5.62659400
H	5.03295900	-2.19702500	-3.50516400
C	3.36445300	-1.80105000	-6.98448700
H	1.48858800	-1.74678100	-5.93686100
C	4.75127700	-1.91054200	-6.88315400
H	6.42988900	-2.13968000	-5.54004400
H	2.89430800	-1.69123000	-7.96515200
H	5.37135900	-1.88708200	-7.78287700

1a-H

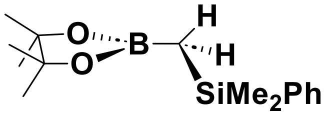

E (ωB97xD) = -1051.69775
E (B3LYP-D3(BJ)) = -1052.004614
E (PBE0-D3(BJ)) = -1050.938742
E (M062X) = -1051.57988
H_{Corr}= 0.3984
H (CBS-4M) = -1049.929179

C	1.99656300	-4.42451000	1.69661200
C	0.91360500	-3.54614000	0.96724800
B	2.99535400	-3.02257900	0.21172300
O	1.66704100	-2.97982500	-0.12313700
O	3.22010700	-3.74148000	1.35768000
C	2.10120300	-5.83777600	1.12567100
H	2.99649700	-6.32523000	1.53909800
H	1.22536400	-6.44642400	1.39237700
H	2.19559400	-5.82304400	0.02932200
C	-0.26370000	-4.32715500	0.40634100
H	-0.79659900	-4.85212900	1.21332000
H	-0.97155500	-3.63711000	-0.07666900
H	0.05635700	-5.06482300	-0.34125700
C	0.41349300	-2.38184000	1.82015400
H	-0.17477000	-1.70208700	1.18606600
H	-0.23080200	-2.73044600	2.63997700
H	1.24862200	-1.80910700	2.25059400
C	1.85572400	-4.48043000	3.20870500
H	0.88297500	-4.91212000	3.48832100
H	2.64586000	-5.11815400	3.63239000
H	1.94153800	-3.48443000	3.66258100
C	4.12722500	-2.39876300	-0.65926300
H	4.98129200	-2.09508600	-0.03099400
H	3.75832400	-1.51533500	-1.20611800
Si	4.74295500	-3.66452100	-1.91352500
C	3.30866100	-4.27322000	-2.96539200
C	5.52244500	-5.12253700	-1.01944700
C	6.04135500	-2.87054300	-3.04139900
H	3.65356800	-4.97361500	-3.74340800
H	2.56510600	-4.79724800	-2.34308500
H	2.79892000	-3.43308600	-3.46476800
H	5.91793200	-5.86229000	-1.73451600
H	6.35418800	-4.79304900	-0.37541500
H	4.77980100	-5.62779800	-0.38142300
C	6.48019100	-1.54788200	-2.87103300
C	6.60672900	-3.61574300	-4.09186700
C	7.44401700	-0.98852200	-3.71356300
H	6.06506200	-0.93437900	-2.06592900
C	7.56951100	-3.06430700	-4.93737100
H	6.29223800	-4.65134100	-4.25958000
C	7.99061400	-1.74629600	-4.74900600
H	7.76873700	0.04389900	-3.56030500
H	7.99364100	-3.66398500	-5.74686700
H	8.74447100	-1.31109000	-5.40996700

1a-rad

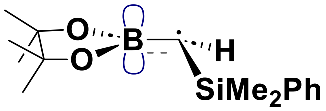

E (ωB97xD) = -1051.02764
E (B3LYP-D3(BJ)) = -1051.337841
E (PBE0-D3(BJ)) = -1050.273948
E (M062X) = -1050.914472
H_{Corr}= 0.385833
H (CBS-4M) = -1049.268443
G_{Corr}= 0.309934

C	1.48320900	-4.73056800	1.14466800
C	0.84003500	-3.29476500	1.16715900
B	2.53419600	-3.37058300	-0.34645000
O	1.38134300	-2.69935800	-0.03035500
O	2.70488100	-4.50136100	0.41578400
C	0.66573400	-5.73980400	0.34030100
H	1.26534000	-6.65066600	0.19547900
H	-0.25954100	-6.01738200	0.86511200
H	0.40061100	-5.34472200	-0.65183500
C	-0.67763700	-3.27815300	1.09159000
H	-1.11045100	-3.81634300	1.94818100
H	-1.04023700	-2.23988600	1.12304400
H	-1.04417100	-3.73923600	0.16505800
C	1.31895600	-2.44522900	2.34305100
H	0.99859200	-1.40474600	2.18564400
H	0.89208900	-2.79654900	3.29331400
H	2.41609600	-2.45532500	2.42715600
C	1.81634100	-5.29887300	2.51415400
H	0.90552700	-5.38660100	3.12525400
H	2.25087500	-6.30360600	2.40387400
H	2.54031800	-4.67130600	3.05025200
C	3.54471600	-2.93343700	-1.42942500
H	3.31037800	-2.03156300	-2.01130600
Si	5.10546900	-3.89212200	-1.78810200
C	4.66451400	-5.59417500	-2.45430200
C	6.08756400	-4.07772500	-0.19319400
C	6.14879300	-2.94482500	-3.05104900
H	5.56584200	-6.21065300	-2.60532100
H	4.01611500	-6.11137800	-1.72904000
H	4.12323500	-5.53137200	-3.41190700
H	7.00809800	-4.65903300	-0.36713500
H	6.37557500	-3.09522800	0.21446600
H	5.48120300	-4.59953900	0.56409400
C	6.39964700	-1.57309400	-2.86970500
C	6.71806900	-3.57017200	-4.17200700
C	7.18700900	-0.85397500	-3.76914000
H	5.97012700	-1.04930300	-2.00885900
C	7.50771200	-2.85687500	-5.07702800
H	6.54476200	-4.63567700	-4.35059900
C	7.74365800	-1.49679400	-4.87675900
H	7.36702700	0.21189900	-3.60707000
H	7.93930700	-3.36541600	-5.94297900
H	8.36030600	-0.93629000	-5.58397300

1a_lut

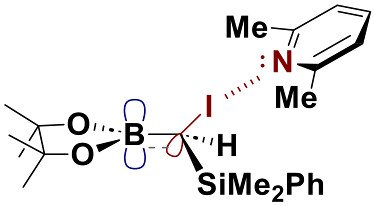

E (ωB97xD) = -1675.833345
E (B3LYP-D3(BJ)) = -1676.286625
E (PBE0-D3(BJ)) = -1674.747936
E (M062X) = -1675.527088
G_{Corr}= 0.44152

C	1.11133600	-4.87533500	1.15807700
C	1.28888500	-3.48814000	1.87831500
B	2.28409900	-3.44747100	-0.15864300
O	1.73061500	-2.64196300	0.79610100
O	2.06164100	-4.77637800	0.07421300
C	-0.26826500	-5.04729900	0.52626500
H	-0.25306000	-5.92915400	-0.13107000
H	-1.04345700	-5.20092200	1.29042400
H	-0.54523900	-4.17279100	-0.08142800
C	0.01371600	-2.91339700	2.47147700
H	-0.39630800	-3.59489500	3.23179200
H	0.23079800	-1.95072300	2.95765900
H	-0.75164800	-2.74276800	1.70302500
C	2.40343900	-3.49278800	2.92235300
H	2.60793400	-2.45656700	3.22951200
H	2.11617900	-4.06651500	3.81501300
H	3.33441700	-3.91739700	2.51739700
C	1.45343300	-6.08333200	2.01329400
H	0.80477700	-6.11901100	2.90133700
H	1.29247400	-7.00608500	1.43623700
H	2.50032600	-6.06505000	2.34338400
C	3.02573200	-2.92747500	-1.43521800
H	3.59316800	-3.75207900	-1.89325500
I	4.50263800	-1.41520300	-0.95663700
Si	1.80256400	-2.27843700	-2.73974100
C	0.71655800	-0.93604300	-2.01428900
C	0.76404500	-3.74615700	-3.28087800
C	2.79941700	-1.60585800	-4.19737700
H	-0.01015900	-0.57165900	-2.75840700
H	0.15489700	-1.33346500	-1.15431100
H	1.30760800	-0.07637000	-1.66079200
H	0.04609400	-3.44953900	-4.06294000
H	1.39547700	-4.55359600	-3.68540900
H	0.19351900	-4.15582500	-2.43155600
C	3.73355500	-2.42671000	-4.85335500
C	2.67220800	-0.27836100	-4.63538500
C	4.51310900	-1.94062400	-5.90266500
H	3.86634500	-3.46670300	-4.53697600
C	3.44794000	0.21456500	-5.68735000
H	1.96080300	0.39375100	-4.14671100
C	4.37178900	-0.61567000	-6.32135700
H	5.23584400	-2.59605200	-6.39523100
H	3.33395100	1.25304800	-6.00840300
H	4.98353100	-0.23139100	-7.14142200
C	7.41460100	1.12857000	0.16314500
C	8.46390600	2.05426400	0.14279000
C	8.72449100	2.74682800	-1.03560800
C	7.93469900	2.49995200	-2.15421900
C	6.90084900	1.56122400	-2.05899000
N	6.66117100	0.89956200	-0.91931900
H	9.53768100	3.47537100	-1.08170700
H	9.06202700	2.22437300	1.04016500
H	8.11036600	3.02662600	-3.09432000
C	7.08564300	0.34376500	1.40203800
H	7.71809000	0.64156600	2.24910700
H	6.03048900	0.49004900	1.68024600
H	7.22906000	-0.73347400	1.22186800
C	6.01299400	1.25797400	-3.23443100
H	6.09918200	0.19847600	-3.52284800
H	4.95800600	1.43706500	-2.97595800
H	6.27135800	1.87780200	-4.10356200

1a_lut_T

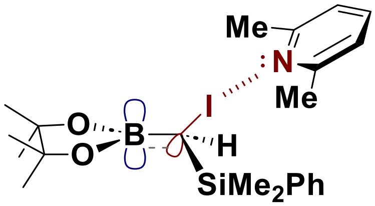

E (ωB97xD) = -1675.702257
E (B3LYP-D3(BJ)) = -1676.161821
E (PBE0-D3(BJ)) = -1674.623754
E (M062X) = -1675.392412
G_{Corr}= 0.444075

C	1.04065300	-4.36157700	1.37747000
C	1.07251400	-2.84037800	1.78075900
B	2.36032200	-3.19125400	-0.04776700
O	1.64706000	-2.22770700	0.60358300
O	2.13052400	-4.44700400	0.42786900
C	-0.23332700	-4.75677100	0.63607400
H	-0.09430100	-5.75246000	0.18993200
H	-1.09312000	-4.80176200	1.31948500
H	-0.46822000	-4.04839300	-0.17173900
C	-0.29019100	-2.22536600	2.04802000
H	-0.78690300	-2.74538500	2.88060500
H	-0.17188000	-1.16816500	2.32796300
H	-0.94039700	-2.27435200	1.16496500
C	2.02333500	-2.54595500	2.93847100
H	2.14796800	-1.45691000	3.02921100
H	1.62664600	-2.92796500	3.88973900
H	3.01620100	-2.98960500	2.76969100
C	1.30593500	-5.32615100	2.52050600
H	0.54542500	-5.20500600	3.30623900
H	1.25430700	-6.36161000	2.15262800
H	2.29792000	-5.16979600	2.96403000
C	3.32083000	-2.90285300	-1.25943300
H	3.95032700	-3.78563200	-1.44732600
I	4.70698600	-1.35727200	-0.65621700
Si	2.34328200	-2.49146100	-2.84526000
C	1.43227900	-0.87020100	-2.64896600
C	1.14408400	-3.91983600	-3.06320800
C	3.52046800	-2.44031300	-4.31481700
H	0.75783700	-0.69583000	-3.50284700
H	0.82411000	-0.89518400	-1.73075100
H	2.12253300	-0.01481800	-2.57695700
H	0.61340800	-3.82609000	-4.02481300
H	1.67228100	-4.88687300	-3.05871400
H	0.39018900	-3.94079700	-2.26045600
C	4.34437300	-3.54364400	-4.59823200
C	3.59108300	-1.33030500	-5.17113000
C	5.22099300	-3.53098900	-5.68223700

H	4.31291500	-4.43060500	-3.95680600
C	4.46311500	-1.31388700	-6.26143800
H	2.96191400	-0.45494400	-4.98557100
C	5.28411200	-2.41205900	-6.51579700
H	5.86071800	-4.39541700	-5.87692000
H	4.50687700	-0.43548900	-6.91019700
H	5.97449700	-2.39710600	-7.36270000
C	7.25290500	-2.13697500	-2.98332800
C	8.13728700	-1.99013100	-4.02749300
C	8.17292400	-0.80282100	-4.80845300
C	7.25220000	0.22620300	-4.47043500
C	6.36433900	0.08172900	-3.42886900
N	6.39721700	-1.08915800	-2.69090100
H	8.87323100	-0.68874900	-5.63697800
H	8.80761200	-2.82508400	-4.25113000
H	7.22012500	1.15683300	-5.04431800
C	7.16095800	-3.38224600	-2.15743000
H	7.87232300	-4.13573600	-2.52215400
H	7.38085100	-3.18338800	-1.09484900
H	6.14712600	-3.81490100	-2.20186800
C	5.35793400	1.12547400	-3.05812600
H	4.33002400	0.73019900	-3.12503800
H	5.49901800	1.47439000	-2.02027300
H	5.44041300	1.99182700	-3.72859000

TS1

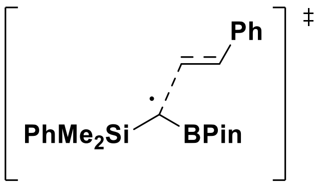

E (ωB97xD) = -1360.689025

G_{Corr}= 0.436086

C	2.14603700	-4.99142300	0.40844800
C	1.19195100	-3.76004300	0.61714700
B	2.93639900	-3.20096800	-0.73723800
O	1.64970200	-2.85221900	-0.40087700
O	3.33065100	-4.36859800	-0.12861400
C	1.63462800	-5.96050700	-0.65768100
H	2.43730900	-6.66881500	-0.91095000
H	0.76789100	-6.53483900	-0.30032700
H	1.34743100	-5.42887600	-1.57807700
C	-0.28206600	-4.05566700	0.39194700
H	-0.63142300	-4.83159400	1.08965300
H	-0.87517700	-3.14595200	0.56904300
H	-0.47454100	-4.39263800	-0.63533800
C	1.39447400	-3.06796100	1.96346300
H	0.85428900	-2.10959300	1.95705100
H	1.00521900	-3.67611300	2.79266900
H	2.45821600	-2.85835000	2.15206700
C	2.50428800	-5.74283700	1.67914100
H	1.59724300	-6.14186600	2.15762800
H	3.16295000	-6.59051200	1.43783800
H	3.02684400	-5.09897000	2.39890700
C	3.77149500	-2.40656100	-1.75993300
H	4.82201900	-2.69737300	-1.88956000
C	4.28295400	-0.48828000	-0.46841300
H	4.82180200	-1.16748300	0.19589200
H	3.23635500	-0.29993800	-0.22033600
C	4.93634100	0.34406300	-1.32633300
H	4.35332200	1.10480600	-1.85638300
C	6.34896300	0.26417100	-1.69929600
C	7.21717100	-0.72237500	-1.19177000
C	6.86829800	1.18952300	-2.62430000
C	8.54954300	-0.77312500	-1.59151100
H	6.84618300	-1.46170900	-0.47823000
C	8.20197300	1.13779600	-3.02299400
H	6.20742000	1.95851900	-3.03409900
C	9.04980000	0.15566000	-2.50793800
H	9.20549500	-1.54749100	-1.18609200
H	8.58211300	1.86758300	-3.74206000
H	10.09572100	0.11142500	-2.82054100
Si	2.86642900	-1.79867100	-3.27518400
C	4.07784300	-1.22952000	-4.59309300
C	1.63618200	-0.44017500	-2.86911700
C	1.91617100	-3.32876000	-3.88224900
H	3.55269800	-0.92846600	-5.51443200
H	4.66066900	-0.36520100	-4.23420900
H	4.79059700	-2.02932100	-4.85215200
H	1.00109600	-0.20637900	-3.73945900
H	0.98684100	-0.74148200	-2.03281400
H	2.16203900	0.48329600	-2.57956000
C	2.62127000	-4.46118300	-4.32683200
C	0.51866800	-3.42689300	-3.79014800
C	1.96069500	-5.64191900	-4.66775700
H	3.71353200	-4.42915200	-4.40023600
C	-0.15021300	-4.60607900	-4.12672000
H	-0.06683900	-2.57229400	-3.43912900
C	0.57026400	-5.71725900	-4.56576800
H	2.53228700	-6.50905200	-5.00865900
H	-1.23889300	-4.65816000	-4.04278100
H	0.04942700	-6.64211600	-4.82631700

2_rad

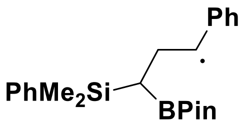

E (ωB97xD) = -1360.739184

G_{Corr}= 0.442363

C	2.07962500	-4.90316900	0.27226500
C	1.20125200	-3.67445100	0.72208200
B	2.84966100	-2.98827400	-0.69151800
O	1.61857700	-2.64519900	-0.19576500
O	3.21543700	-4.26030600	-0.34268600
C	1.41169600	-5.75685900	-0.80245500
H	2.14945900	-6.46675000	-1.20436600
H	0.56880800	-6.33154600	-0.39253900
H	1.04478300	-5.13945200	-1.63432500
C	-0.29782200	-3.88394700	0.58265300
H	-0.62825000	-4.73074400	1.20260200
H	-0.83191100	-2.98387700	0.92196700
H	-0.58462000	-4.07776000	-0.45938500
C	1.53594800	-3.17733900	2.12685400
H	1.02960600	-2.21519700	2.29447500
H	1.19553700	-3.88393500	2.89724500
H	2.61767000	-3.01787600	2.25068500
C	2.56565100	-5.78481700	1.41123300
H	1.71145800	-6.21363800	1.95638700
H	3.16348600	-6.61558800	1.00760700
H	3.19161600	-5.22812200	2.12089000
C	3.72007600	-2.01340800	-1.55440100

H	4.66084100	-2.53123000	-1.81081800
C	4.05074000	-0.70957200	-0.76787800
H	4.57937000	-0.98439300	0.16276300
H	3.10915100	-0.23306700	-0.45455400
C	4.86341100	0.28234900	-1.53685600
H	4.34554900	1.13324700	-1.99167400
C	6.25208100	0.15877000	-1.80401100
C	7.04464100	-0.90300400	-1.28249900
C	6.90690200	1.11409000	-2.63292800
C	8.39879100	-0.99517000	-1.57717300
H	6.58431500	-1.65677300	-0.63976700
C	8.25959700	1.01131500	-2.92107600
H	6.32097500	1.93926100	-3.04757600
C	9.01821300	-0.04409200	-2.39690900
H	8.98461600	-1.82029600	-1.16393600
H	8.73518300	1.75824800	-3.56187000
H	10.08345700	-0.12391200	-2.62555000
Si	2.81836700	-1.70216900	-3.19880500
C	4.01433700	-1.15767500	-4.54557000
C	1.47011800	-0.41860000	-2.96070500
C	2.04257900	-3.34354600	-3.74297700
H	3.50023900	-1.11550200	-5.52030900
H	4.43021300	-0.15949400	-4.33659600
H	4.85734700	-1.86197200	-4.63720500
H	0.88241200	-0.27728800	-3.88238900
H	0.78375700	-0.71312400	-2.15126100
H	1.91074400	0.55590300	-2.69477400
C	2.86009000	-4.45817800	-3.99967400
C	0.65549100	-3.50445300	-3.89049700
C	2.31728500	-5.68199100	-4.39131200
H	3.94632600	-4.37639100	-3.88889400
C	0.10425400	-4.72685900	-4.28165200
H	-0.01620600	-2.66371000	-3.69389600
C	0.93524900	-5.81844900	-4.53392800
H	2.97400000	-6.53427600	-4.58365900
H	-0.97888900	-4.82726600	-4.38855400
H	0.50676300	-6.77639400	-4.83914500

2_cat

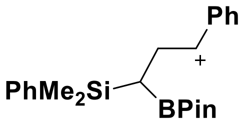

E (ωB97xD) = -1360.572527

G_{Corr}= 0.445783

C	2.12524600	-4.90819700	0.24236900
C	1.28657800	-3.66527400	0.73106000
B	2.87343000	-3.00949600	-0.75569800
O	1.67843800	-2.64009500	-0.20793500
O	3.24090100	-4.28161300	-0.43205800
C	1.39526200	-5.75562300	-0.79499700
H	2.10219100	-6.47845800	-1.22777700
H	0.56530300	-6.31546900	-0.34098500
H	0.99717000	-5.13661100	-1.61100500
C	-0.21971600	-3.85061800	0.65769500
H	-0.53441700	-4.69258200	1.29198800
H	-0.72364700	-2.94261700	1.02079800
H	-0.55530500	-4.03971200	-0.37047400
C	1.69294100	-3.17055300	2.11682000
H	1.20738900	-2.20182100	2.30611500
H	1.37836100	-3.87208600	2.90243800
H	2.78134600	-3.02625200	2.19111100
C	2.65775400	-5.79045000	1.35916000
H	1.82624400	-6.20574000	1.94784500
H	3.22389300	-6.63022400	0.92966400
H	3.32469100	-5.23821100	2.03405700
C	3.72846700	-2.04177500	-1.65486300
H	4.65781400	-2.56159100	-1.94150500
C	4.11046700	-0.78892600	-0.78815200
H	4.66544500	-1.11369800	0.10284300
H	3.19170700	-0.27388600	-0.47317900
C	4.90537700	0.11498200	-1.62244500
H	4.35312400	0.85025500	-2.22131300
C	6.28913500	0.09822400	-1.80025600
C	7.13601100	-0.81654500	-1.10625300
C	6.85886800	1.01093800	-2.73663700
C	8.49291700	-0.81193000	-1.35132900
H	6.71104400	-1.51874400	-0.38759100
C	8.22043500	1.00809300	-2.96754200
H	6.20132400	1.70486100	-3.26545900
C	9.02960000	0.09795600	-2.27625200
H	9.15041100	-1.50883600	-0.82985700
H	8.66376200	1.70340000	-3.68147900
H	10.10651800	0.09486500	-2.46111000
Si	2.73758500	-1.70687600	-3.25676700
C	3.87966400	-1.14577000	-4.63938900
C	1.41854700	-0.42282600	-2.91300900
C	1.95002000	-3.34950200	-3.75552000
H	3.32084500	-1.09229800	-5.58842000
H	4.30749000	-0.14866600	-4.44803100
H	4.71374900	-1.85279700	-4.77742500
H	0.78462000	-0.25672400	-3.79898400
H	0.77492800	-0.73357200	-2.07541000
H	1.87866700	0.54346700	-2.64923200
C	2.75735000	-4.46458900	-4.04003300
C	0.55799300	-3.50472700	-3.85308600
C	2.19751400	-5.68680200	-4.41124700
H	3.84688200	-4.38481600	-3.96879900
C	-0.00879600	-4.72574500	-4.22455500
H	-0.10351100	-2.66188200	-3.63307600
C	0.81096100	-5.81892200	-4.50508100
H	2.84444800	-6.54096300	-4.62634700
H	-1.09505500	-4.82399100	-4.29341000
H	0.36967700	-6.77585600	-4.79459600

1b

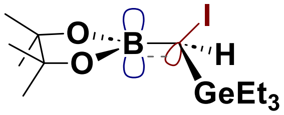

E (ωB97xD) = -3062.641614

E (B3LYP-D3(BJ)) = -3062.88728

E (PBE0-D3(BJ)) = -3061.654553

E (M062X) = -3062.304673

H_{Corr}= 0.422607

G_{Corr}= 0.341837

C	1.50285800	-4.60090100	1.02030600
C	1.04758800	-3.23138900	1.65225400
B	2.55027700	-2.86435400	-0.00912800
O	1.53514700	-2.27460200	0.68937800
O	2.64701700	-4.20651000	0.23047400
C	0.47280300	-5.19108100	0.06083100

H	0.92890600	-6.03035300	-0.48434600
H	-0.40457300	-5.57067900	0.60355200
H	0.13362900	-4.44874900	-0.67591600
C	-0.45602700	-3.06729300	1.79518000
H	-0.86747700	-3.85050300	2.44919100
H	-0.68130800	-2.09080900	2.24909400
H	-0.96431200	-3.11704600	0.82327700
C	1.74246400	-2.91932300	2.97570800
H	1.52998900	-1.87603300	3.25194300
H	1.38019500	-3.57042700	3.78392900
H	2.83401000	-3.03367200	2.89560900
C	1.93781300	-5.65049900	2.02954500
H	1.10739900	-5.89645700	2.70810100
H	2.23242400	-6.57077300	1.50364600
H	2.79278400	-5.31092300	2.62863000
C	3.48471500	-2.10824400	-1.01450100
H	4.28404000	-2.77981200	-1.35852300
I	4.53413400	-0.54492700	0.06616400
Ge	2.52232400	-1.45426400	-2.63331800
C	1.13036900	-2.78495600	-3.02032900
C	1.70938000	0.30424700	-2.34201800
C	3.79326200	-1.38746000	-4.12621600
H	0.42592300	-2.79183600	-2.17131600
H	0.55818200	-2.41583700	-3.88873800
C	1.66191000	-4.19333800	-3.28614000
H	2.50732900	1.00816600	-2.05298000
H	1.34642000	0.64342000	-3.32801700
C	0.57626900	0.32961100	-1.31712300
H	4.19509100	-2.40508900	-4.26777000
H	3.20319500	-1.16243000	-5.03185000
C	4.93557700	-0.38386600	-3.97375900
H	0.84419100	-4.90861300	-3.47349200
H	2.32455000	-4.21648000	-4.16609500
H	2.23859100	-4.57568300	-2.42917200
H	-0.25982000	-0.31977900	-1.62358600
H	0.91236000	-0.02580000	-0.33107100
H	0.17029800	1.34660200	-1.18957200
H	5.58603500	-0.37297300	-4.86391000
H	4.56127400	0.64262600	-3.83005500
H	5.57208200	-0.62296500	-3.10702200

1b-H

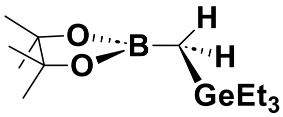

E (ωB97xD) = -2765.456424
E (B3LYP-D3(BJ)) = -2765.684567
E (PBE0-D3(BJ)) = -2764.52724
E (M062X) = -2765.269852
H_{Corr}= 0.430444
H (CBS-4M) = -2762.672206

C	1.91205200	-4.33520500	1.72932100
C	0.84993500	-3.48725300	0.93738800
B	2.95754700	-2.97481200	0.23380000
O	1.63666800	-2.94856000	-0.14256500
O	3.13961100	-3.65021700	1.41598300
C	2.04756700	-5.76212800	1.20032400
H	2.94103500	-6.22520300	1.64483800

H	1.17407900	-6.37498500	1.46525900
H	2.16438100	-5.77464000	0.10644300
C	-0.30256400	-4.29384700	0.36104700
H	-0.86464600	-4.79011600	1.16646800
H	-0.99307700	-3.62527700	-0.17447200
H	0.04690700	-5.05882200	-0.34474900
C	0.31336000	-2.30178100	1.73728700
H	-0.25743900	-1.64568800	1.06351900
H	-0.35594000	-2.63026700	2.54532300
H	1.12987500	-1.71028600	2.17795100
C	1.71789700	-4.35150400	3.23641000
H	0.73821900	-4.78143500	3.49363300
H	2.49592300	-4.97287900	3.70464200
H	1.78299000	-3.34318500	3.66594700
C	4.11785800	-2.37173400	-0.60528300
H	4.93640300	-2.02893700	0.04881000
H	3.77902600	-1.52026300	-1.21653300
Ge	4.90892300	-3.70279300	-1.83320400
C	5.36042600	-5.33805300	-0.84274400
C	3.64889000	-4.12069400	-3.28404500
C	6.54262900	-2.92316900	-2.59901700
H	4.42161400	-5.86201000	-0.60017400
H	5.91154600	-5.99936400	-1.53404900
C	6.16779700	-5.09775100	0.43242500
H	3.29605100	-3.16381800	-3.70551800
H	4.22299400	-4.61475200	-4.08733300
C	2.45780800	-4.98662300	-2.87136000
H	7.26124500	-2.76405300	-1.77677800
H	6.99489000	-3.68182600	-3.26134100
C	6.31359200	-1.61869600	-3.36155600
H	6.43748300	-6.04478100	0.92909800
H	7.10773700	-4.55874200	0.22902900
H	5.58936700	-4.50154100	1.15499100
H	2.78293800	-5.97026200	-2.49539800
H	1.87152000	-4.50572400	-2.07321400
H	1.77591800	-5.17233700	-3.71787300
H	7.25260700	-1.21342100	-3.77361900
H	5.62060100	-1.75811400	-4.20733100
H	5.88134500	-0.84053900	-2.71147700

1b-rad

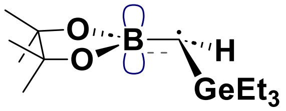

E (ωB97xD) = -2764.785816
E (B3LYP-D3(BJ)) = -2765.017058
E (PBE0-D3(BJ)) = -2763.862471
E (M062X) = -2764.604615
H_{Corr}= 0.417629
H (CBS-4M) = -2762.009413
G_{Corr}= 0.339114

C	1.84799900	-4.65503800	1.50706200
C	0.65481200	-3.71091200	1.10716700
B	2.42147700	-3.37852700	-0.28796100
O	1.05568800	-3.25414300	-0.19663200
O	2.94665000	-4.08802700	0.76494600
C	1.66618100	-6.08882300	1.01160000
H	2.61172800	-6.63513400	1.14432500
H	0.88330800	-6.61418900	1.57714500

H	1.40408200	-6.11598800	-0.05680600
C	-0.69443900	-4.40241600	1.00085400
H	-0.97563000	-4.84962800	1.96610400
H	-1.46731100	-3.66879700	0.72685600
H	-0.68690700	-5.19064400	0.23654100
C	0.54471700	-2.47560700	1.99978000
H	-0.16404700	-1.76827300	1.54443000
H	0.17600900	-2.73475200	3.00266700
H	1.51478000	-1.96682300	2.10467000
C	2.19031500	-4.65263400	2.98784800
H	1.32704000	-4.98821300	3.58197000
H	3.02460000	-5.34413400	3.17847300
H	2.48877000	-3.65468300	3.33492900
C	3.24481600	-2.77849100	-1.44351200
H	4.33303800	-2.92946000	-1.43296000
Ge	2.46999000	-1.72944100	-2.88470500
C	0.75098900	-2.52083700	-3.41027700
C	2.18570900	0.10939900	-2.23691500
C	3.70200200	-1.70272700	-4.41565300
H	0.05184500	-2.37689000	-2.57142500
H	0.35769800	-1.93657900	-4.26011800
C	0.83791400	-4.00342000	-3.76958500
H	3.17469200	0.54752600	-2.02118800
H	1.76787100	0.69001700	-3.07788400
C	1.28157600	0.21466500	-1.00973900
H	3.84640600	-2.74322400	-4.75316200
H	3.19266200	-1.17951200	-5.24340700
C	5.05180200	-1.04820700	-4.12330500
H	-0.14396600	-4.41362500	-4.05864800
H	1.52621200	-4.18281700	-4.61167500
H	1.20206400	-4.59810200	-2.91608400
H	0.27653200	-0.19120400	-1.20705200
H	1.69271300	-0.34687000	-0.15614900
H	1.15447400	1.26136900	-0.68720400
H	5.71996600	-1.07883800	-4.99979500
H	4.93701600	0.01016300	-3.83871000
H	5.57815800	-1.55227800	-3.29619200

1b_lut

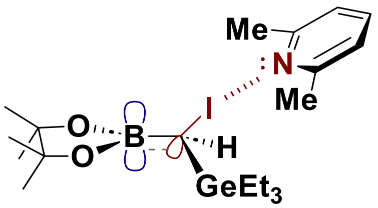

E (ωB97xD) = -3389.59053
E (B3LYP-D3(BJ)) = -3389.965228
E (PBE0-D3(BJ)) = -3388.334761
E (M062X) = -3389.216444
G_{Corr}= 0.47041

C	1.26735900	-4.74541900	0.93028600
C	0.89107900	-3.34816800	1.55069600
B	2.40799400	-3.07921700	-0.11723700
O	1.42116300	-2.43108600	0.57370100
O	2.43340800	-4.42281900	0.14133800
C	0.20694400	-5.27948200	-0.02949300
H	0.61453800	-6.14754400	-0.56814500
H	-0.69389200	-5.60325800	0.51102800
H	-0.08343800	-4.52222100	-0.77231000
C	-0.60027800	-3.10246500	1.70490400
H	-1.04753500	-3.85686900	2.36927400
H	-0.76919100	-2.11141100	2.15177100
H	-1.11897000	-3.13308200	0.73769300
C	1.61397400	-3.06061900	2.86514500
H	1.46443200	-2.00333000	3.12954400
H	1.22236600	-3.67971600	3.68482700
H	2.69653900	-3.23835100	2.77753100
C	1.63746000	-5.81145500	1.94798600
H	0.79266800	-6.00390500	2.62616300
H	1.87964100	-6.75128200	1.42988200
H	2.50926200	-5.51751900	2.54703500
C	3.38357800	-2.37612300	-1.11690500
H	4.13014300	-3.09729500	-1.47985700
I	4.53115600	-0.88954700	-0.02479200
Ge	2.45799000	-1.63461400	-2.71395300
C	0.98170100	-2.86348900	-3.12918000
C	1.76052400	0.17004500	-2.39621800
C	3.72603200	-1.60625900	-4.21239000
H	0.27321100	-2.83502800	-2.28370900
H	0.43971100	-2.44999100	-3.99695700
C	1.42343400	-4.30058700	-3.40590000
H	2.60601700	0.81894900	-2.11305900
H	1.40785700	0.54014500	-3.37493400
C	0.64498100	0.26322500	-1.35629700
H	4.06819500	-2.64125400	-4.38244800
H	3.14882700	-1.32326300	-5.11007800
C	4.92577500	-0.67508300	-4.03825000
H	0.56365100	-4.96038100	-3.60837600
H	2.09201500	-4.35772600	-4.27988600
H	1.96640100	-4.72830500	-2.54816300
H	-0.23496600	-0.33007900	-1.65376900
H	0.97059300	-0.11836600	-0.37662000
H	0.30644800	1.30348900	-1.21910200
H	5.57225000	-0.67568600	-4.93144400
H	4.61298600	0.36704100	-3.86238100
H	5.55108500	-0.97508400	-3.18234800
C	6.53646900	2.46101700	0.73897000
C	7.14674000	3.55521000	1.36273500
C	7.34509700	3.51957500	2.73935600
C	6.93164600	2.39923400	3.45333800
C	6.32818700	1.34246300	2.76224600
N	6.14330200	1.38771200	1.43678000
H	7.81972300	4.35948100	3.25248400
H	7.45895800	4.41803100	0.77110700
H	7.07241400	2.33714900	4.53419600
C	6.29218900	2.44603600	-0.74426300
H	6.69282800	3.34785200	-1.22628100
H	6.76108000	1.56238000	-1.20401300
H	5.21270500	2.38751700	-0.95549300
C	5.85709000	0.10818000	3.47907000
H	4.76783800	-0.00983000	3.36536500
H	6.32813000	-0.78947500	3.04961600
H	6.09253300	0.15019600	4.55097300

1c

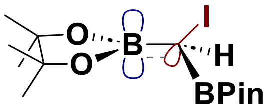

E (ωB97xD) = -1159.174678
E (B3LYP-D3(BJ)) = -1159.501632
E (PBE0-D3(BJ)) = -1158.391478

E (M062X) = -1158.913981
H_{Corr}= 0.405737
G_{Corr}= 0.329341

C	1.81565400	-4.70431600	0.86635900
C	0.95964700	-3.38733100	0.96975800
B	2.93763100	-2.95741100	-0.04404000
O	1.66095800	-2.49687100	0.07035300
O	3.11979000	-4.18922800	0.51652700
C	1.37741700	-5.61548800	-0.27751600
H	2.12199300	-6.41447400	-0.40564400
H	0.40579300	-6.08378800	-0.06537600
H	1.30115800	-5.06376200	-1.22653800
C	-0.47981800	-3.52811400	0.50635600
H	-1.00544000	-4.27896400	1.11498800
H	-1.00337500	-2.56757900	0.62220200
H	-0.54020200	-3.82539700	-0.54875200
C	1.01271600	-2.74666900	2.35443200
H	0.56787800	-1.74217800	2.30051200
H	0.44638300	-3.33509000	3.09019300
H	2.04859500	-2.64218200	2.71104400
C	1.92246700	-5.49480800	2.15904400
H	0.92474600	-5.81278200	2.49665800
H	2.53007600	-6.39669600	1.99299600
H	2.39412300	-4.90771400	2.95777800
C	4.09520600	-2.18728900	-0.78607800
I	5.24985100	-1.14481200	0.71699700
H	3.66852200	-1.40162900	-1.42513100
B	4.93253700	-3.20953400	-1.63545500
O	4.40621300	-3.74344700	-2.77818800
O	6.15308600	-3.71834100	-1.31574400
C	5.21766100	-4.88888200	-3.12464300
C	6.58191800	-4.54897000	-2.41884600
C	5.29664900	-5.00681900	-4.63656700
C	4.52775600	-6.11431900	-2.52933100
C	7.50867000	-3.69781700	-3.28308100
C	7.32917600	-5.75258100	-1.87032400
H	5.98669600	-5.81515200	-4.92131200
H	4.30316400	-5.24743500	-5.04315900
H	5.63900200	-4.07251500	-5.10046800
H	3.50407800	-6.17570900	-2.92694000
H	5.05594200	-7.04120100	-2.79434400
H	4.46389600	-6.04446400	-1.43343200
H	8.33613200	-3.32657100	-2.66072500
H	7.93557200	-4.28374400	-4.10946100
H	6.98210200	-2.82881500	-3.70510800
H	7.57451500	-6.45322500	-2.68239500
H	8.27107200	-5.42551400	-1.40551200
H	6.74058600	-6.28474200	-1.11170400

1c-H

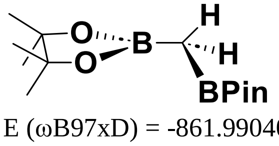

E (ωB97xD) = -861.9904009
E (B3LYP-D3(BJ)) = -862.3006946
E (PBE0-D3(BJ)) = -861.2655743
E (M062X) = -861.8802697
H_{Corr}= 0.413951
H (CBS-4M) = -860.542268

C	1.83443600	-4.62426200	1.26534600
C	1.07532700	-3.35720300	0.72393600
B	3.32310500	-3.01976500	0.65486500
O	2.14030500	-2.60960100	0.10167100
O	3.16954900	-4.11563500	1.46320000
C	1.93380600	-5.74442700	0.23163700
H	2.64852700	-6.49955600	0.59102100
H	0.96307900	-6.23618800	0.07514500
H	2.29470600	-5.36639200	-0.73619600
C	0.00281300	-3.65428900	-0.31127500
H	-0.76954100	-4.31272200	0.11380800
H	-0.48229800	-2.71760500	-0.62402500
H	0.42189100	-4.13534200	-1.20477000
C	0.50394900	-2.48143600	1.83724600
H	0.17522200	-1.52432900	1.40598200
H	-0.36242800	-2.95724300	2.31864600
H	1.25858600	-2.26681800	2.60872200
C	1.30477200	-5.16905000	2.58117500
H	0.25166600	-5.47014000	2.47593900
H	1.88474900	-6.05604900	2.87686700
H	1.37967200	-4.42831900	3.38810300
C	4.71176000	-2.36223200	0.35149800
H	5.29991500	-2.27588500	1.27834700
H	4.56278500	-1.36088300	-0.08192600
B	5.46403100	-3.27974500	-0.67100900
O	5.13305100	-3.35501700	-1.99857600
O	6.47847700	-4.13555900	-0.33672400
C	5.80845300	-4.50087300	-2.55604700
C	7.00683500	-4.69719400	-1.55582400
C	6.21752300	-4.19416200	-3.98690600
C	4.81466900	-5.66054200	-2.52790500
C	8.23941200	-3.87660700	-1.93034900
C	7.39727800	-6.14535700	-1.30946000
H	6.82042000	-5.01721300	-4.39902400
H	5.32048800	-4.08181300	-4.61398500
H	6.79865500	-3.26505300	-4.05375600
H	3.90902400	-5.36833100	-3.07983200
H	5.23151700	-6.56053200	-3.00210300
H	4.52043900	-5.91181200	-1.49825200
H	8.95184400	-3.89932500	-1.09245600
H	8.74046100	-4.28652200	-2.81896200
H	7.97985000	-2.82587500	-2.12859300
H	7.70660900	-6.62538200	-2.25002100
H	8.24555000	-6.18808400	-0.61009000
H	6.56945300	-6.72180100	-0.87618100

1c-rad

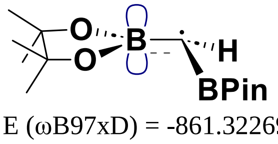

E (ωB97xD) = -861.3226954
E (B3LYP-D3(BJ)) = -861.6362777
E (PBE0-D3(BJ)) = -860.6032277
E (M062X) = -861.2180692
H_{Corr}= 0.401506
H (CBS-4M) = -859.882747
G_{Corr}= 0.330106

C	1.50198100	-4.72168300	1.40301300
C	1.15437500	-3.18763100	1.38833700

B	2.01952800	-3.81402500	-0.61605100
O	1.22704400	-2.87244200	-0.01614000
O	2.31912100	-4.85776500	0.22292700
C	0.27806200	-5.61406200	1.20375800
H	0.61377700	-6.64558700	1.02109400
H	-0.36784400	-5.61505400	2.09341900
H	-0.31923400	-5.29151300	0.33779400
C	-0.23321200	-2.84587900	1.90585200
H	-0.34376700	-3.17024000	2.95146900
H	-0.38625700	-1.75698500	1.86862800
H	-1.01984700	-3.32099700	1.30515300
C	2.20432900	-2.32971100	2.09254600
H	2.00912800	-1.27082200	1.86739300
H	2.16686100	-2.46241700	3.18326500
H	3.22027600	-2.57060600	1.74533200
C	2.29350500	-5.18189200	2.61612700
H	1.72530500	-4.99305000	3.53931400
H	2.48372300	-6.26344300	2.54828800
H	3.26184700	-4.66960600	2.68870500
C	2.54095300	-3.73943800	-2.07142400
H	3.25258000	-4.52133000	-2.36977600
B	2.18948700	-2.69153100	-3.15492400
O	1.21886800	-1.73356100	-3.03677300
O	2.84036100	-2.63154900	-4.36165700
C	1.05528000	-1.10973400	-4.32577800
C	2.43349700	-1.41494400	-5.01976200
C	0.75601600	0.36748900	-4.13020800
C	-0.12421400	-1.79981200	-5.00889300
C	3.50111300	-0.36517600	-4.71597300
C	2.35015900	-1.65879700	-6.51759900
H	0.72408200	0.88507200	-5.10069800
H	-0.22534600	0.48714000	-3.64742900
H	1.50899700	0.85424200	-3.49657000
H	-1.01002200	-1.71564100	-4.36212500
H	-0.35862200	-1.33274600	-5.97616600
H	0.07602900	-2.86885500	-5.17554300
H	4.47810500	-0.74156500	-5.05348900
H	3.29702000	0.57960300	-5.23980200
H	3.56889100	-0.15980200	-3.63710400
H	1.94508800	-0.77235000	-7.02841300
H	3.35553100	-1.85390400	-6.91971000
H	1.71459200	-2.52202800	-6.75492500

1c_lut

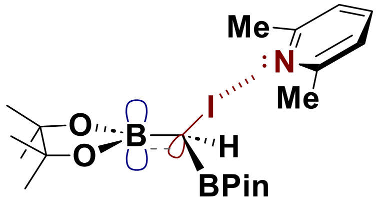

E (ωB97xD) = -1486.123366
E (B3LYP-D3(BJ)) = -1486.579562
E (PBE0-D3(BJ)) = -1485.071617
E (M062X) = -1485.825682
G_{Corr}= 0.459076

C	1.62639000	-4.72830200	0.67272100
C	0.80460900	-3.38775100	0.72723200
B	2.77591000	-3.05125300	-0.33364600
O	1.50652000	-2.56279600	-0.23047600
O	2.93586400	-4.26114700	0.28307300
C	1.14966700	-5.67944000	-0.42244400
H	1.87544200	-6.49908200	-0.52700700
H	0.17217300	-6.11726400	-0.17478700
H	1.06902800	-5.16844100	-1.39370000
C	-0.64842700	-3.51584500	0.30438800
H	-1.17700000	-4.22277300	0.96123600
H	-1.14682100	-2.53833600	0.38403200
H	-0.74089600	-3.86301400	-0.73306800
C	0.90640100	-2.67988600	2.07664800
H	0.48704700	-1.66748800	1.98111100
H	0.34275900	-3.21423100	2.85456800
H	1.95301000	-2.58496800	2.40411900
C	1.73270000	-5.46172600	1.99880900
H	0.73264000	-5.73845200	2.36473600
H	2.31518400	-6.38553100	1.86629900
H	2.23088400	-4.85091700	2.76297000
C	3.95006800	-2.32644500	-1.08793600
I	5.02468200	-1.19284900	0.41594500
H	3.53969800	-1.58703900	-1.79025200
B	4.82038000	-3.39382300	-1.83771600
O	4.36590200	-3.97539900	-2.98984100
O	6.00935300	-3.90619200	-1.41420200
C	5.17258400	-5.15135300	-3.21977300
C	6.49342500	-4.79901800	-2.44179800
C	5.35040600	-5.35386400	-4.71434000
C	4.41714800	-6.32952000	-2.60763000
C	7.49297800	-4.00980700	-3.28377400
C	7.17816200	-5.98561100	-1.78480600
H	6.04173800	-6.18776100	-4.90780400
H	4.38189900	-5.59988300	-5.17455400
H	5.74205500	-4.45217400	-5.20323700
H	3.41960300	-6.39069300	-3.06718200
H	4.93838100	-7.28037400	-2.78824600
H	4.28467700	-6.19847900	-1.52344200
H	8.28410200	-3.62015100	-2.62631300
H	7.96254200	-4.64464100	-4.04870700
H	7.01329900	-3.15521500	-3.78396600
H	7.46257800	-6.73135700	-2.54217600
H	8.09351400	-5.65127400	-1.27428800
H	6.52993900	-6.46797700	-1.04161300
C	7.65546600	0.96680800	2.49293900
C	8.33676800	1.65208400	3.50522800
C	7.73709200	1.77480300	4.75457900
C	6.48040100	1.21393400	4.95893400
C	5.85862000	0.54287000	3.89979200
N	6.44595600	0.43082700	2.70125700
H	8.24564400	2.30446000	5.56379200
H	9.32165300	2.08030500	3.30890400
H	5.97988400	1.29162600	5.92606300
C	8.25432000	0.80035900	1.12461600
H	9.23094700	1.29701900	1.04953700
H	8.38633700	-0.26764500	0.89043800
H	7.58562900	1.22185000	0.35816700
C	4.50171100	-0.08270600	4.06449100
H	3.79114700	0.33987700	3.33726800
H	4.55187800	-1.16682200	3.87751000
H	4.10553600	0.07909800	5.07590100

1d

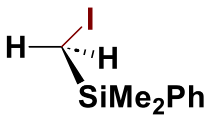

E (ωB97xD) = -938.1447087
E (B3LYP-D3(BJ)) = -938.3227602
E (PBE0-D3(BJ)) = -937.6677613
E (M062X) = -937.9218882
H_{Corr}= 0.207653
G_{Corr}= 0.150384

C	3.27073600	-2.71343600	-1.50297500
H	3.95645200	-3.55888600	-1.65908800
I	4.53351300	-1.01869700	-1.06520200
Si	2.13231400	-2.45370500	-2.99734500
C	0.93769300	-1.05101400	-2.66409900
C	1.21496900	-4.07089100	-3.25426500
C	3.21240200	-2.07445300	-4.49866300
H	0.24644000	-0.91491400	-3.51172300
H	0.33356200	-1.27694000	-1.77007700
H	1.45745000	-0.09578600	-2.48922700
H	0.53004000	-3.99574400	-4.11481200
H	1.91310800	-4.90147100	-3.44634900
H	0.61727400	-4.32730400	-2.36419200
C	4.15268100	-3.01926400	-4.94613200
C	3.12770300	-0.85511600	-5.18931500
C	4.97737500	-2.75714200	-6.03987900
H	4.24966200	-3.98148500	-4.43259100
C	3.94955400	-0.58682200	-6.28620000
H	2.40978000	-0.09397400	-4.86993600
C	4.87639200	-1.53773800	-6.71278700
H	5.70193200	-3.50593400	-6.36979200
H	3.86627100	0.36951300	-6.80870100
H	5.52139700	-1.32937800	-7.57010600
H	2.69574300	-2.90537500	-0.58496200

1d-H

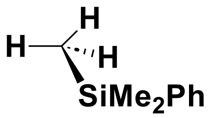

E (ωB97xD) = -640.9581548
E (B3LYP-D3(BJ)) = -641.1192635
E (PBE0-D3(BJ)) = -640.5393724
E (M062X) = -640.8859495
H_{Corr}= 0.215395
H (CBS-4M) = -639.86651

C	4.14908400	-2.40444000	-0.65368100
H	4.97349600	-2.04814500	-0.01464600
H	3.68835400	-1.52885300	-1.13951900
Si	4.74420100	-3.66339500	-1.91405400
C	3.30420500	-4.25075700	-2.97201600
C	5.51961500	-5.13675900	-1.03868900
C	6.04616500	-2.87338300	-3.04278200
H	3.63682600	-4.98337100	-3.72561000
H	2.53360200	-4.73482300	-2.34902500
H	2.83204100	-3.40821400	-3.50357300
H	5.88848600	-5.88142300	-1.76303300
H	6.37073800	-4.82330300	-0.41203600
H	4.78335300	-5.63651200	-0.38719600
C	6.46459200	-1.54154100	-2.89073900
C	6.63146300	-3.62662100	-4.07652800
C	7.42761100	-0.98185100	-3.73410200
H	6.03443300	-0.92016200	-2.09956400
C	7.59359800	-3.07506400	-4.92274000
H	6.33347200	-4.66930500	-4.23007500
C	7.99428600	-1.74827600	-4.75224500
H	7.73618600	0.05750100	-3.59486100
H	8.03356800	-3.68157200	-5.71860500
H	8.74787200	-1.31302400	-5.41349800
H	3.38986300	-2.86049500	0.00330000

1d-rad

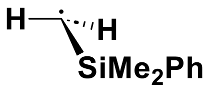

E (ωB97xD) = -640.2830084
E (B3LYP-D3(BJ)) = -640.4464401
E (PBE0-D3(BJ)) = -639.8694955
E (M062X) = -640.2157665
H_{Corr}= 0.201931
H (CBS-4M) = -639.201398
G_{Corr}= 0.149831

C	3.58916200	-2.88441800	-1.40706100
H	3.01884700	-2.36026700	-2.18526100
Si	5.10262000	-3.89591600	-1.78157000
C	4.63336900	-5.57146400	-2.49822300
C	6.09210700	-4.13649700	-0.20142100
C	6.14830900	-2.94583500	-3.04431100
H	5.53034800	-6.18392700	-2.68833700
H	3.99421300	-6.12398300	-1.79034900
H	4.08057700	-5.46655300	-3.44569600
H	6.99546900	-4.73795100	-0.39491600
H	6.41100100	-3.16887100	0.21889100
H	5.49191600	-4.65891700	0.56206200
C	6.34862500	-1.56226200	-2.89336300
C	6.76942600	-3.58125600	-4.13185000
C	7.13943400	-0.84141900	-3.78859500
H	5.87414800	-1.03195500	-2.06051300
C	7.56203900	-2.86612700	-5.03257100
H	6.63523300	-4.65595000	-4.28744600
C	7.74888400	-1.49426400	-4.86183300
H	7.27985700	0.23376500	-3.65062600
H	8.03455500	-3.38238900	-5.87218000
H	8.36782600	-0.93250100	-5.56604800
H	3.16574900	-2.79410000	-0.39822100

1d_lut

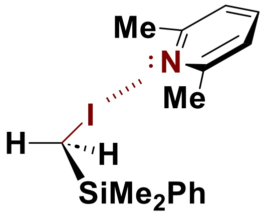

E (ωB97xD) = -1265.093634
E (B3LYP-D3(BJ)) = -1265.400828
E (PBE0-D3(BJ)) = -1264.347909
E (M062X) = -1264.833546
G_{Corr}= 0.281084

C	2.79221800	-3.15832900	-1.60340400
H	3.20611400	-4.17132000	-1.71591600
I	4.41291500	-1.92998400	-0.87559400
Si	2.07594300	-2.51257500	-3.23530200
C	1.17432500	-0.89723600	-2.93770400
C	0.90309700	-3.82152500	-3.89503100
C	3.51255500	-2.25522000	-4.43698000
H	0.74068200	-0.50630800	-3.87253400
H	0.34891700	-1.05418400	-2.22395800
H	1.84049400	-0.12567400	-2.51996600
H	0.44617100	-3.49309300	-4.84302600
H	1.42878100	-4.77187000	-4.08161100
H	0.09119000	-4.01750100	-3.17539000
C	4.33791100	-3.33632800	-4.79344400
C	3.82610700	-0.99141800	-4.96182900
C	5.43520200	-3.16241700	-5.63679500
H	4.12950300	-4.33750800	-4.40180300
C	4.92208700	-0.81028900	-5.80867500
H	3.21168400	-0.12373700	-4.70431400
C	5.73006200	-1.89587300	-6.14594100
H	6.06455200	-4.01717500	-5.89732800
H	5.14838000	0.18400500	-6.20201700
H	6.59044500	-1.75644400	-6.80525700
H	2.02895800	-3.18978100	-0.81125300
C	7.53988900	0.48179500	-1.52018900
C	8.71755900	1.23117300	-1.41582800
C	9.27390800	1.43810100	-0.15735200
C	8.64397600	0.89521100	0.95829600
C	7.46905900	0.15690600	0.77676700
N	6.94277400	-0.03578000	-0.43882400
H	10.19257800	2.01937800	-0.04670700
H	9.18497200	1.64363900	-2.31216200
H	9.05195700	1.03820000	1.96083400
C	6.89454500	0.22483200	-2.85417000
H	7.44110100	0.72007800	-3.66804500
H	6.85893200	-0.85574200	-3.06447700
H	5.85540100	0.58830500	-2.85586900
C	6.74224300	-0.45813000	1.93976400
H	5.70005200	-0.10436600	1.97123200
H	6.71172500	-1.55422200	1.83549300
H	7.22578600	-0.21273700	2.89484800

1e

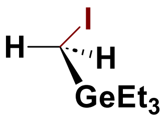

E (ωB97xD) = -2651.901251
E (B3LYP-D3(BJ)) = -2652.001105
E (PBE0-D3(BJ)) = -2651.255019
E (M062X) = -2651.610752
H_{Corr}= 0.238568
G_{Corr}= 0.180391

C	3.36400100	-2.11114100	-0.98576800
H	4.02981500	-2.96430200	-1.17528600
I	4.57622800	-0.66567500	0.06626800
Ge	2.52859100	-1.47174900	-2.66076200
C	1.13952100	-2.79356600	-3.08645700
C	1.72380900	0.29118500	-2.36955600
C	3.85373100	-1.42281600	-4.10706500
H	0.40471200	-2.79037700	-2.26371900
H	0.60387400	-2.42931900	-3.98020000
C	1.66912300	-4.20832800	-3.32056100
H	2.53470400	1.01357800	-2.17756000
H	1.25782800	0.59868500	-3.32164100
C	0.70025700	0.32666000	-1.23500900
H	4.36667300	-2.39941300	-4.12407500
H	3.28371200	-1.36212500	-5.05065100
C	4.87476800	-0.28743500	-4.03539700
H	0.85570500	-4.91380100	-3.55714400
H	2.38359800	-4.24187900	-4.15883400
H	2.19043600	-4.59853400	-2.43133500
H	-0.13781200	-0.36445800	-1.42001400
H	1.15366700	0.04039400	-0.27174300
H	0.27067700	1.33356400	-1.10591800
H	5.55764100	-0.30367400	-4.90081600
H	4.38553400	0.69981800	-4.02444500
H	5.49503300	-0.35354000	-3.12768700
H	2.59852800	-2.42595800	-0.26305800

1e-H

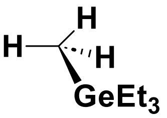

E (ωB97xD) = -2354.71482
E (B3LYP-D3(BJ)) = -2354.797374
E (PBE0-D3(BJ)) = -2354.127061
E (M062X) = -2354.574984
H_{Corr}= 0.247147
H (CBS-4M) = -2352.606499

C	4.03025700	-2.48172400	-0.65674300
H	4.74350500	-2.03744100	0.05558700
H	3.56710700	-1.66335000	-1.23056700
Ge	4.93193200	-3.75027700	-1.85089700
C	5.41939200	-5.36270400	-0.83714100
C	3.71791900	-4.23452700	-3.31960800
C	6.55350800	-2.91373800	-2.58304000
H	4.48621200	-5.87096900	-0.53916500

H	5.93710600	-6.05216700	-1.52651000
C	6.28526200	-5.08916000	0.39269500
H	3.55698800	-3.33425300	-3.93730700
H	4.24168400	-4.96011400	-3.96597700
C	2.37568700	-4.80316000	-2.85902300
H	7.26310300	-2.76344600	-1.75129700
H	7.02982600	-3.63947600	-3.26497100
C	6.30164500	-1.59003500	-3.30518200
H	6.53796600	-6.01723500	0.93184700
H	7.23609200	-4.60177400	0.12192400
H	5.77292200	-4.42645900	1.10922000
H	2.50492600	-5.71842900	-2.25857600
H	1.82238300	-4.08127300	-2.23627500
H	1.72556700	-5.06274600	-3.71097900
H	7.23369200	-1.15547000	-3.70306800
H	5.61210400	-1.71426900	-4.15609300
H	5.85513700	-0.84018500	-2.63180800
H	3.23808100	-2.98266700	-0.07797600

1e-rad

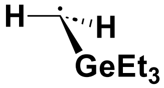

E (ωB97xD) = -2354.038574
E (B3LYP-D3(BJ)) = -2354.123492
E (PBE0-D3(BJ)) = -2353.456278
E (M062X) = -2353.903725
H_{Corr}= 0.232667
H (CBS-4M) = -2351.940488
G_{Corr}= 0.177364

C	3.26957400	-2.77340600	-1.40686300
H	4.34333500	-2.98850200	-1.33638700
Ge	2.48754400	-1.73782200	-2.84419700
C	0.75515200	-2.53753700	-3.31477100
C	2.20493300	0.11605500	-2.24192300
C	3.70100200	-1.75505700	-4.38849300
H	0.11957300	-2.51628500	-2.41307300
H	0.26712200	-1.87651000	-4.05174800
C	0.85741600	-3.96280900	-3.85707700
H	3.18937800	0.54714000	-1.99385900
H	1.82418400	0.68872200	-3.10554700
C	1.25473200	0.24330000	-1.05205600
H	3.86758300	-2.80634600	-4.67827100
H	3.17147800	-1.28257300	-5.23385000
C	5.03723200	-1.05531200	-4.14017800
H	-0.13445300	-4.38842200	-4.08215100
H	1.44754100	-4.00229400	-4.78699900
H	1.34613900	-4.63579700	-3.13306700
H	0.25028000	-0.14263300	-1.28991500
H	1.62263500	-0.32131900	-0.17951900
H	1.13376900	1.29244900	-0.73543000
H	5.69654200	-1.10925900	-5.02217800
H	4.89876700	0.01163000	-3.90210200
H	5.58427100	-1.50873500	-3.29735700
H	2.66940200	-3.17331600	-0.57923700

1e_lut

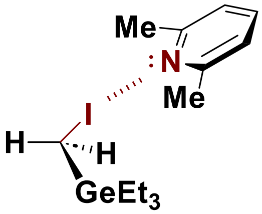

E (ωB97xD) = -2978.849517
E (B3LYP-D3(BJ)) = -2979.078267
E (PBE0-D3(BJ)) = -2977.934308
E (M062X) = -2978.522168
G_{Corr}= 0.308487

C	2.86096200	-2.43128000	-0.99180400
H	3.38381500	-3.38750900	-1.13441800
I	4.15930100	-1.22031700	0.23867100
Ge	2.40408800	-1.60869800	-2.72642400
C	1.05018900	-2.77410100	-3.54417100
C	1.66878400	0.18279800	-2.41652900
C	3.99366000	-1.53624000	-3.88174700
H	0.16583200	-2.78927100	-2.88491500
H	0.72730700	-2.29217700	-4.48334800
C	1.53621500	-4.19799800	-3.81498800
H	2.48490300	0.83176000	-2.05675200
H	1.36250700	0.58896300	-3.39604000
C	0.49871500	0.20943500	-1.43370900
H	4.58044200	-2.45296900	-3.70100900
H	3.63308900	-1.60697500	-4.92265400
C	4.87630100	-0.29799400	-3.71939900
H	0.75373000	-4.81363600	-4.28839600
H	2.40987300	-4.20893400	-4.48686100
H	1.83402300	-4.70863800	-2.88484100
H	-0.33795800	-0.42069300	-1.77644200
H	0.79936100	-0.15860300	-0.43898800
H	0.10415400	1.22967200	-1.29710600
H	5.73808300	-0.32412600	-4.40663000
H	4.31841000	0.62809200	-3.93184100
H	5.27536700	-0.21004500	-2.69669800
H	1.95822200	-2.62032900	-0.39359300
C	6.14465800	0.75115100	3.16016400
C	6.81896200	1.73595100	3.89104000
C	7.42734000	2.78247700	3.20501800
C	7.34830200	2.81678100	1.81630400
C	6.65747700	1.79613700	1.15299500
N	6.07475300	0.79424300	1.82384600
H	7.96080800	3.56578000	3.74898900
H	6.86246800	1.67592000	4.98028600
H	7.81569600	3.62178600	1.24579900
C	5.46251200	-0.40019000	3.84425100
H	5.63396800	-0.38409300	4.92892700
H	4.37661000	-0.36452700	3.66269300
H	5.82851600	-1.35756500	3.44257900
C	6.53867600	1.78136500	-0.34572800
H	6.92749200	0.83520300	-0.75253300
H	5.48253800	1.85763100	-0.64895500
H	7.09212700	2.61354100	-0.80108500

References

(1) Zhang, L.; Oestreich, M. Diastereotopic Group-Selective Intramolecular Aldol Reactions Initiated by Enantioselective Conjugate Silylation: Diastereodivergence Controlled by the Silicon Nucleophile. *ACS Catalysis* **2021**, *11*, 3516-3522.
(2) Kurandina, D.; Parasram, M.; Gevorgyan, V. Visible Light-Induced Room-Temperature Heck Reaction of Functionalized Alkyl Halides with Vinyl Arenes/Heteroarenes. *Angewandte Chemie International Edition* **2017**, *56*, 14212-14216.
(3) Selmani, A.; Schoetz, M. D.; Queen, A. E.; Schoenebeck, F. Modularity in the Csp3 Space─Alkyl Germanes as Orthogonal Molecular Handles for Chemoselective Diversification. *ACS Catalysis* **2022**, *12*, 4833-4839.
(4) Zhang, Z.-Q.; Yang, C.-T.; Liang, L.-J.; Xiao, B.; Lu, X.; Liu, J.-H.; Sun, Y.-Y.; Marder, T. B.; Fu, Y. Copper-Catalyzed/Promoted Cross-coupling of gem-Diborylalkanes with Nonactivated Primary Alkyl Halides: An Alternative Route to Alkylboronic Esters. *Organic Letters* **2014**, *16*, 6342-6345.
(5) Hu, J.; Tang, M.; Wang, J.; Wu, Z.; Friedrich, A.; Marder, T. B. Photocatalyzed Borylcyclopropanation of Alkenes with a (Diborylmethyl)iodide Reagent. *Angewandte Chemie International Edition* **2023**, *62*, e202305175.
(6) Mercadante, M. A.; Kelly, C. B.; Hamlin, T. A.; Delle Chiaie, K. R.; Drago, M. D.; Duffy, K. K.; Dumas, M. T.; Fager, D. C.; Glod, B. L. C.; Hansen, K. E.; et al. 1,3-γ-Silyl-elimination in electron-deficient cationic systems. *Chem Sci* **2014**, *5*, 3983-3994.
(7) Molloy, J. J.; Seath, C. P.; West, M. J.; McLaughlin, C.; Fazakerley, N. J.; Kennedy, A. R.; Nelson, D. J.; Watson, A. J. B. Interrogating Pd(II) Anion Metathesis Using a Bifunctional Chemical Probe: A Transmetalation Switch. *Journal of the American Chemical Society* **2018**, *140*, 126-130.
(8) Schnell, S. D.; González, J. A.; Sklyaruk, J.; Linden, A.; Gademann, K. Boron Trifluoride-Mediated Cycloaddition of 3-Bromotetrazine and Silyl Enol Ethers: Synthesis of 3-Bromo-pyridazines. *The Journal of Organic Chemistry* **2021**, *86*, 12008-12023.
(9) Körte, L. A.; Warner, R.; Vishnevskiy, Y. V.; Neumann, B.; Stammler, H.-G.; Mitzel, N. W. Intramolecular pyridine-based frustrated Lewis-pairs. *Dalton Transactions* **2015**, *44*, 9992-10002.
(10) Zhou, Y.; Bandar, J. S.; Buchwald, S. L. Enantioselective CuH-Catalyzed Hydroacylation Employing Unsaturated Carboxylic Acids as Aldehyde Surrogates. *Journal of the American Chemical Society* **2017**, *139*, 8126-8129.
(11) Lawer, A.; Rossi-Ashton, J. A.; Stephens, T. C.; Challis, B. J.; Epton, R. G.; Lynam, J. M.; Unsworth, W. P. Internal Nucleophilic Catalyst Mediated Cyclisation/Ring Expansion Cascades for the Synthesis of Medium-Sized Lactones and Lactams. *Angewandte Chemie International Edition* **2019**, *58*, 13942-13947.
(12) Su, N.; Theorell, J. A.; Wink, D. J.; Driver, T. G. Copper-Catalyzed Formation of α-Alkoxycycloalkenones from N-Tosylhydrazones. *Angewandte Chemie International Edition* **2015**, *54*, 12942-12946.
(13) Lee, Y. H.; Morandi, B. Palladium-Catalyzed Intermolecular Aryliodination of Internal Alkynes. *Angewandte Chemie International Edition* **2019**, *58*, 6444-6448.
(14) Ratushnyy, M.; Kamenova, M.; Gevorgyan, V. A mild light-induced cleavage of the S–O bond of aryl sulfonate esters enables efficient sulfonylation of vinylarenes. *Chem Sci* **2018**, *9*, 7193-7197.
(15) Spinnato, D.; Schweitzer-Chaput, B.; Goti, G.; Ošeka, M.; Melchiorre, P. A Photochemical Organocatalytic Strategy for the α-Alkylation of Ketones by using Radicals. *Angewandte Chemie International Edition* **2020**, *59*, 9485-9490.
(16) Bae, H. Y.; Höfler, D.; Kaib, P. S. J.; Kasaplar, P.; De, C. K.; Döhring, A.; Lee, S.; Kaupmees, K.; Leito, I.; List, B. Approaching sub-ppm-level asymmetric organocatalysis of a highly challenging and scalable carbon–carbon bond forming reaction. *Nature Chemistry* **2018**, *10*, 888-894.
(17) Khan, I.; Reed-Berendt, B. G.; Melen, R. L.; Morrill, L. C. FLP-Catalyzed Transfer Hydrogenation of Silyl Enol Ethers. *Angewandte Chemie International Edition* **2018**, *57*, 12356-12359.

(18) Pieck, J. C.; Kuch, D.; Grolle, F.; Linne, U.; Haas, C.; Carell, T. PNA-Based Reagents for the Direct and Site-Specific Synthesis of Thymine Dimer Lesions in Genomic DNA. *Journal of the American Chemical Society* **2006**, *128*, 1404-1405.
(19) Cotman, A. E.; Durcik, M.; Benedetto Tiz, D.; Fulgheri, F.; Secci, D.; Sterle, M.; Možina, Š.; Skok, Ž.; Zidar, N.; Zega, A.; et al. Discovery and Hit-to-Lead Optimization of Benzothiazole Scaffold-Based DNA Gyrase Inhibitors with Potent Activity against Acinetobacter baumannii and Pseudomonas aeruginosa. *Journal of Medicinal Chemistry* **2023**, *66*, 1380-1425.
(20) Dokai, Y.; Fujioka, A.; Saito, K.; Yamada, T. Rhenium-Catalyzed Decarboxylative Coupling of Cyclic Enol Carbonates with Silyl Enol Ethers and Ketene Silyl Acetals. *Organic Letters* **2023**, *25*, 2275-2279.
(21) Liu, Q.; Zhu, F.-P.; Jin, X.-L.; Wang, X.-J.; Chen, H.; Wu, L.-Z. Visible-Light-Driven Intermolecular [2+2] Cycloadditions between Coumarin-3-Carboxylates and Acrylamide Analogs. *Chemistry – A European Journal* **2015**, *21*, 10326-10329.
(22) *Kessil Photoscience products overview*. https://kessil.com/products/science_main.php (accessed March 2023).
(23) Shin, M.; Kim, M.; Hwang, C.; Lee, H.; Kwon, H.; Park, J.; Lee, E.; Cho, S. H. Facile Synthesis of α-Boryl-Substituted Allylboronate Esters Using Stable Bis[(pinacolato)boryl]methylzinc Reagents. *Organic Letters* **2020**, *22*, 2476-2480.
(24) Nagaraju, A.; Saiaede, T.; Eghbarieh, N.; Masarwa, A. Photoredox-Mediated Deoxygenative Radical Additions of Aromatic Acids to Vinyl Boronic Esters and gem-Diborylalkenes**. *Chemistry – A European Journal* **2023**, *29*, e202202646.
(25) Roth, H. G.; Romero, N. A.; Nicewicz, D. A. Experimental and Calculated Electrochemical Potentials of Common Organic Molecules for Applications to Single-Electron Redox Chemistry. *Synlett* **2016**, *27*, 714-723.
(26) Cismesia, M. A.; Yoon, T. P. Characterizing chain processes in visible light photoredox catalysis. *Chem Sci* **2015**, *6*, 5426-5434.
(27) (a) Teegardin, K.; Day, J. I.; Chan, J.; Weaver, J. Advances in Photocatalysis: A Microreview of Visible Light Mediated Ruthenium and Iridium Catalyzed Organic Transformations. *Organic Process Research & Development* **2016**, *20*, 1156-1163. (b) Romero, N. A.; Nicewicz, D. A. Organic Photoredox Catalysis. *Chem Rev* **2016**, *116*, 10075-10166. (c) Nevesely, T.; Wienhold, M.; Molloy, J. J.; Gilmour, R. Advances in the E -> Z Isomerization of Alkenes Using Small Molecule Photocatalysts. *Chem Rev* **2022**, *122*, 2650-2694. (d) Shang, T.-Y.; Lu, L.-H.; Cao, Z.; Liu, Y.; He, W.-M.; Yu, B. Recent advances of 1,2,3,5-tetrakis(carbazol-9-yl)-4,6-dicyanobenzene (4CzIPN) in photocatalytic transformations. *Chemical Communications* **2019**, *55*, 5408-5419. (e) Happ, B.; Friebe, C.; Winter, A.; Hager, M. D.; Hoogenboom, R.; Schubert, U. S. 2-(1 H-1,2,3-Triazol-4-yl)-Pyridine Ligands as Alternatives to 2,2′-Bipyridines in Ruthenium(II) Complexes. *Chemistry – An Asian Journal* **2009**, *4*, 154-163.
(28) Hayashi, T.; Kabeta, K.; Hamachi, I.; Kumada, M. Erythroselectivity in addition of γ-substituted allylsilanes to aldehydes in the presence of titanium chloride. *Tetrahedron Letters* **1983**, *24*, 2865-2868.
(29) Li, Z.; Wang, Z.; Zhu, L.; Tan, X.; Li, C. Silver-Catalyzed Radical Fluorination of Alkylboronates in Aqueous Solution. *Journal of the American Chemical Society* **2014**, *136*, 16439-16443.
(30) Ruan, Z.; Huang, Z.; Xu, Z.; Mo, G.; Tian, X.; Yu, X.-Y.; Ackermann, L. Catalyst-Free, Direct Electrochemical Tri- and Difluoroalkylation/Cyclization: Access to Functionalized Oxindoles and Quinolinones. *Organic Letters* **2019**, *21*, 1237-1240.
(31) Ou, W.; Zhang, G.; Wu, J.; Su, C. Photocatalytic Cascade Radical Cyclization Approach to Bioactive Indoline-Alkaloids over Donor–Acceptor Type Conjugated Microporous Polymer. *ACS Catalysis* **2019**, *9*, 5178-5183.
(32) *Gaussian 16 Rev. C.01*, Frisch, M. J.; Trucks, G. W.; Schlegel, H. B.; Scuseria, G. E.; Robb, M. A.; Cheeseman, J. R.; Scalmani, G.; Barone, V.; Petersson, G. A.; Nakatsuji, H.; Li, X.; Caricato, M.; Marenich, A. V.; Bloino, J.; Janesko, B. G.; Gomperts, R.; Mennucci, B.; Hratchian, H. P.; Ortiz, J. V.; Izmaylov, A. F.; Sonnenberg, J. L.; Williams-Young, D.; Ding, F.; Lipparini, F.; Egidi, F.; Goings, J.; Peng, B.; Petrone, A.; Henderson, T.; Ranasinghe, D.; Zakrzewski, V. G.; Gao, J.; Rega, N.; Zheng, G.;

Liang, W.; Hada, M.; Ehara, M.; Toyota, K.; Fukuda, R.; Hasegawa, J.; Ishida, M.; Nakajima, T.; Honda, Y.; Kitao, O.; Nakai, H.; Vreven, T.; Throssell, K.; Montgomery, J. A., Jr.; Peralta, J. E.; Ogliaro, F.; Bearpark, M. J.; Heyd, J. J.; Brothers, E. N.; Kudin, K. N.; Staroverov, V. N.; Keith, T. A.; Kobayashi, R.; Normand, J.; Raghavachari, K.; Rendell, A. P.; Burant, J. C.; Iyengar, S. S.; Tomasi, J.; Cossi, M.; Millam, J. M.; Klene, M.; Adamo, C.; Cammi, R.; Ochterski, J. W.; Martin, R. L.; Morokuma, K.; Farkas, O.; Foresman, J. B.; Fox, D. J. Gaussian, Inc., Wallingford, CT, 2016. (accessed 2023).
(33) Chai, J.-D.; Head-Gordon, M. Long-range corrected hybrid density functionals with damped atom–atom dispersion corrections. *Physical Chemistry Chemical Physics* **2008**, *10*, 6615-6620.
(34) (a) Weigend, F. Accurate Coulomb-fitting basis sets for H to Rn. *Physical Chemistry Chemical Physics* **2006**, *8*, 1057-1065. (b) Weigend, F.; Ahlrichs, R. Balanced basis sets of split valence, triple zeta valence and quadruple zeta valence quality for H to Rn: Design and assessment of accuracy. *Physical Chemistry Chemical Physics* **2005**, *7*, 3297-3305.
(35) Marenich, A. V.; Cramer, C. J.; Truhlar, D. G. Universal Solvation Model Based on Solute Electron Density and on a Continuum Model of the Solvent Defined by the Bulk Dielectric Constant and Atomic Surface Tensions. *The Journal of Physical Chemistry B* **2009**, *113*, 6378-6396.
(36) (a) Foster, J. P.; Weinhold, F. Natural hybrid orbitals. *Journal of the American Chemical Society* **1980**, *102*, 7211-7218. (b) Reed, A. E.; Curtiss, L. A.; Weinhold, F. Intermolecular interactions from a natural bond orbital, donor-acceptor viewpoint. *Chem Rev* **1988**, *88*, 899-926.
(37) (a) Tian, L.; Feiwu, C. Quantitative analysis of molecular surface based on improved Marching Tetrahedra algorithm. *Journal of Molecular Graphics and Modelling* **2012**, *38*, 314-323. (b) Lu, T.; Chen, F. Multiwfn: A multifunctional wavefunction analyzer. *Journal of Computational Chemistry* **2012**, *33*, 580-592.
(38) Legauklt, C. Y., *CYLview*, 1.0b, Université de Sherbrooke, **2009**. http://www.cylview.org (accessed 2023).
(39) St. John, P. C.; Guan, Y.; Kim, Y.; Kim, S.; Paton, R. S. Prediction of organic homolytic bond dissociation enthalpies at near chemical accuracy with sub-second computational cost. *Nature Communications* **2020**, *11*, 2328.
(40) Goerigk, L.; Grimme, S. A thorough benchmark of density functional methods for general main group thermochemistry, kinetics, and noncovalent interactions. *Physical Chemistry Chemical Physics* **2011**, *13*, 6670-6688.

NMR Spectra

^{1}**H-NMR of 1a** (400 MHz, $CDCl_3$)

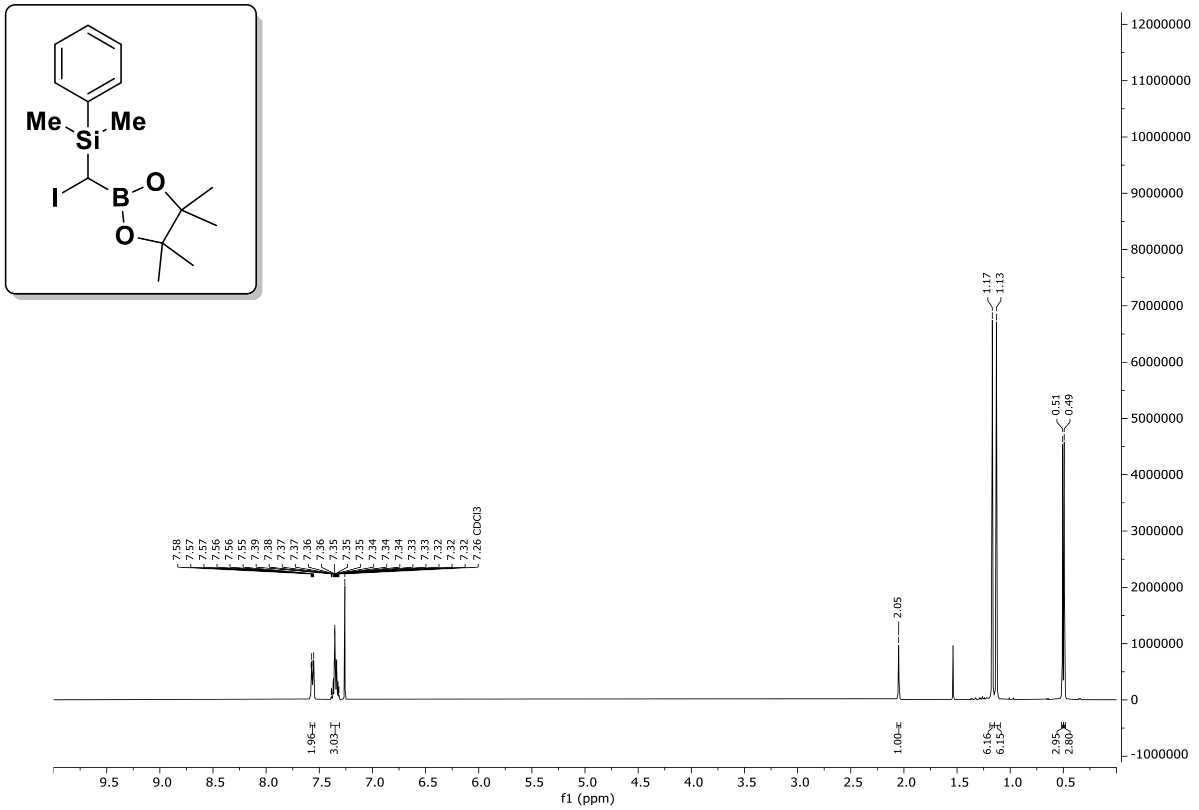

^{1}**H-NMR of S2** (400 MHz, $CDCl_3$)

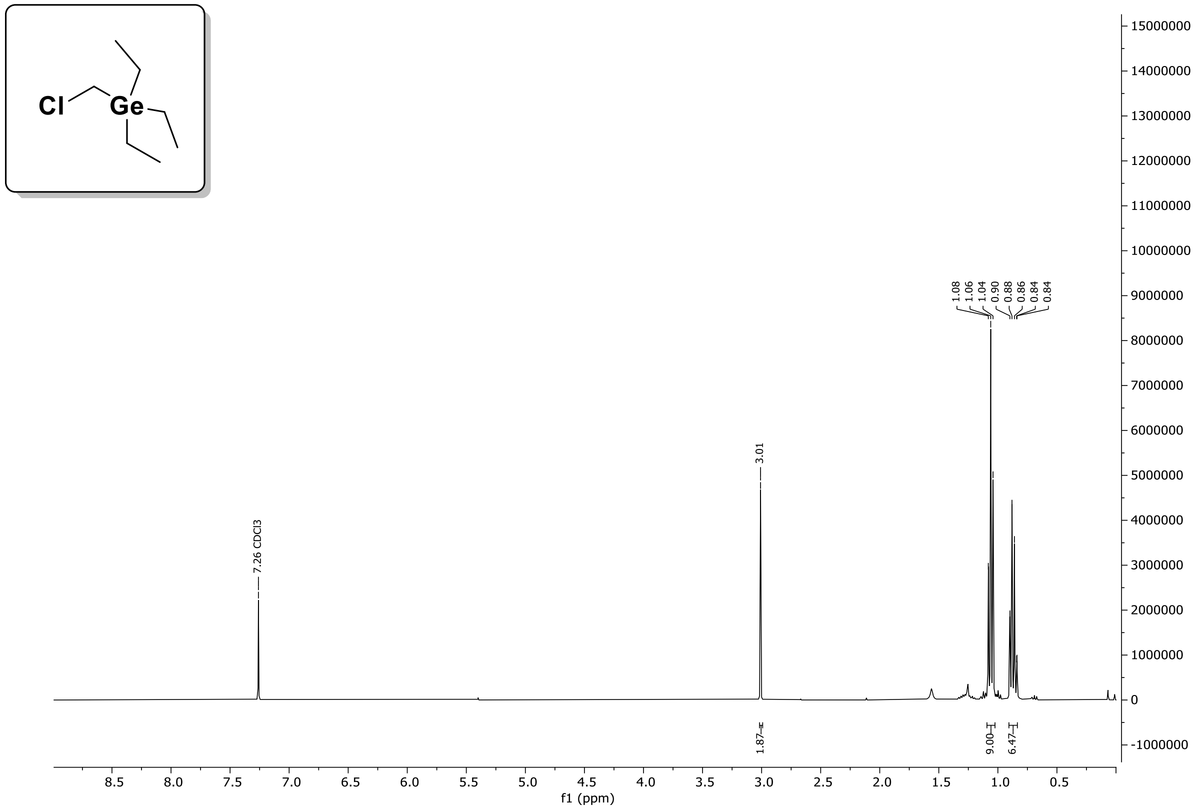

^{1}H-NMR of 1b (400 MHz, $CDCl_3$)

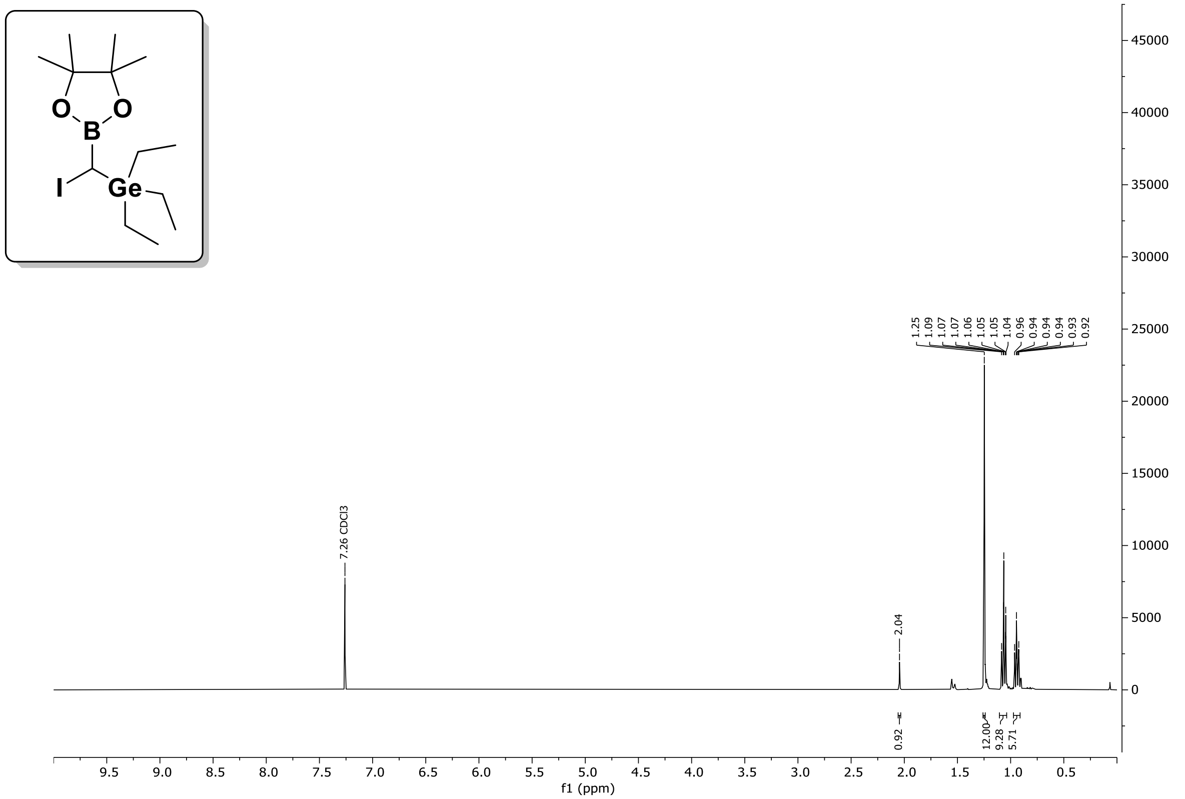

^{1}H-NMR of S3 (400 MHz, $CDCl_3$)

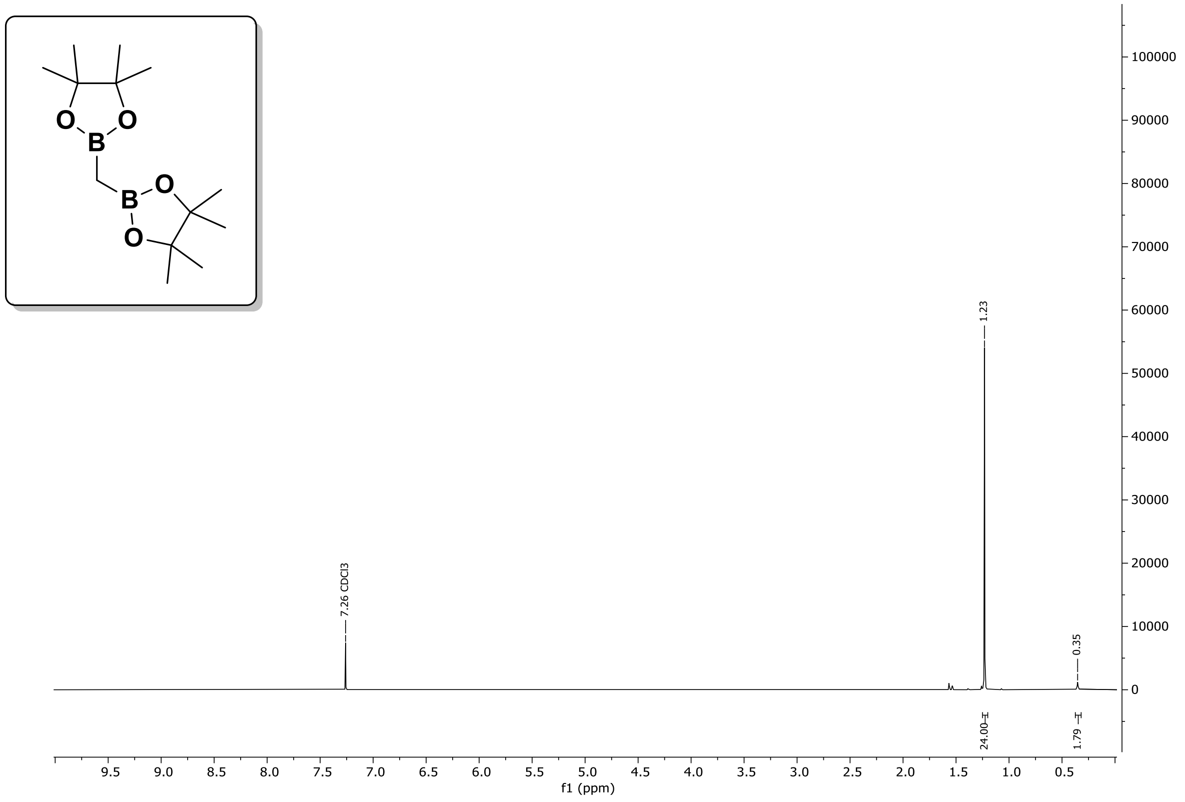

^{1}H-NMR of 1c (400 MHz, $CDCl_3$)

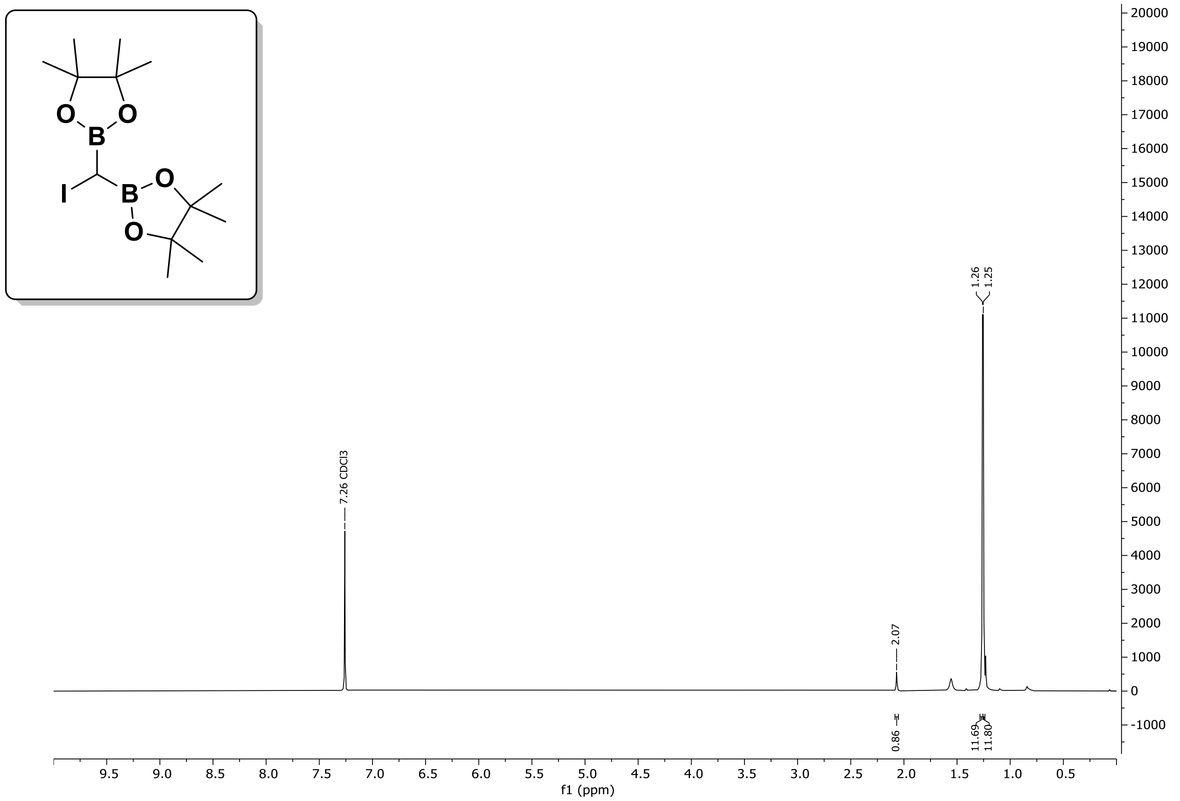

^{1}H-NMR of 1d (400 MHz, $CDCl_3$)

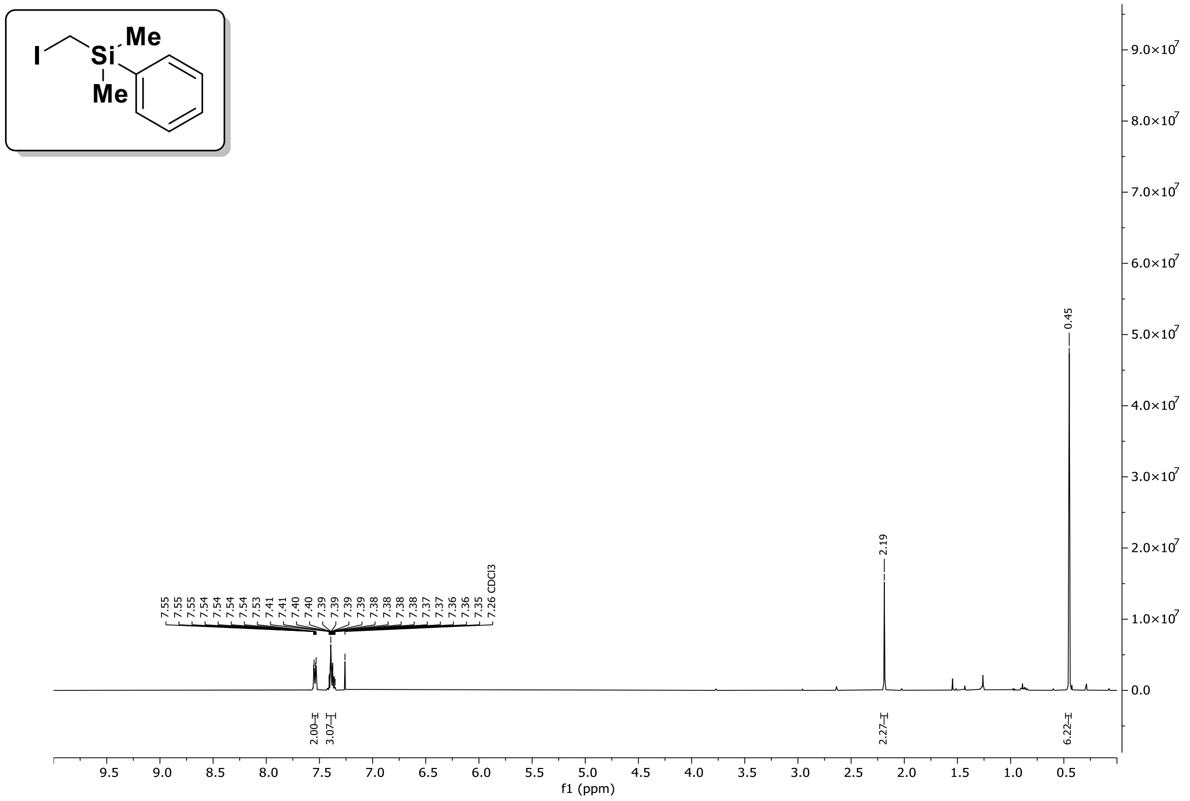

^{1}H-NMR of 1e (400 MHz, $CDCl_3$)

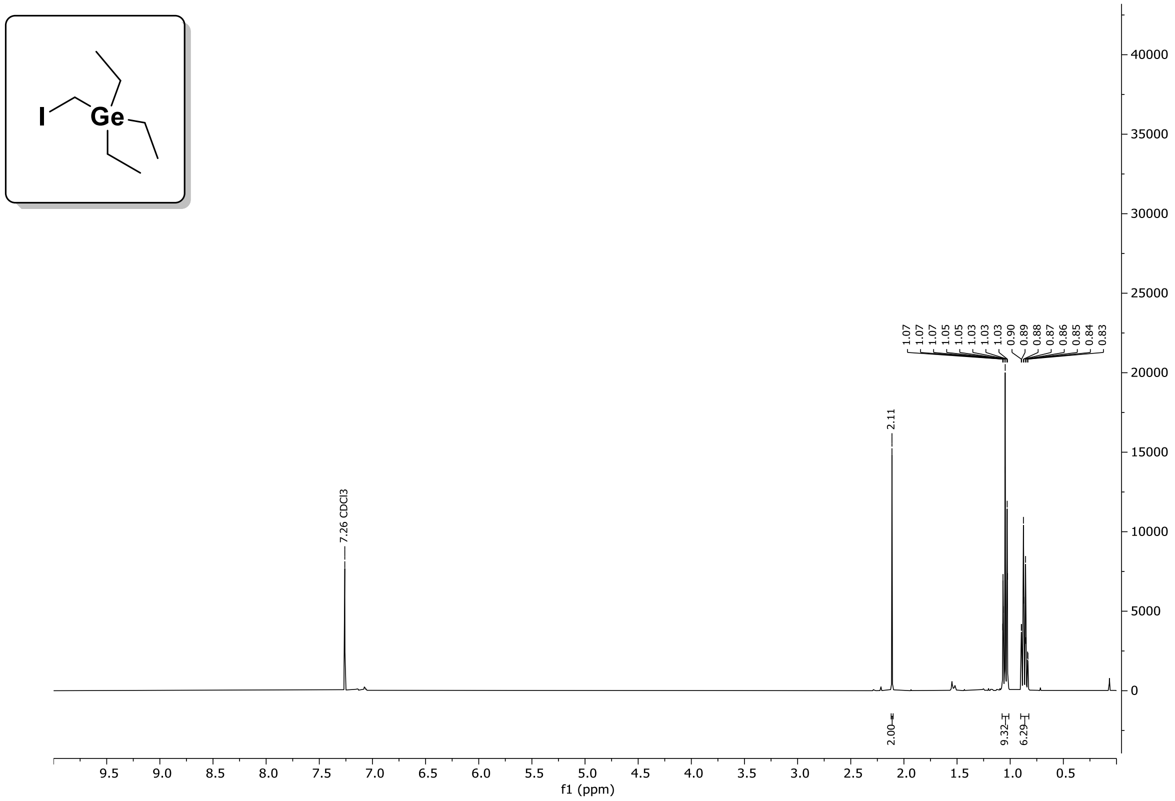

^{13}C-NMR of 1e (101 MHz, $CDCl_3$)

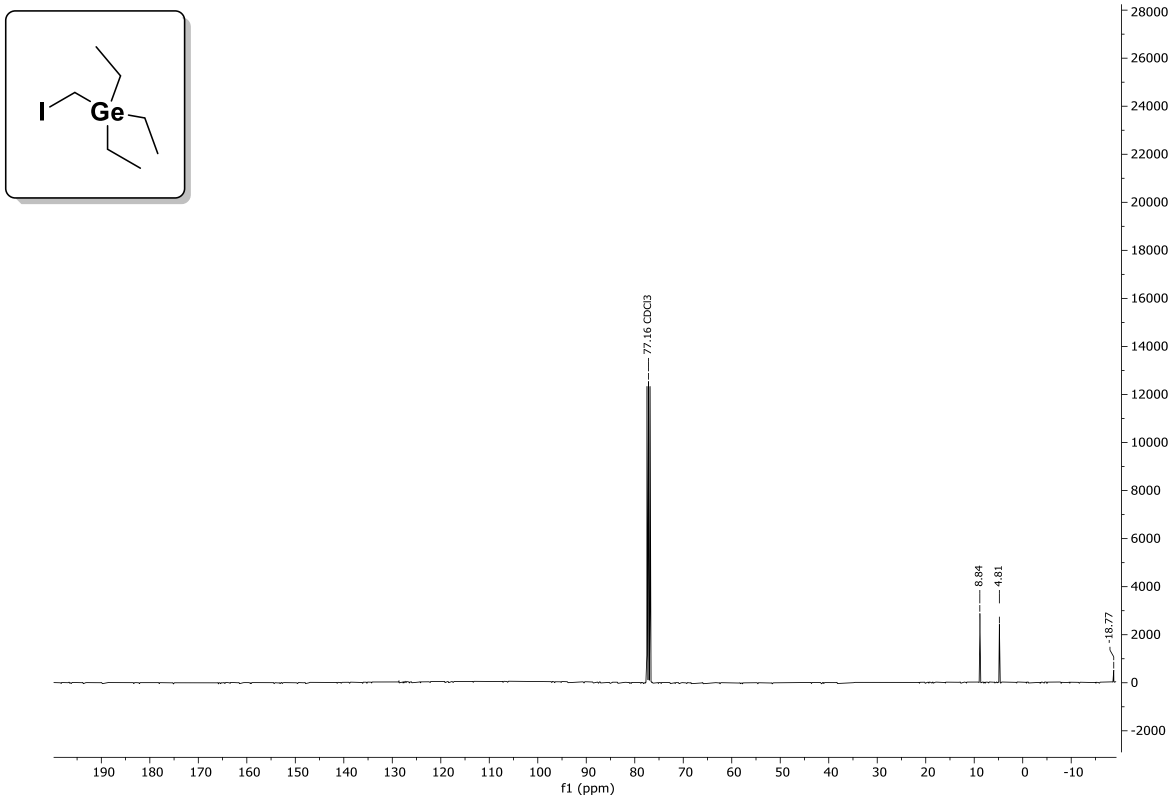

^{1}H-NMR of S4 (400 MHz, $CDCl_3$)

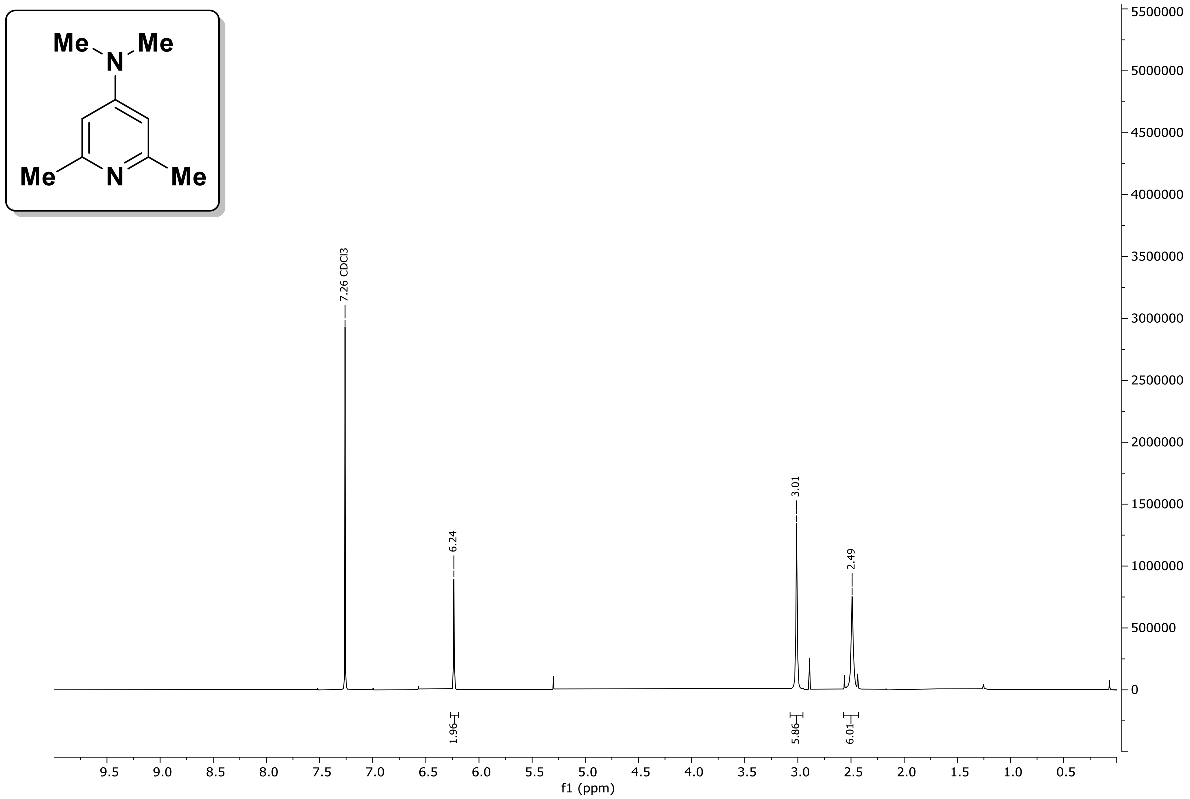

^{1}H-NMR of S5 (400 MHz, $CDCl_3$)

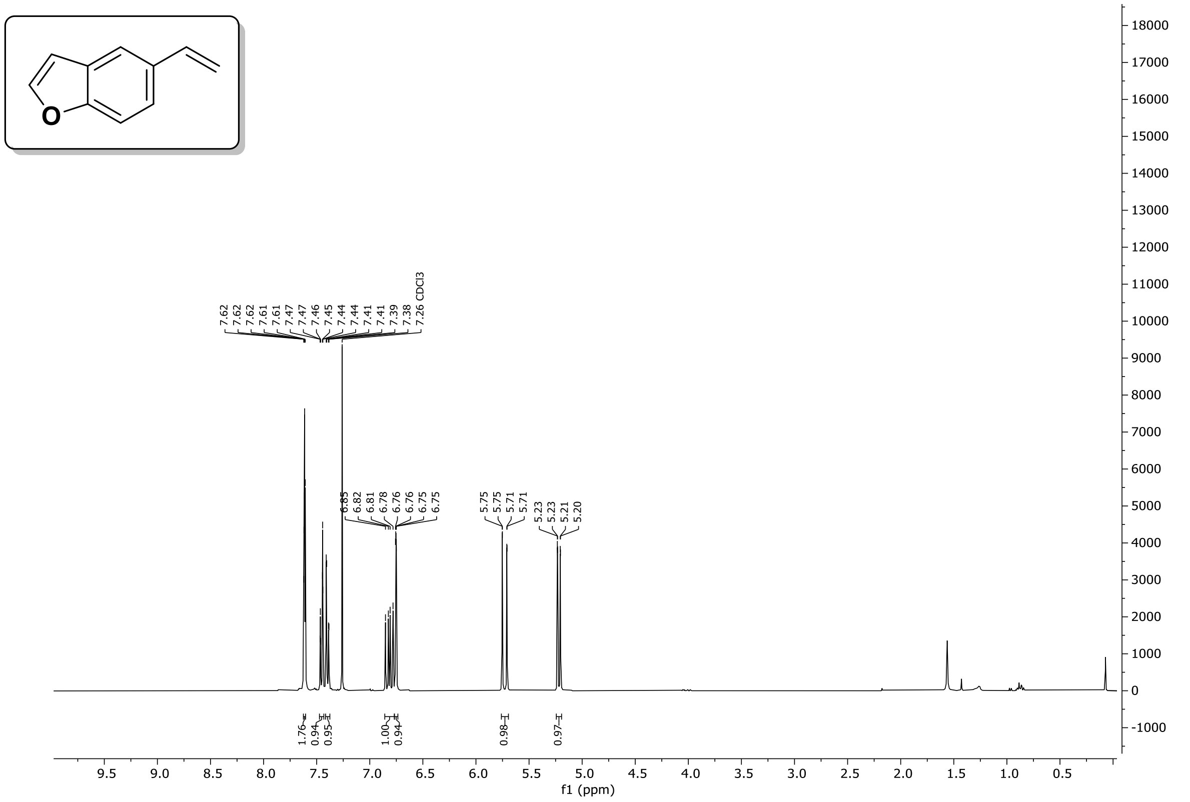

^{1}H-NMR of S6 (400 MHz, $CDCl_3$)

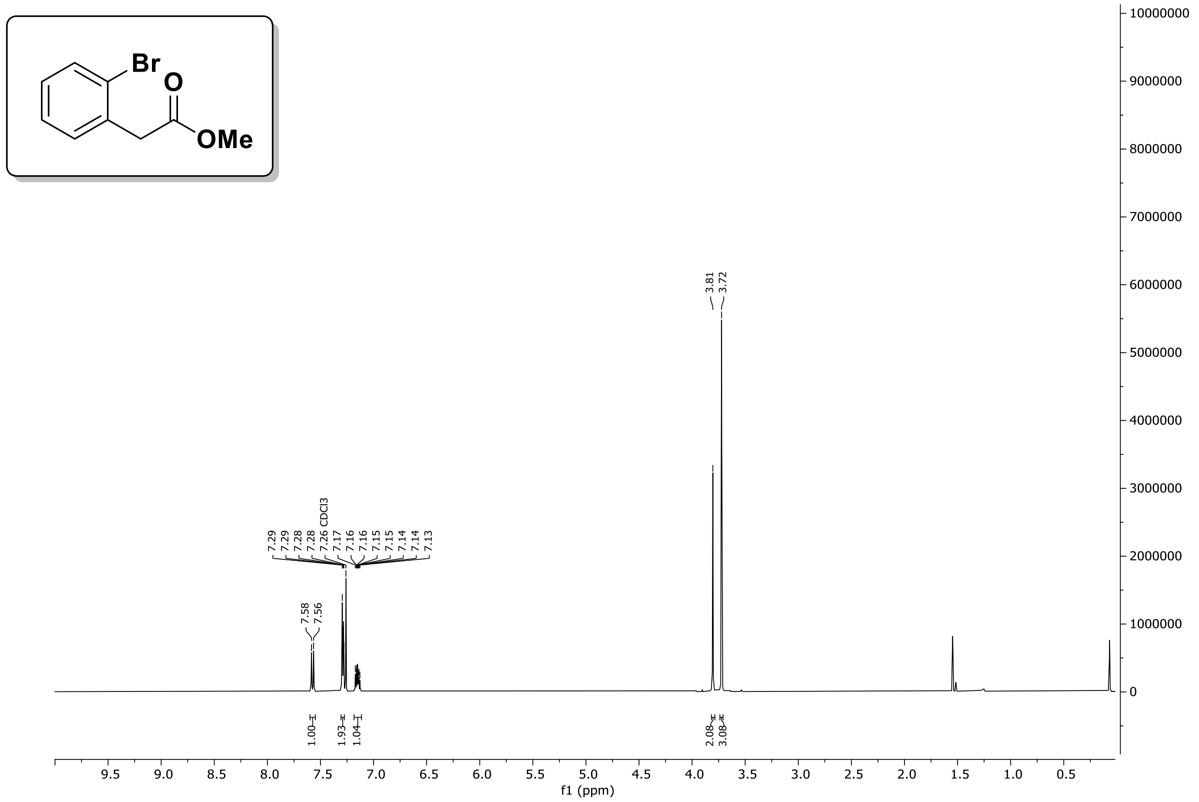

^{1}H-NMR of S7 (400 MHz, $CDCl_3$)

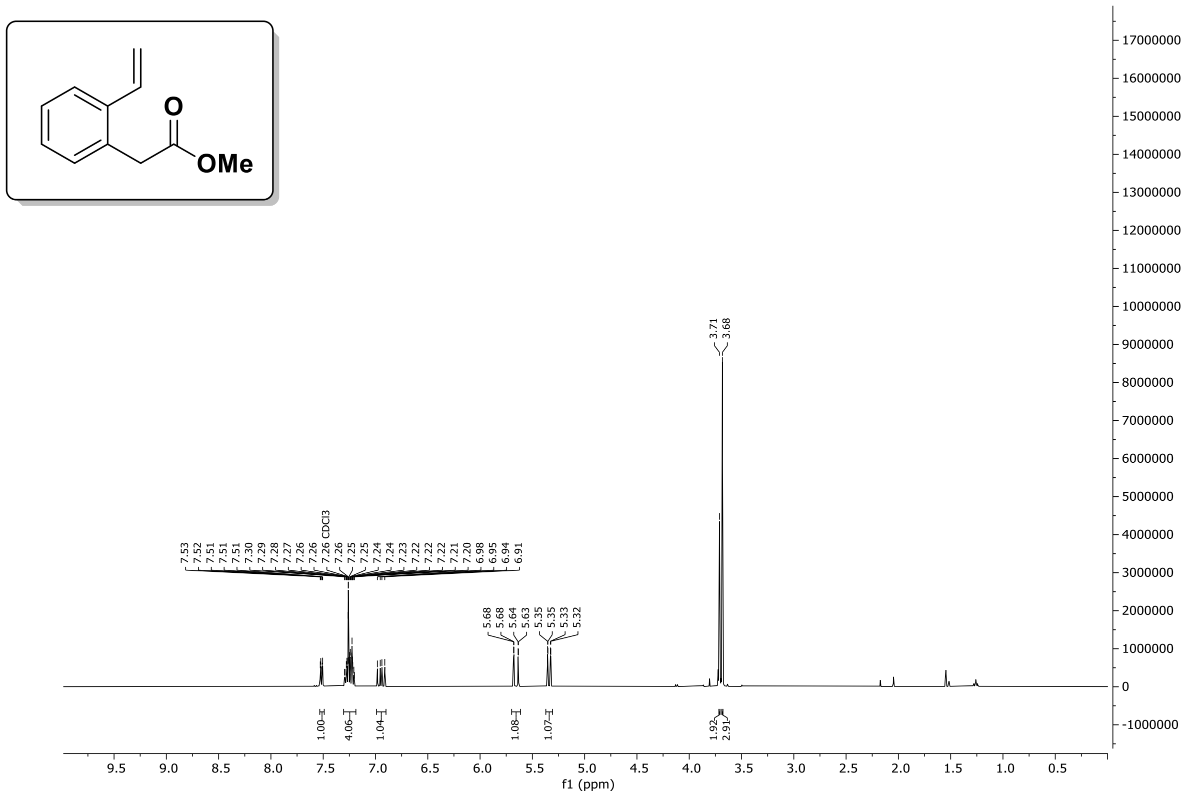

^{1}H-NMR of S8 (400 MHz, $CDCl_3$)

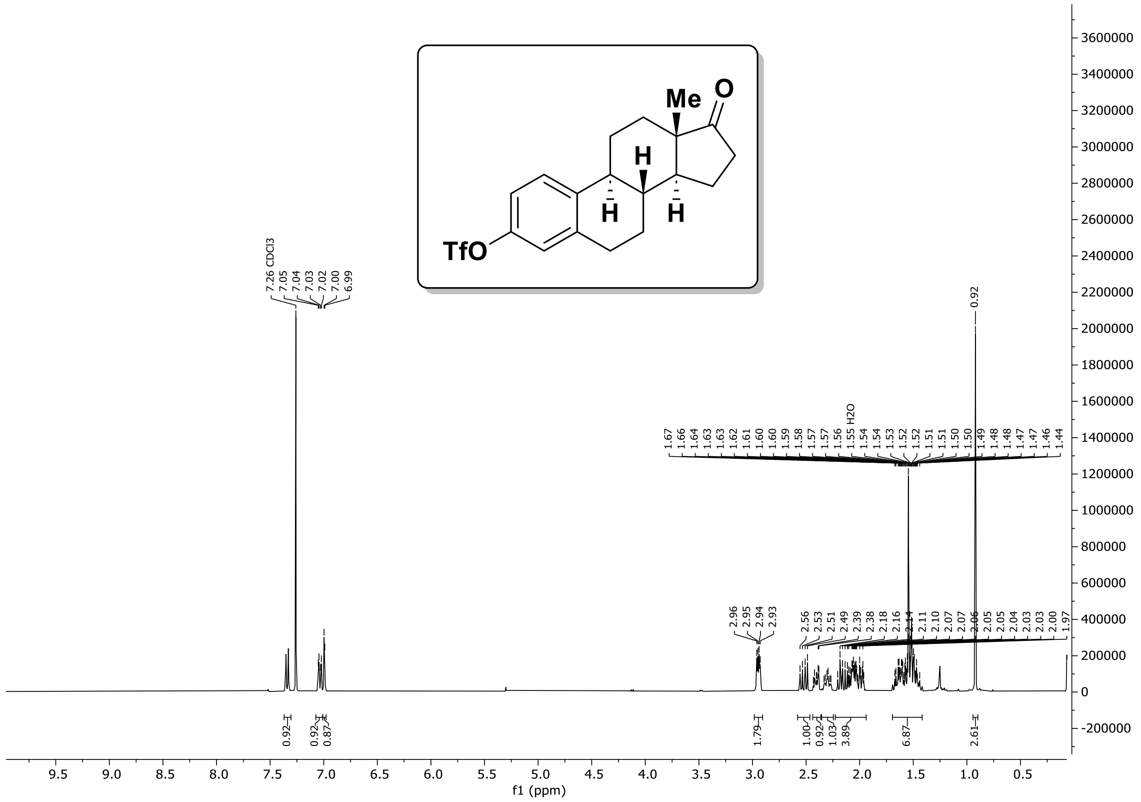

^{1}H-NMR of S9 (400 MHz, $CDCl_3$)

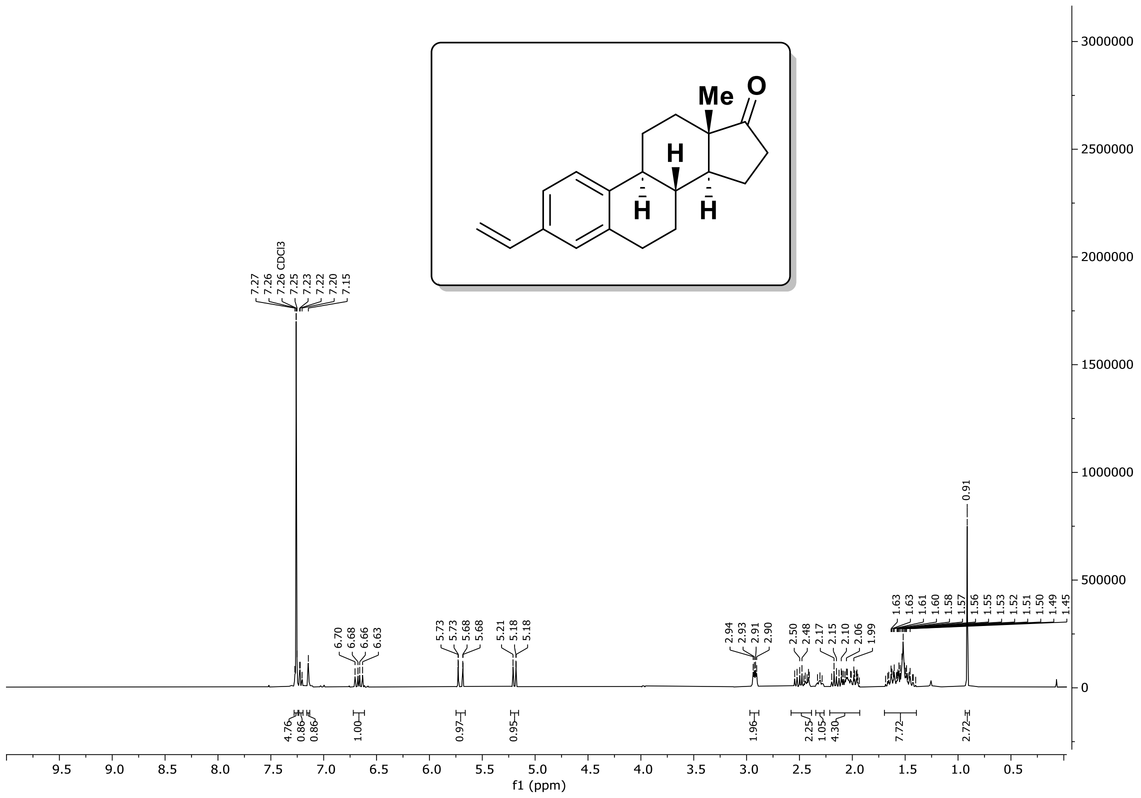

^{1}H-NMR of S10 (400 MHz, DMSO-d_6)

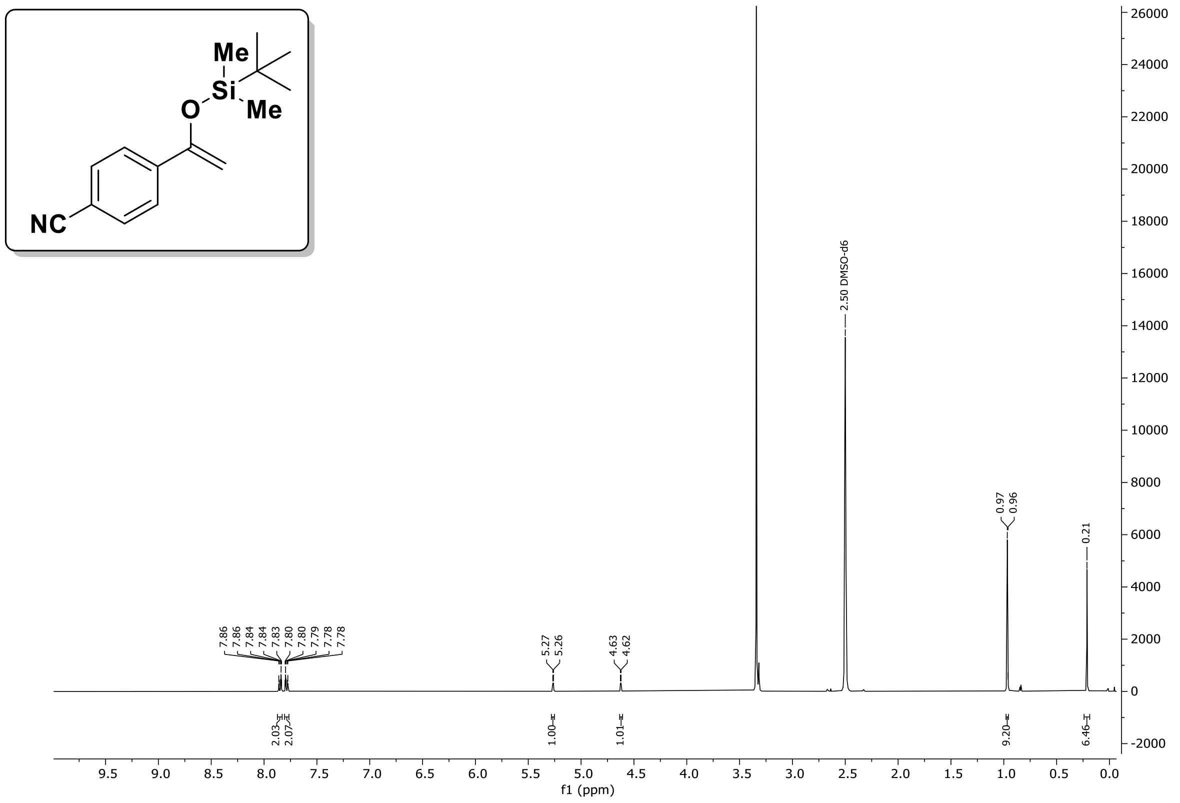

^{1}H-NMR of S11 (400 MHz, DMSO-d_6)

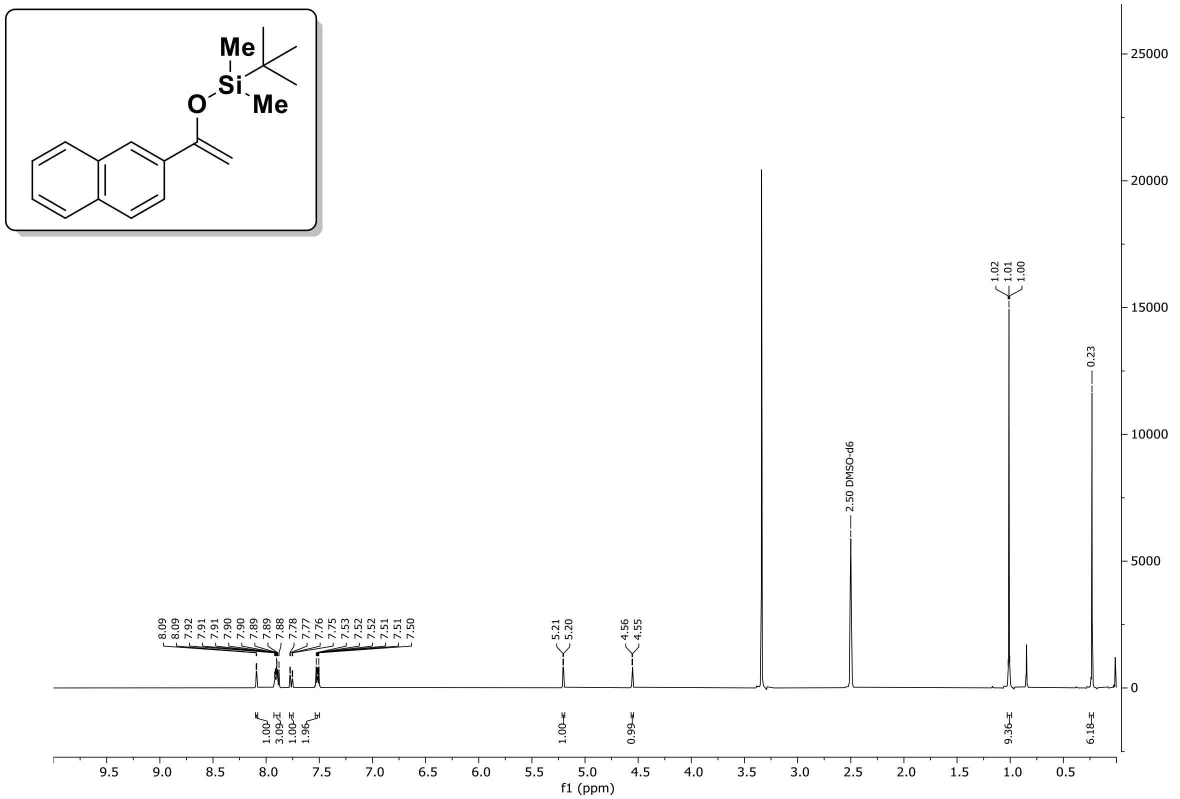

^{1}H-NMR of S12 (400 MHz, DMSO-d_6)

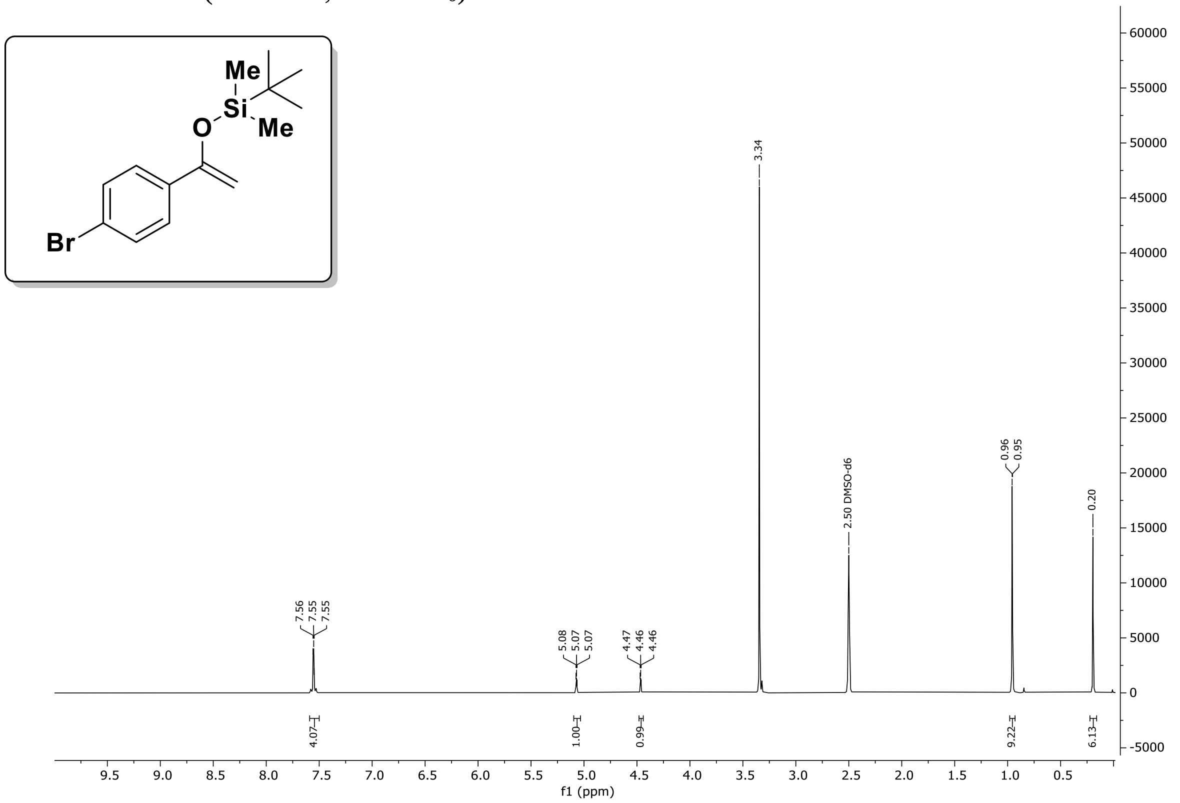

^{1}H-NMR of S13 (400 MHz, DMSO-d_6)

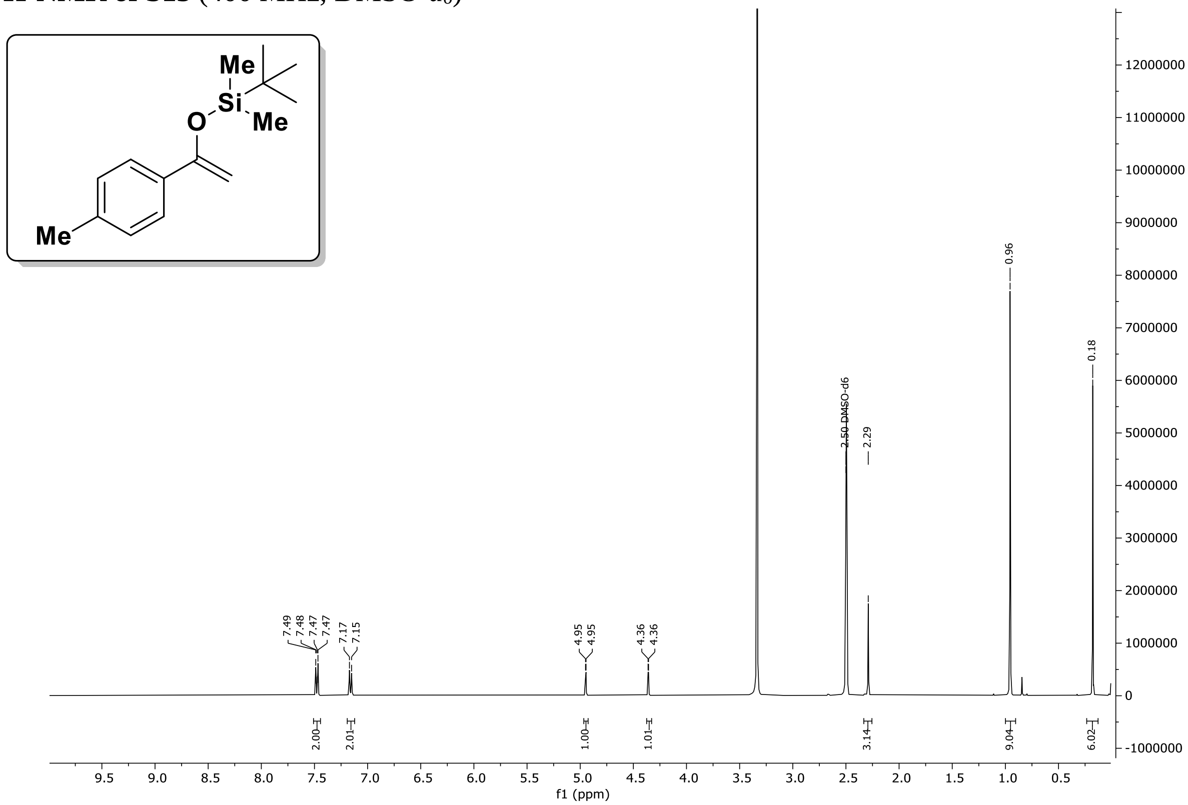

^{1}H NMR of S14 (400 MHz, $CDCl_3$)

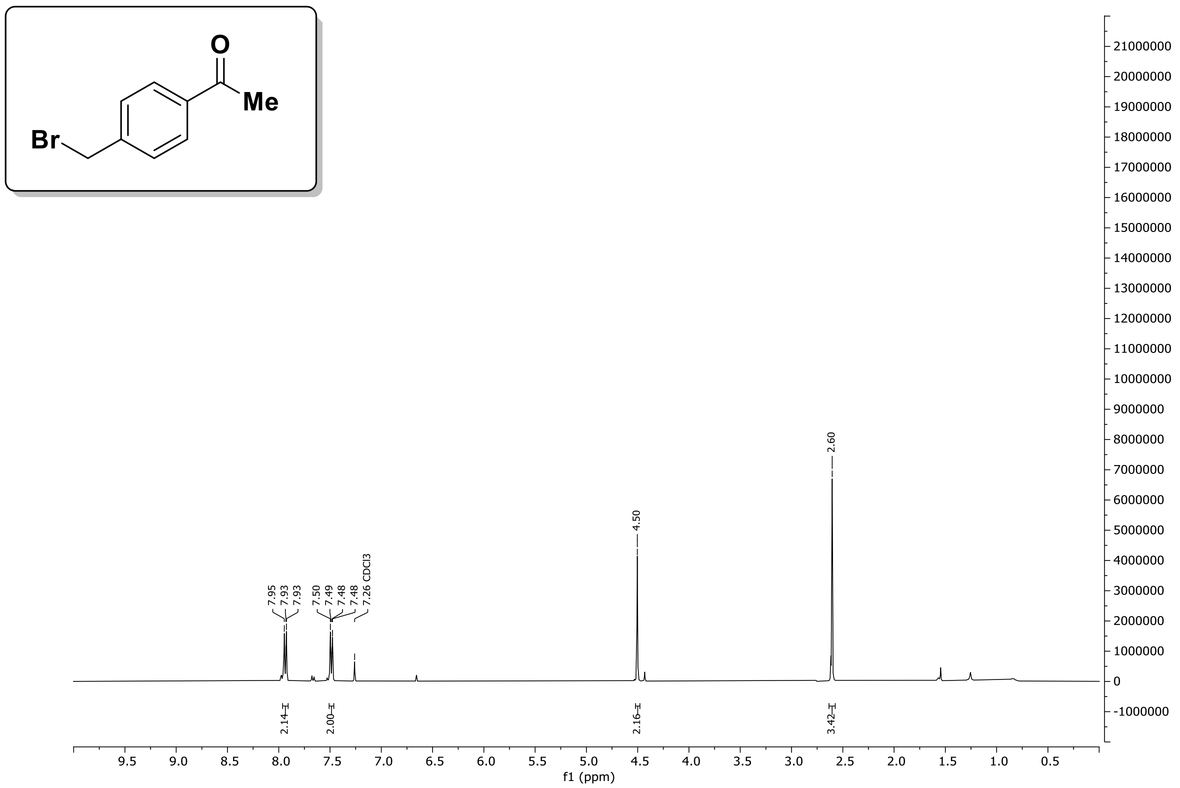

^{1}H NMR of S15 (400 MHz, $CDCl_3$)

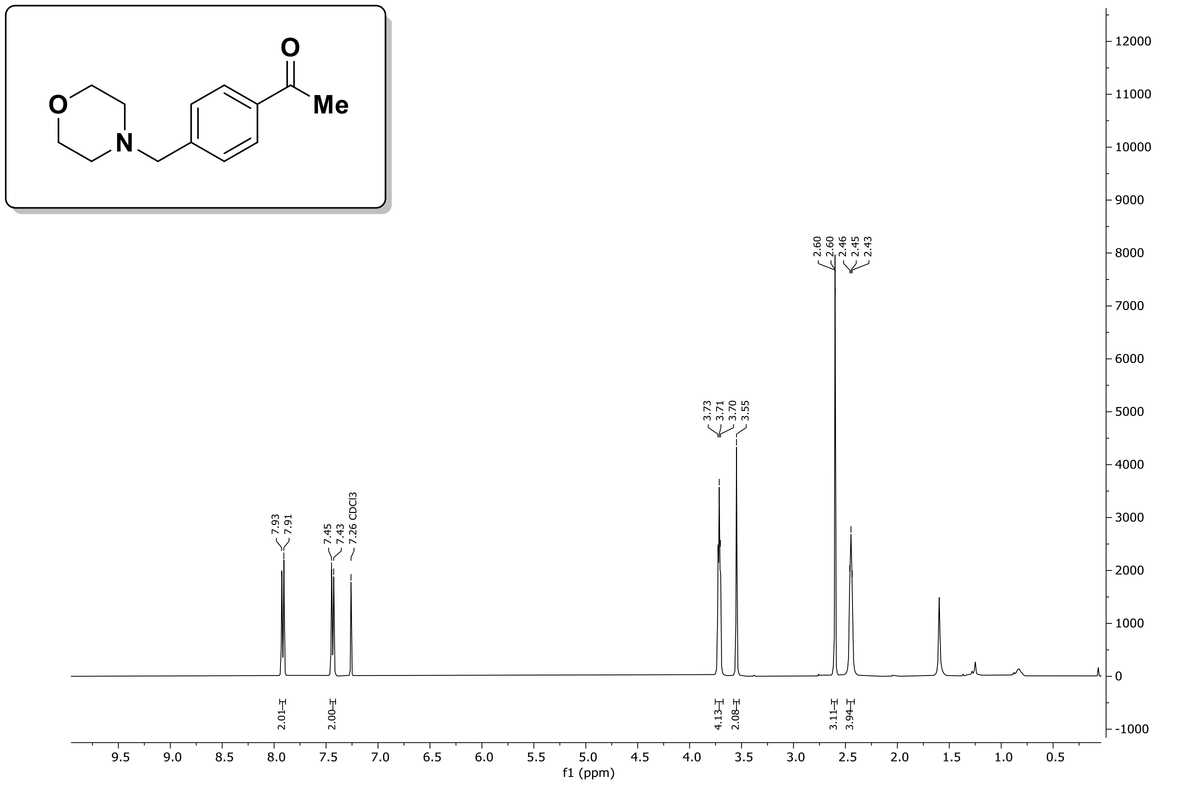

^{1}H-NMR of S16 (400 MHz, DMSO)

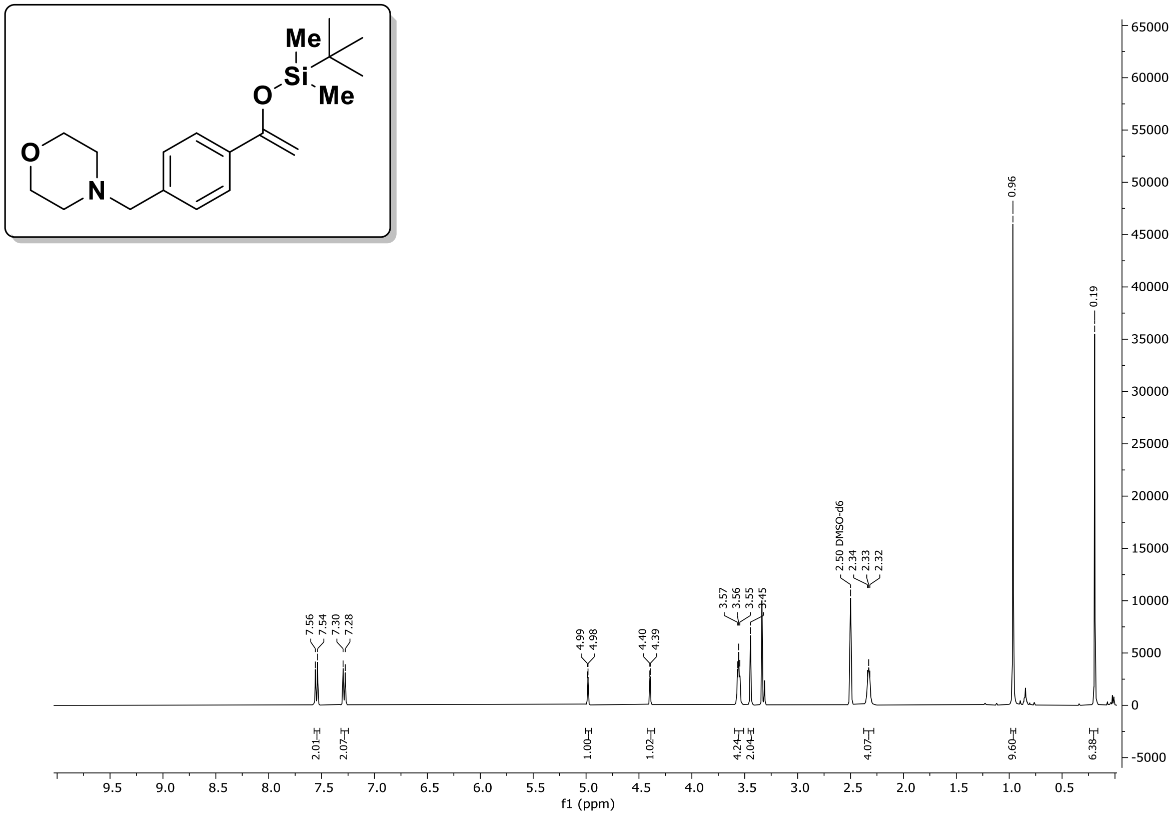

^{13}C NMR of S16 (101 MHz, DMSO)

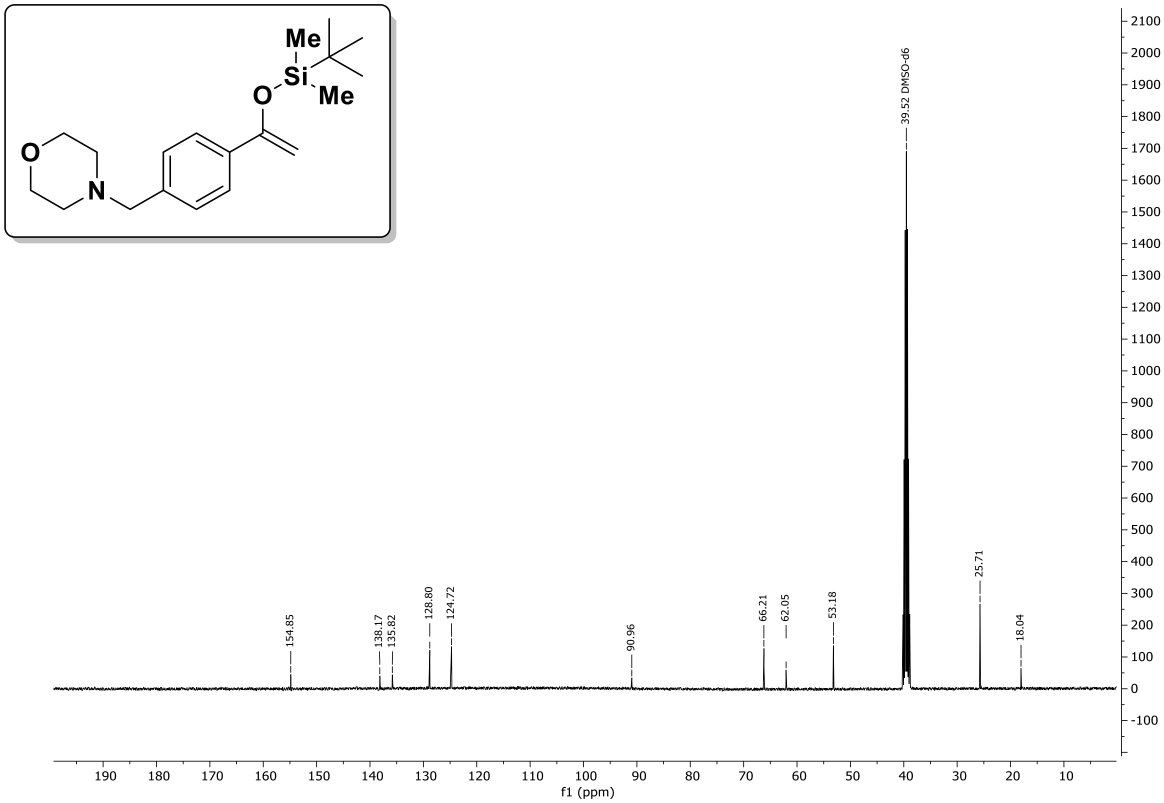

^{1}H NMR of S17 (400 MHz, DMSO)

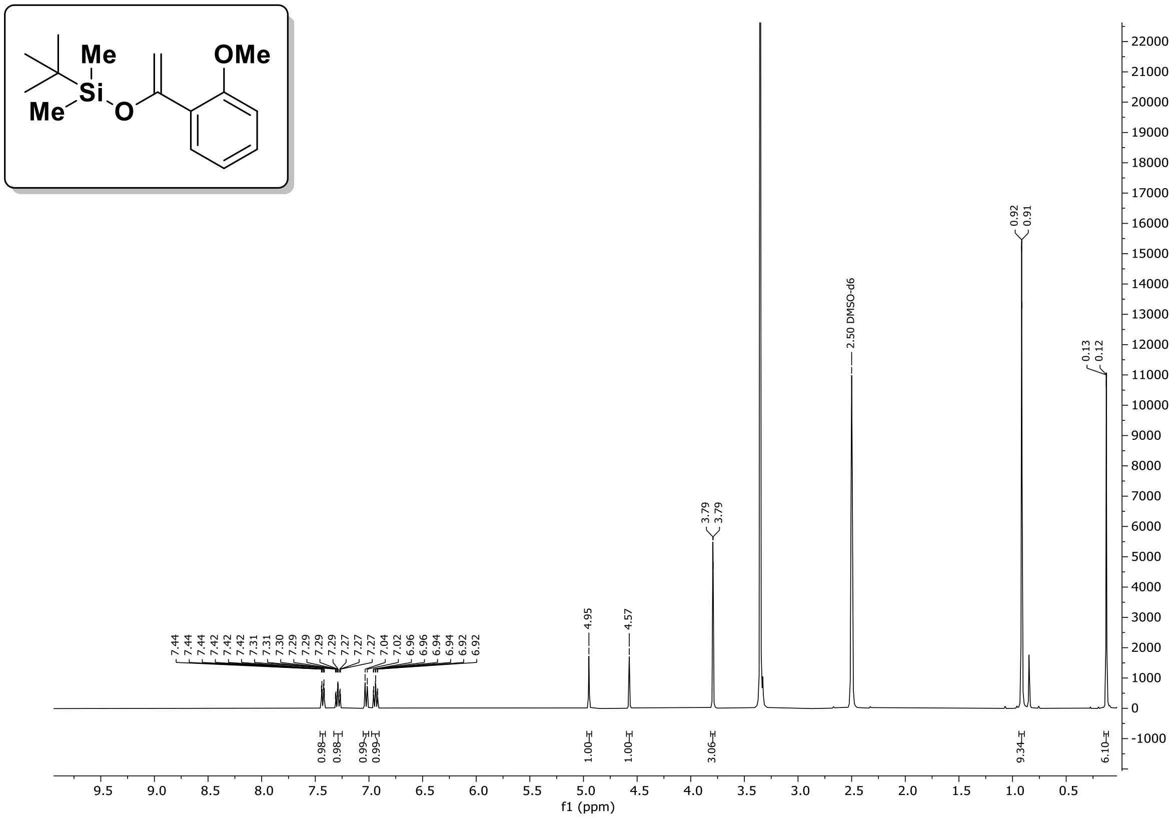

^{1}H NMR of S18 (400 MHz, DMSO)

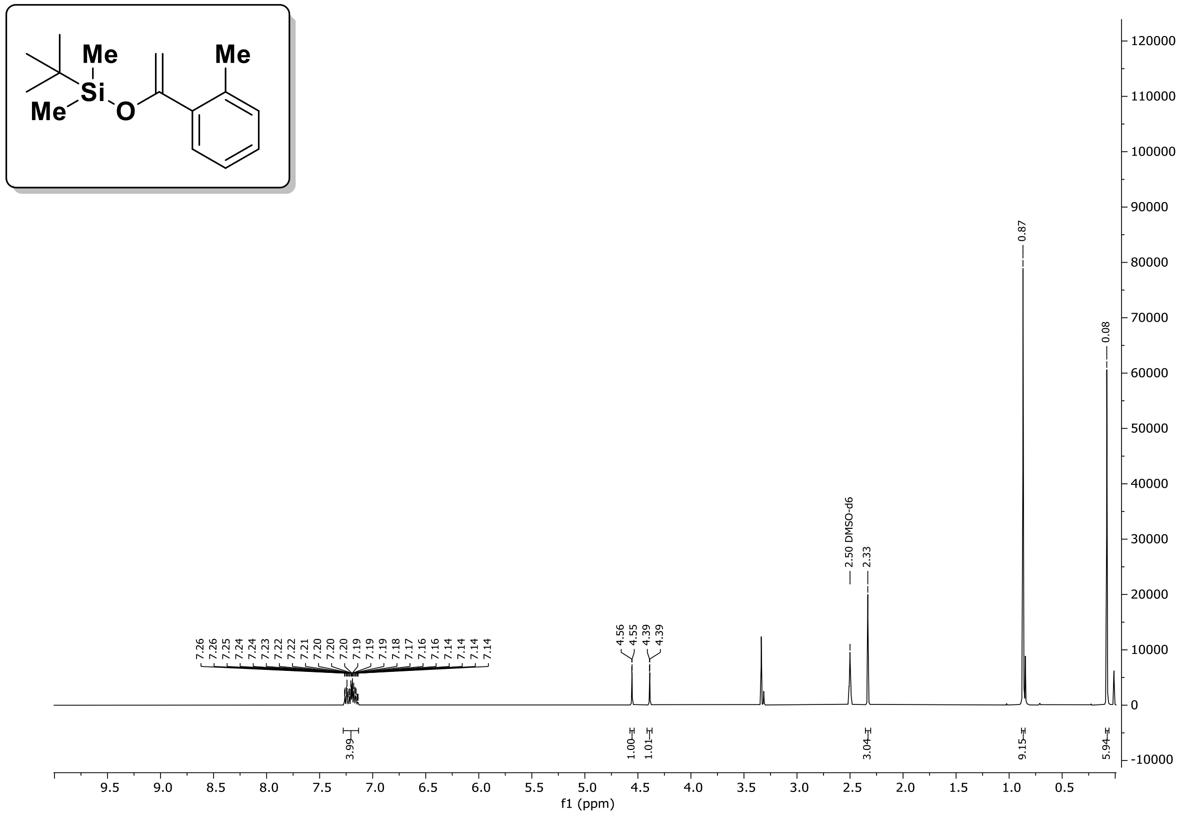

^{13}C NMR of S18 (101 MHz, DMSO)

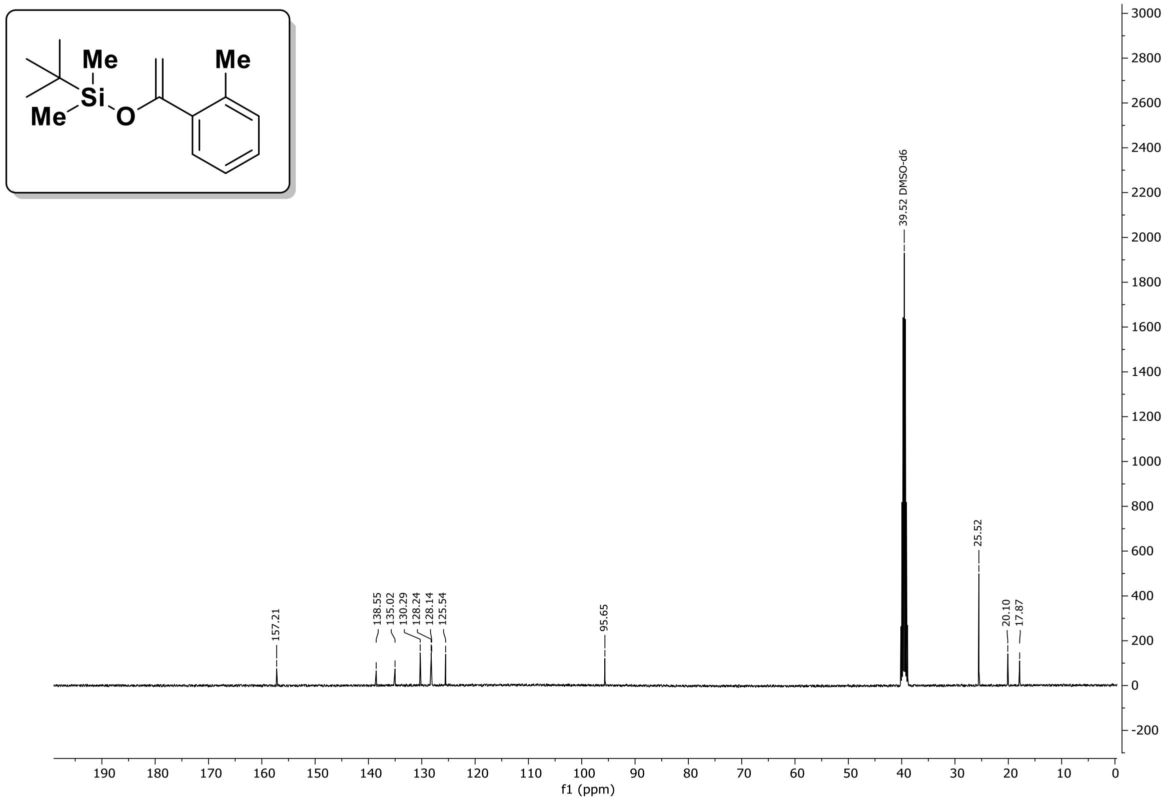

^{1}H NMR of S19 (400 MHz, CD_3CN)

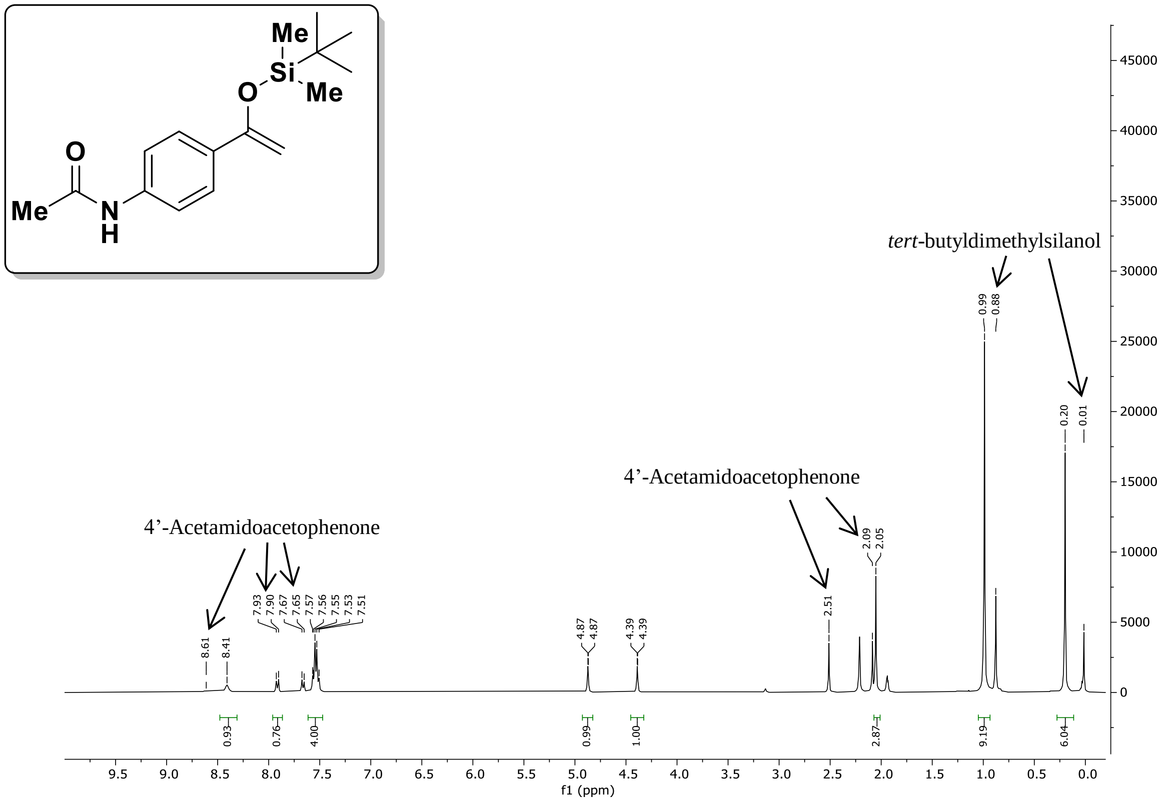

^{13}C NMR of S19 (101 MHz, CD_3CN)

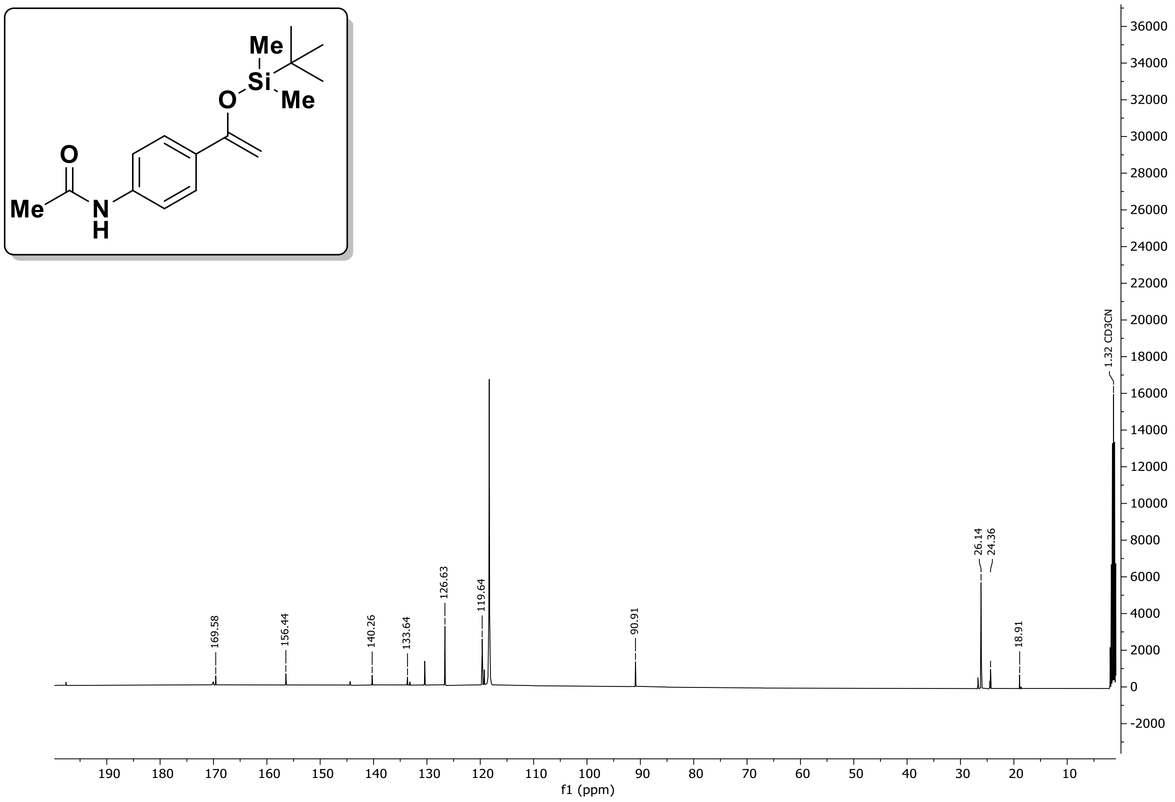

^{1}H NMR of S20 (400 MHz, CD_3CN)

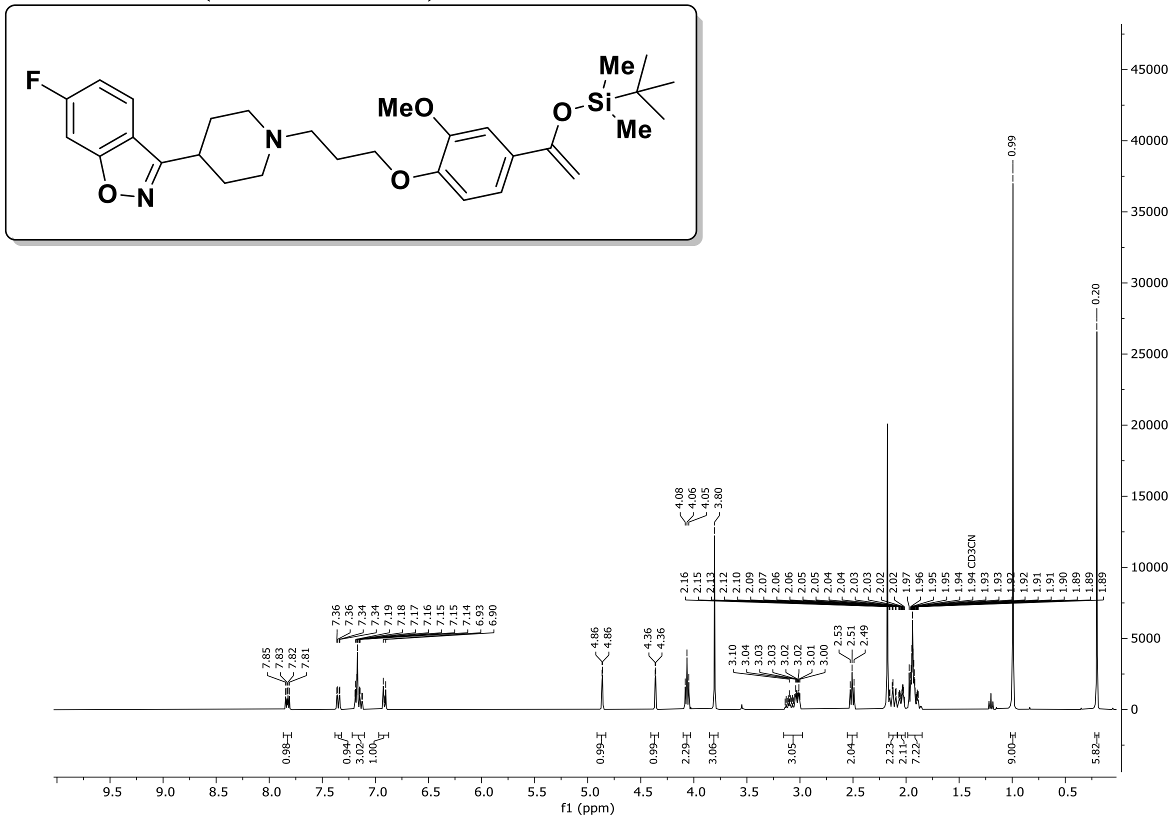

^{13}C NMR of S20 (101 MHz, CD_3CN)

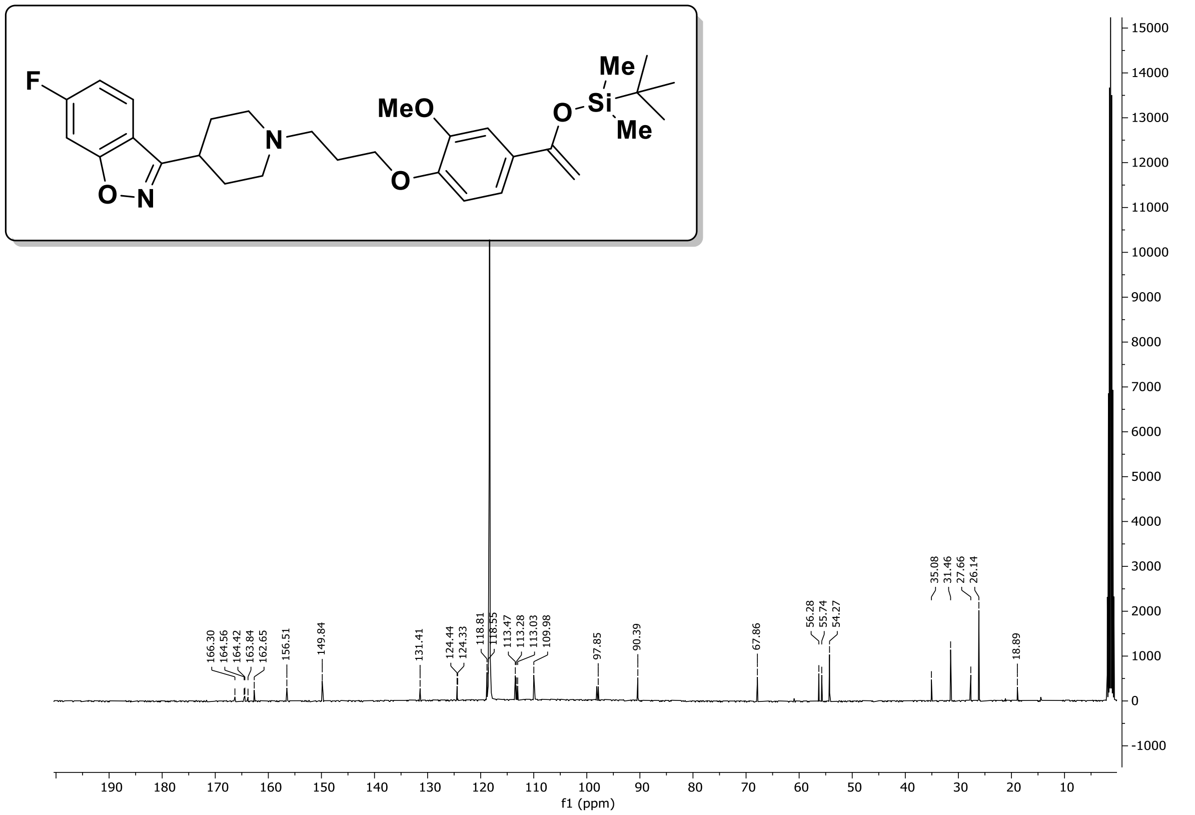

^{19}F NMR of S20 (564 MHz, CD_3CN)

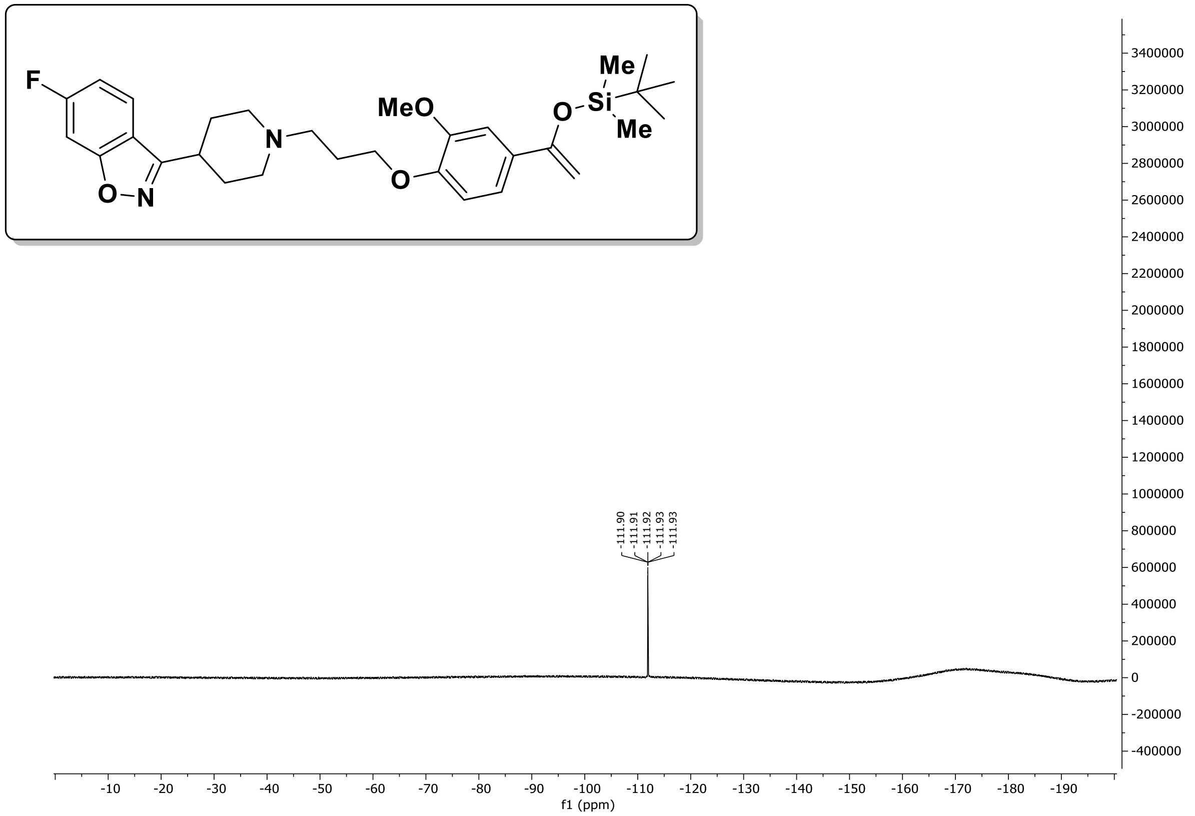

^{1}H-NMR of S21 (400 MHz, DMSO-d_6)

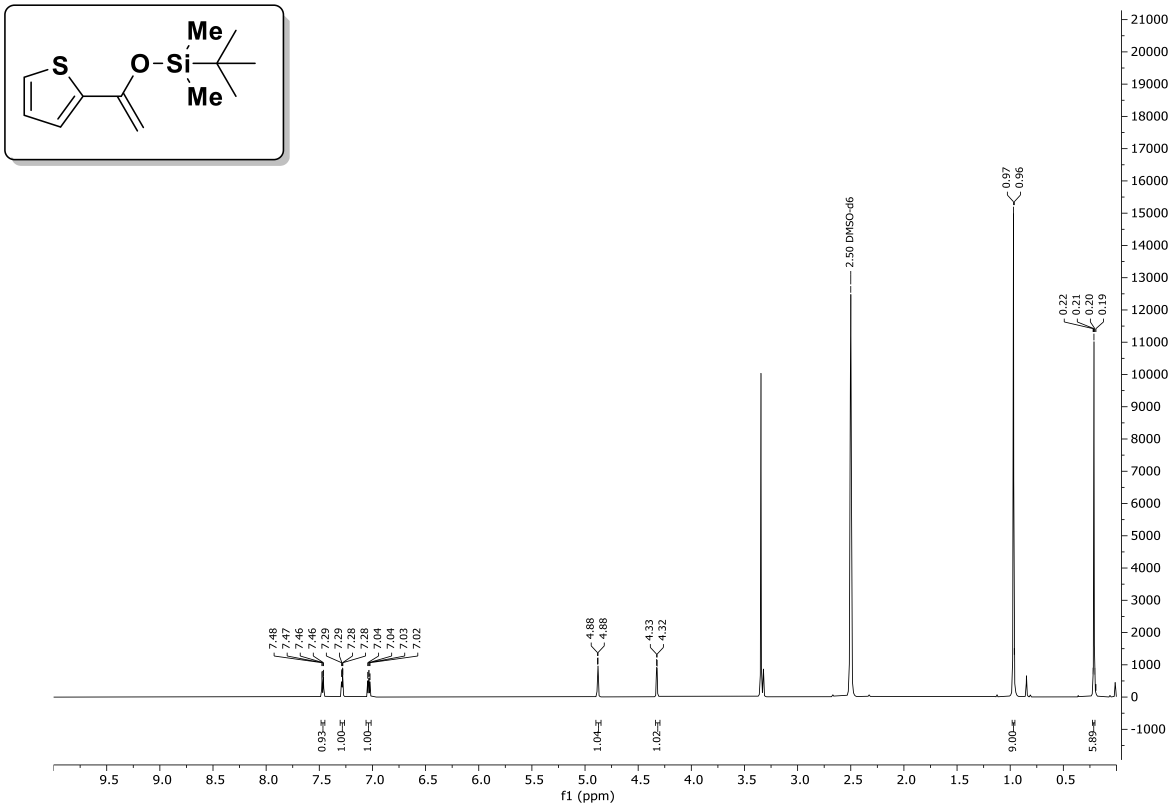

^{1}H-NMR of S22 (400 MHz, $CDCl_3$)

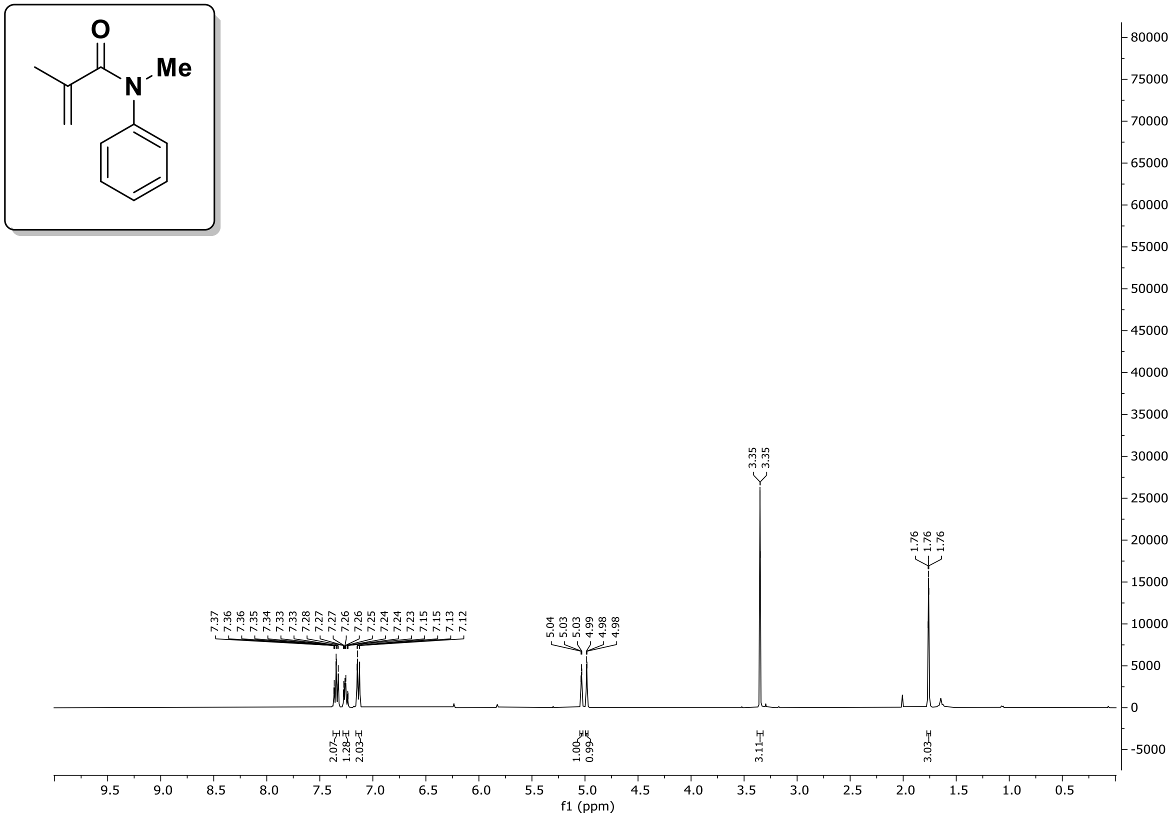

^{1}H-NMR of 3 (400 MHz, $CDCl_3$)

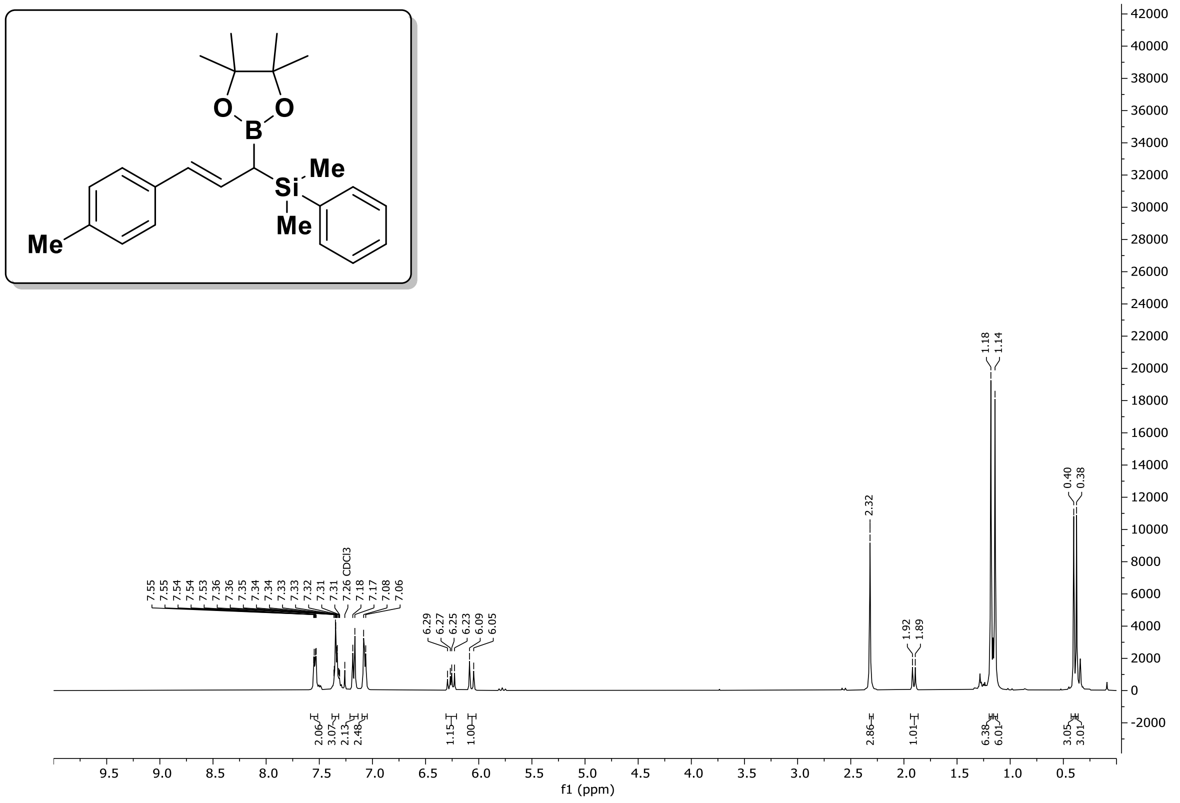

^{13}C-NMR of 3 (101 MHz, $CDCl_3$)

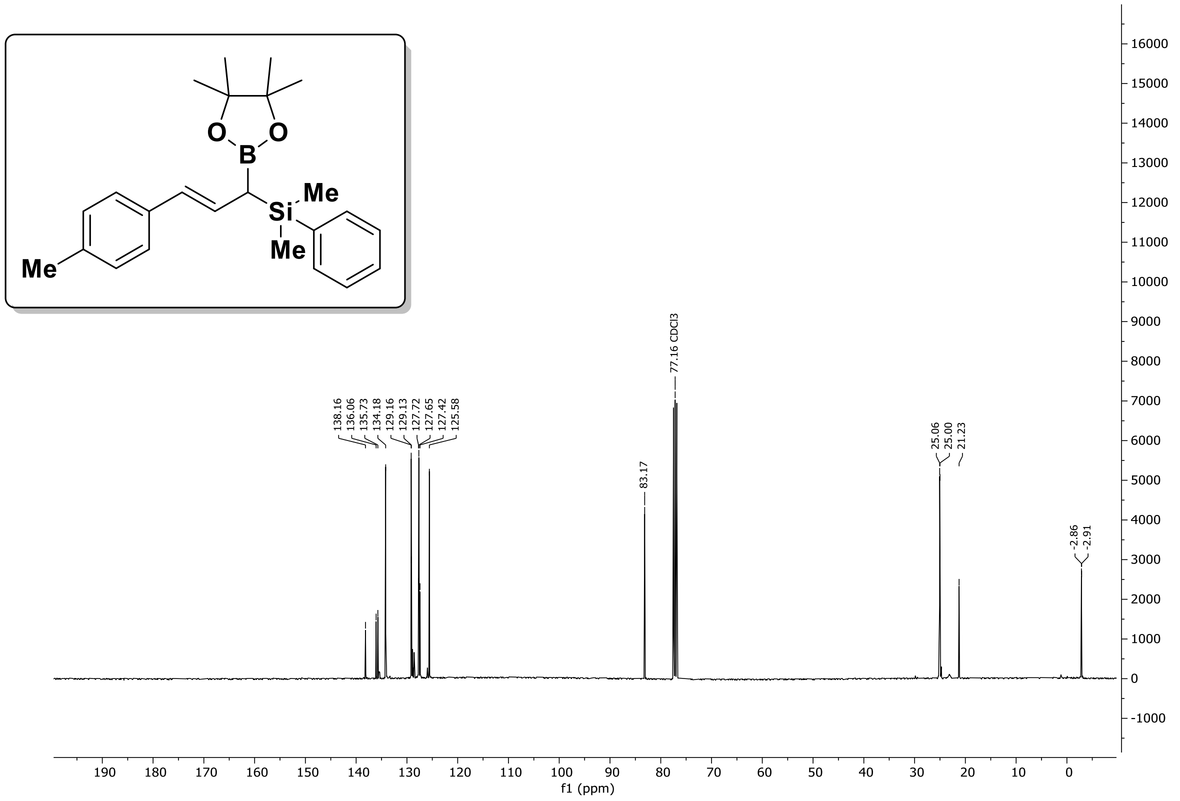

^{1}H-NMR of 4 (400 MHz, $CDCl_3$)

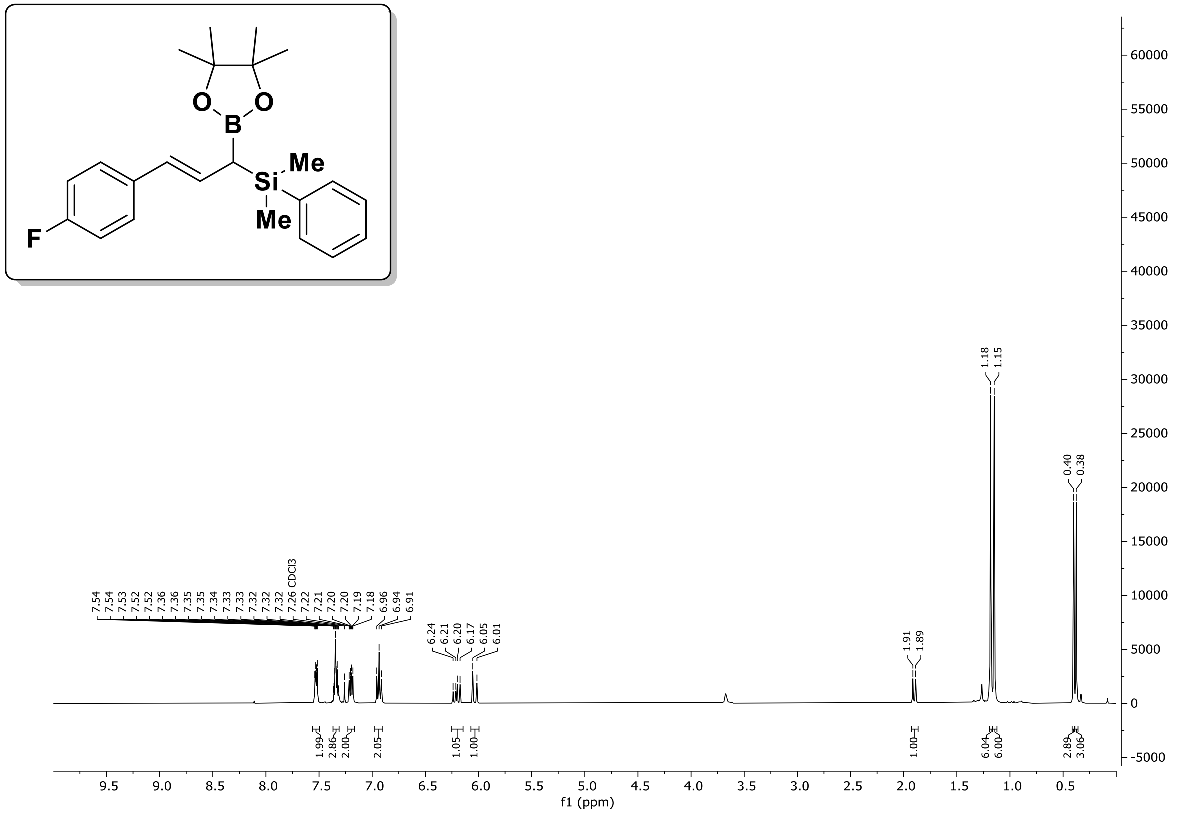

^{13}C-NMR of 4 (101 MHz, $CDCl_3$)

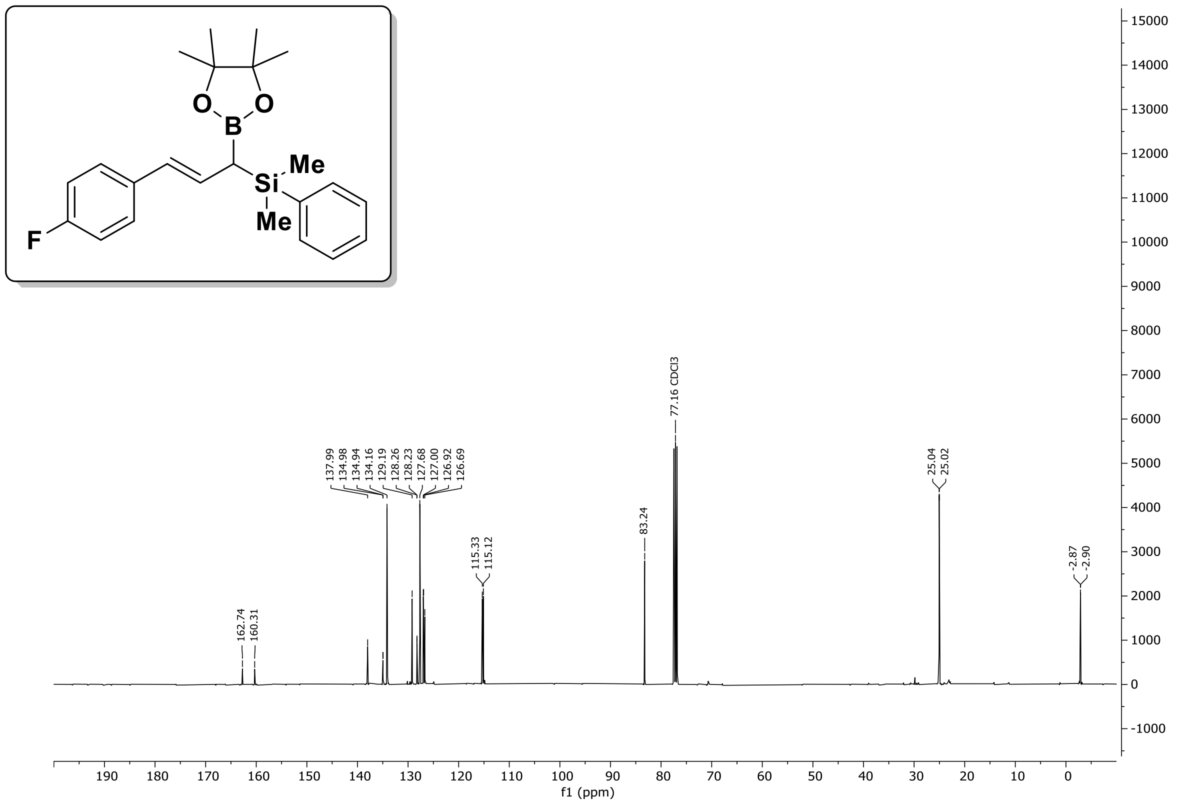

^{19}F NMR of 4 (376 MHz, $CDCl_3$)

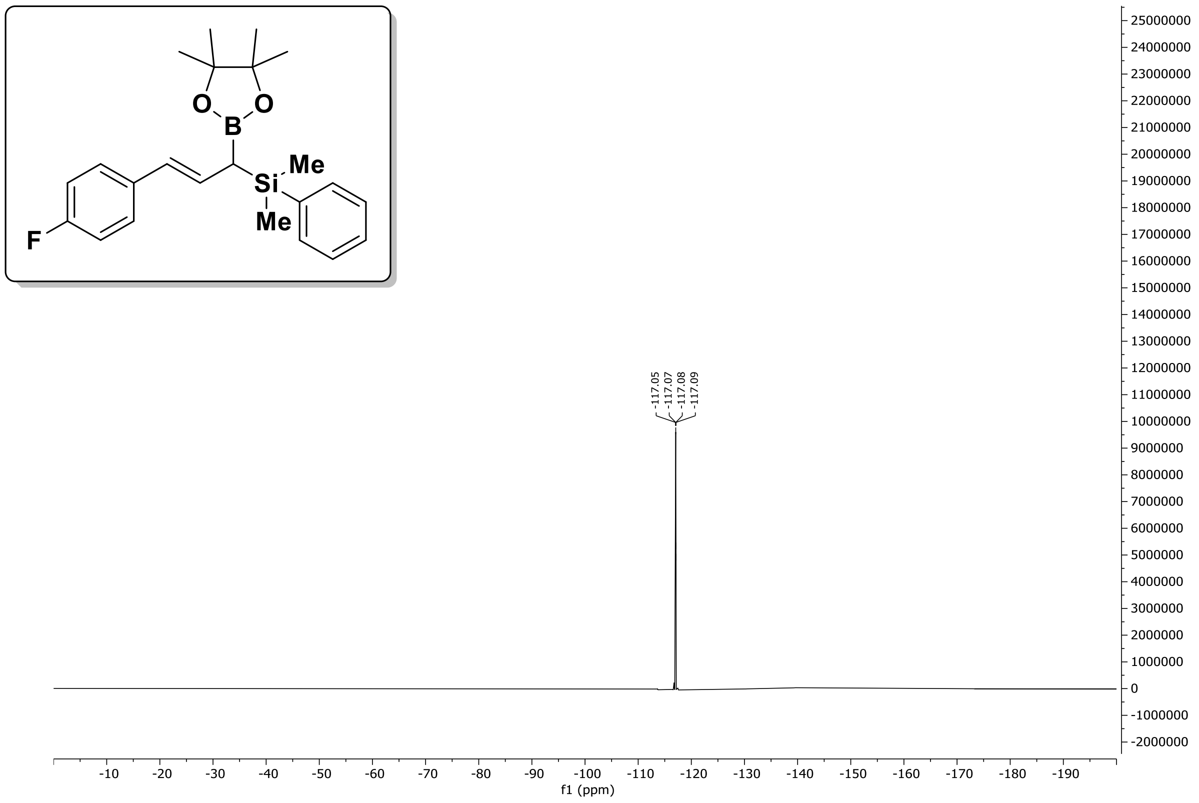

^{1}H-NMR of 5 (400 MHz, $CDCl_3$)

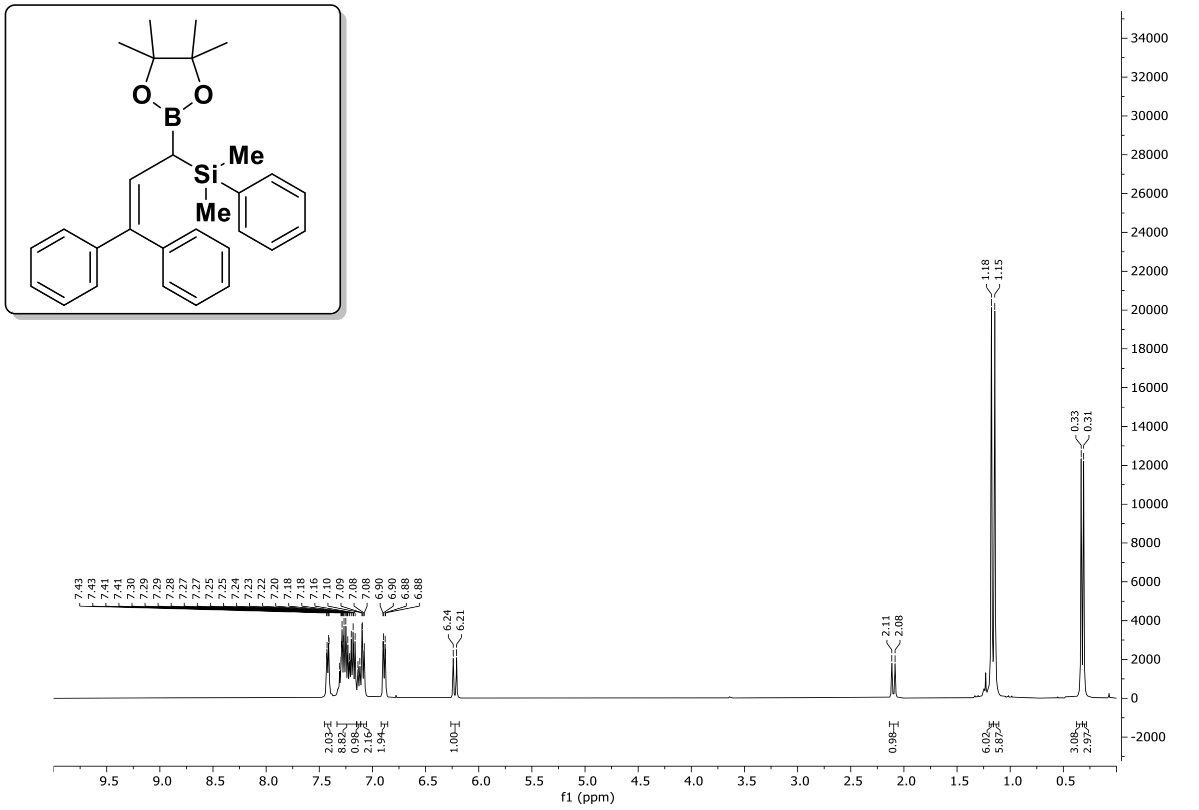

^{13}C-NMR of 5 (101 MHz, $CDCl_3$)

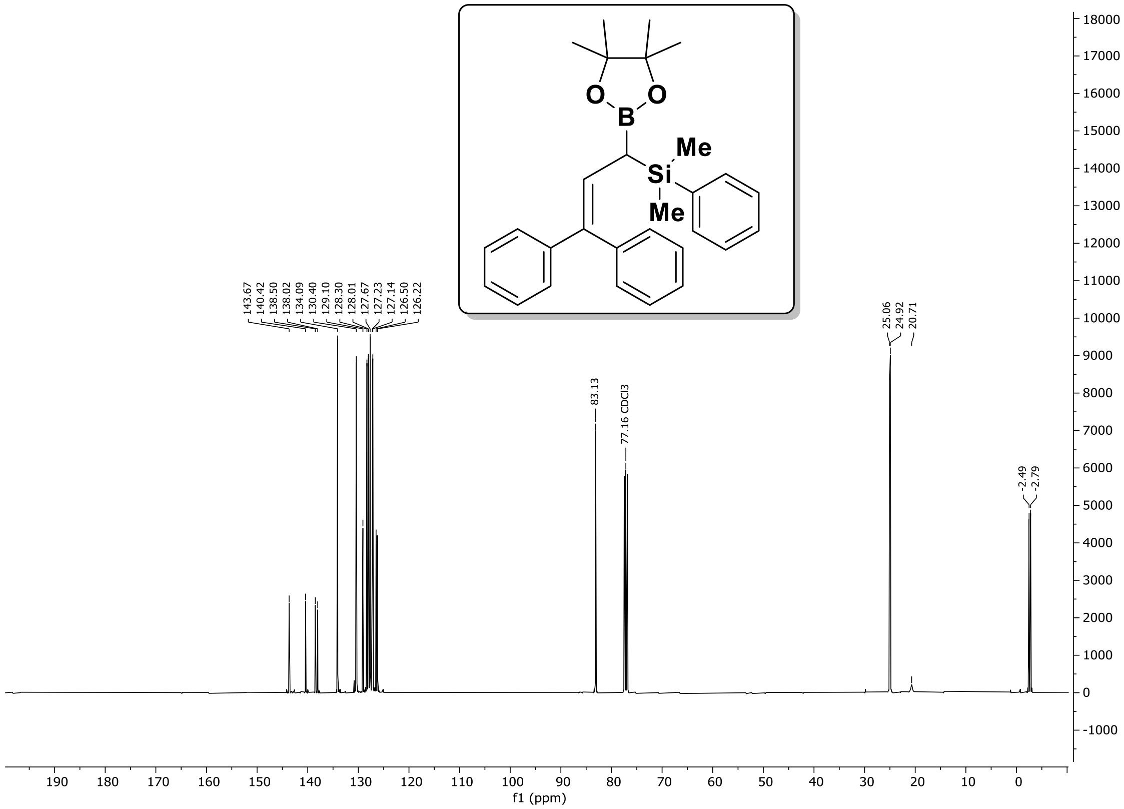

^{1}H-NMR of 6 (400 MHz, $CDCl_3$)

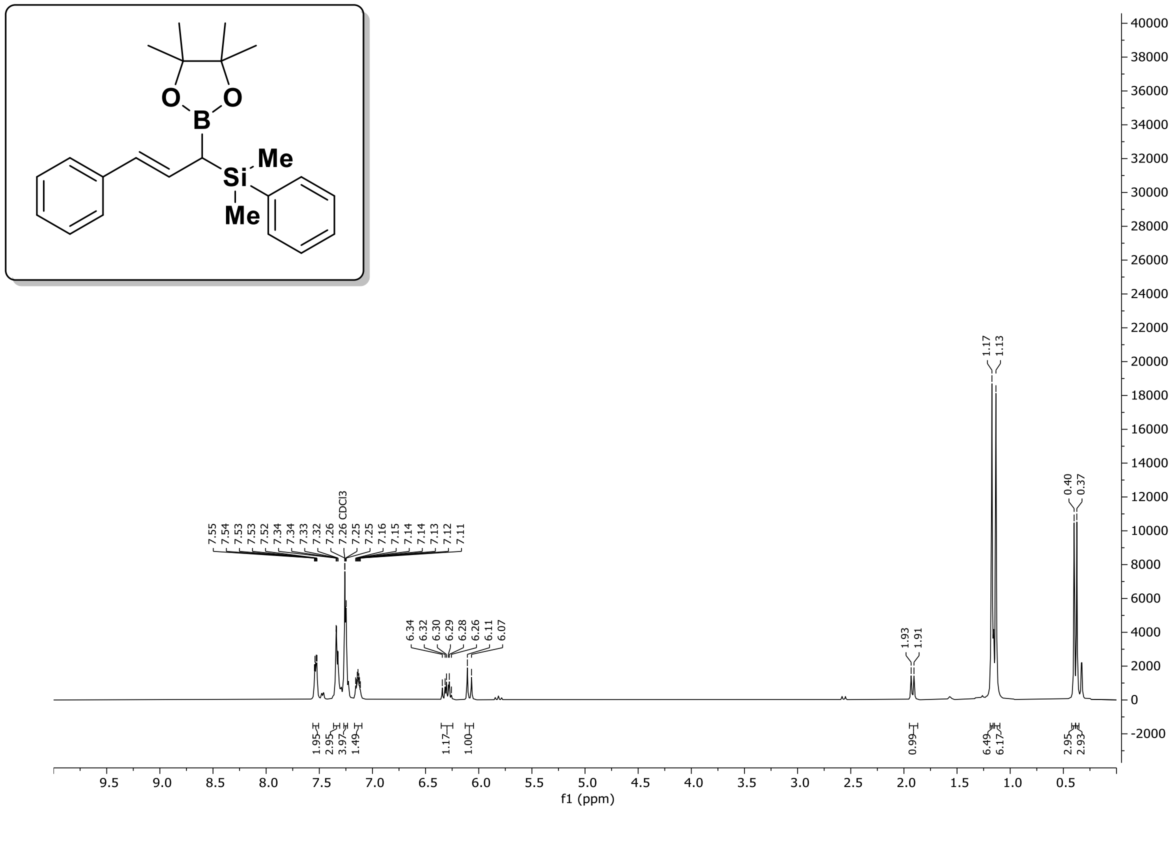

^{1}H-NMR of 7 (400 MHz, $CDCl_3$)

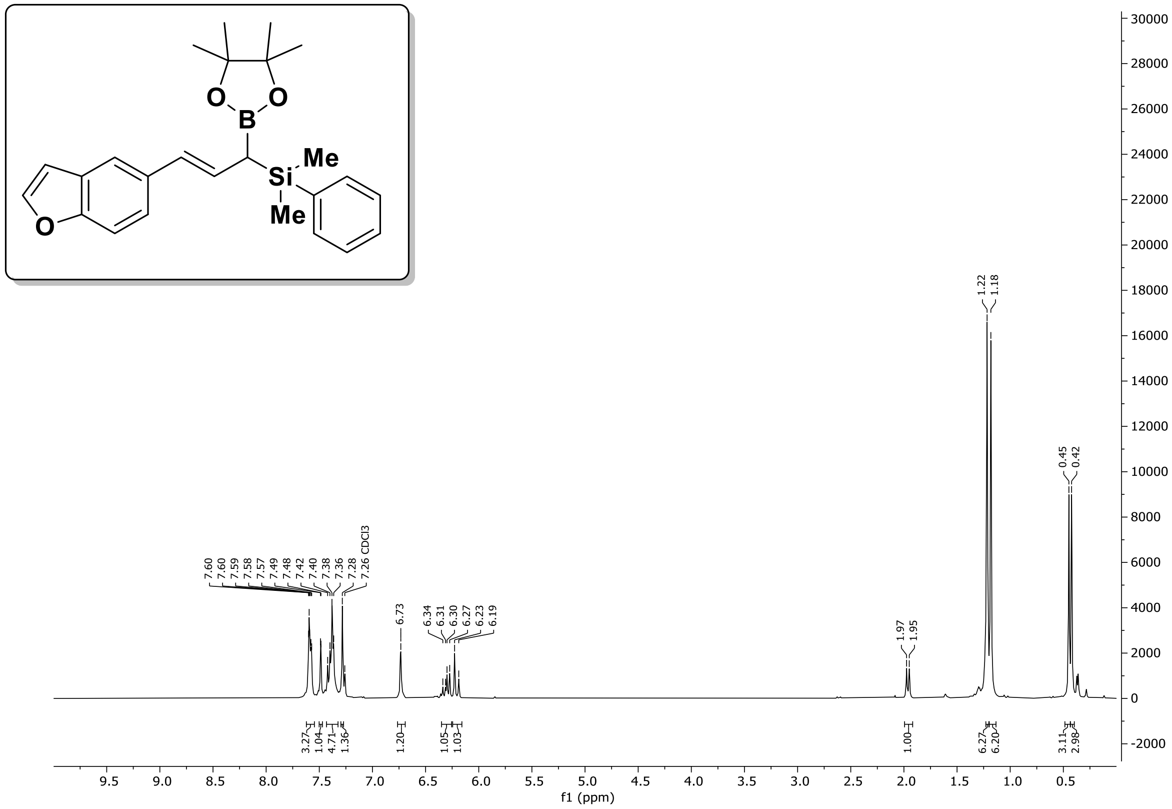

^{13}C-NMR of 7 (101 MHz, $CDCl_3$)

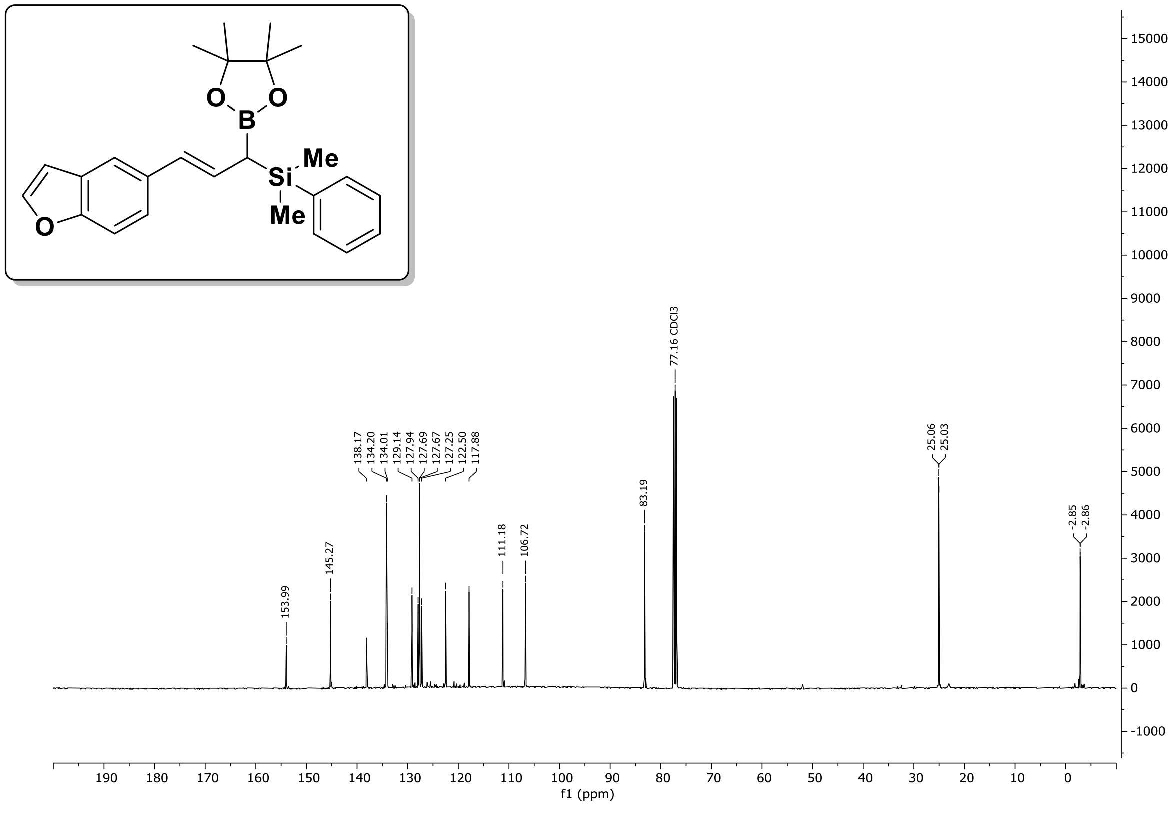

^{1}H-NMR of 8 (400 MHz, $CDCl_3$)

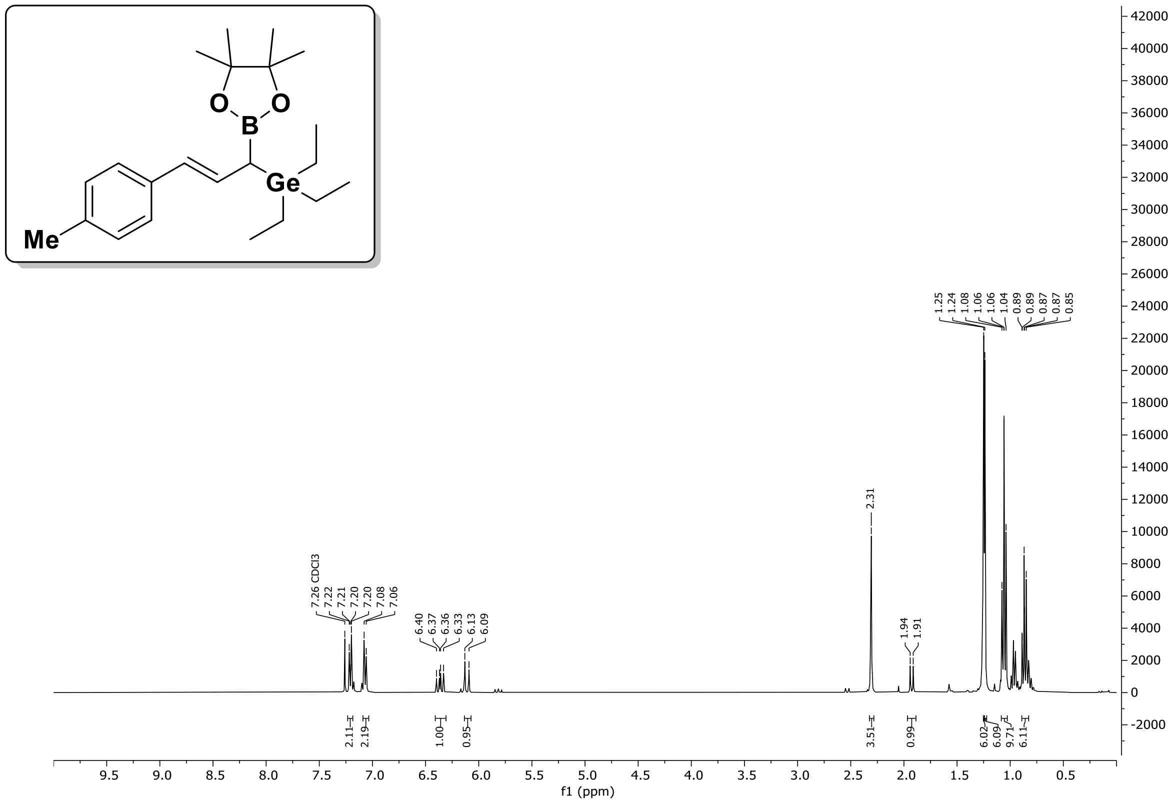

^{13}C-NMR of 8 (101 MHz, $CDCl_3$)

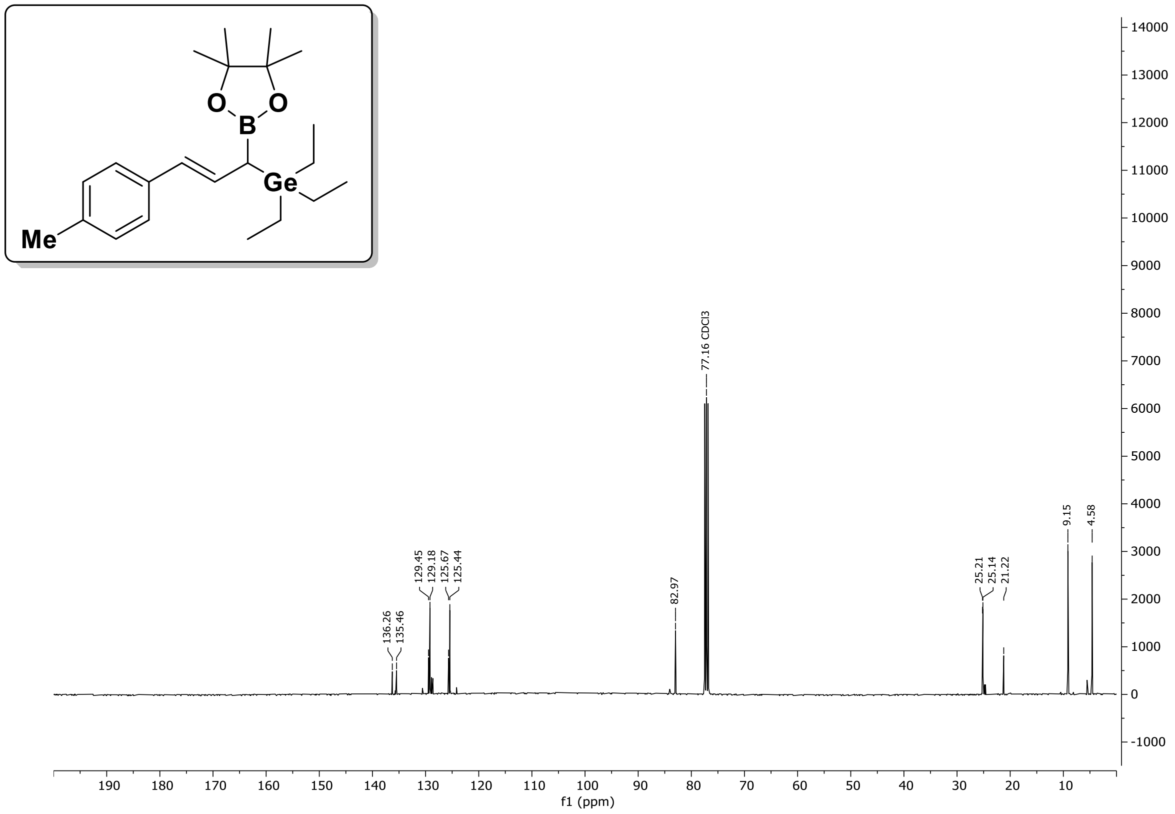

^{1}H-NMR of 9 (400 MHz, $CDCl_3$)

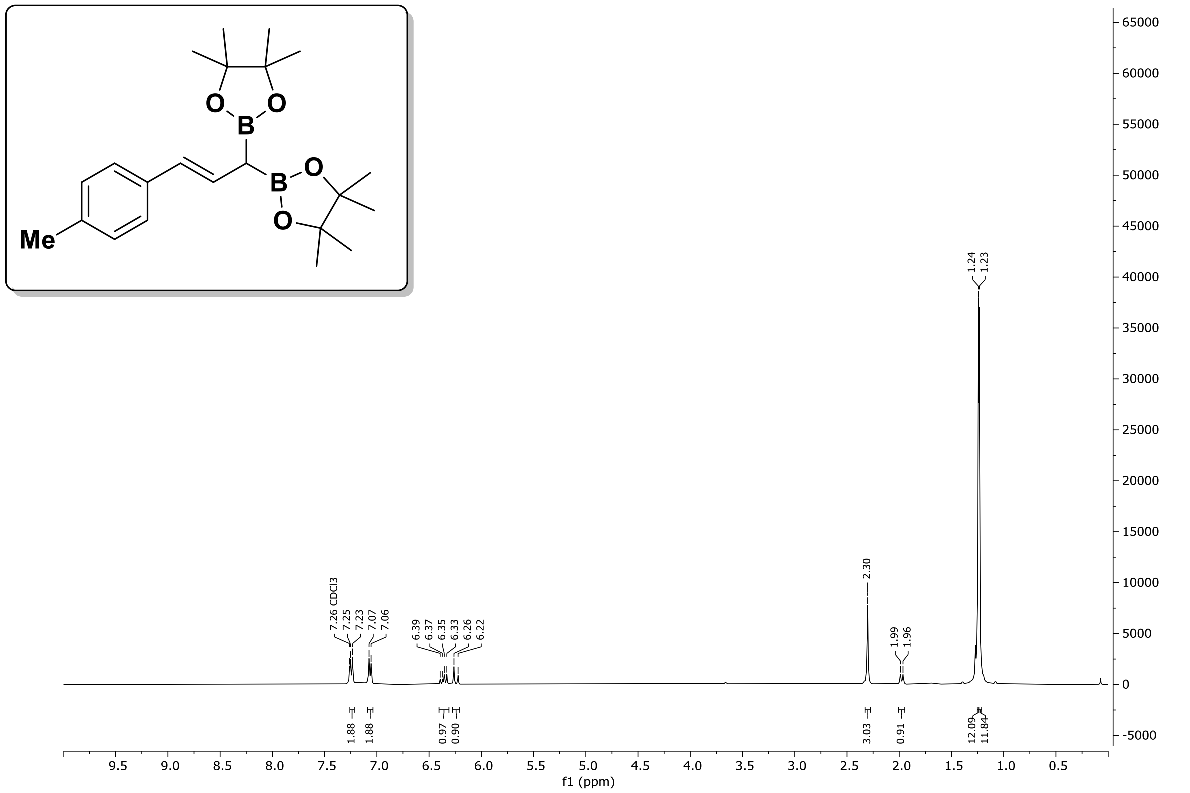

^{1}H-NMR of 10 (400 MHz, $CDCl_3$)

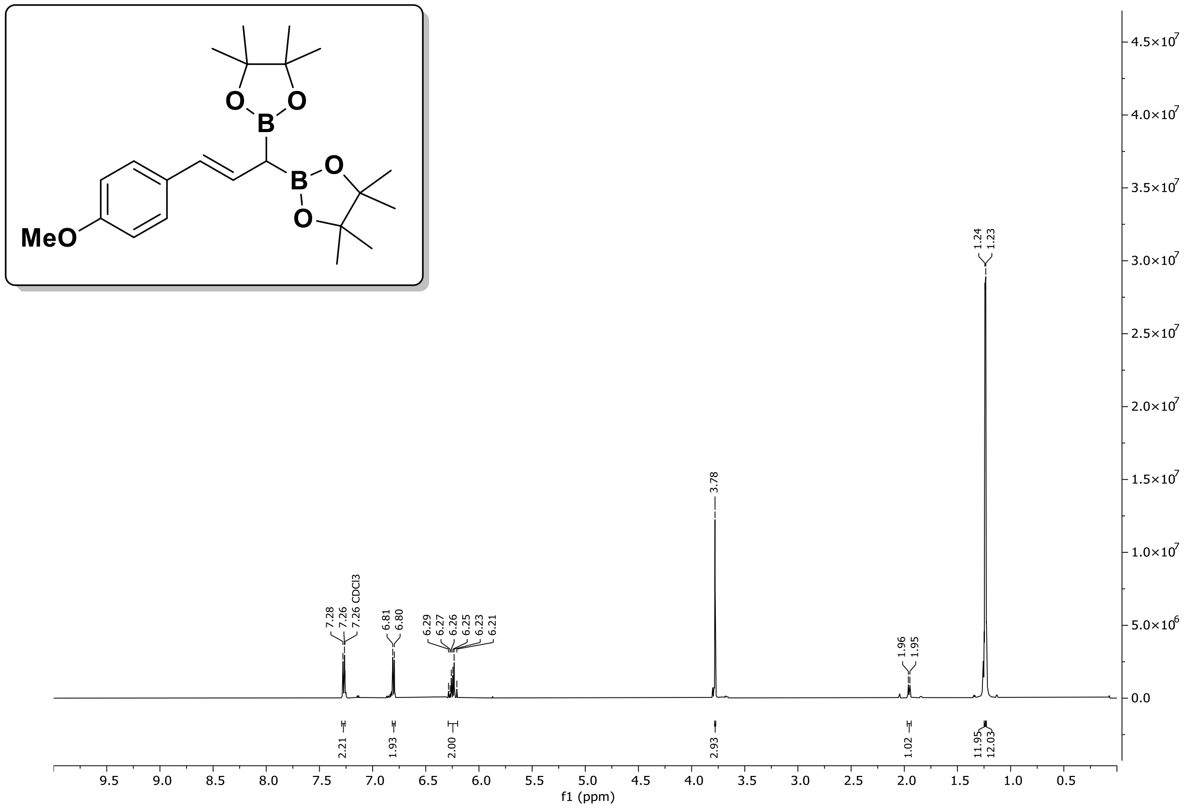

^{13}C-NMR of 10 (101 MHz, $CDCl_3$)

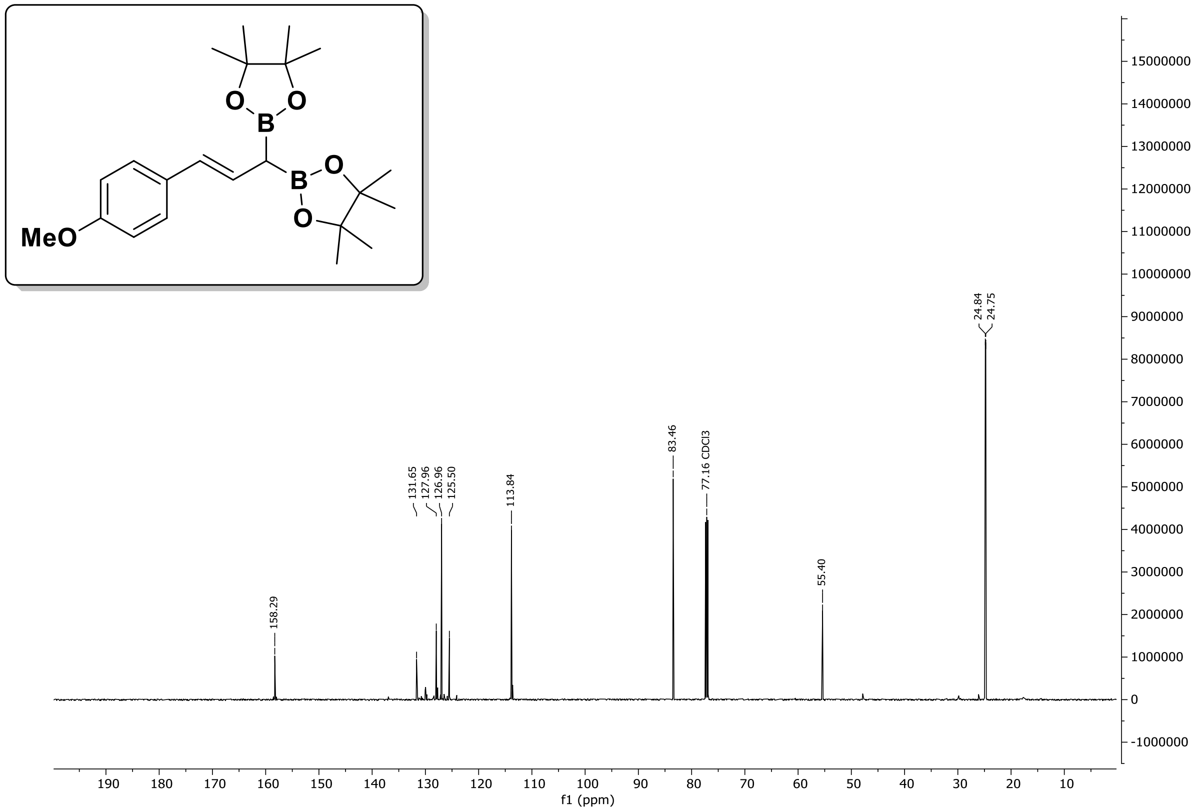

^{1}H-NMR of 11 (400 MHz, $CDCl_3$)

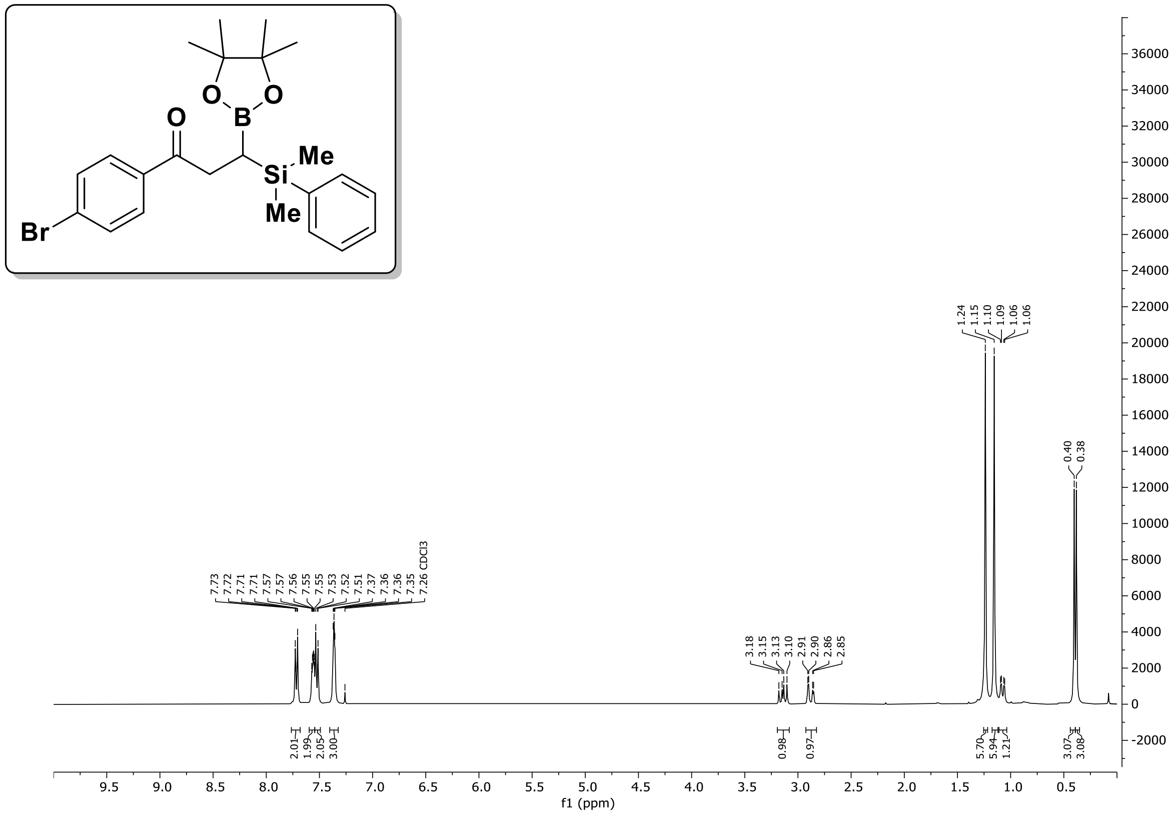

^{13}C-NMR of 11 (101 MHz, $CDCl_3$)

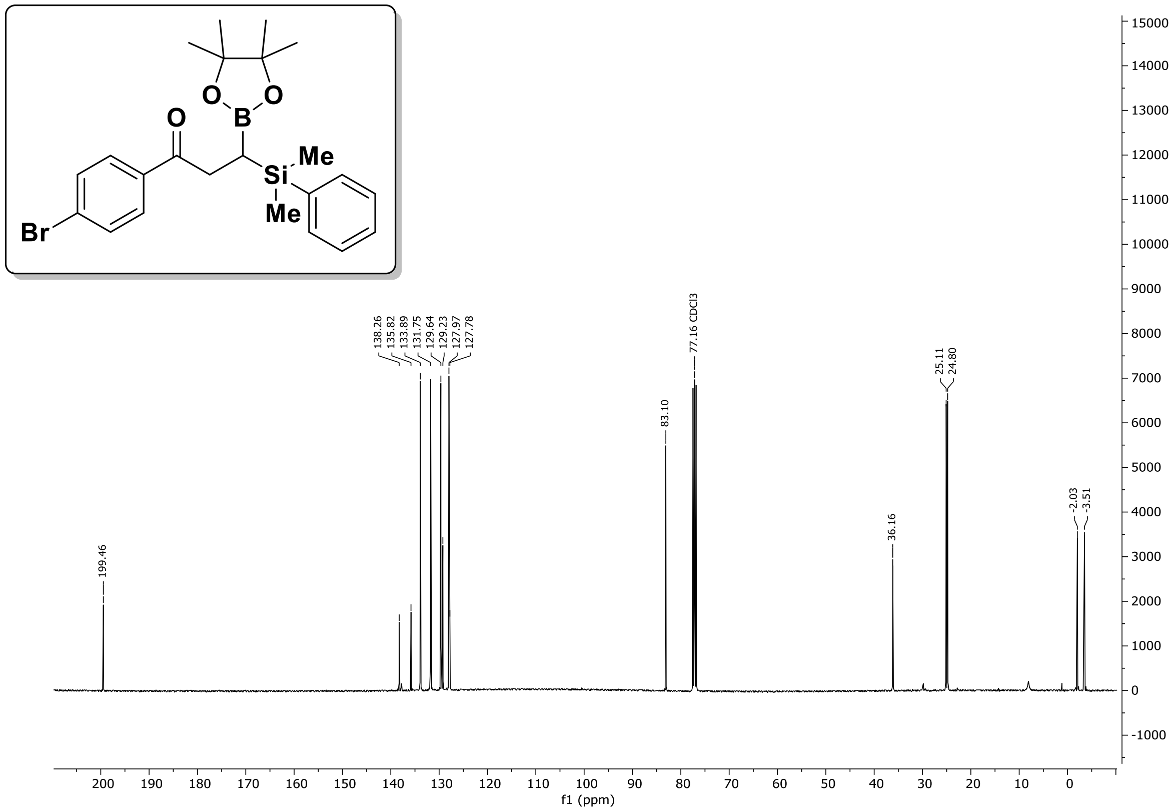

^{1}H-NMR of 12 (400 MHz, $CDCl_3$)

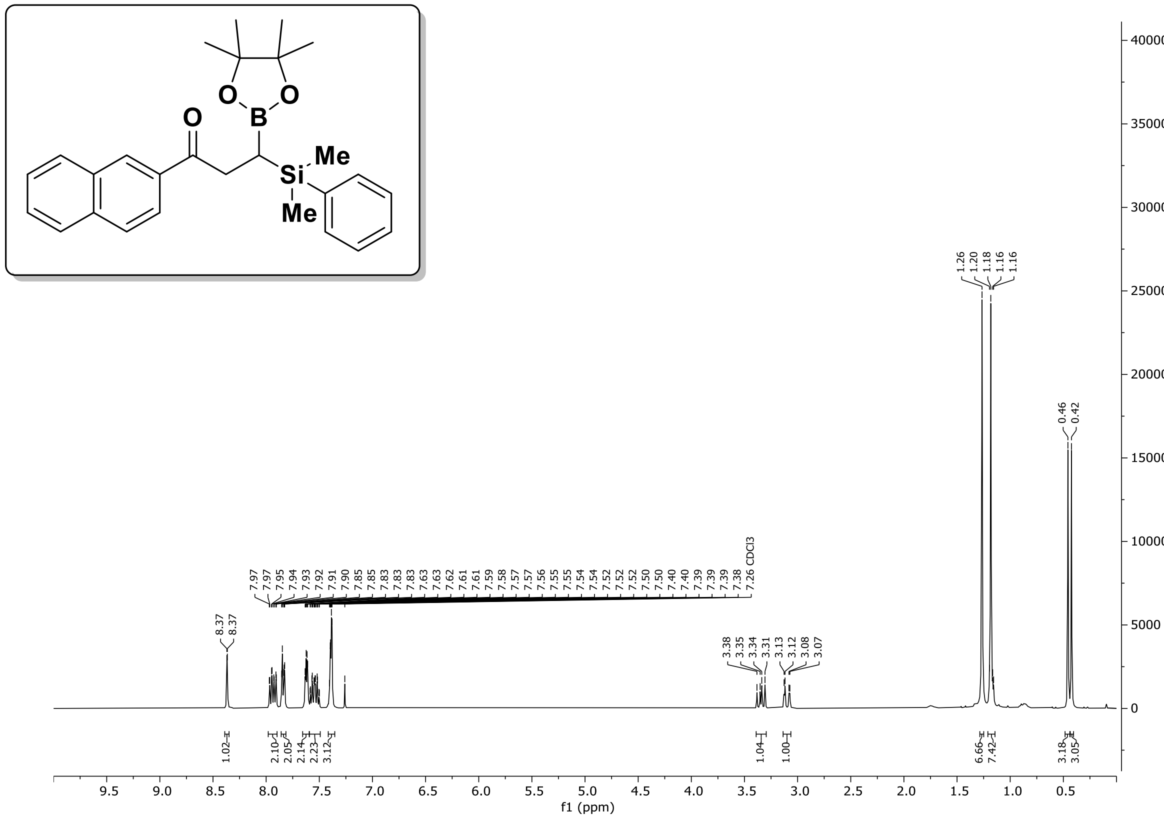

^{13}C-NMR of 12 (101 MHz, $CDCl_3$)

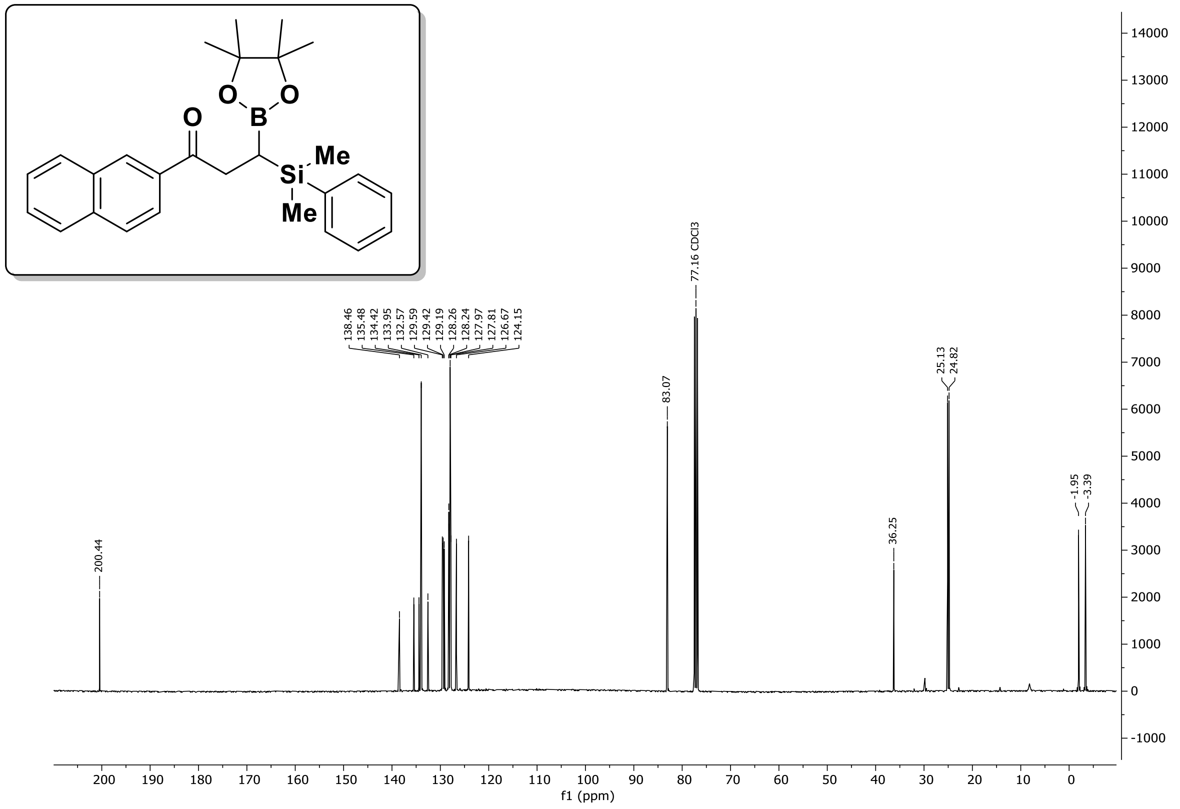

^{1}H-NMR of 13 (400 MHz, $CDCl_3$)

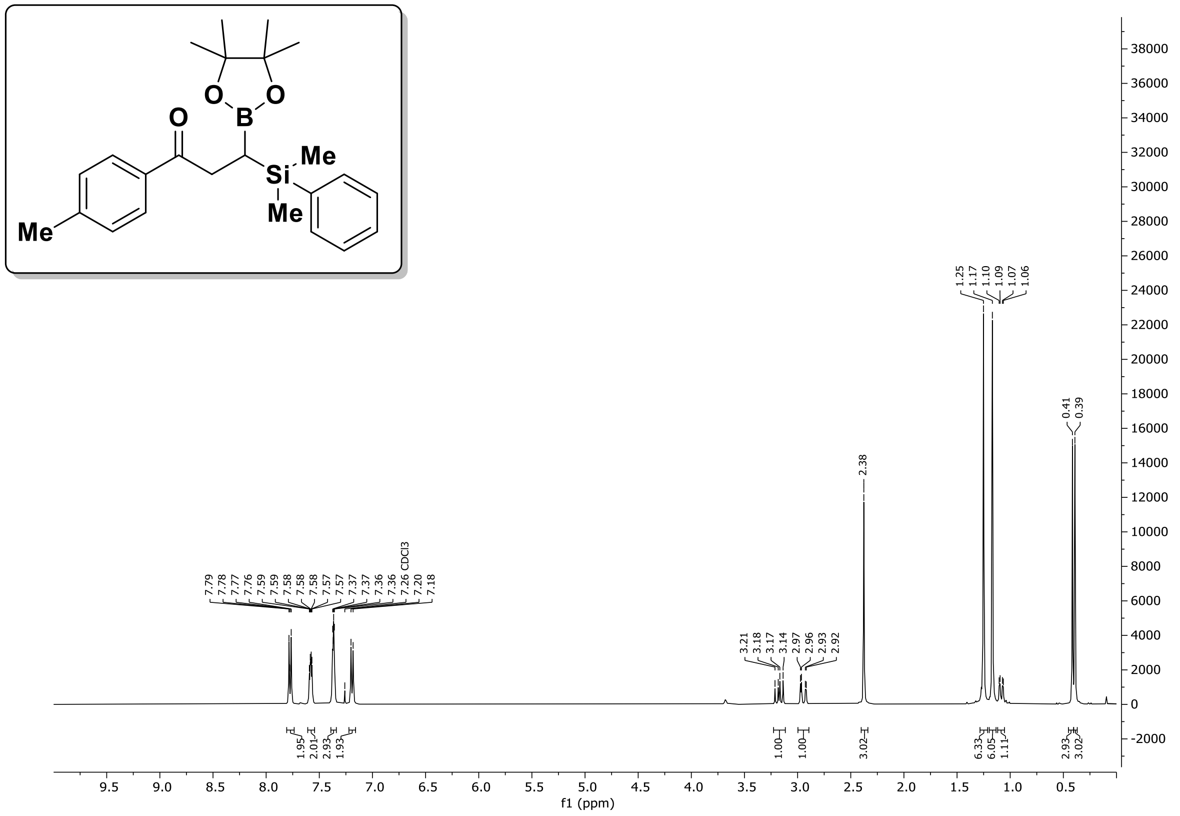

^{13}C-NMR of 13 (101 MHz, $CDCl_3$)

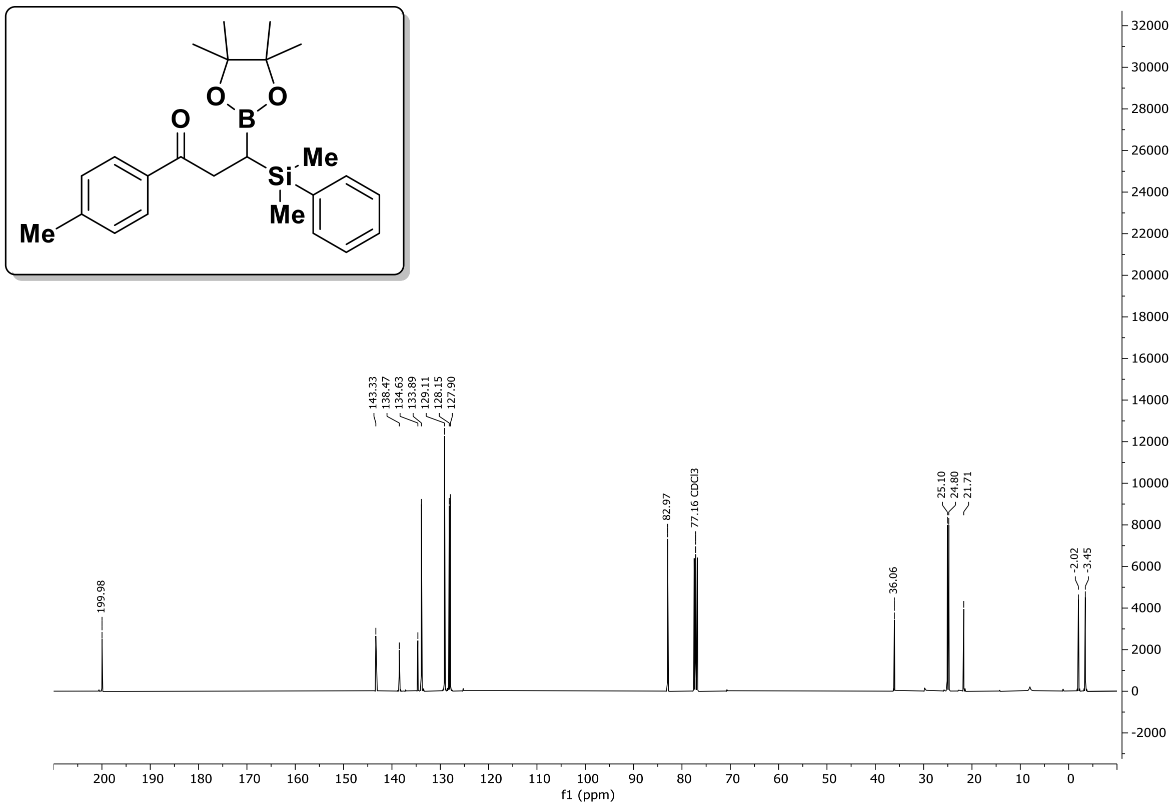

^{1}H-NMR of 14 (400 MHz, $CDCl_3$)

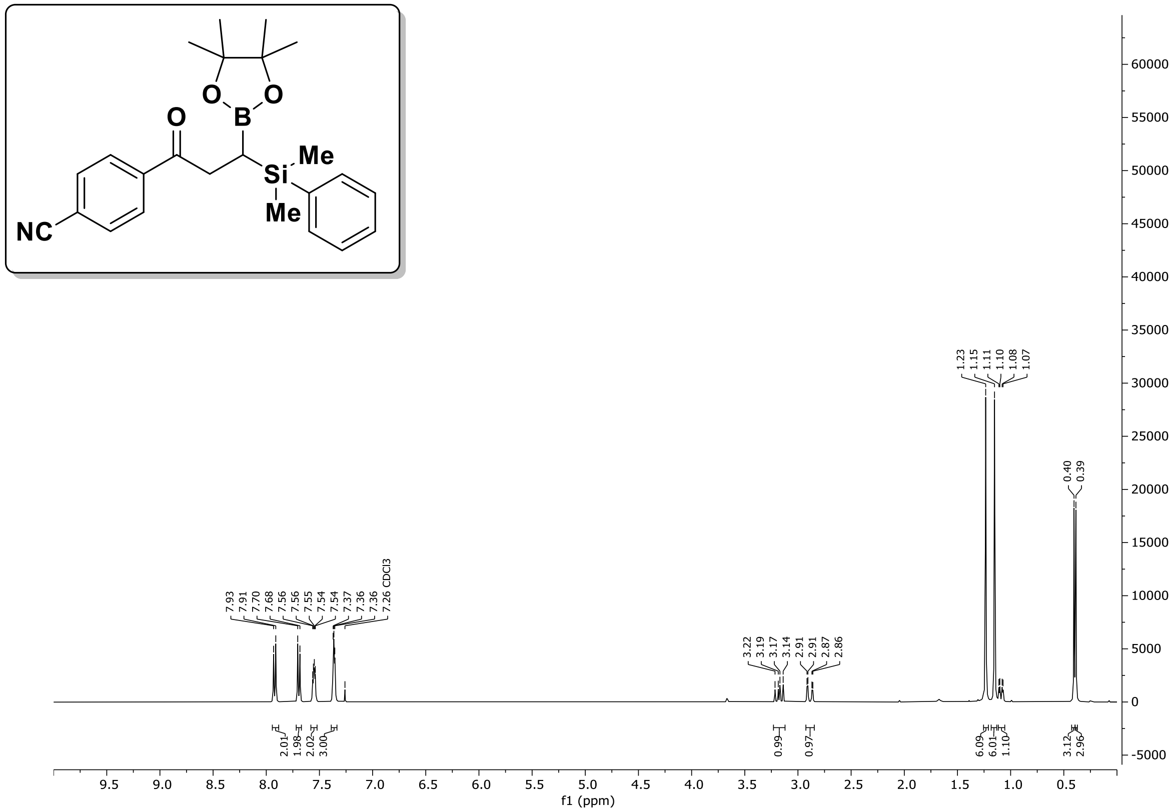

^{13}C-NMR of 14 (101 MHz, $CDCl_3$)

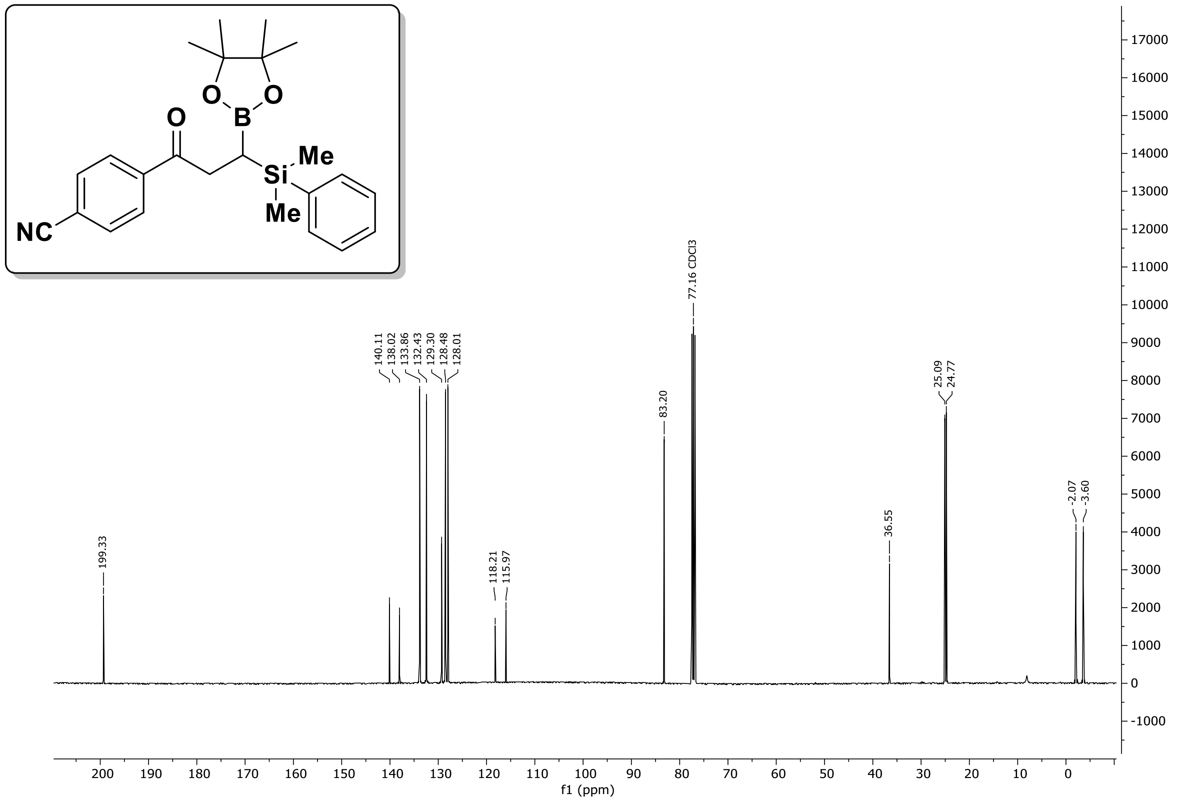

^{1}H-NMR of 15 (400 MHz, $CDCl_3$)

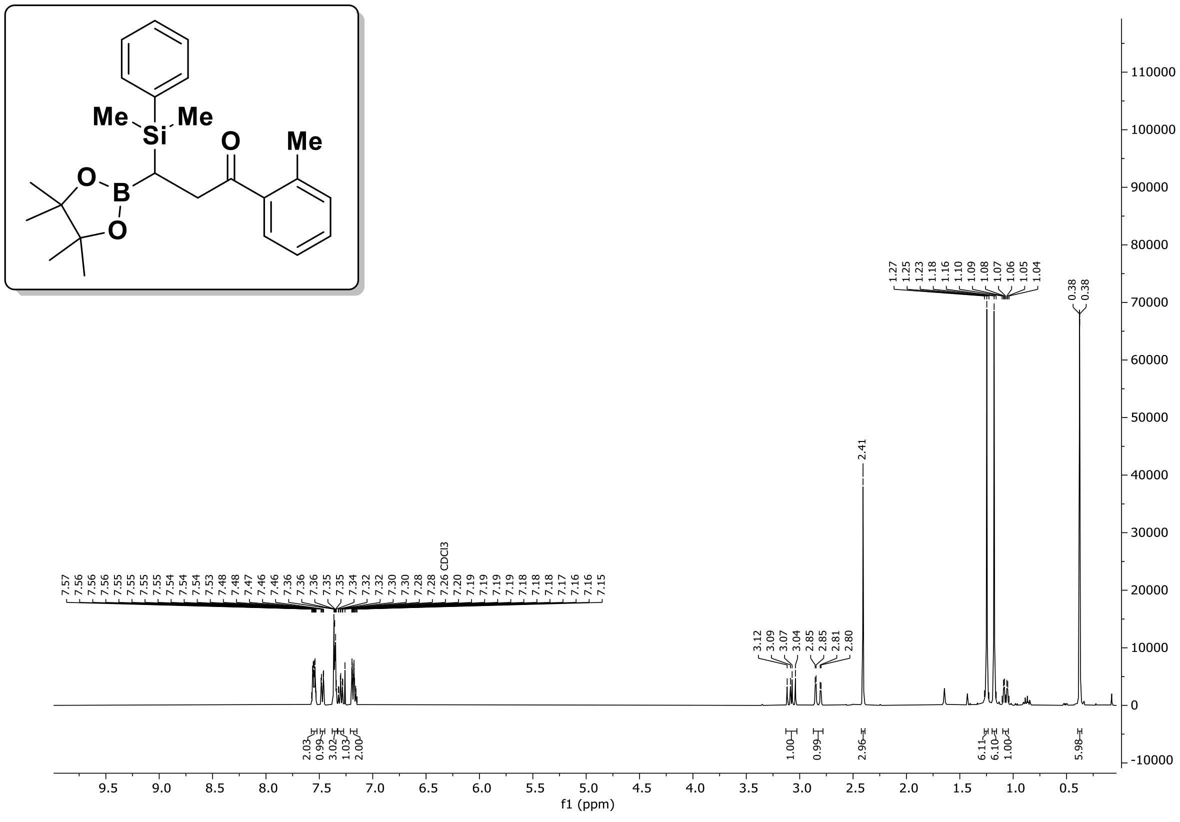

^{13}C NMR of 15 (101 MHz, $CDCl_3$)

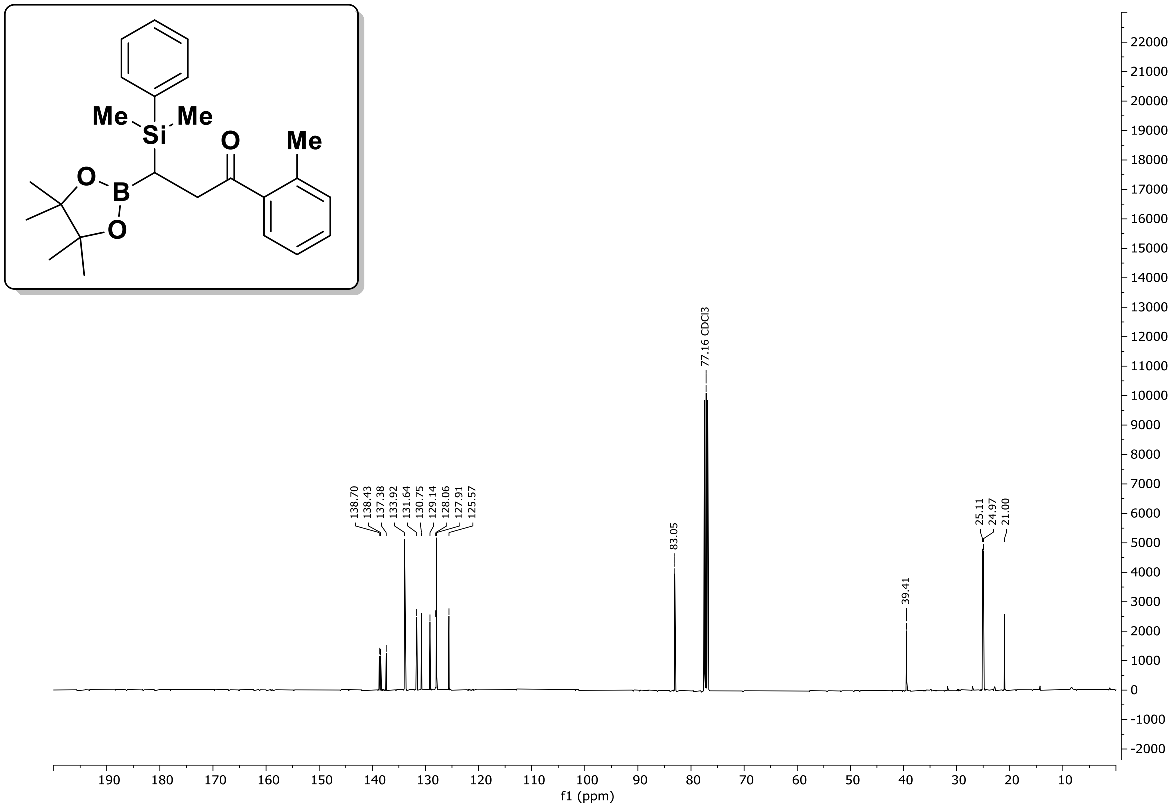

^{1}H-NMR of 16 (400 MHz, $CDCl_3$)

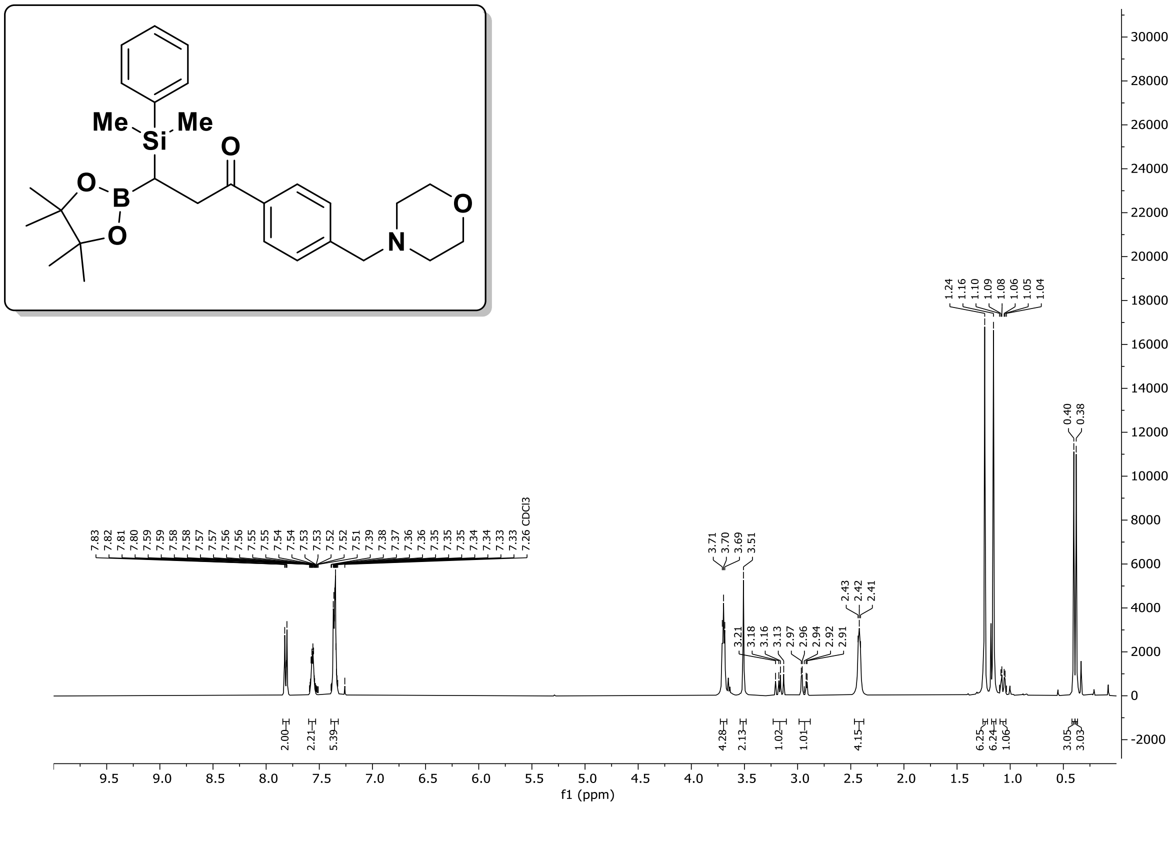

^{13}C NMR of 16 (101 MHz, $CDCl_3$)

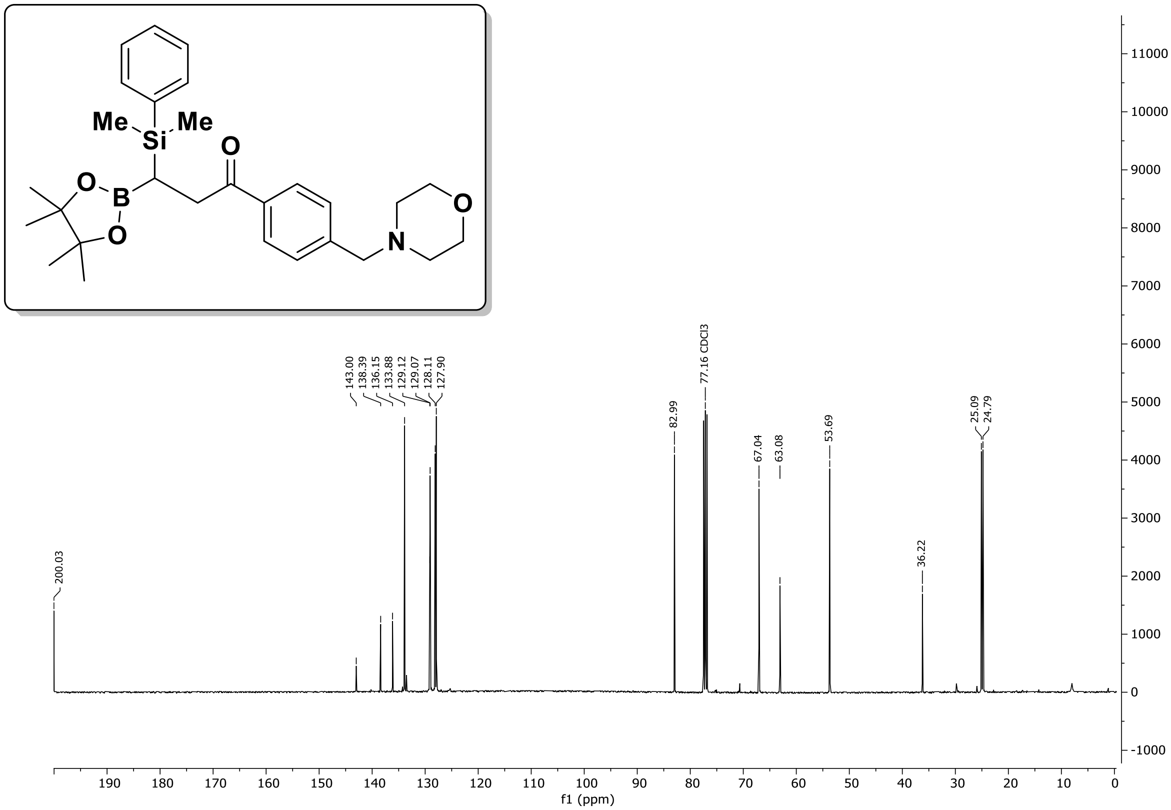

^{1}H-NMR of 17 (400 MHz, $CDCl_3$)

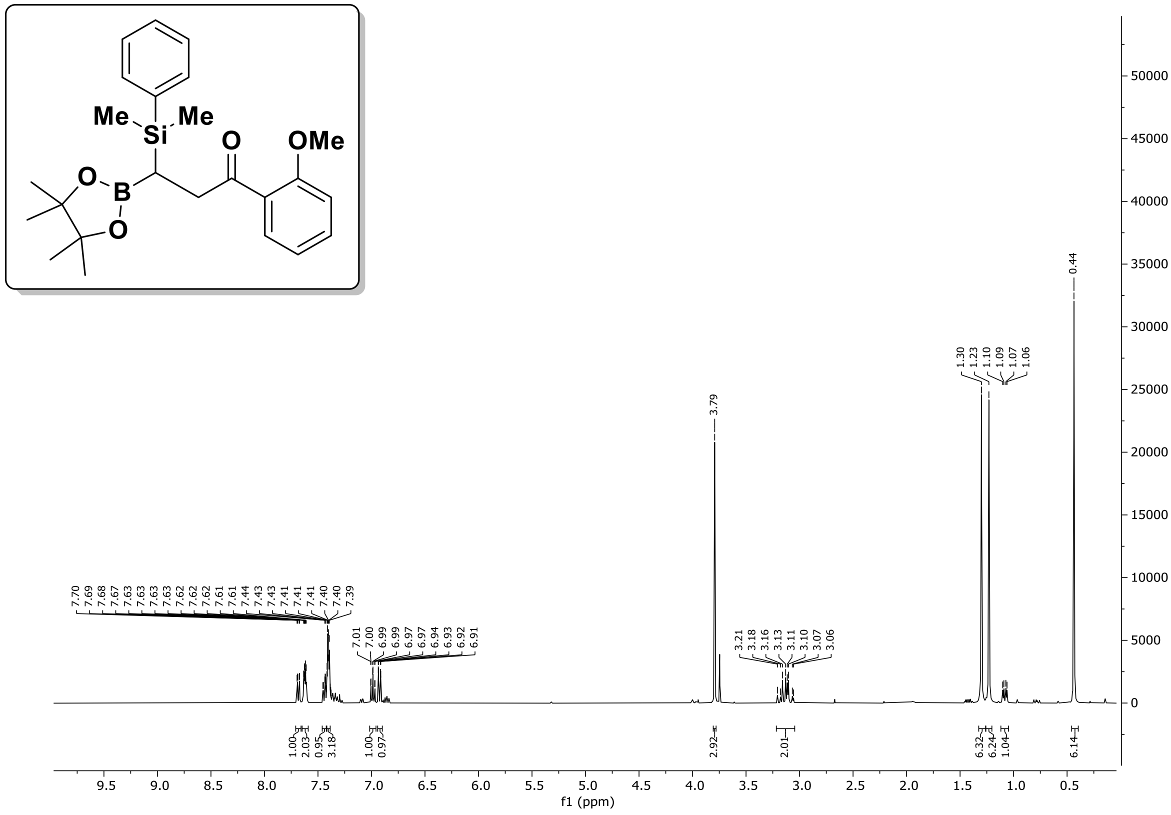

^{13}C NMR of 17 (101 MHz, $CDCl_3$)

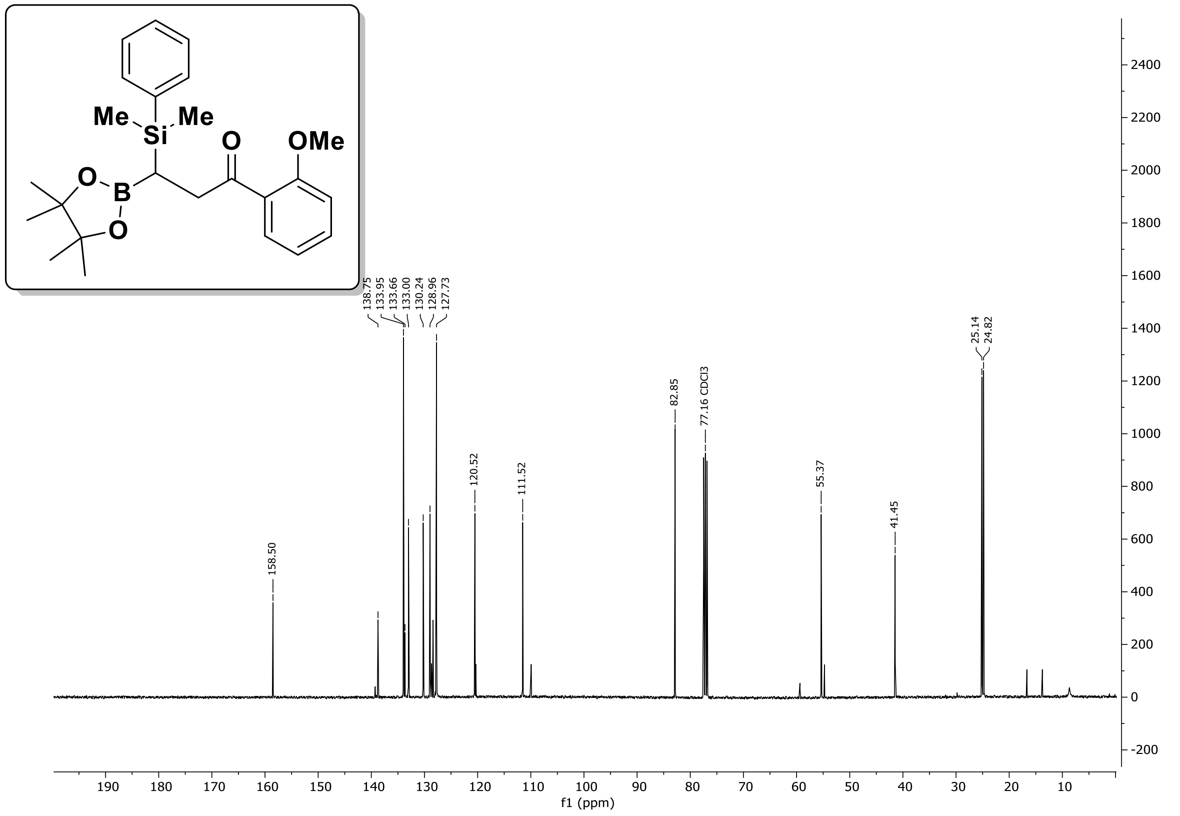

^{1}H-NMR of 18 (400 MHz, $CDCl_3$)

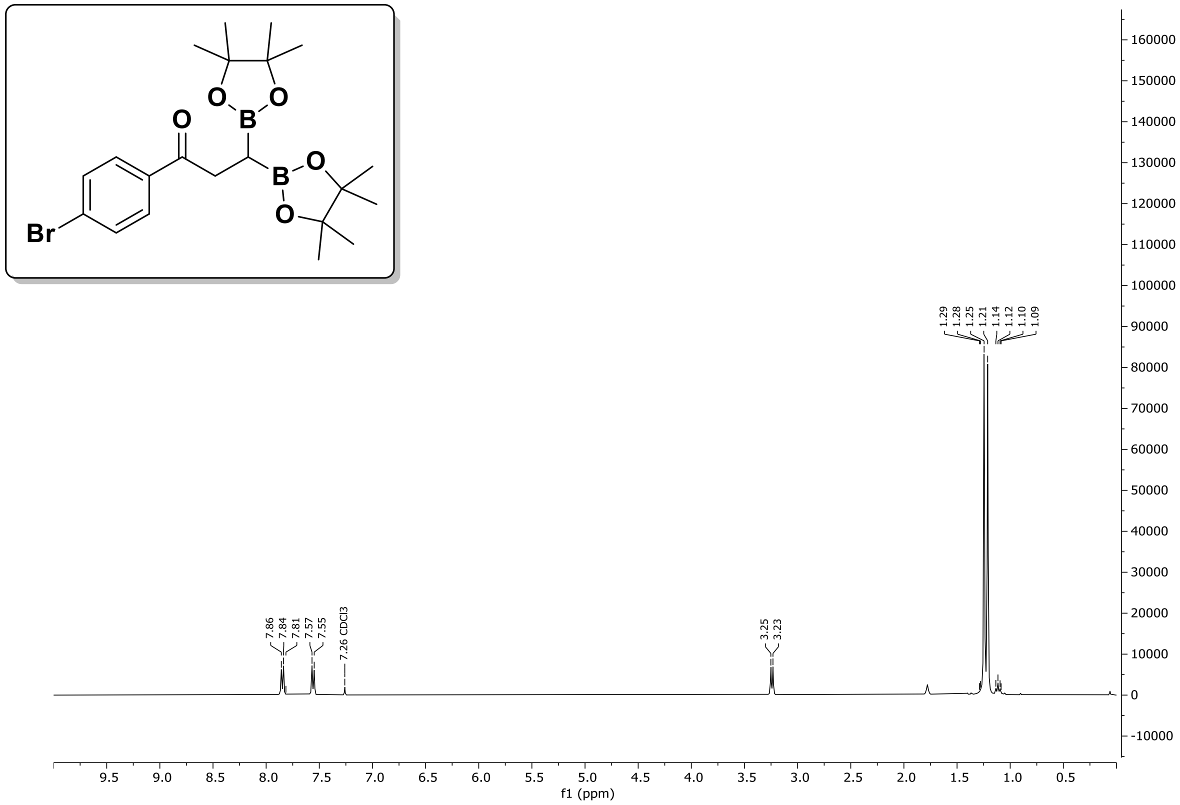

^{1}H-NMR of 19 (400 MHz, $CDCl_3$)

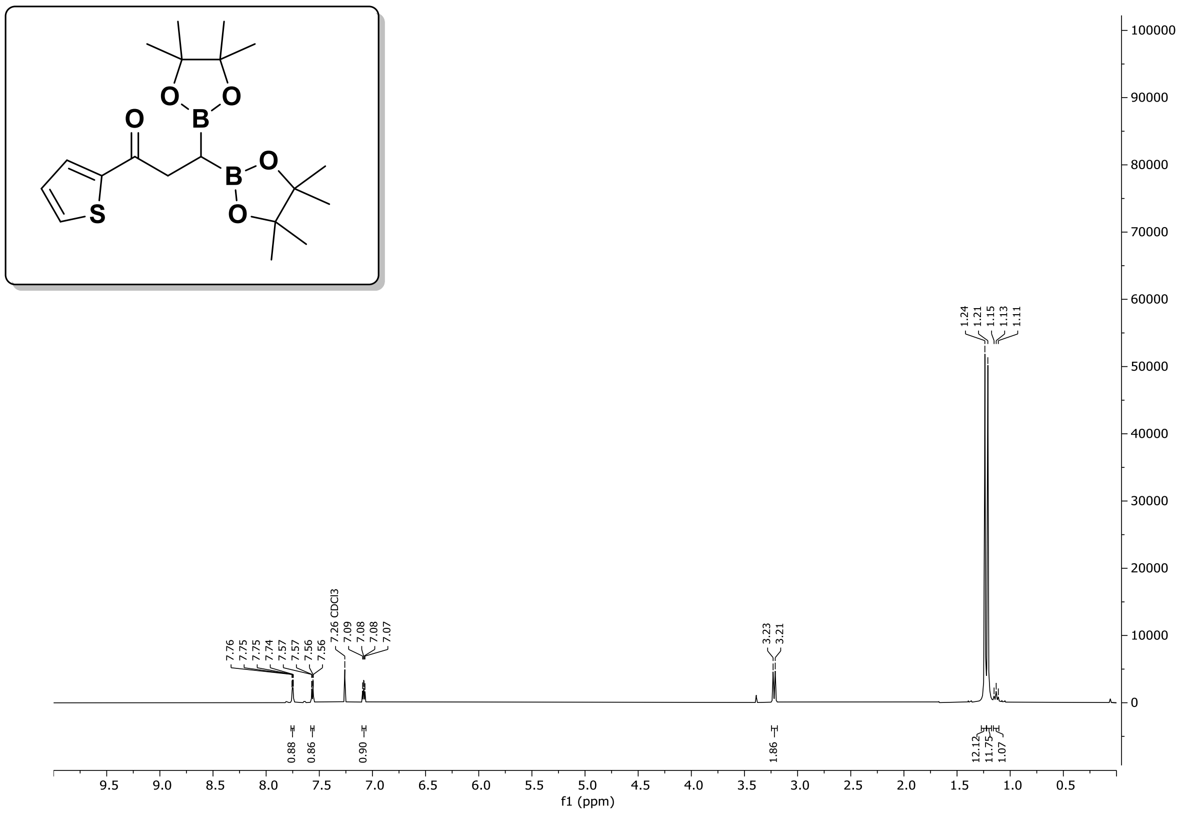

^{1}H-NMR of 20 (600 MHz, $CDCl_3$)

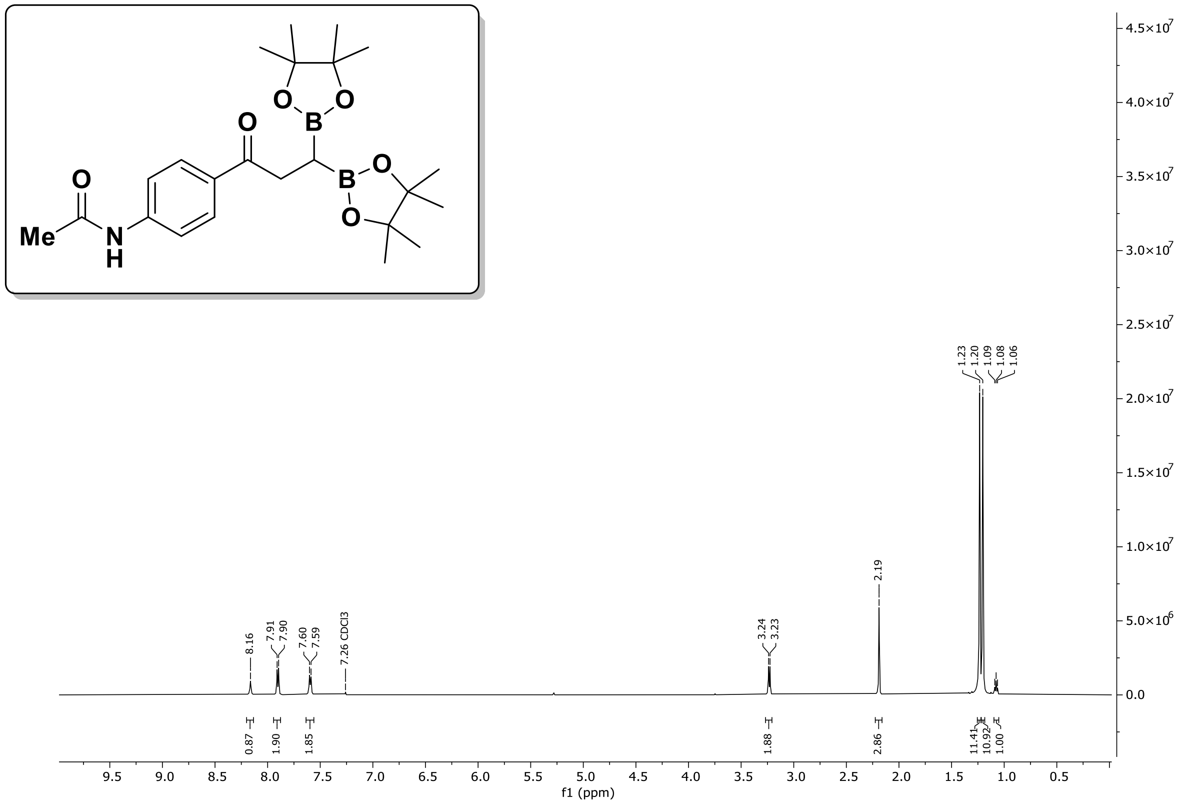

^{13}C NMR of 20 (150.9 MHz, $CDCl_3$)

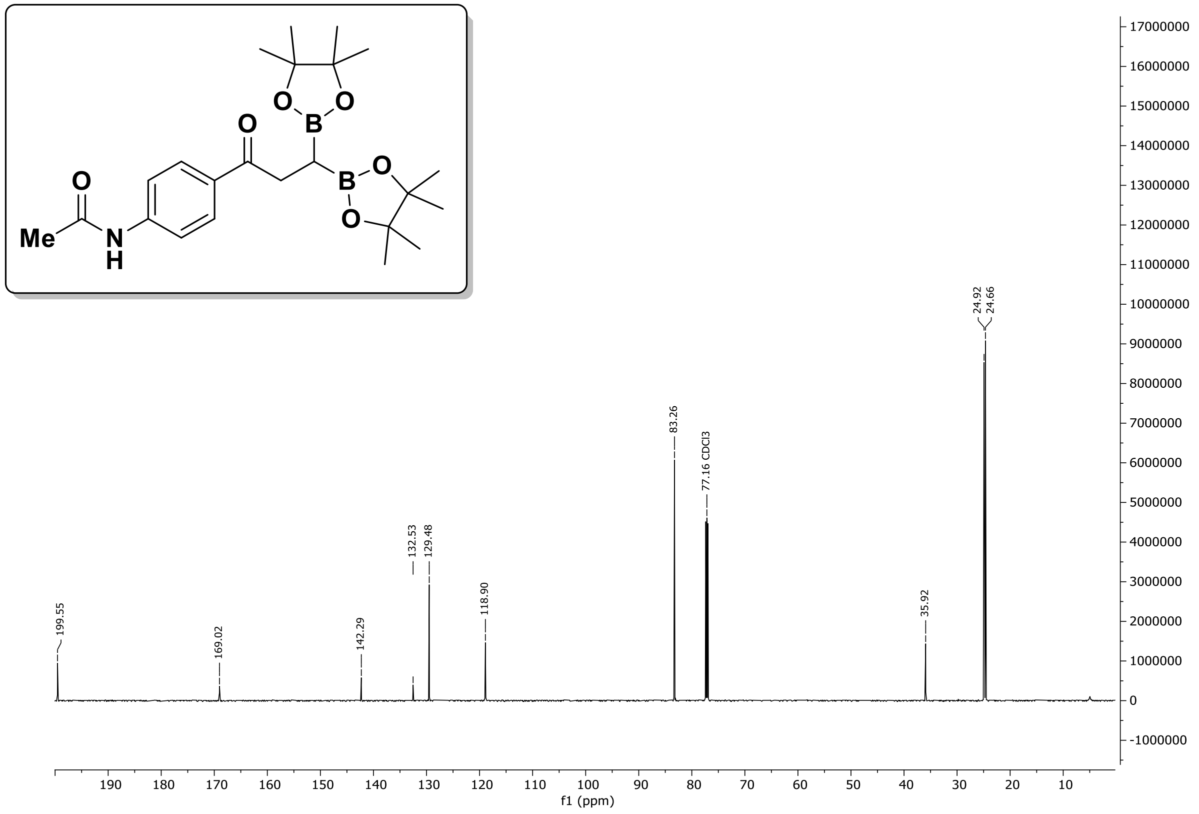

^{1}H-NMR of 21 (400 MHz, $CDCl_3$)

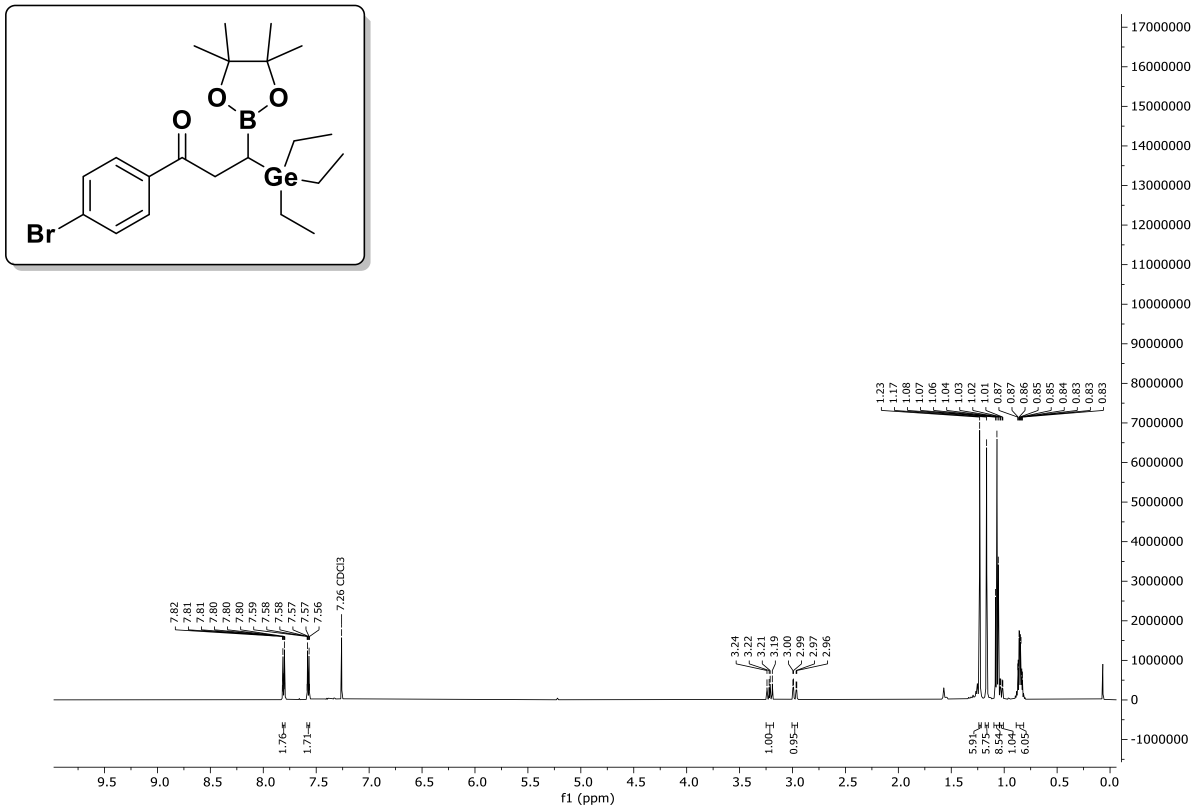

^{13}C-NMR of 21 (101 MHz, $CDCl_3$)

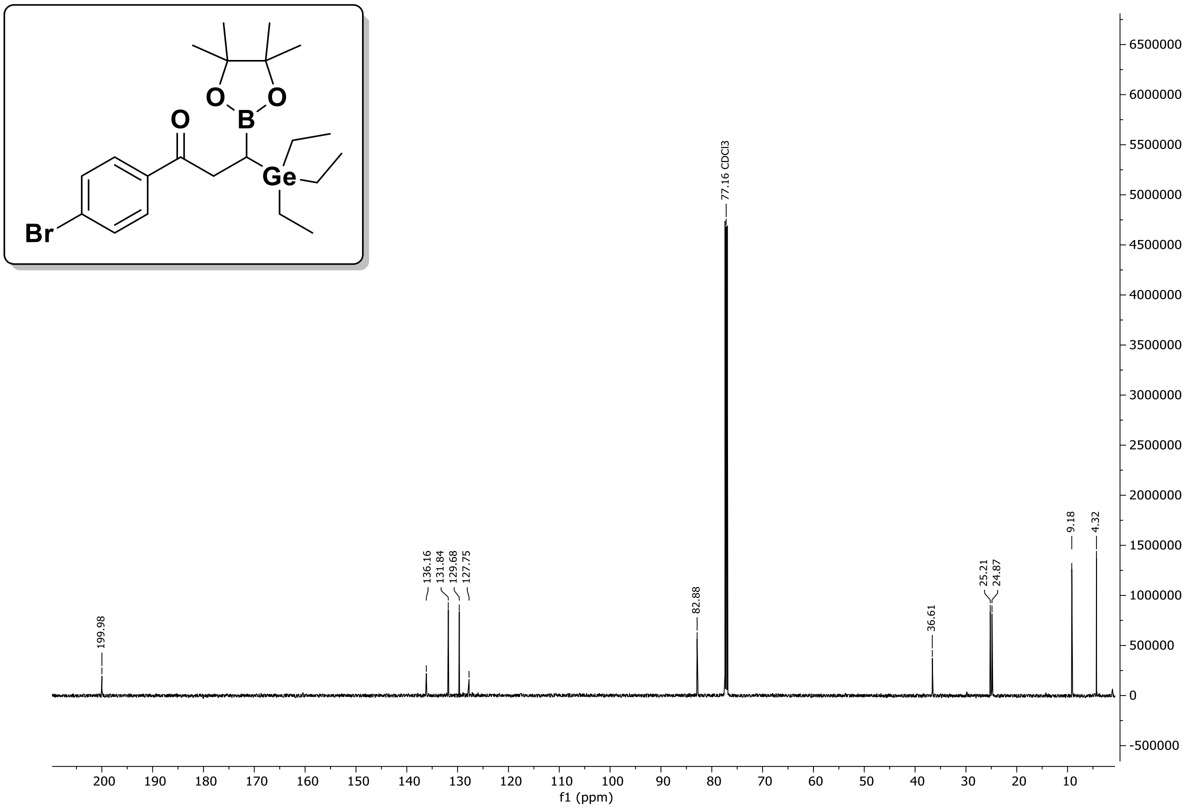

^{1}H-NMR of 22 (400 MHz, $CDCl_3$)

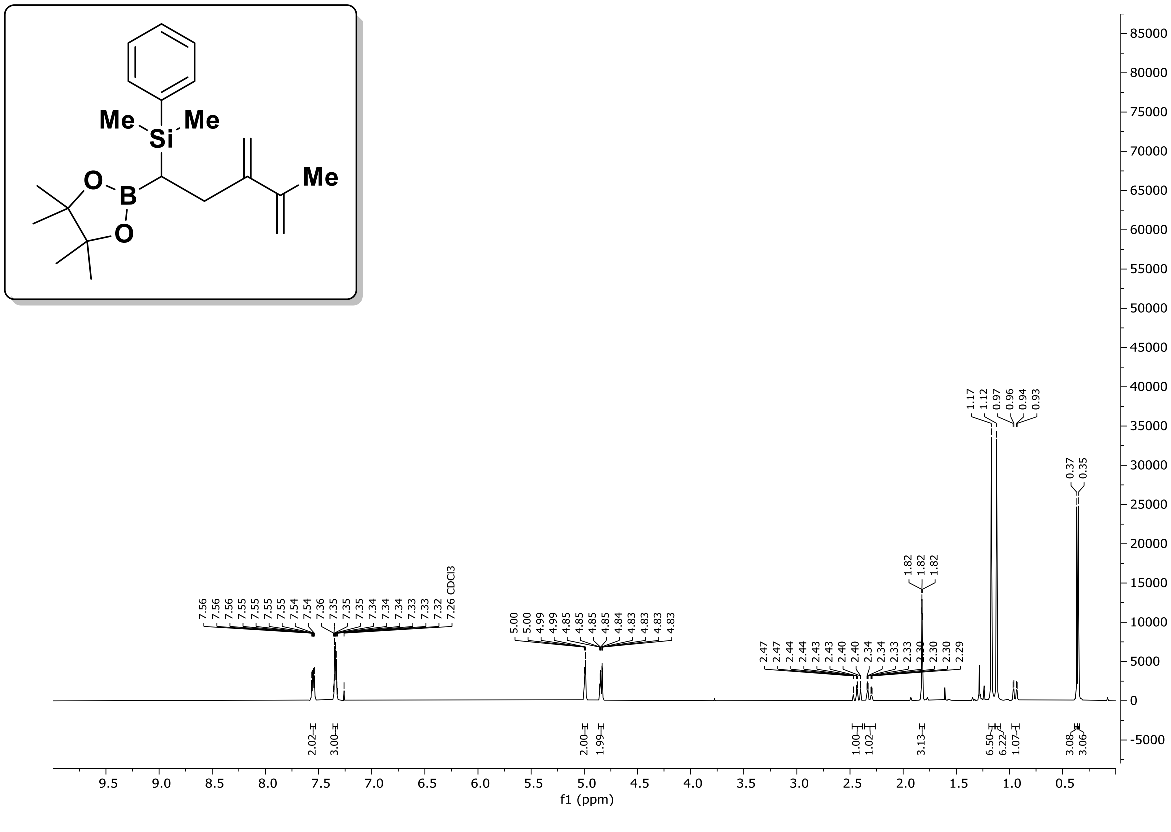

^{13}C NMR of 22 (101 MHz, $CDCl_3$)

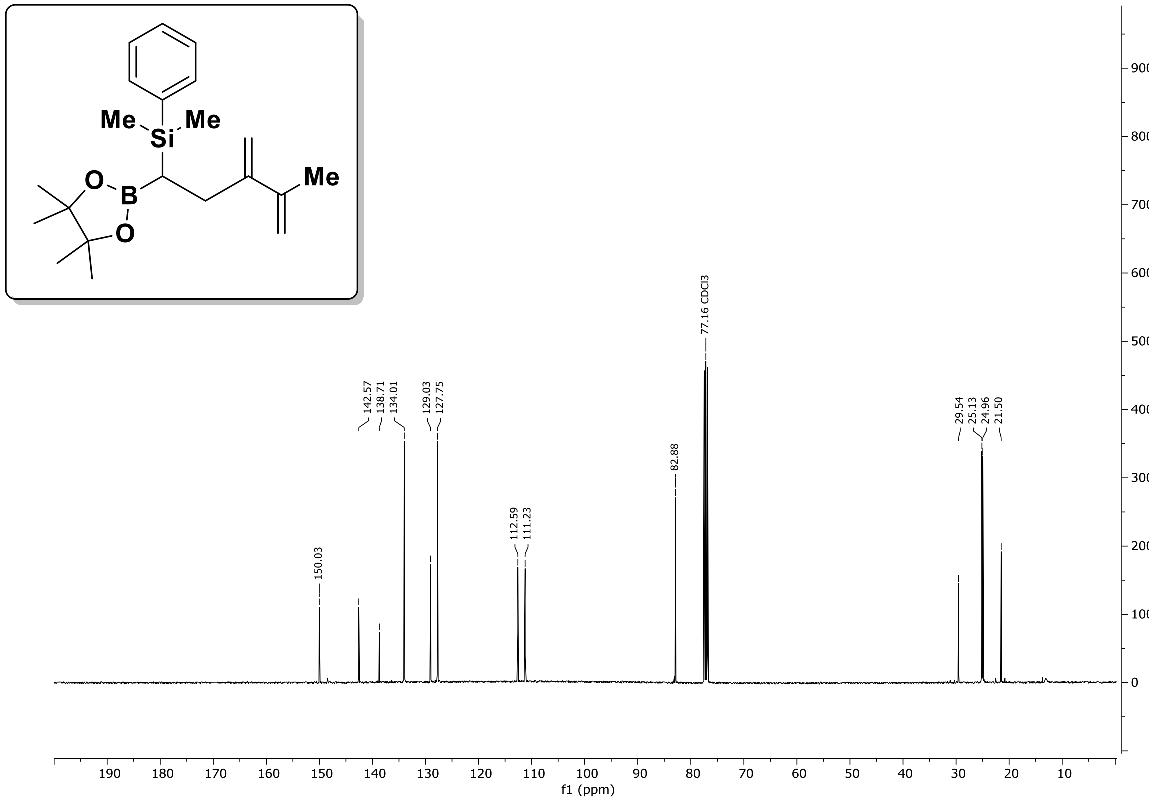

^{1}H-NMR of 23 (400 MHz, $CDCl_3$)

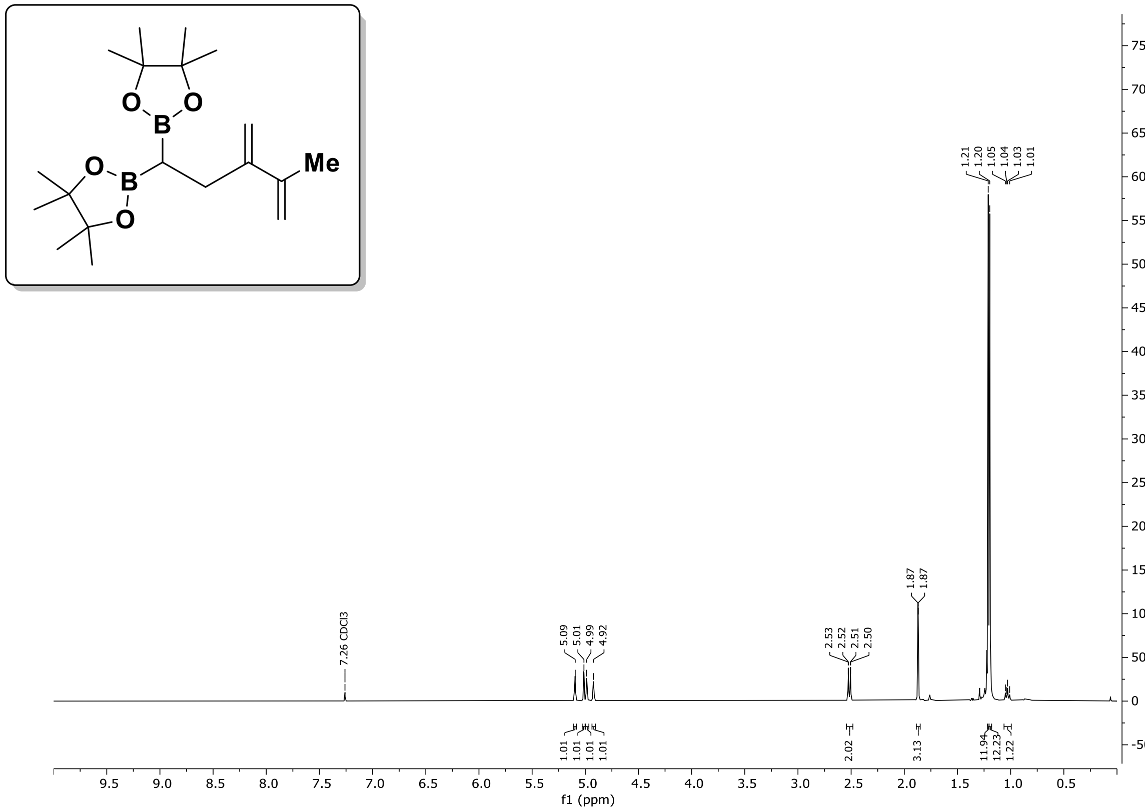

^{13}C NMR of 23 (101 MHz, $CDCl_3$)

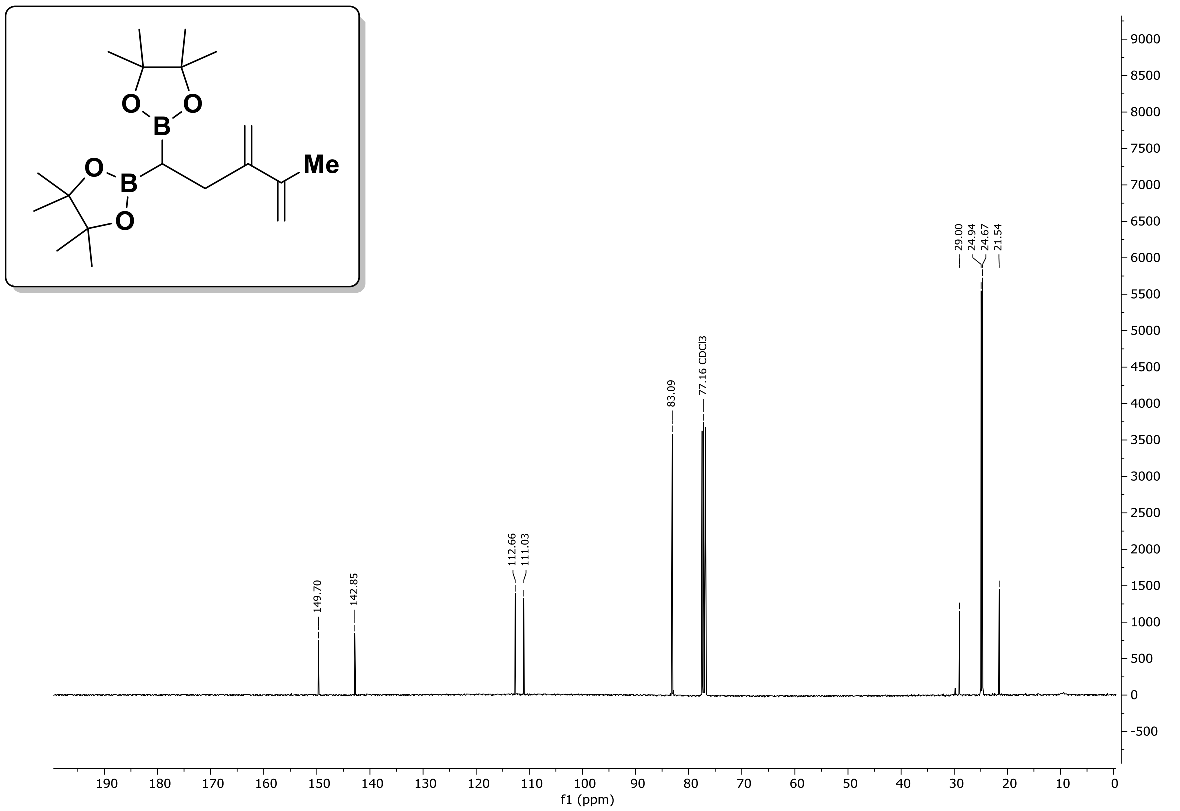

^{1}H-NMR of 24 (400 MHz, $CDCl_3$)

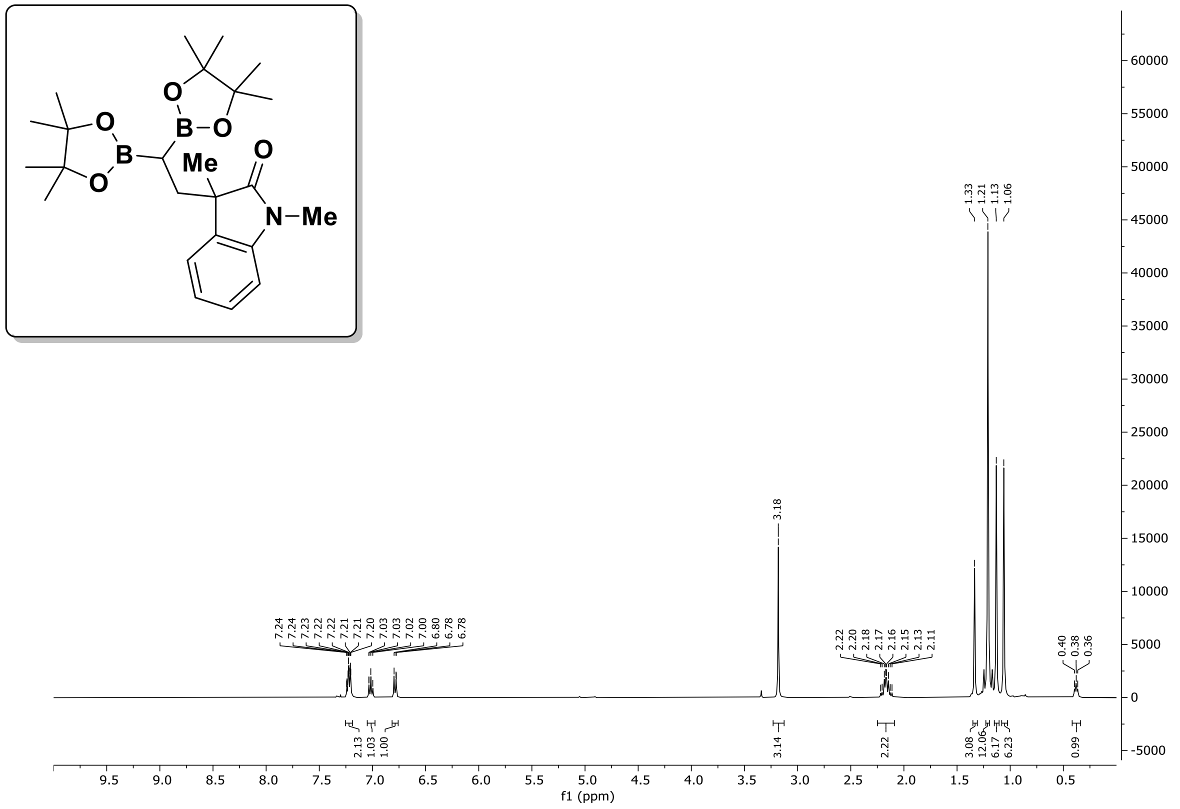

^{13}C NMR of 24 (101 MHz, $CDCl_3$)

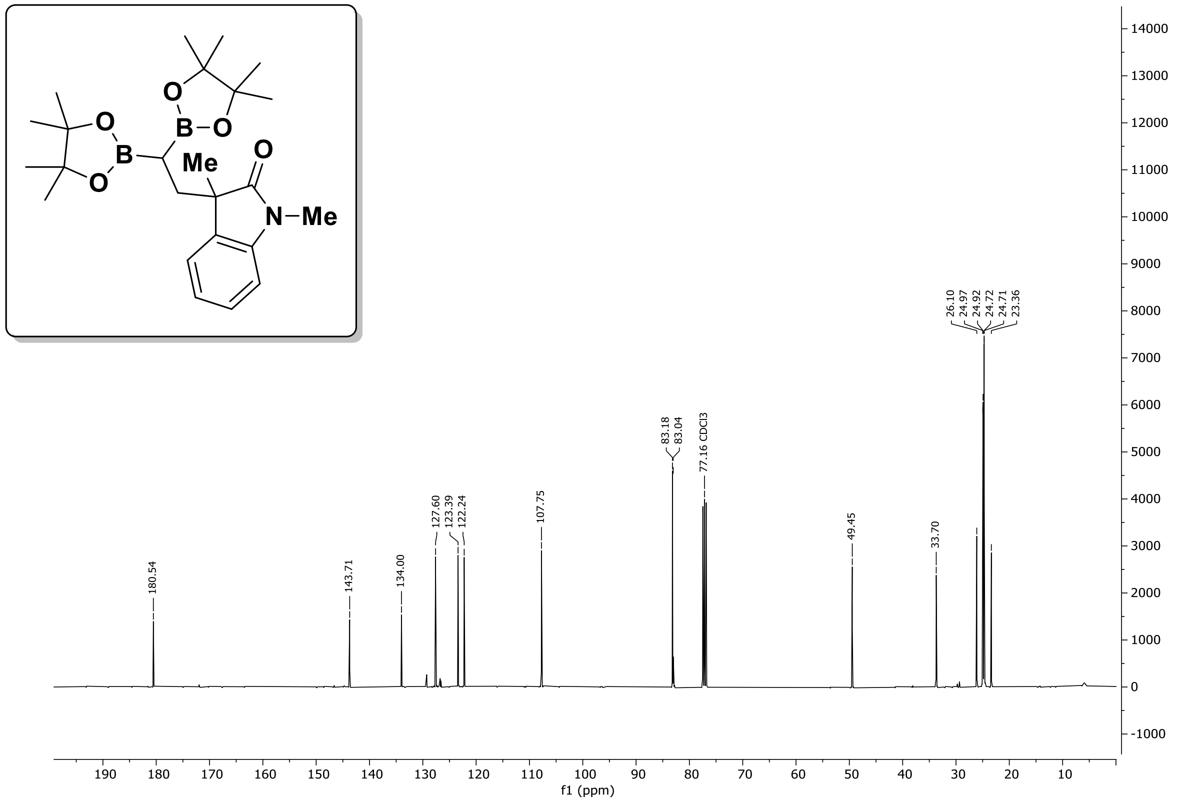

^{1}H-NMR of 25 (400 MHz, $CDCl_3$)

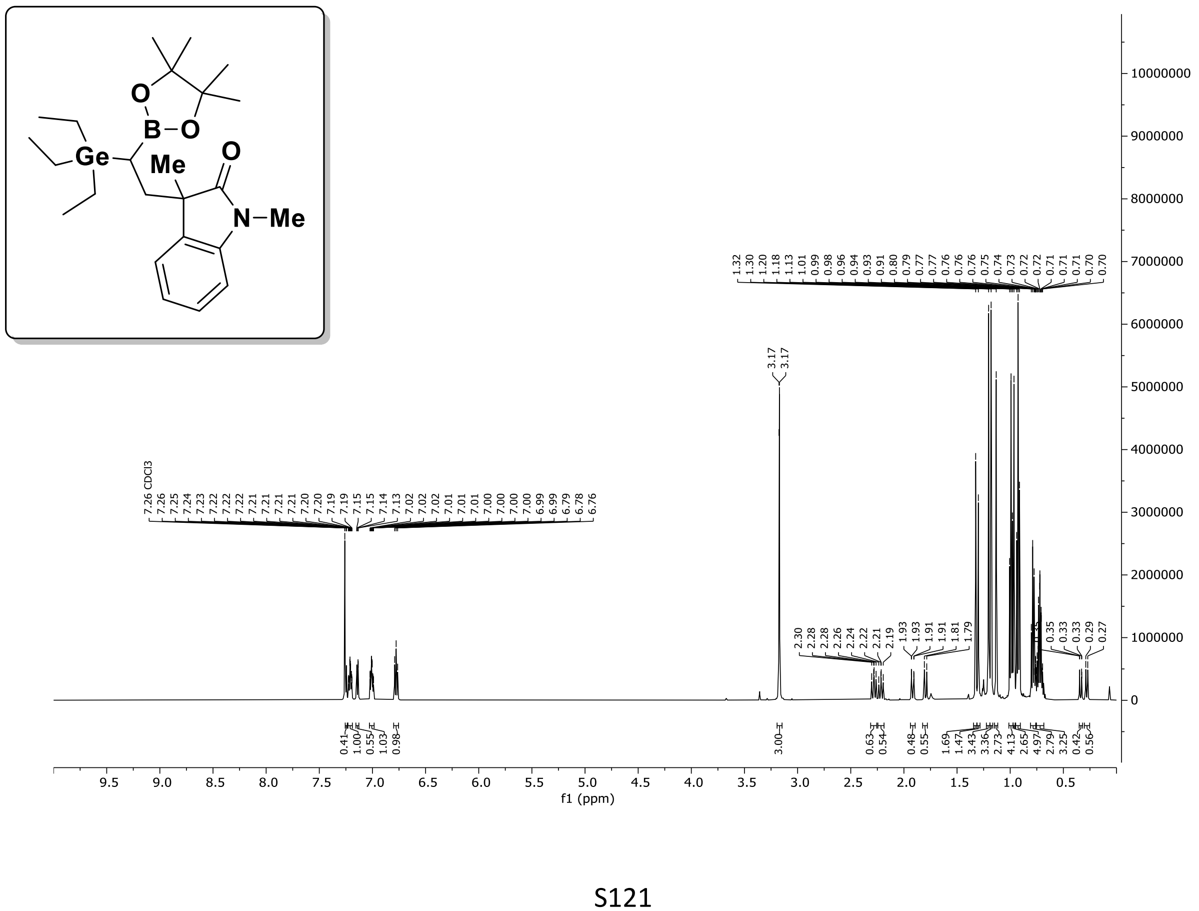

^{13}C-NMR of 25 (101 MHz, $CDCl_3$)

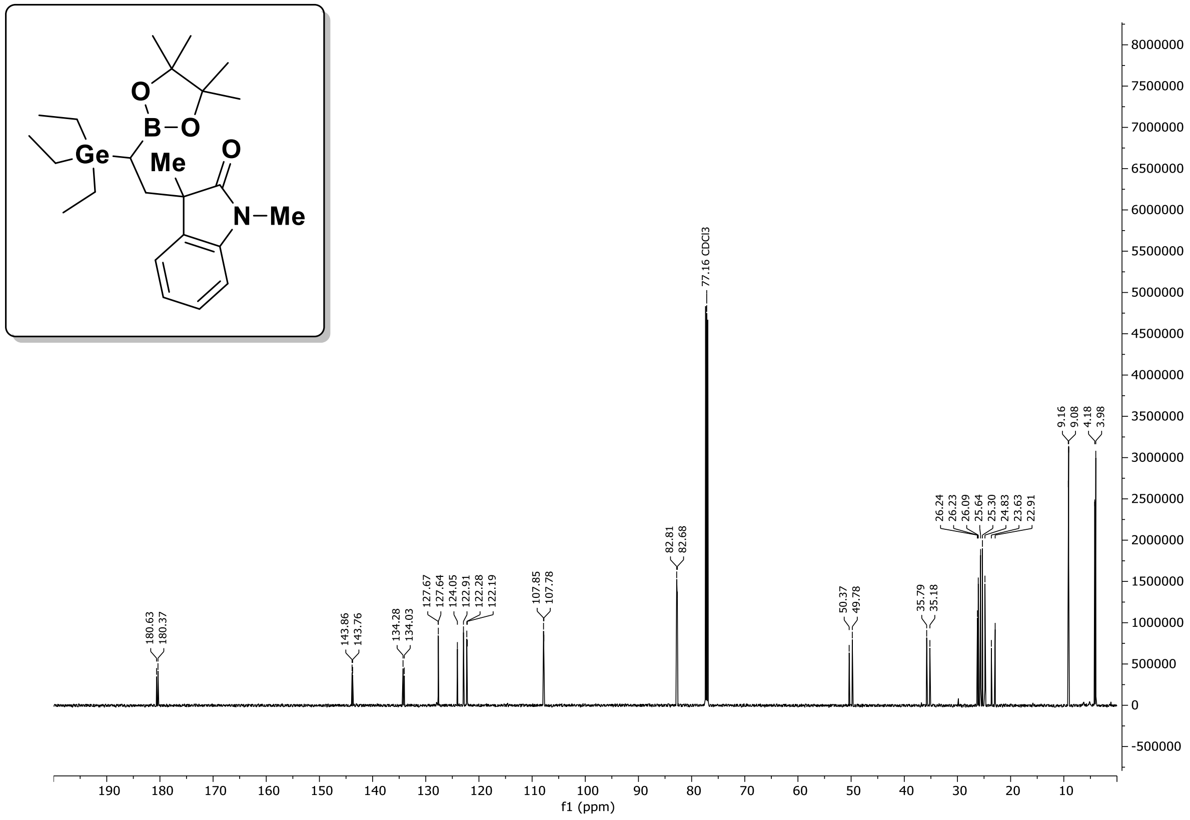

^{1}H NMR of 26 (600 MHz, $CDCl_3$)

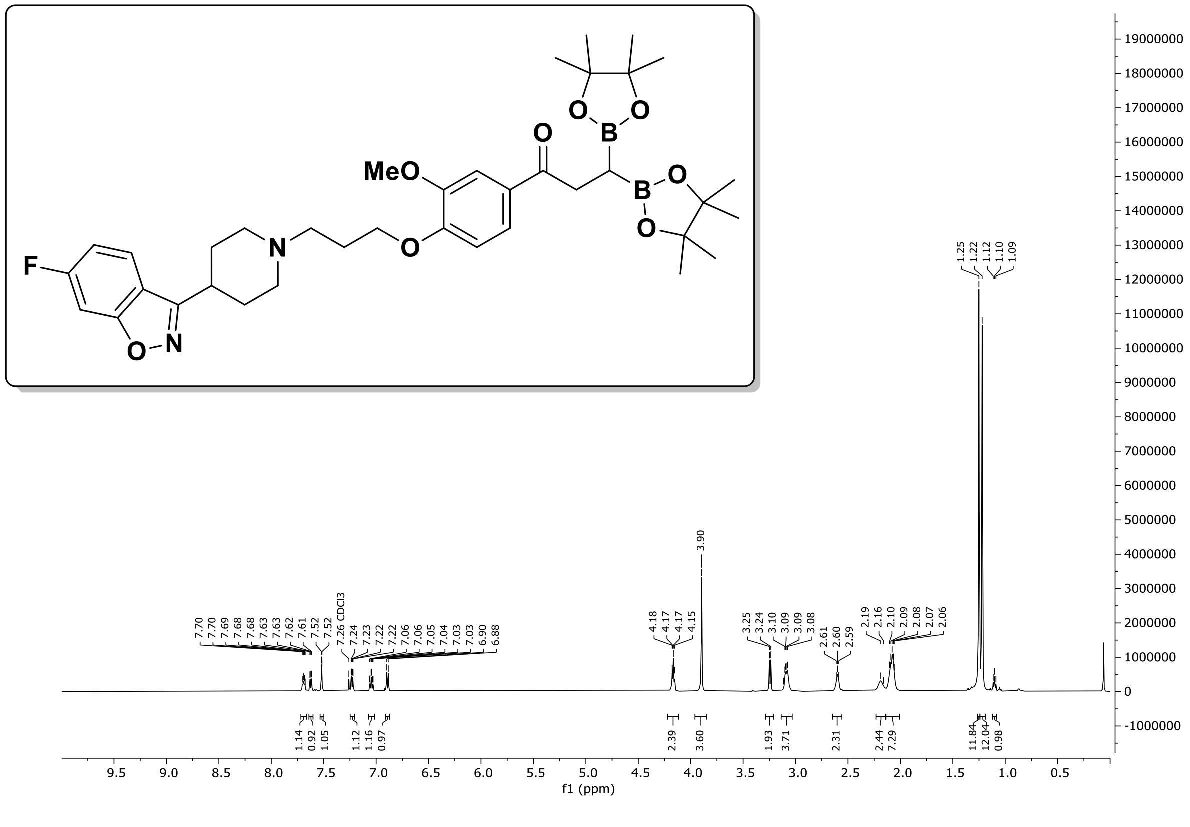

^{13}C NMR of 26 (150.9 MHz, $CDCl_3$)

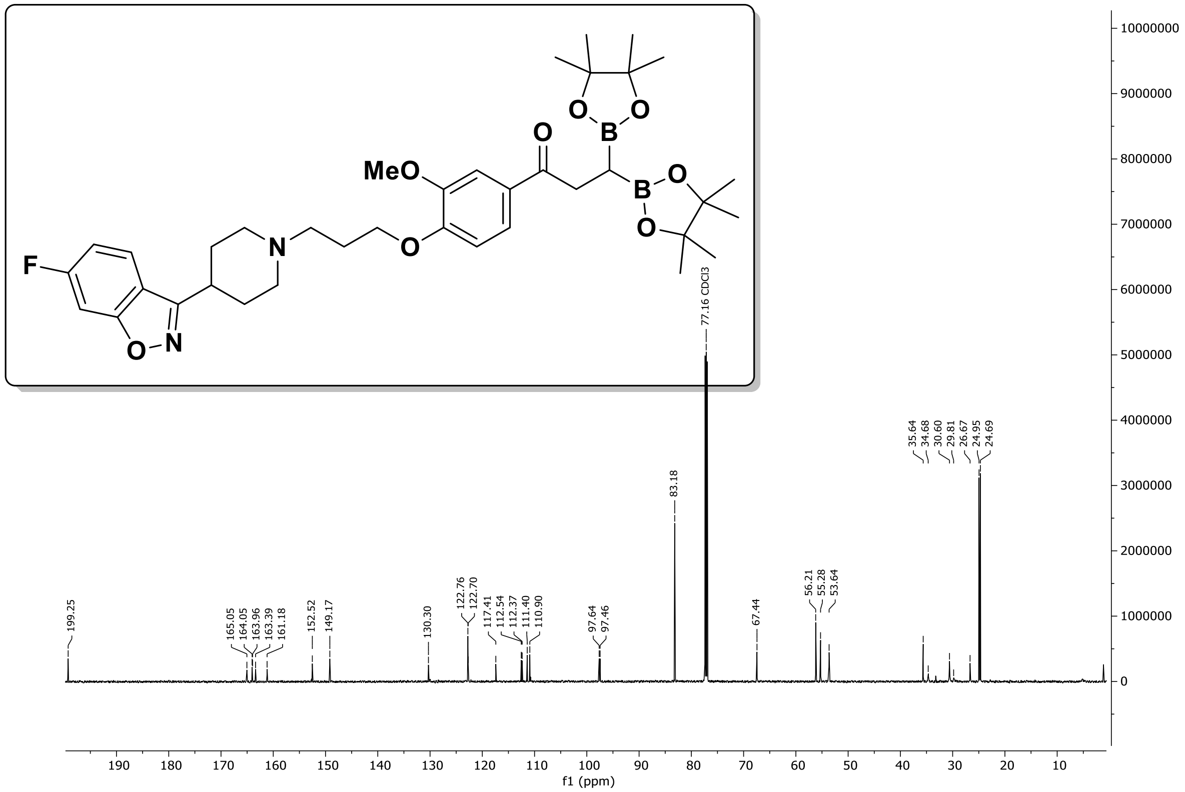

^{19}F NMR of 26 (564.7 MHz, $CDCl_3$)

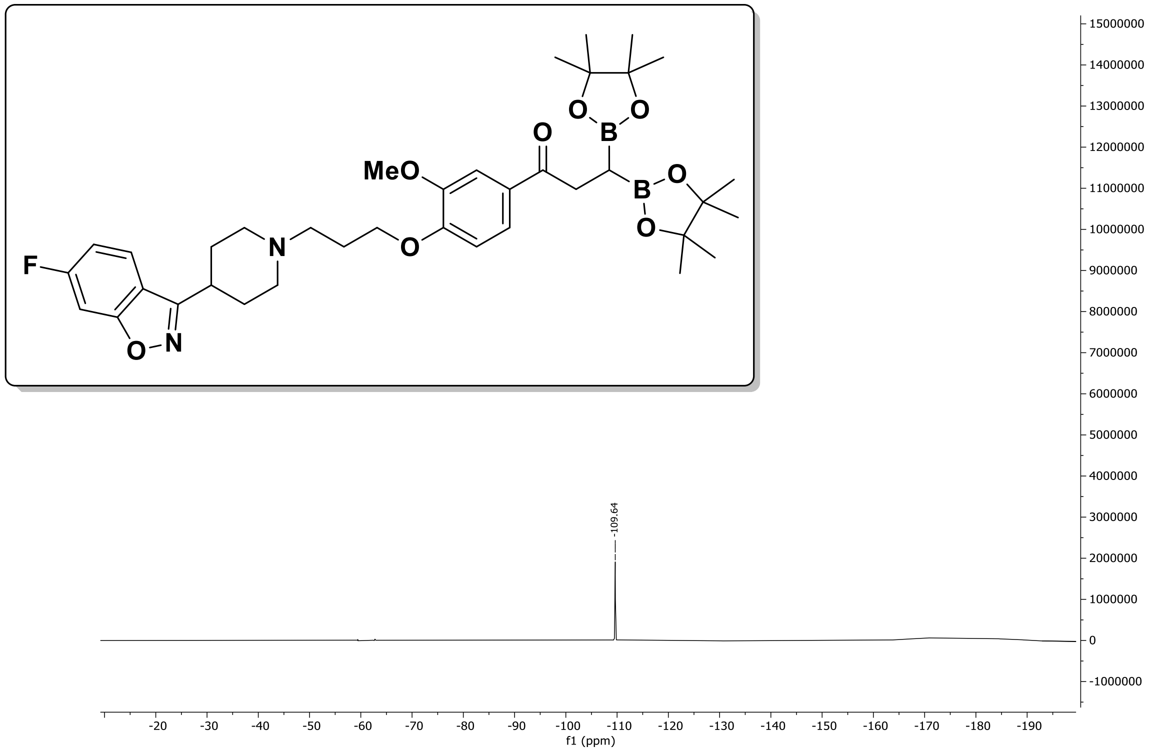

^{1}H-NMR of S23 (400 MHz, $CDCl_3$)

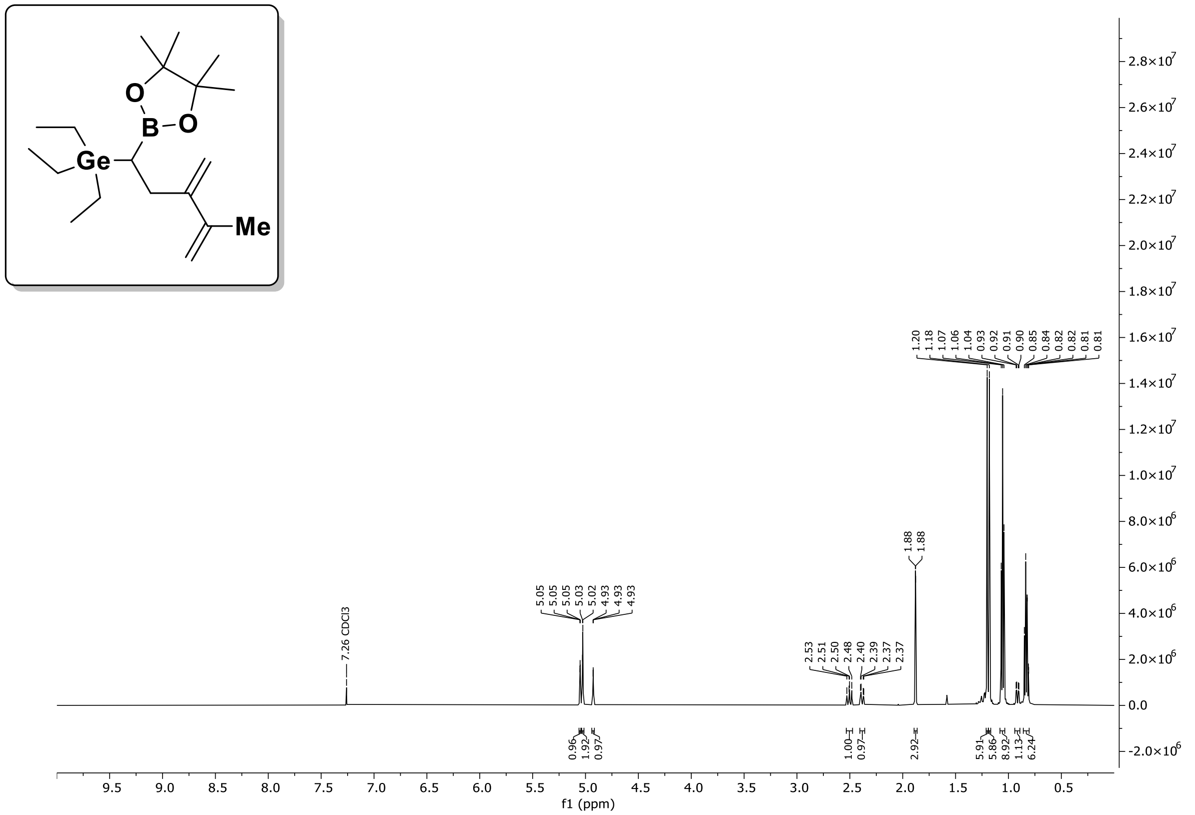

^{13}C-NMR of S23 (101 MHz, $CDCl_3$)

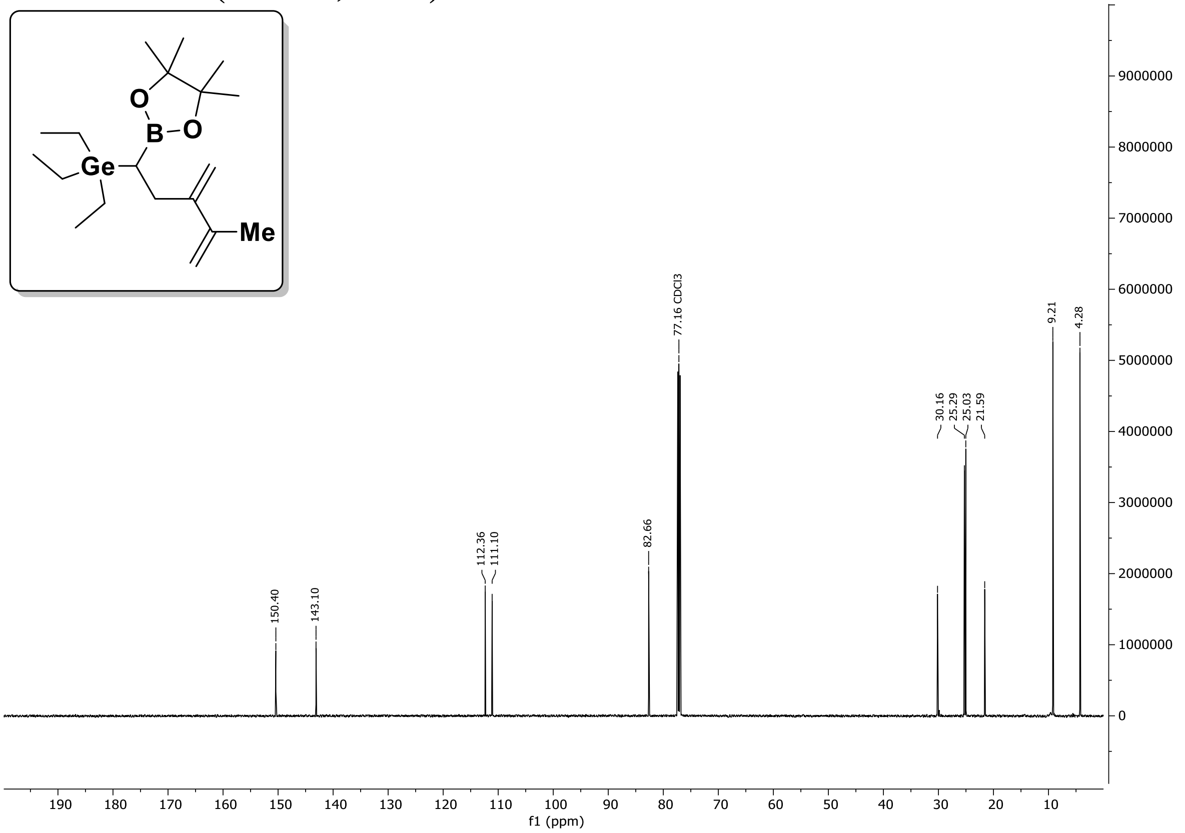

^{1}H-NMR of 27 (400 MHz, $CDCl_3$)

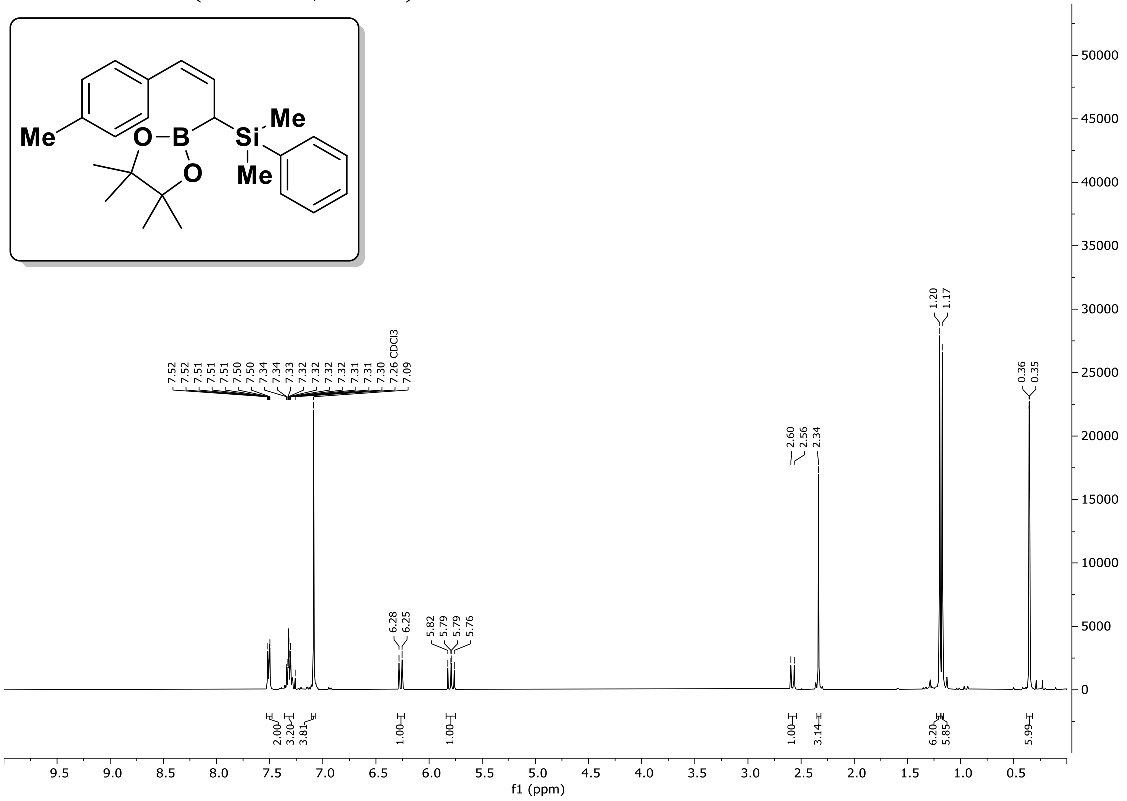

^{13}C-NMR of 27 (101 MHz, $CDCl_3$)

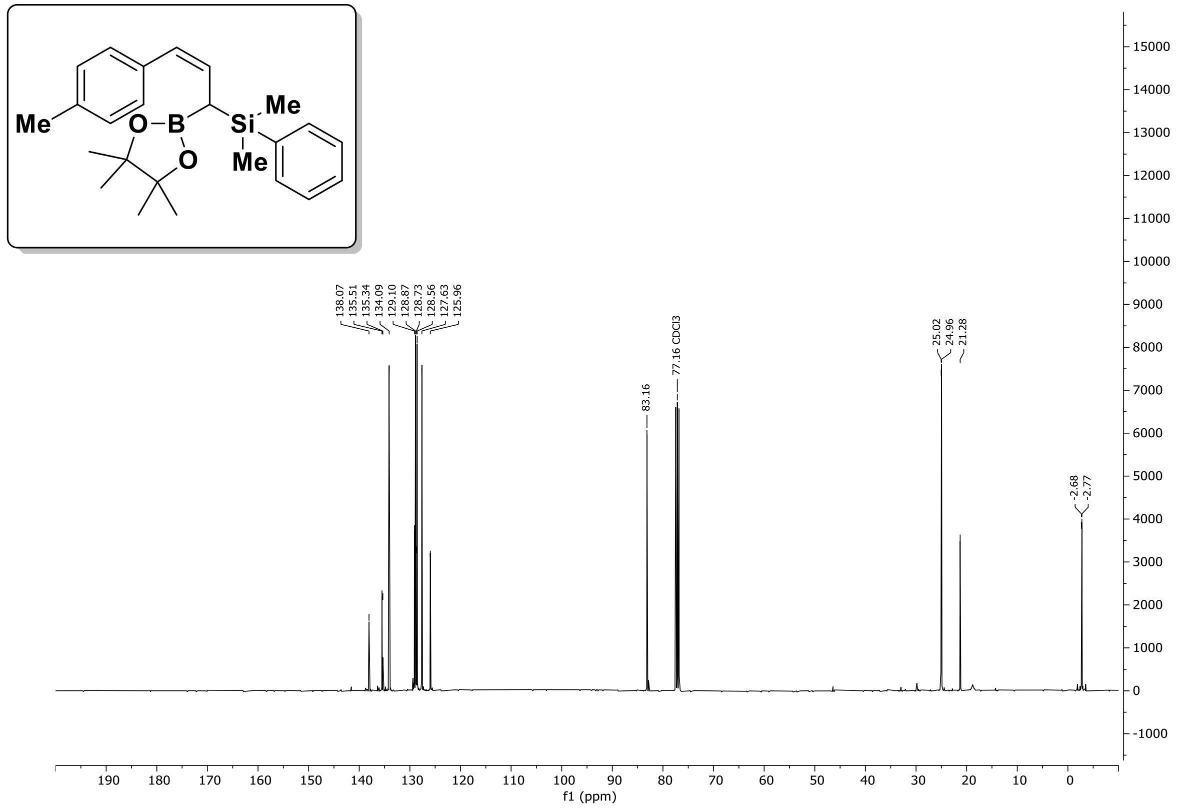

^{1}H-NMR of 28 (400 MHz, $CDCl_3$)

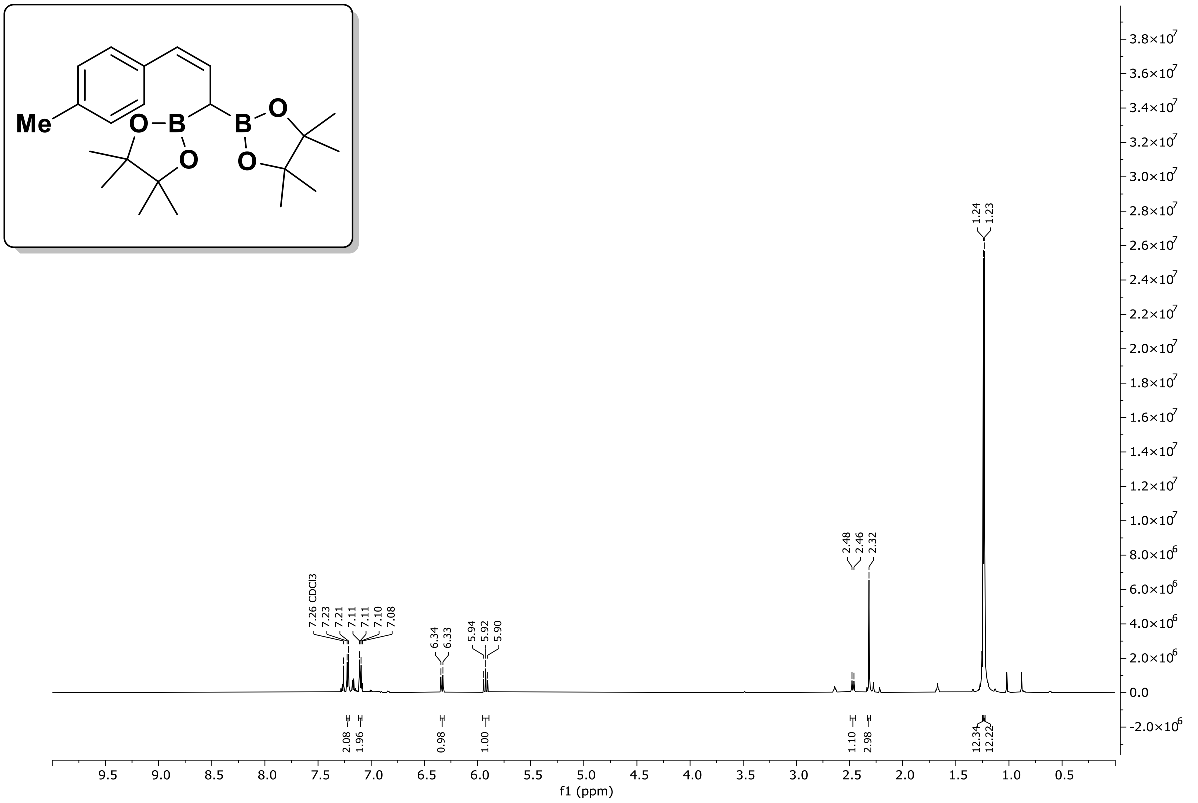

^{13}C-NMR of 28 (101 MHz, $CDCl_3$)

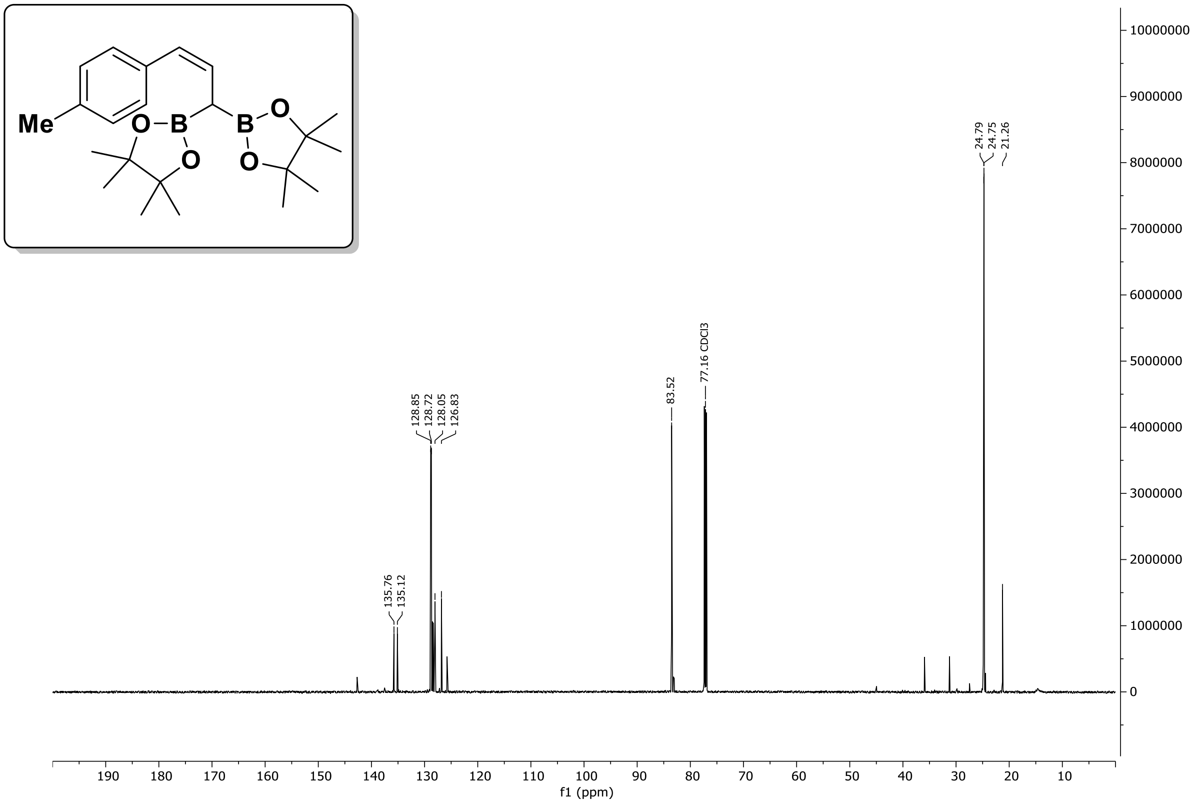

^{1}H-NMR of 30 (400 MHz, $CDCl_3$)

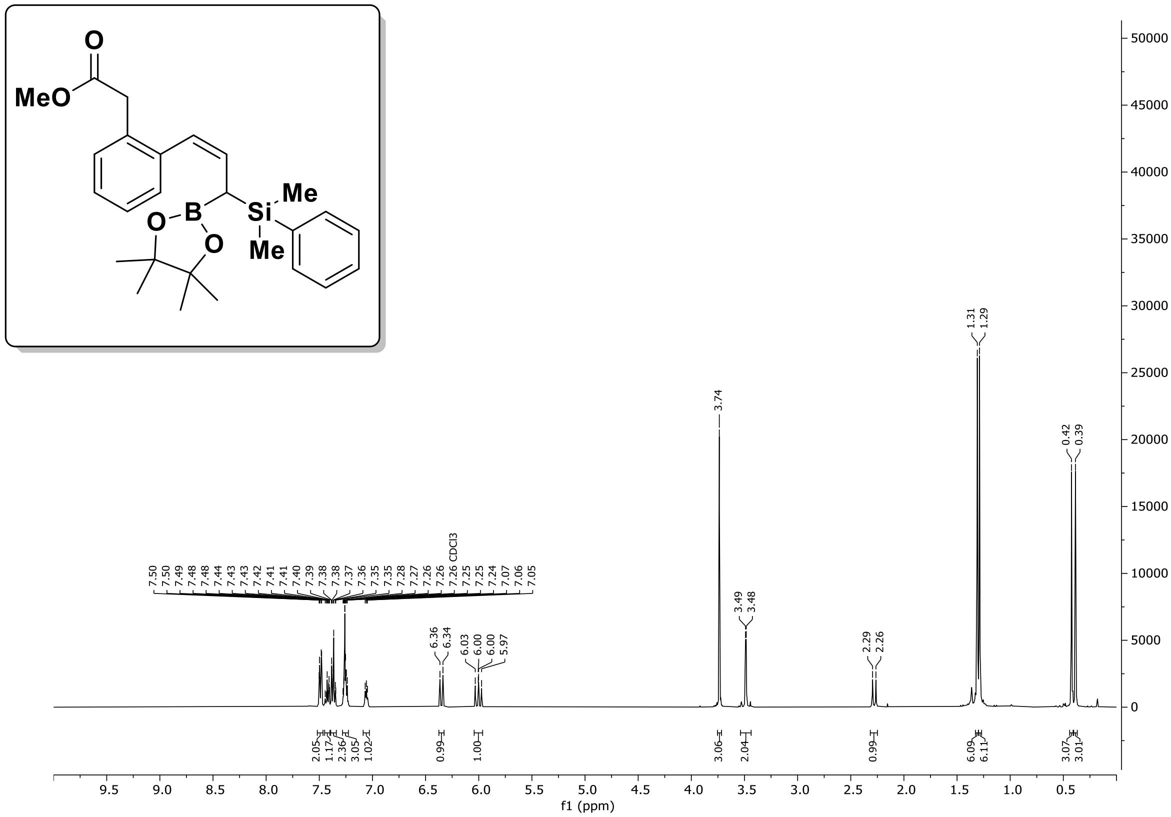

^{13}C-NMR of 30 (101 MHz, $CDCl_3$)

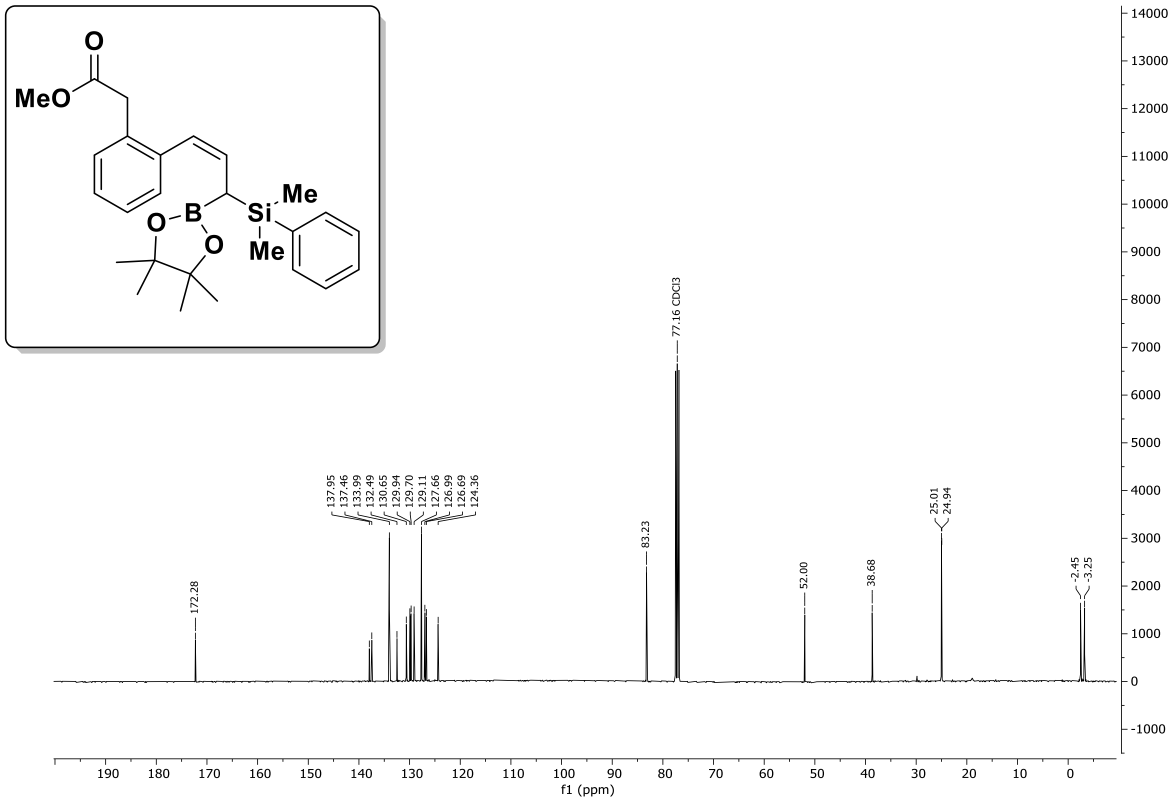

^{1}H-NMR of 31 (400 MHz, $CDCl_3$)

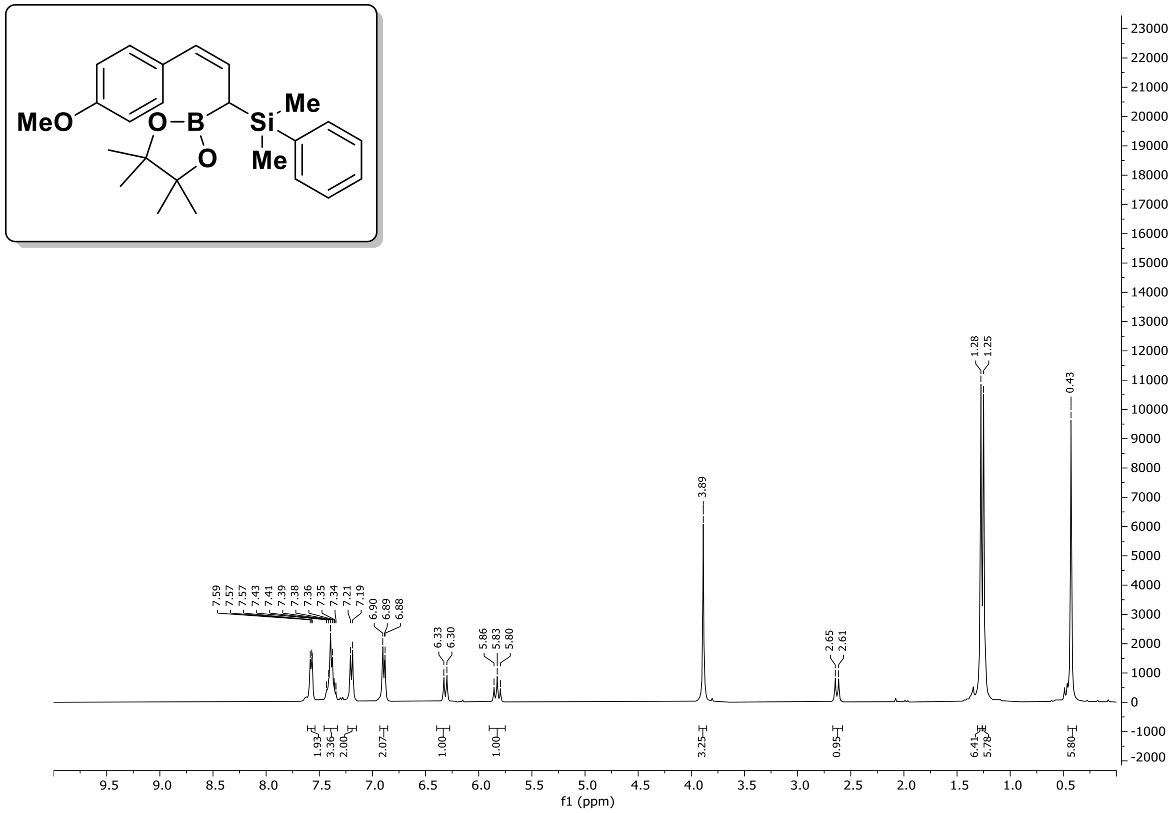

^{13}C-NMR of 31 (101 MHz, $CDCl_3$)

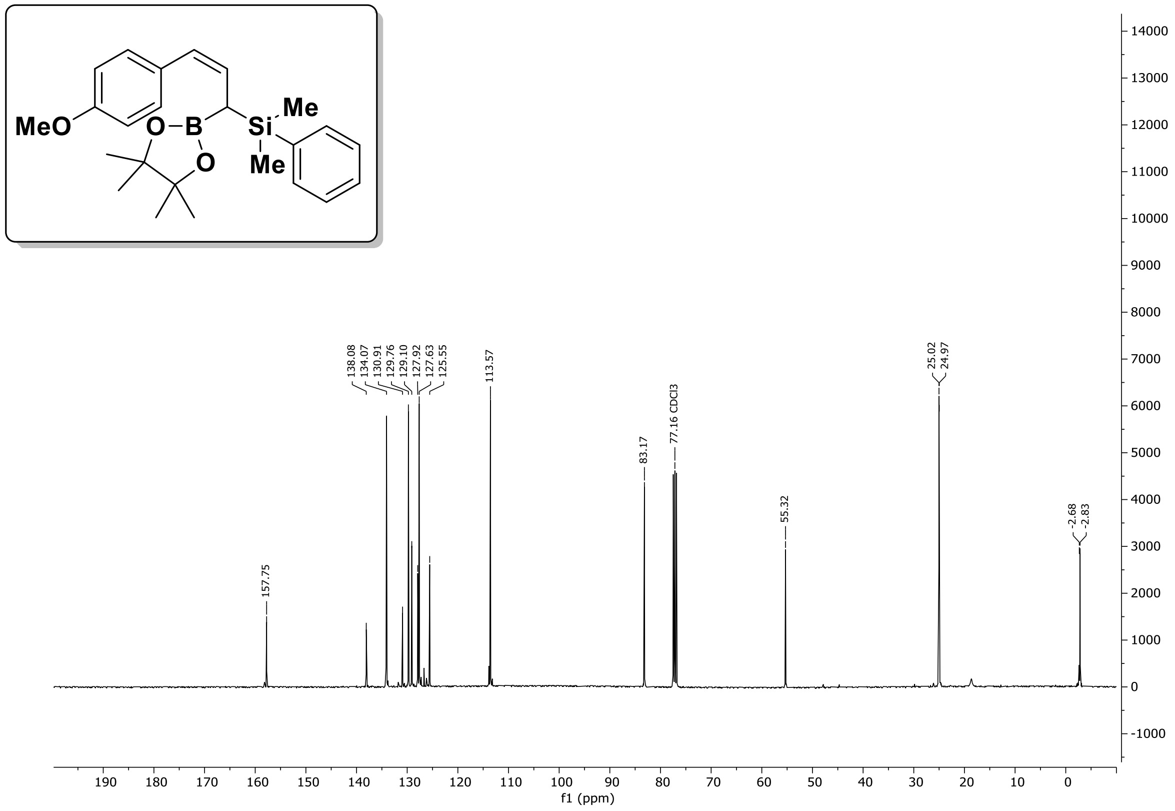

^{1}H-NMR of 32 (400 MHz, $CDCl_3$)

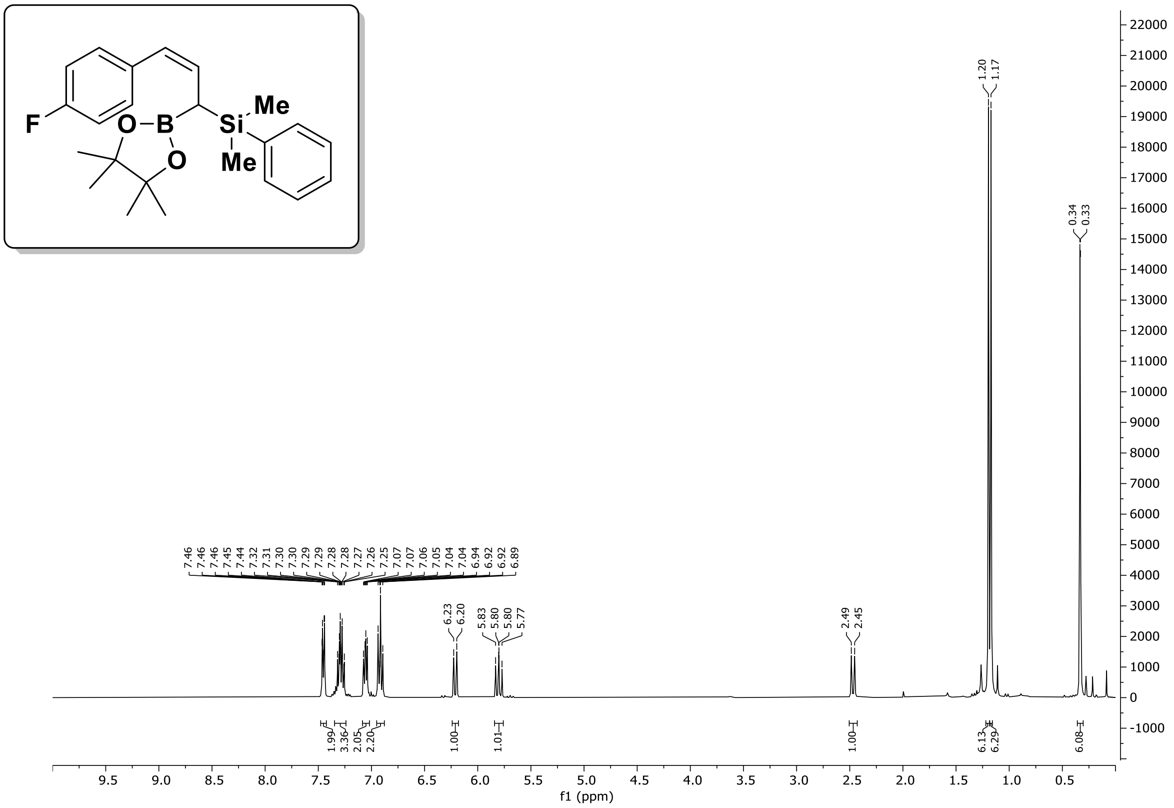

^{13}C-NMR of 32 (101 MHz, $CDCl_3$)

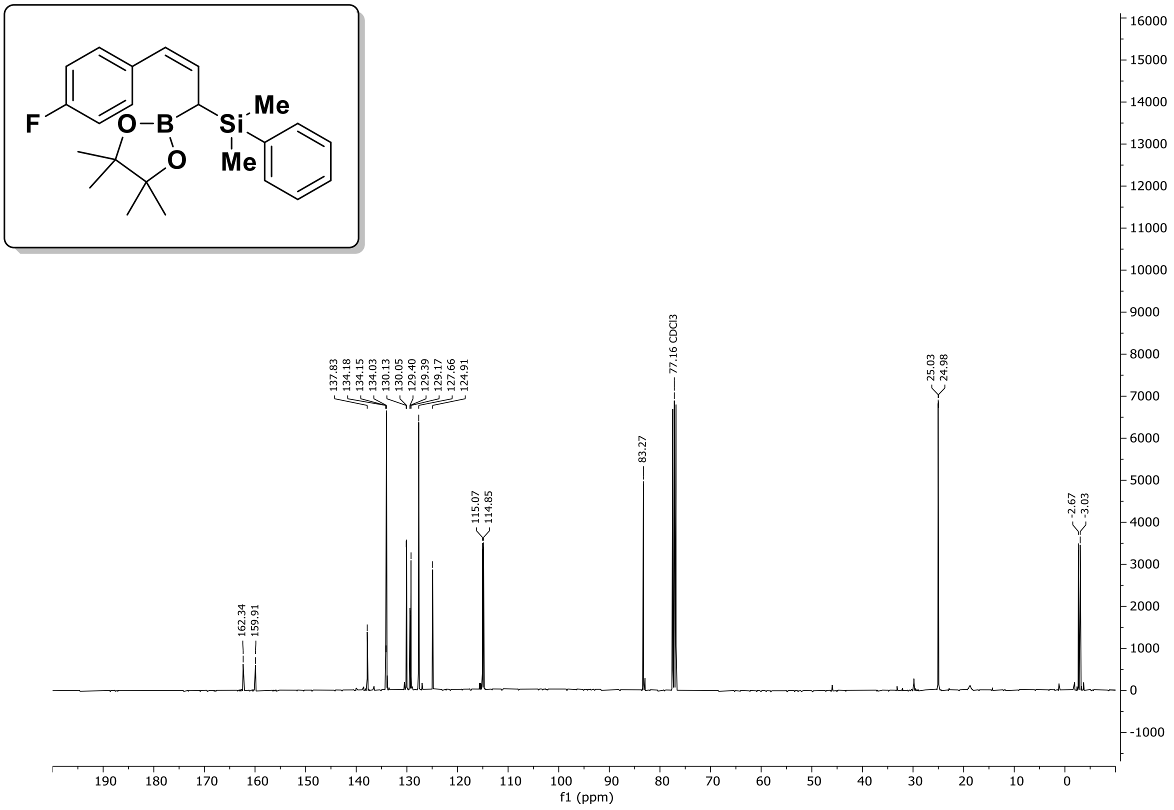

^{19}F NMR of 32 (376 MHz, $CDCl_3$)

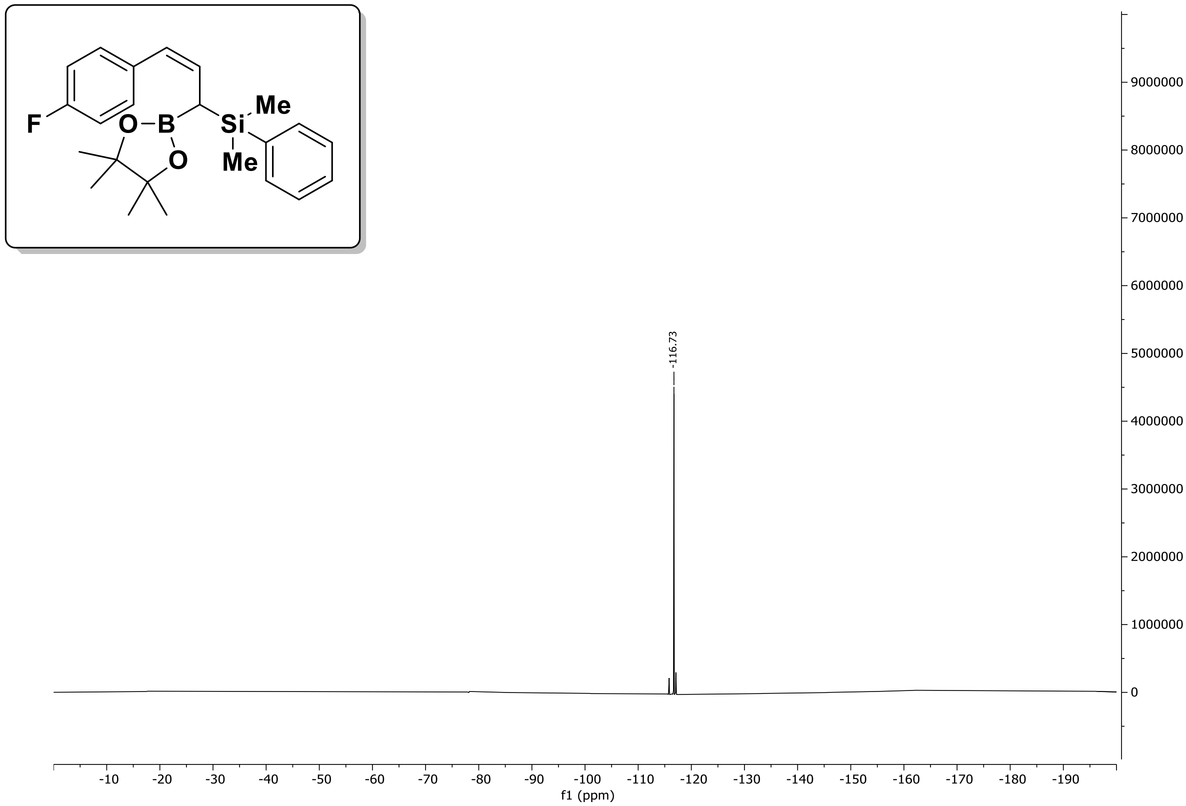

^{1}H-NMR of 33 (400 MHz, $CDCl_3$)

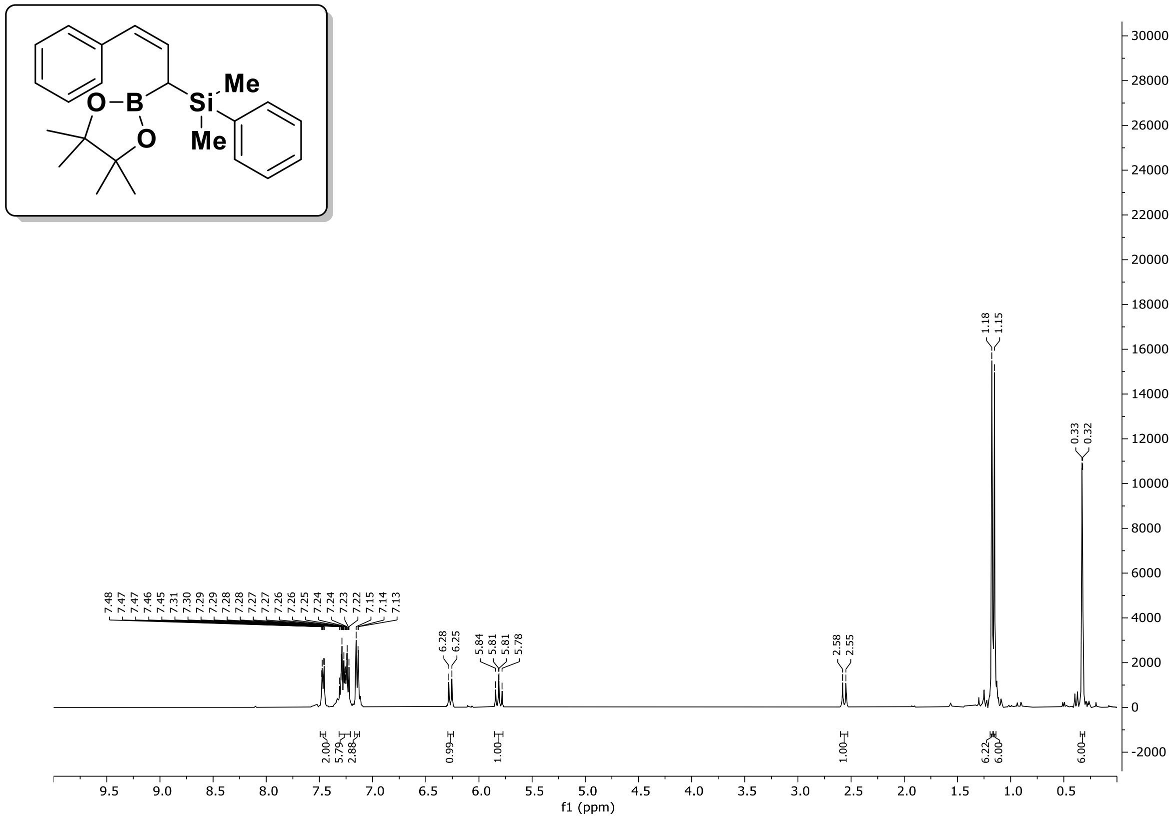

^{13}C-NMR of 33 (101 MHz, $CDCl_3$)

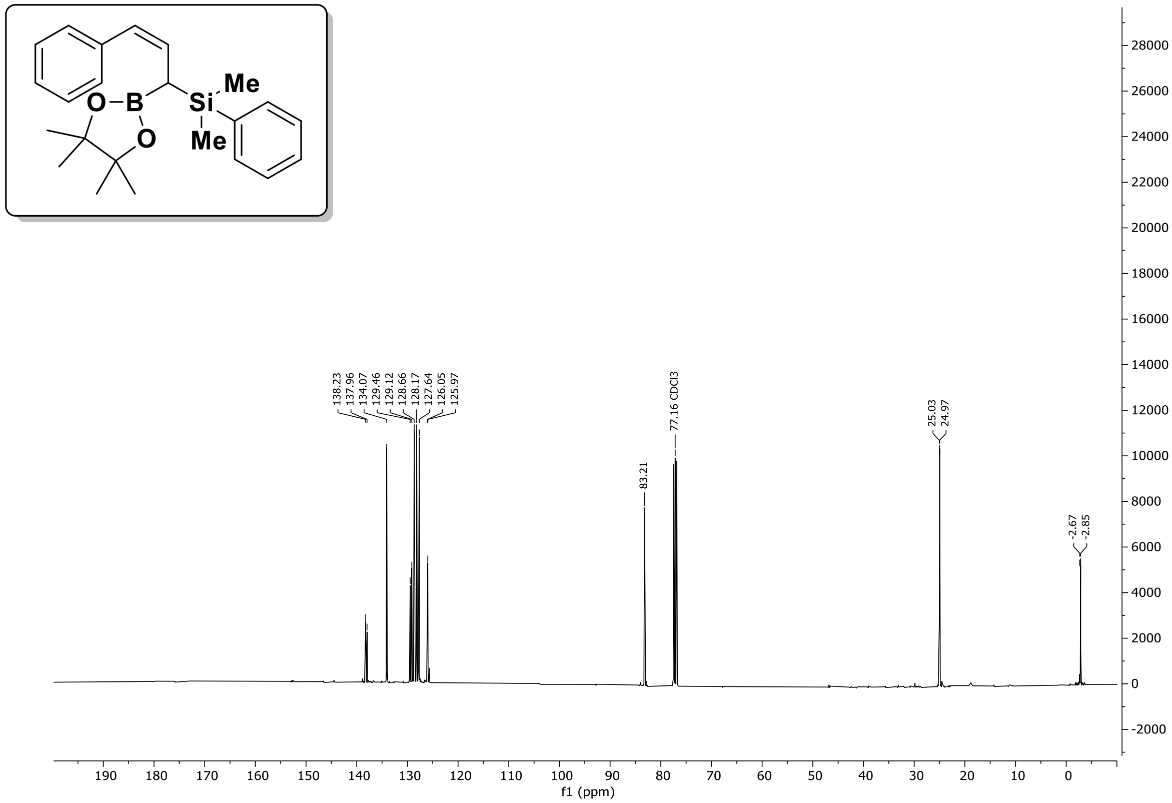

^{1}H-NMR of 34 (400 MHz, $CDCl_3$)

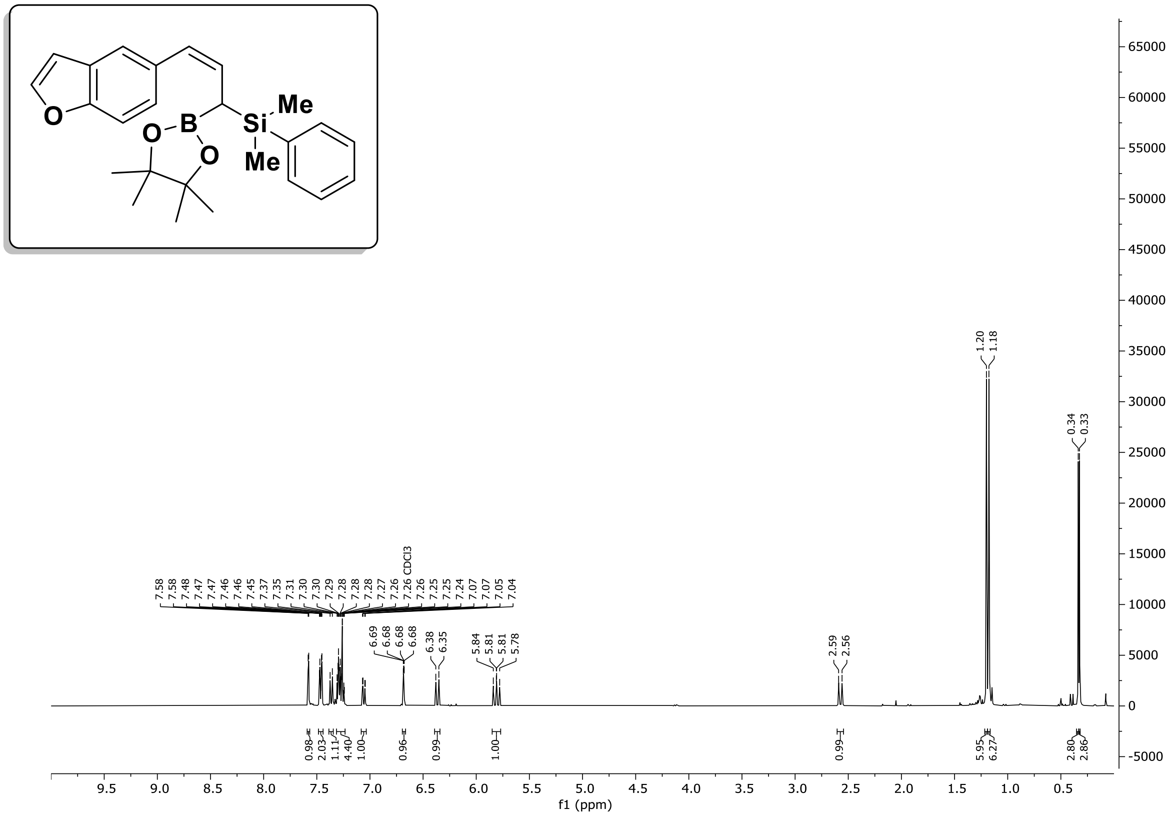

^{13}C-NMR of 34 (101 MHz, CDCl$_3$)

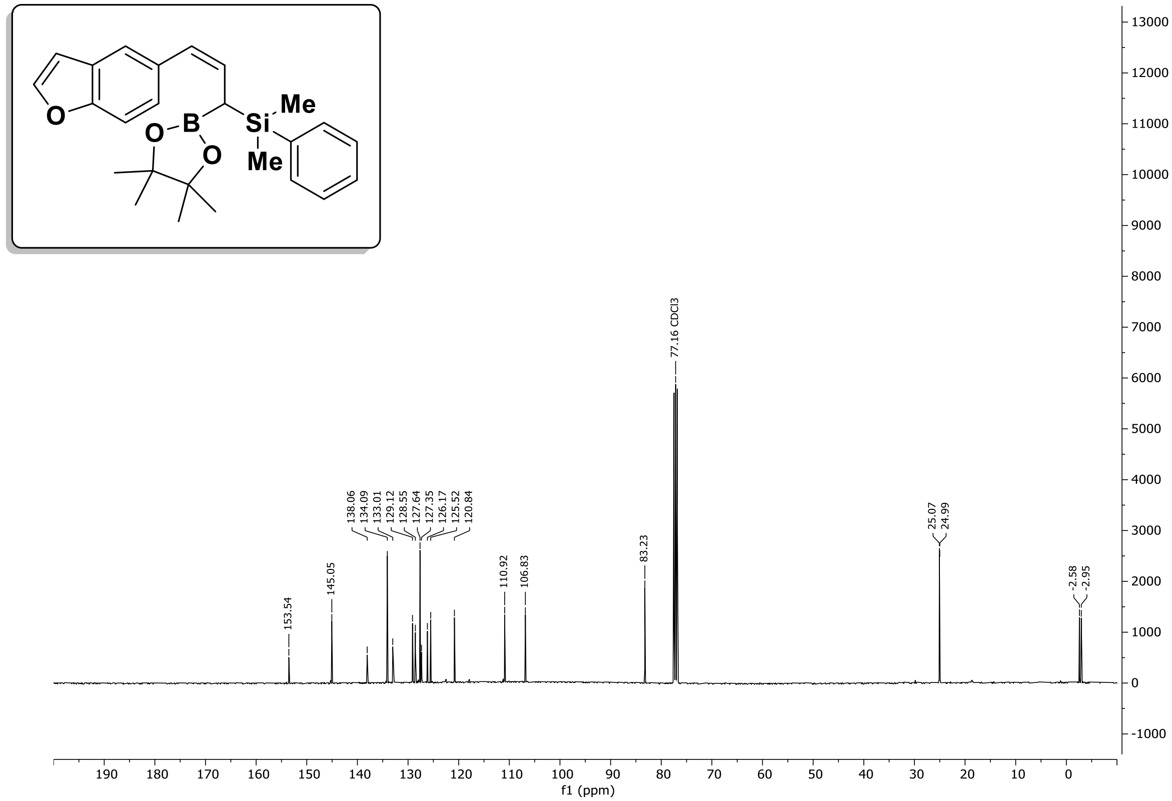

^{1}H-NMR of 35 (400 MHz, CDCl$_3$)

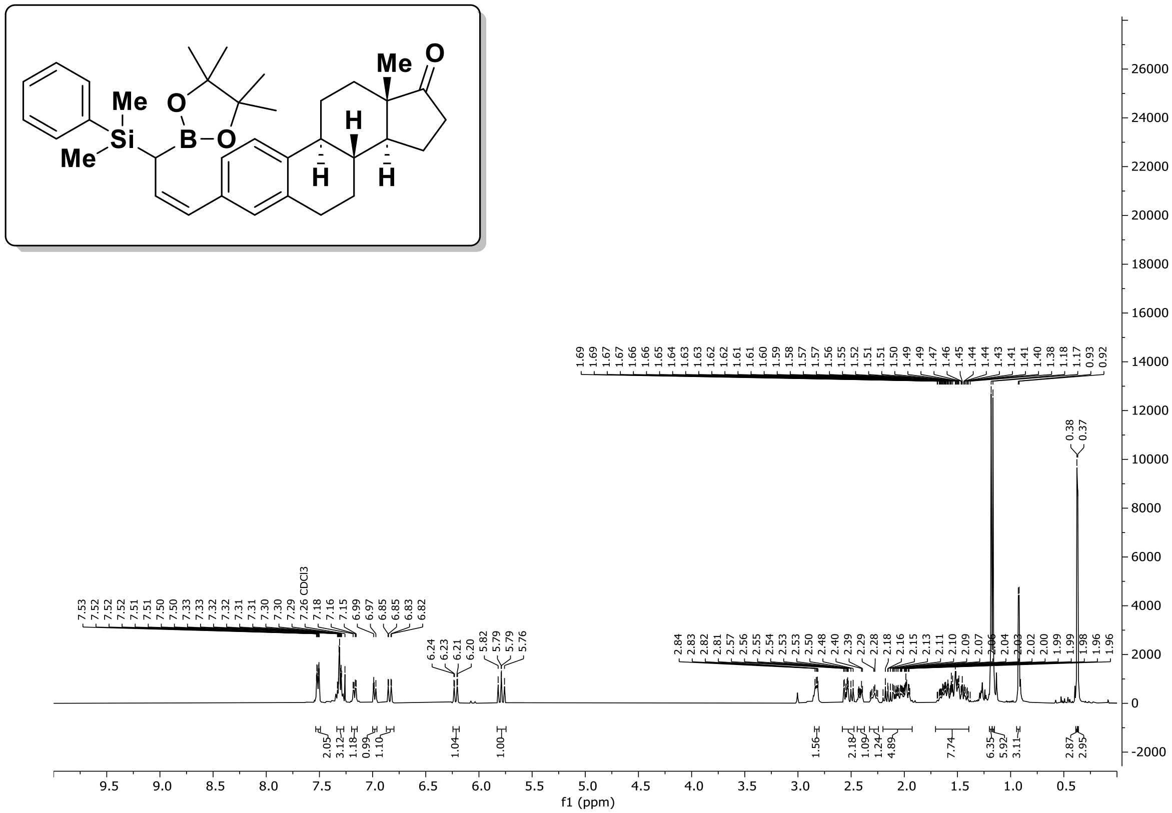

^{13}C-NMR of 35 (101 MHz, $CDCl_3$)

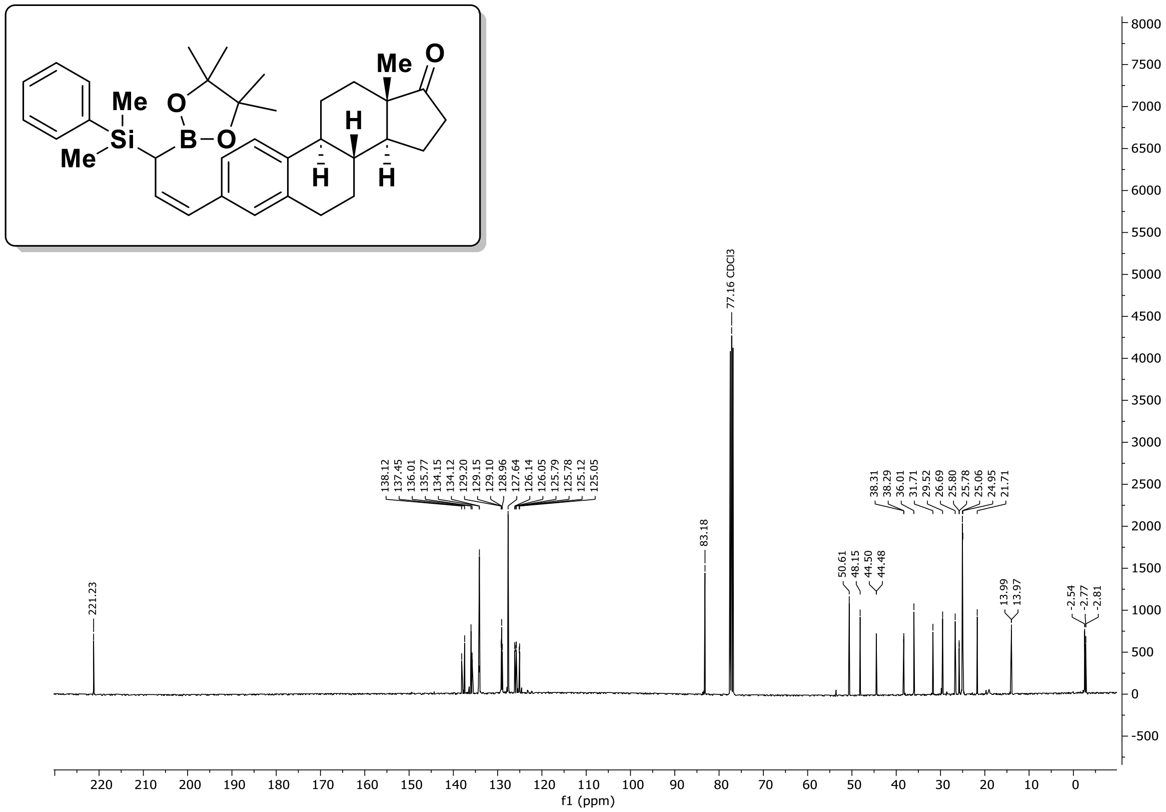

^{1}H-NMR of 36 (400 MHz, $CDCl_3$)

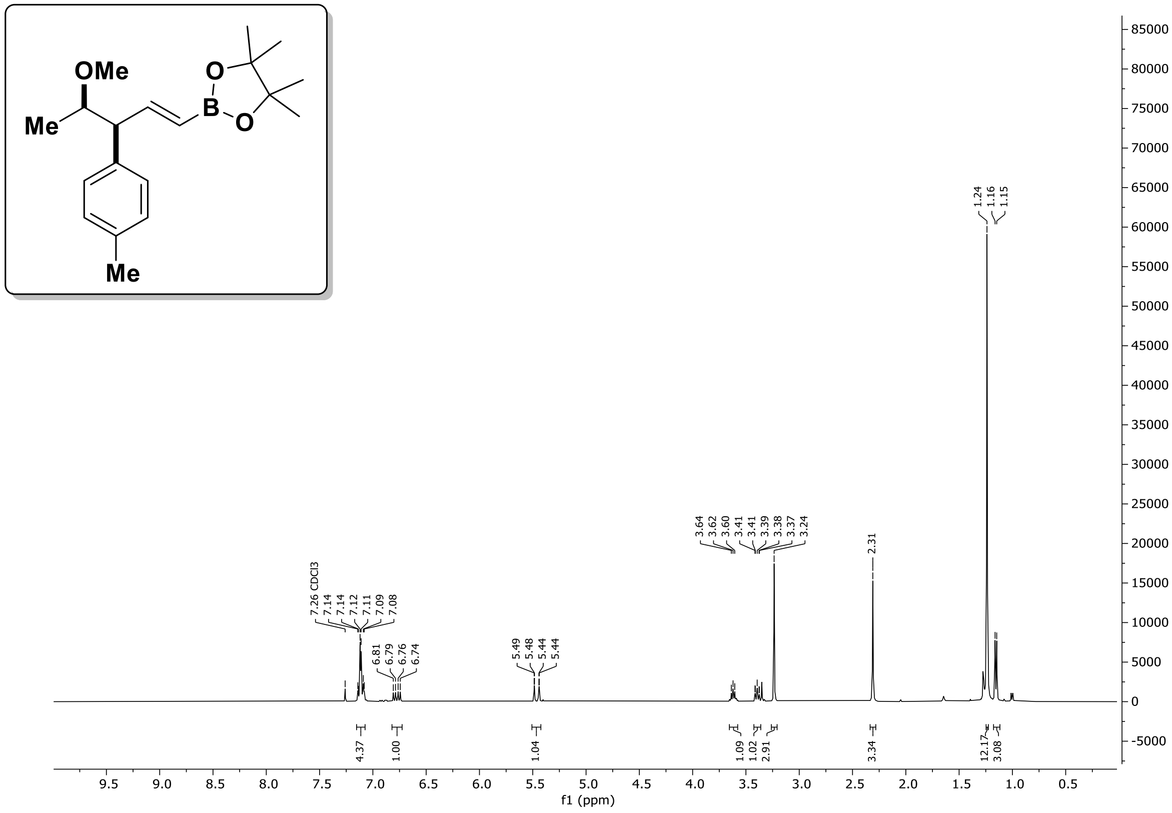

^{13}C-NMR of 36 (101 MHz, $CDCl_3$)

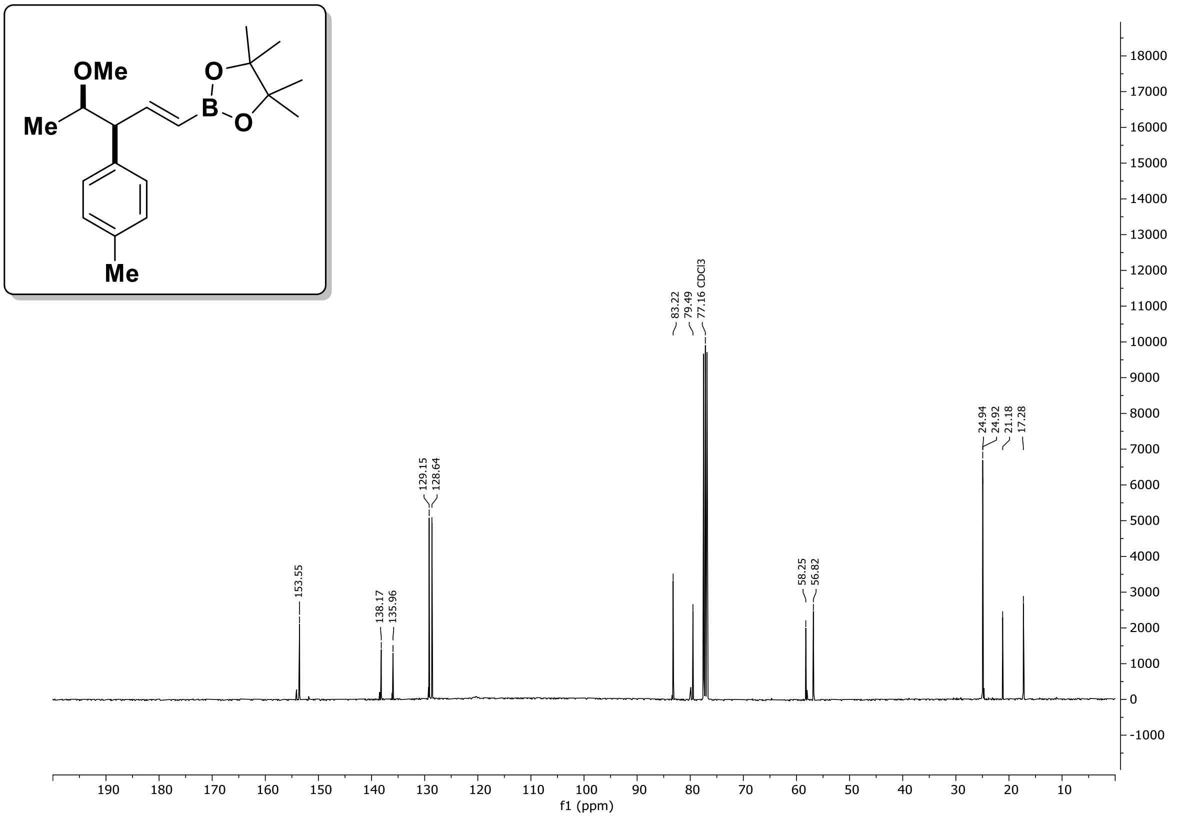

^{1}H-NMR of 37 (400 MHz, $CDCl_3$)

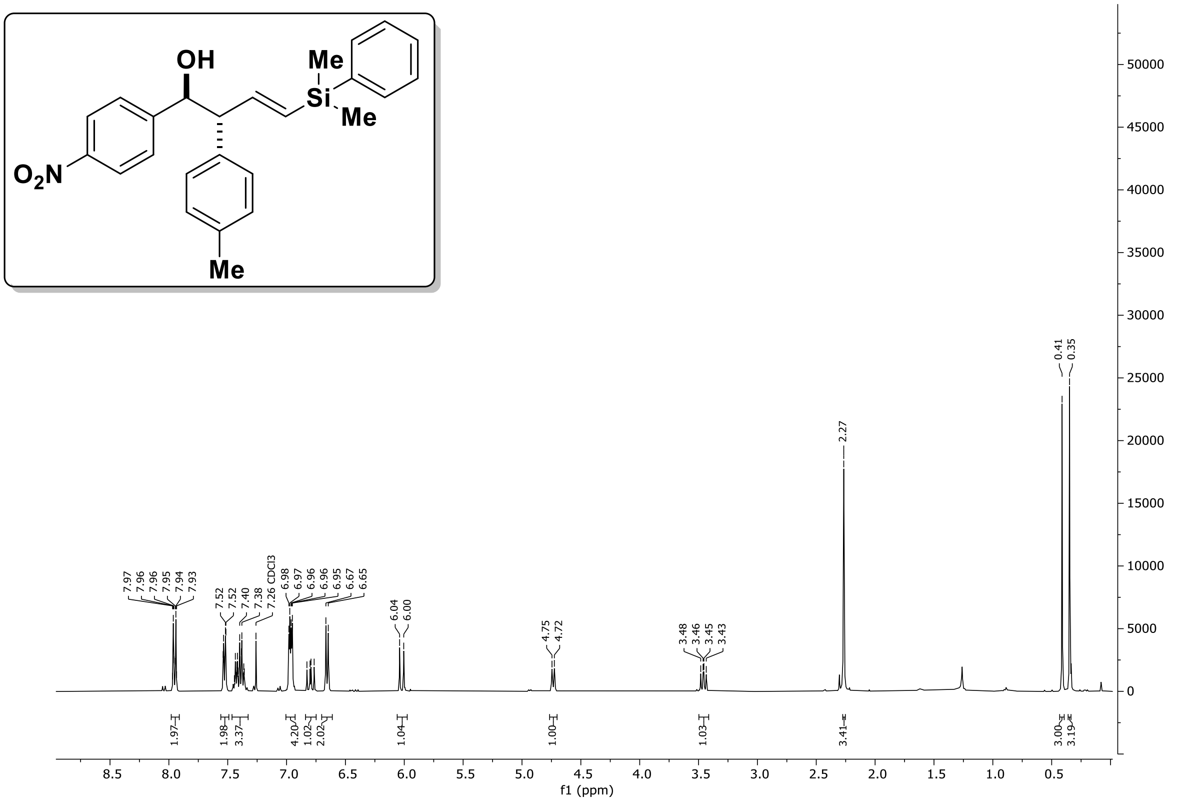

^{13}**C-NMR of 37** (101 MHz, $CDCl_3$)

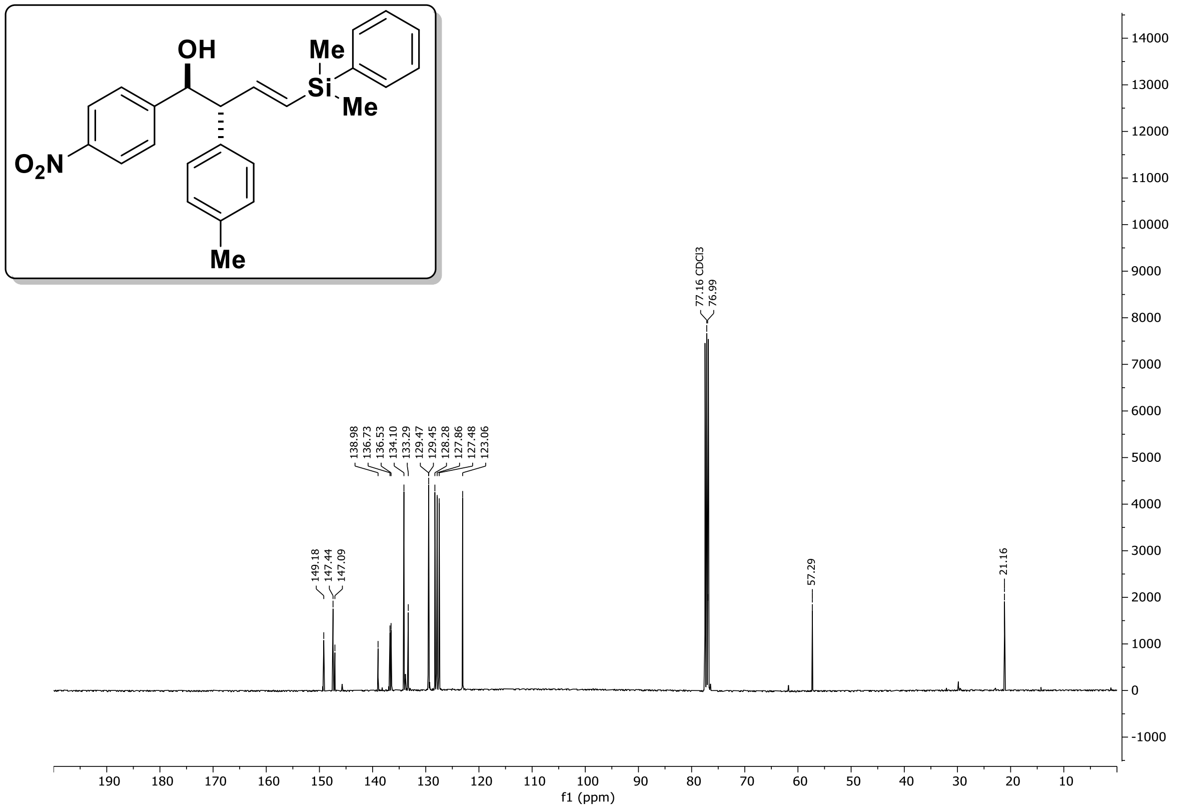

^{1}**H-NMR of 38** (400 MHz, $CDCl_3$)

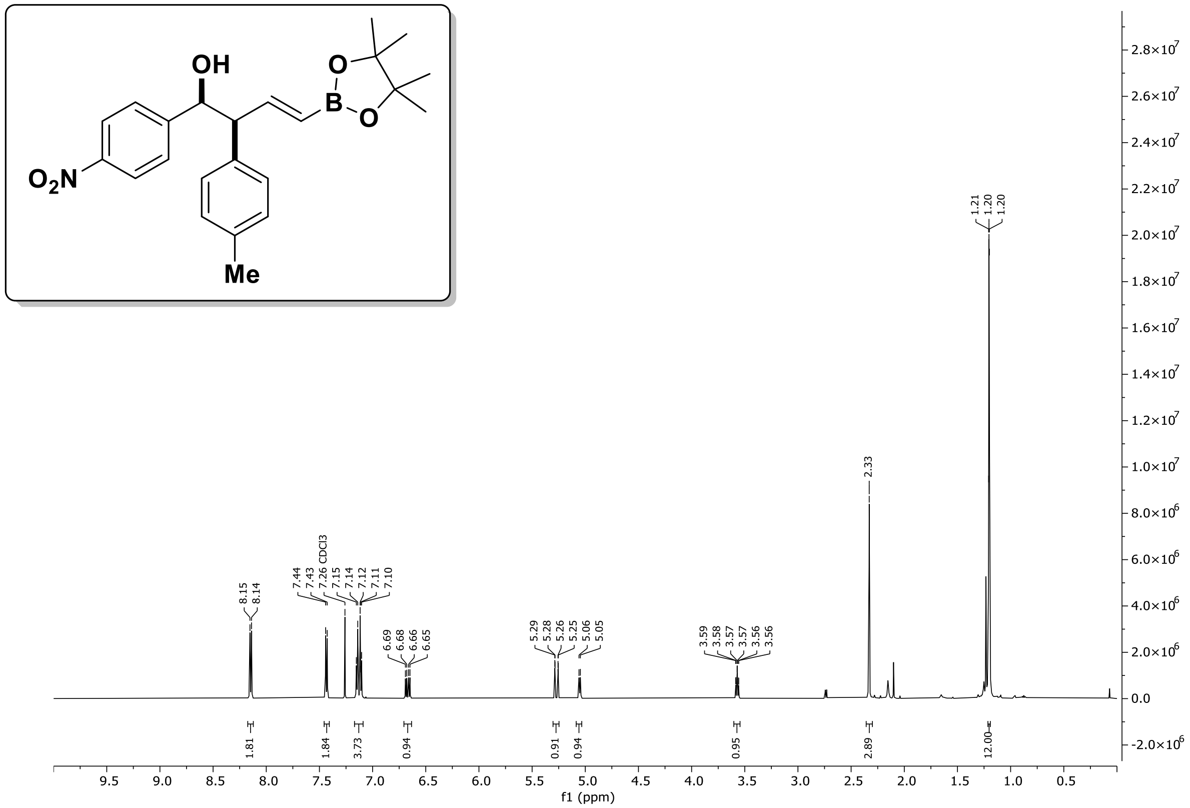

^{13}C-NMR of 38 (101 MHz, $CDCl_3$)

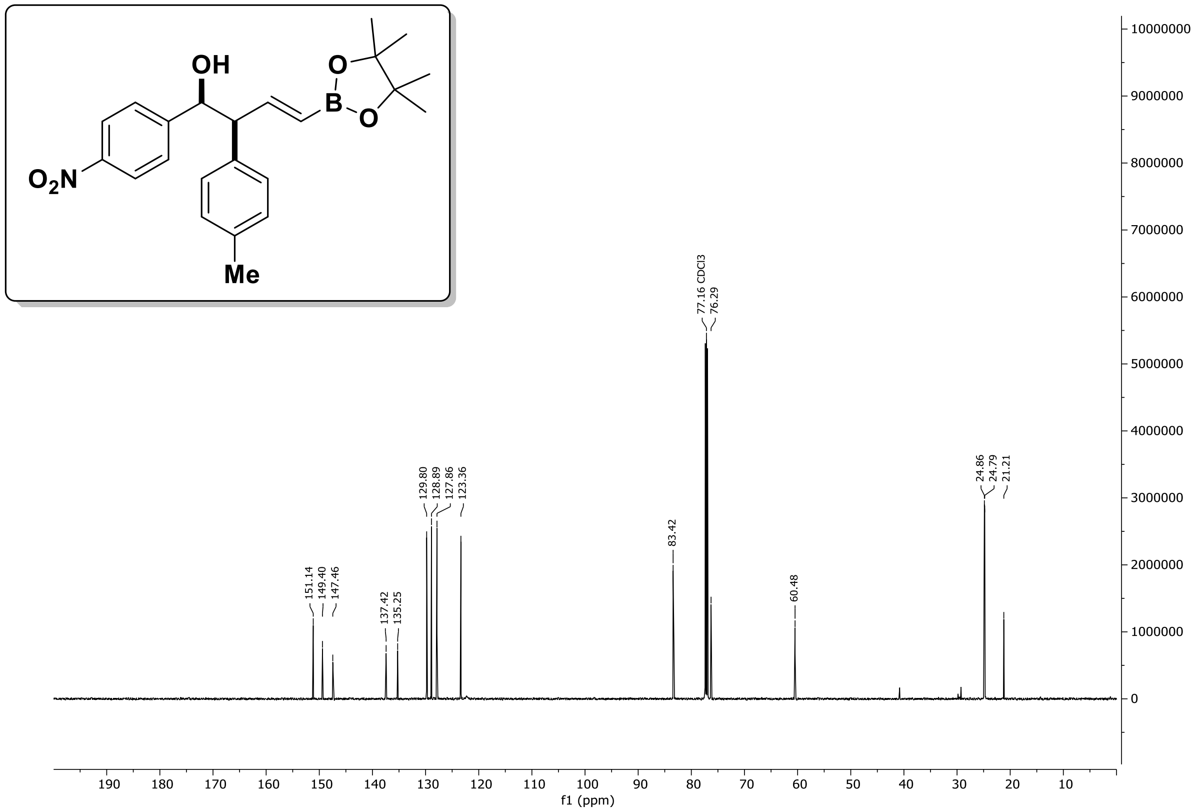

^{1}H-NMR of 39 (400 MHz, $CDCl_3$)

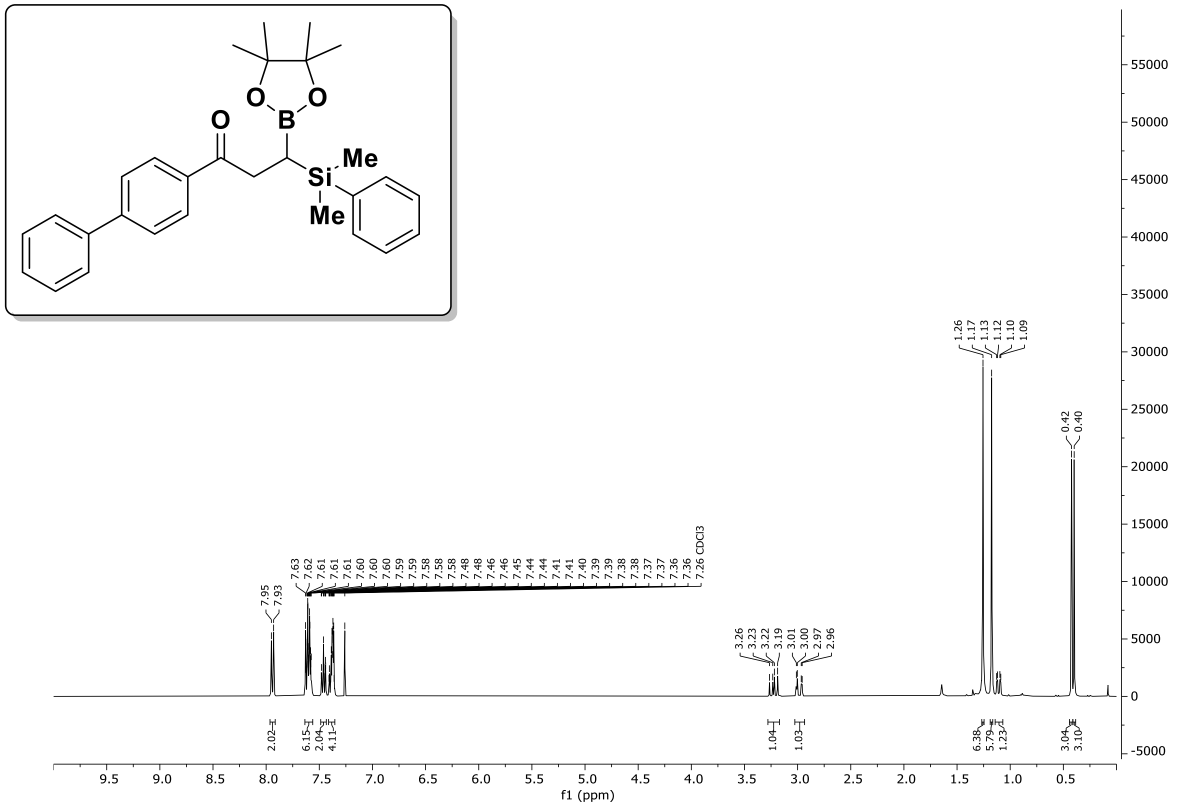

^{13}C-NMR of 39 (101 MHz, $CDCl_3$)

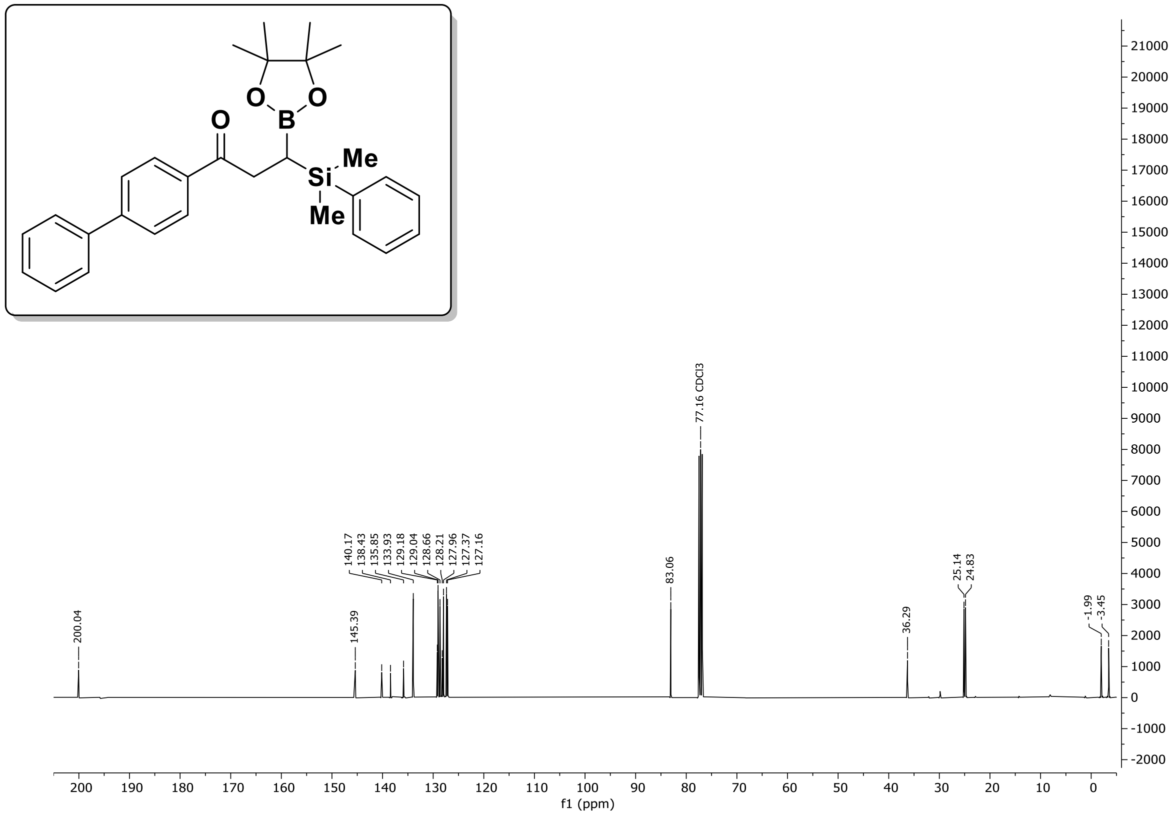

^{1}H NMR of 40 (400 MHz, $CDCl_3$)

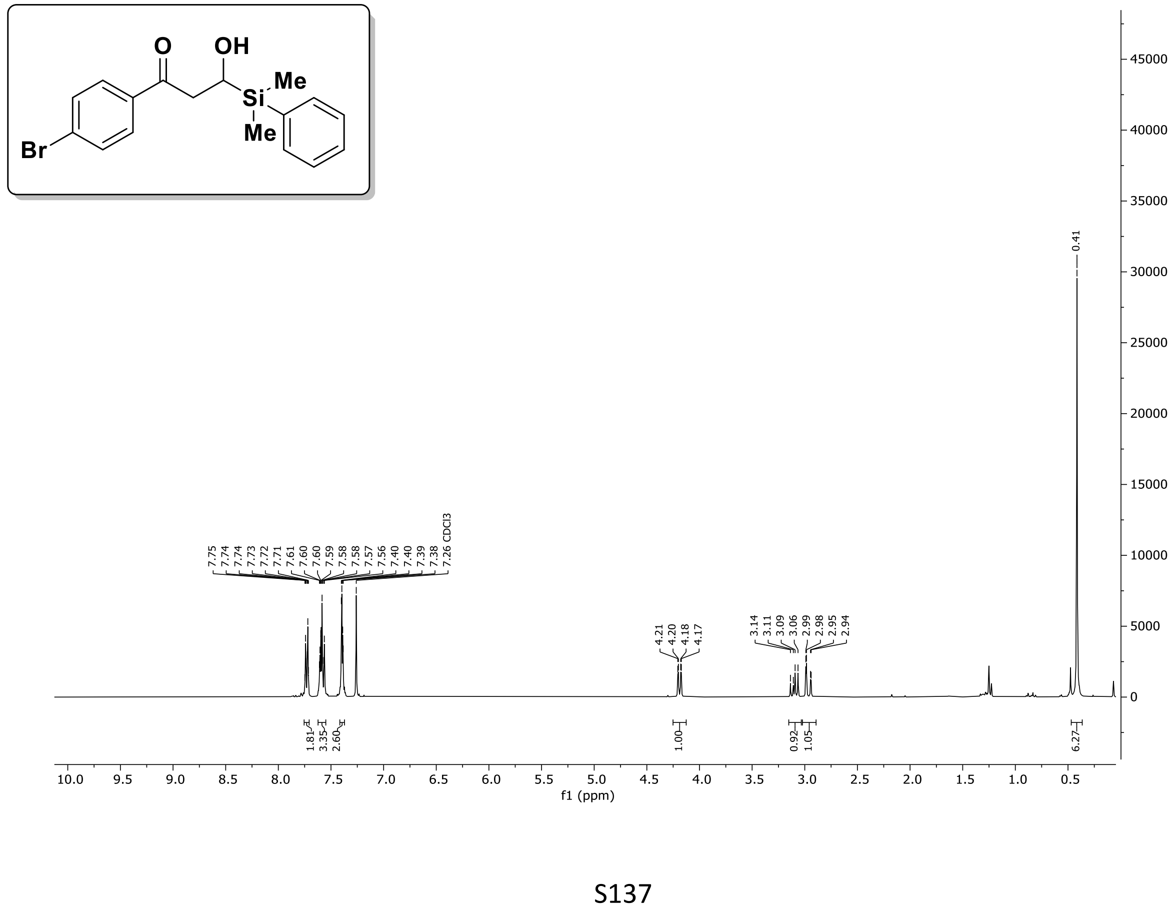

^{13}C NMR of 40 (151 MHz, $CDCl_3$)

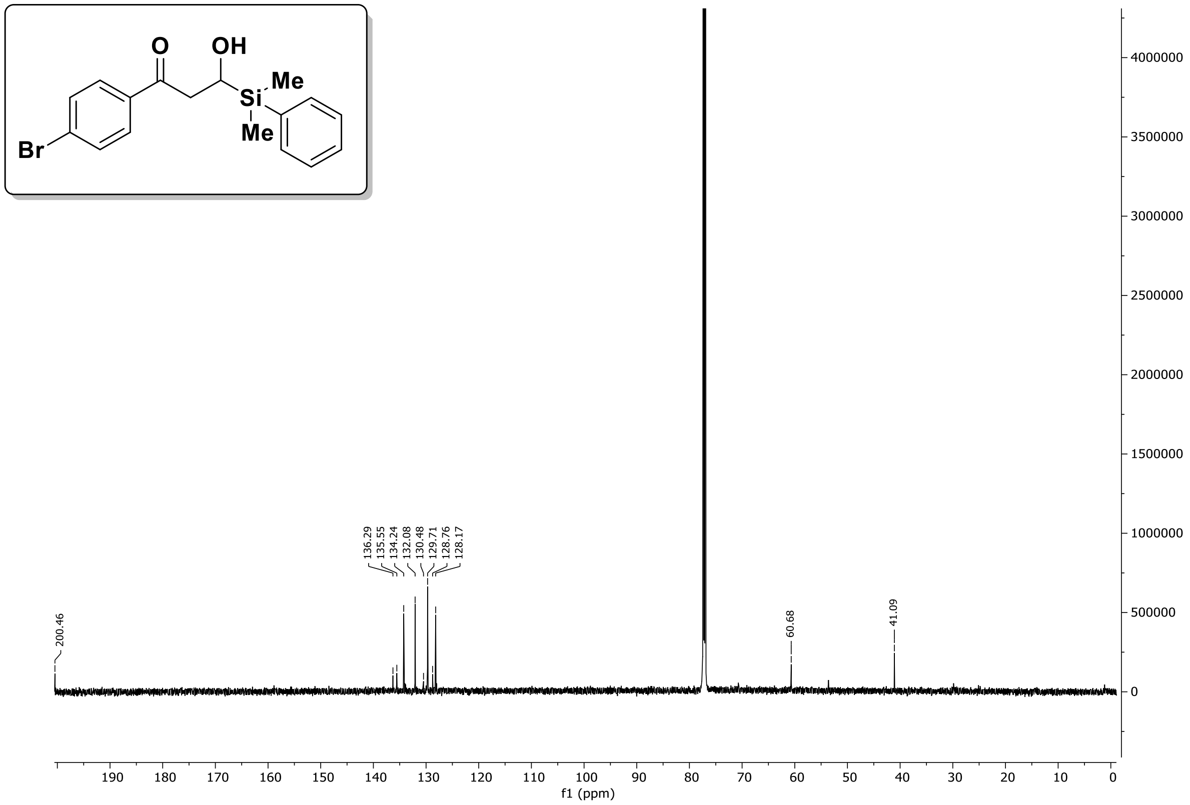

^{1}H NMR of 41 (600 MHz, $CDCl_3$)

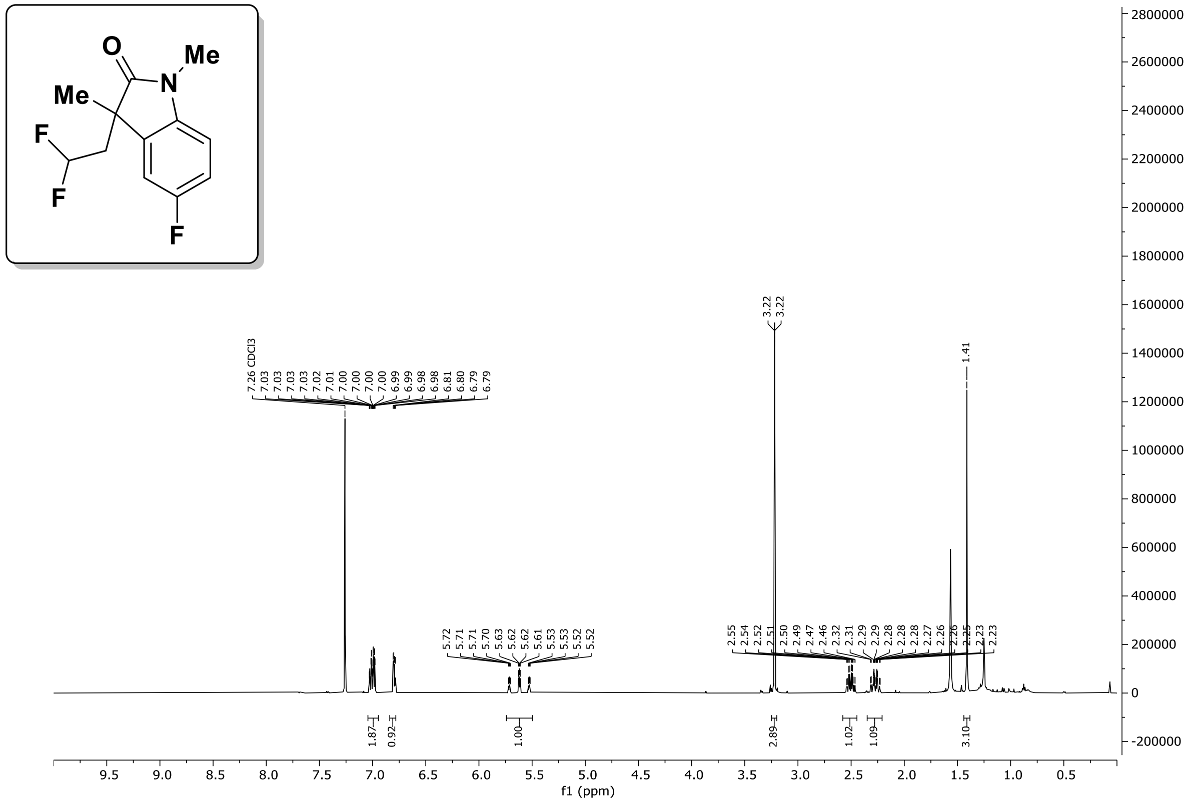

^{13}C NMR of 41 (151 MHz, $CDCl_3$)

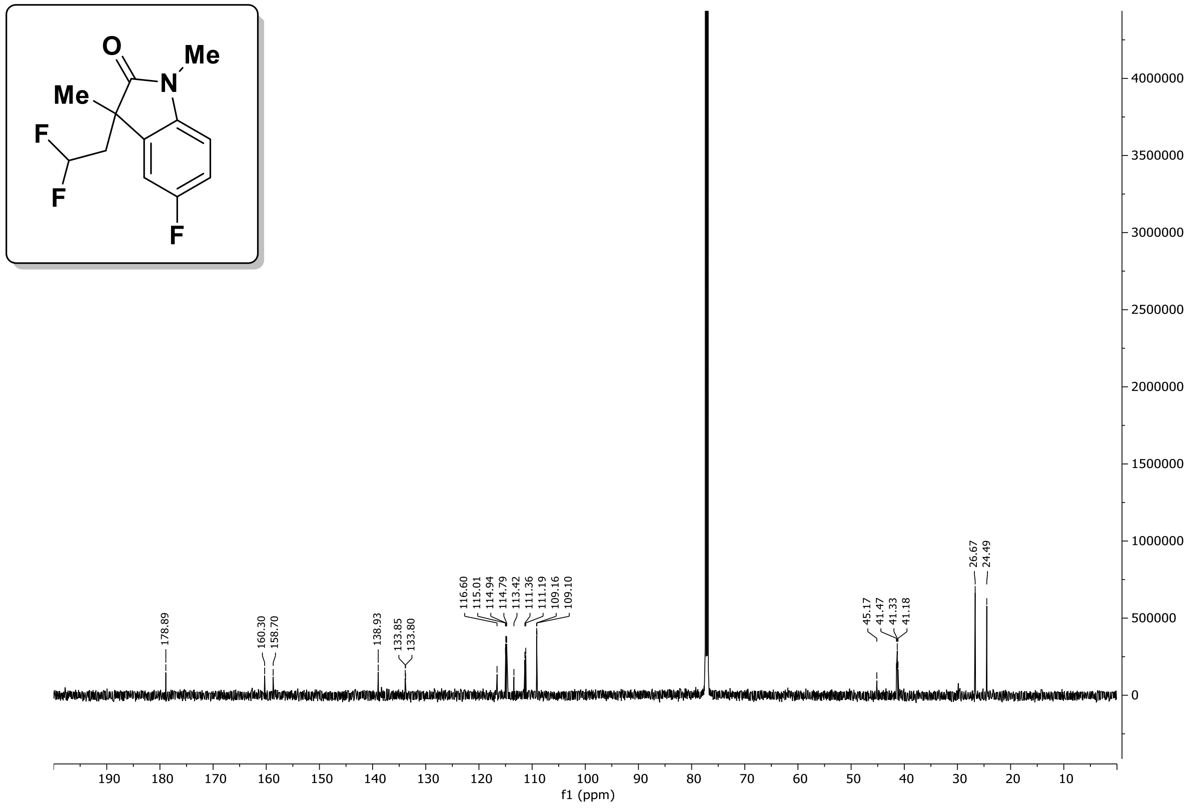

^{19}F NMR of 41 (376 MHz, $CDCl_3$)

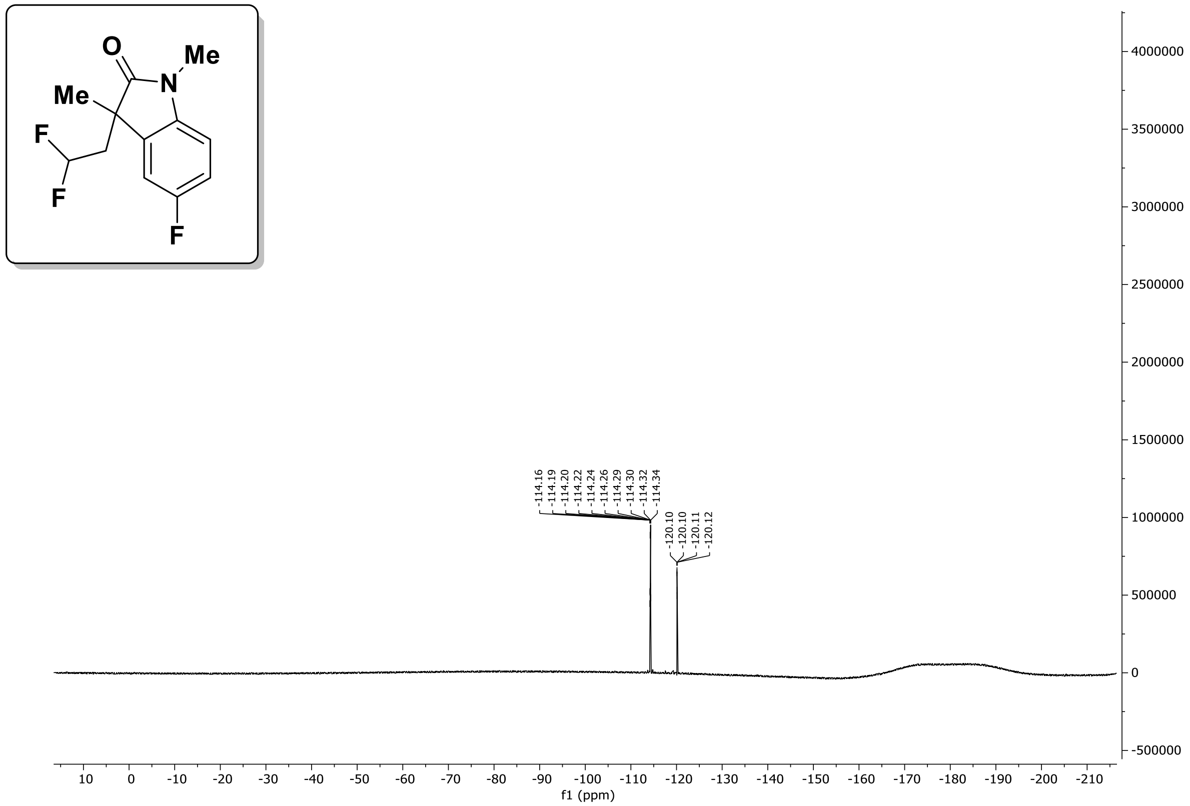

^{1}H NMR of 42 (600 MHz, $CDCl_3$)

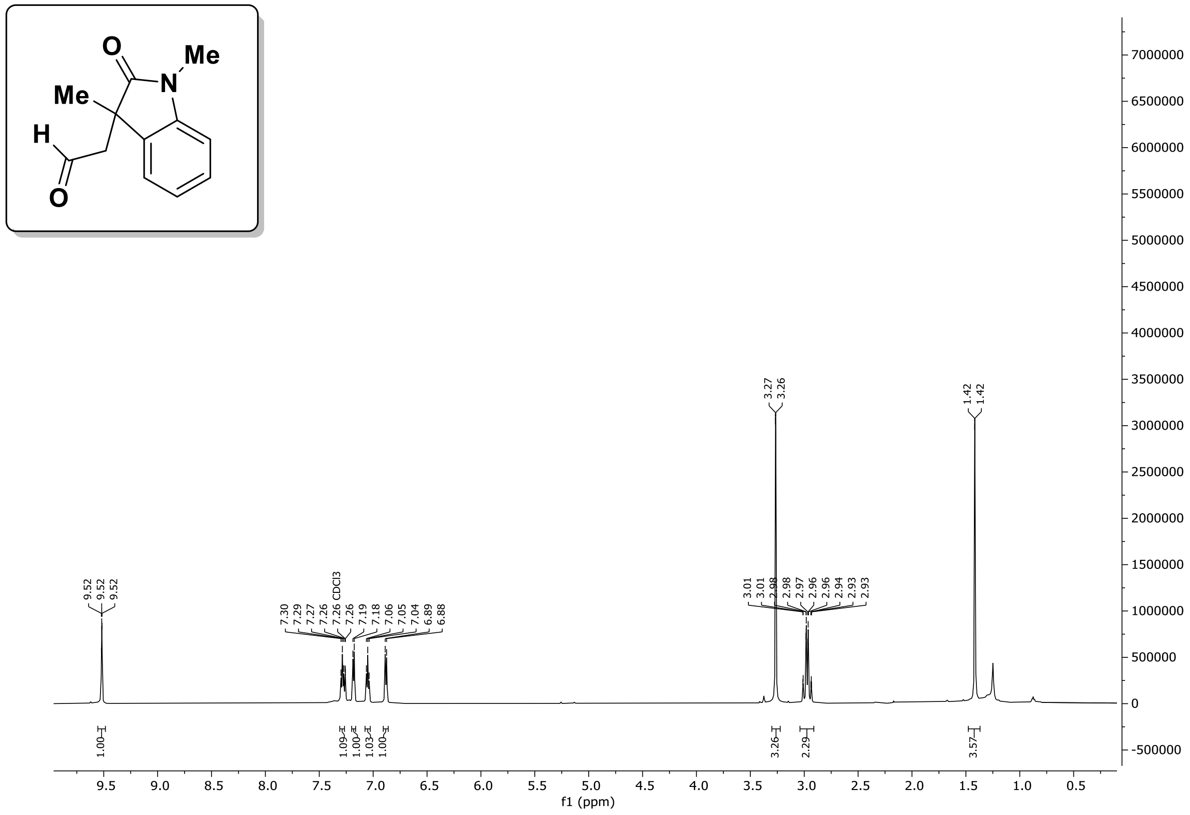

^{13}C NMR of **42** (151 MHz, $CDCl_3$)

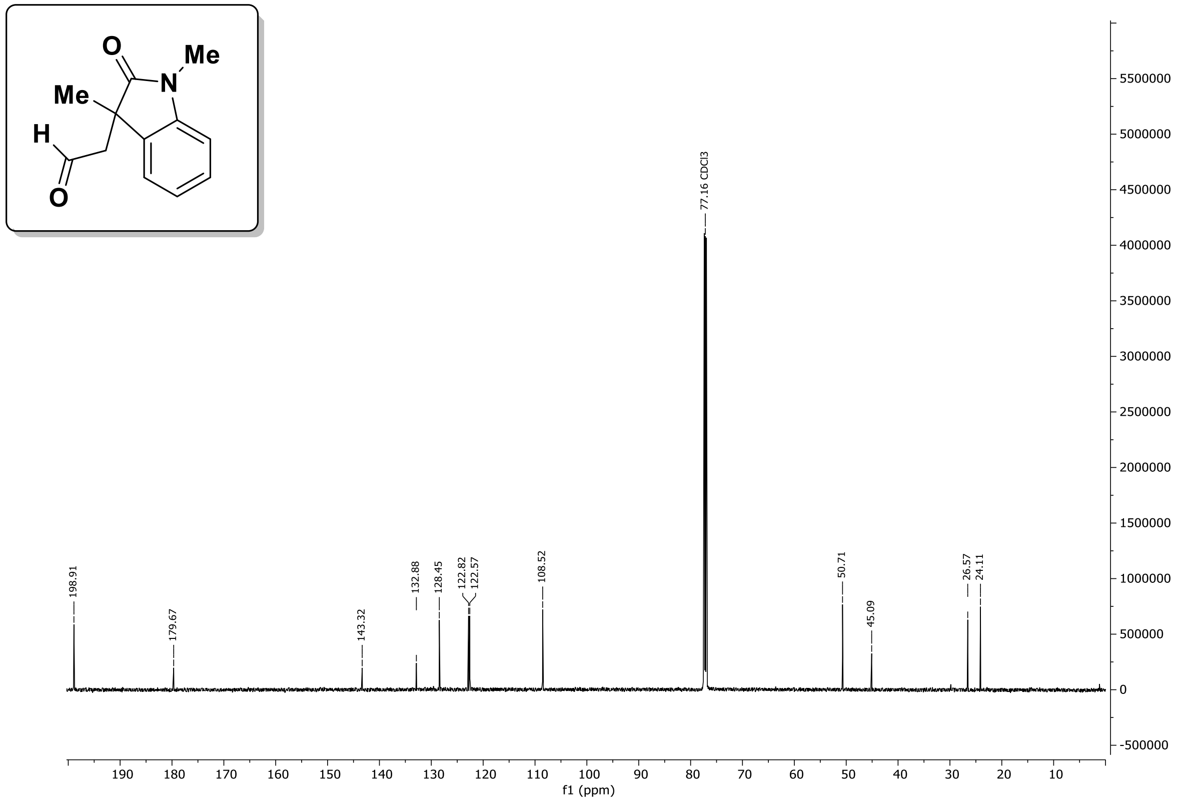

Supplement: Supplementary file 1 — ja4c02261_si_001.pdf [file ja4c02261_si_001.pdf]
